# Supplementary material for: N-Heterocyclic carbene-catalyzed enantioselective synthesis of planar-chiral cyclophanes via dynamic kinetic resolution
Source: Nat Commun. 2024 Mar 15;15:2338. doi: 10.1038/s41467-024-46376-8 (PMC10943026; doi:10.1038/s41467-024-46376-8)
Supplement: Supplementary file 1 — Supplementary Information [file 41467_2024_46376_MOESM1_ESM.pdf]

Supplementary Information

N-Heterocyclic Carbene-Catalyzed Enantioselective Synthesis of Planar-Chiral Cyclophanes via Dynamic Kinetic Resolution

Jiayan Li,[#1] Ziyang Dong,[#1] Yang Chen,[2] Zhanhui Yang,[2] Xinen, Yan,[1] Meng, Wang,[3] Chenyang, Li,[*3] and Changgui Zhao[*1]

1 Key Laboratory of Radiopharmaceuticals, Ministry of Education, College of Chemistry, Beijing Normal University, Beijing 100875, China.

2 Department of Organic Chemistry, College of Chemistry, Beijing University of Chemical Technology, Beijing 100029, China

3 Key Laboratory of Theoretical and Computational Photochemistry, Ministry of Education, College of Chemistry, Beijing Normal University, Beijing 100875, China.

[#]These authors contributed equally to this work

* Correspondence: cgzhao@bnu.edu.cn and chenyang.li@bnu.edu.cn

Table of Contents

1. Supplementary Methods......3
1.1 General remarks......3
1.2 Methods and procedures......3
1.2.1 Procedures for the synthesis of 2,5-dimethoxy-benzaldehyde derivatives......3
1.2.2 Procedures for the synthesis of 2,5-dihydroxy-benzaldehyde derivatives......4
1.2.3 Procedures for the synthesis of dibromides chains...... 4
1.2.4 Procedures for the synthesis of macrocycle substrates **1a-1i**, **4a**, **4c**, **4n**, **4o**......5
1.2.5 Procedures for the synthesis of macrocycle substrates **4m**......6
1.2.6 Procedures for the synthesis of macrocycle substrates **1k**, **4f**, **4g**, **4h**, **4i**......6
1.2.7 Procedures for the synthesis of macrocycle substrates **4b** and **4e**......6
1.2.8 Procedures for the synthesis of macrocycle substrates **1l** and **4d**......7
1.2.9 Procedures for the NHC-catalyzed enantioselective synthesis of planar-chiral cyclophanes......7
1.3 Synthetic transformation of enantioenriched [14]paracyclophane **3ae**......8
1.4 Racemization experiment......11
1.5 Controlled experiments......13
1.6 DFT Calculations......14
2. Supplementary Notes......17
2.1 Characterization data......17
2.1.1 Characterization data of unknown macrocycles substrates......17
2.1.2 Characterization data of [n]paracyclophanes......24
2.2 X-Ray report......41
2.3 Copies of NMR spectra......43
2.4 Copies of HPLC spectra......102
3. Supplementary References......144

1. Supplementary Methods

1.1 General remarks

All the commercially available reagents were obtained from commercial suppliers and used without further purification. All the catalytic experiments were carried out using standard techniques. Chromatography was carried out over silica gel (Innochem 200 ~ 300 mesh) and TLC was performed using silica gel 60 F254 (Merck) plates. ^{1}H NMR (400 or 600 MHz), ^{13}C NMR (100 or 150 MHz) and ^{31}P NMR spectra were recorded on a JEOL Delta spectrometer in $CDCl_3$ using TMS as an internal reference with chemical shift values reported in ppm. Abbreviations used in the NMR follow-up experiments: s, singlet; d, doublet; t, triplet; q, quartet; m, multiplet. HPLC analysis was conducted on SHIMADZU LC-20ADXR instrument with chiral columns (Chiralpak IB N-5 and IA, column 4.6 × 250 mm, (Daicel Chemical Ind., Ltd.)). High resolution mass spectra (HRMS) were recorded on a Waters LCT Premier XE mass spectrometer with TOF. Crystallographic data were collected using a Rigaku Oxford Diffraction XtaLAB Synergy diffractometer equipped with a HyPix-6000E area detector at 100 K using Cu Kα (λ = 1.54184 Å) from a PhotonJet micro-focus X-ray source.

1.2 Methods and procedures

1.2.1 Procedures for the synthesis of 2,5-dimethoxy-benzaldehyde derivatives

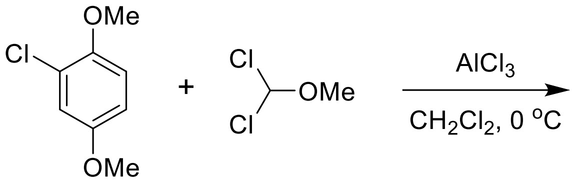

S1 **S2** **S3**

To a solution of **S1** (10.3 g, 60.0 mmol) in dry CH_2Cl_2 (250 mL) was added $AlCl_3$ (19.8 g, 150 mmol), and the mixture was cooled to 0 °C. Then, 1,1'-dichlorodimethyl ether **S2** (9.6 g, 84.0 mmol) was added slowly through a syringe, and the mixture was stirred at 0 °C for 2 h. Afterward, the reaction mixture was quenched carefully with ice water, acidified by dilute hydrochloric acid, and extracted with CH_2Cl_2. The combined organic phases were washed by brine (30 mL) and dried over anhydrous Na_2SO_4. After filtration and removing the solvent in vacuum, the residue was purified by flash column chromatography on silica gel to afford the product **S3**.

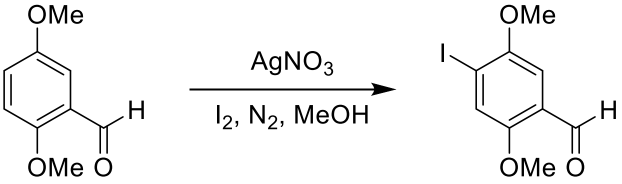

S4 **S5**

To a solution of 2,5-dimethoxybenzaldehyde **S4** (10.2 g, 61.4 mmol) in MeOH (250 mL) was added $AgNO_3$ (10.4 g, 61.4 mmol) and I_2 (16.2 g, 64.0 mmol) under nitrogen. The reaction was stirred overnight, then filtered the yellow precipitate and washed the solid with MeOH. The solution

was washed by saturated sodium bisulfite solution. Solvent was removed under reduced pressure, and the residue was recrystallized from ethanol to afford 4-iodo-2,5-dimethoxybenzaldehyde **S5**.[1]

1.2.2 Procedures for the synthesis of 2,5-dihydroxy-benzaldehyde derivatives

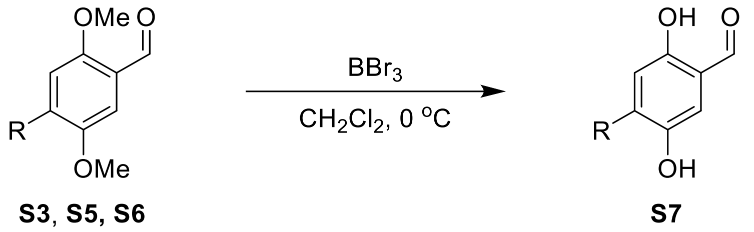

To a solution of 2,5-dihydroxy-benzaldehyde derivatives (10.0 mmol) in dry CH_2Cl_2 (100 mL) was added BBr_3 (12.0 mmol) dropwise at 0 °C. The reaction mixture was stirred for 6 hours and quenched carefully by water. The aqueous phase was extracted with CH_2Cl_2 three times. The combined organic phase was washed by brine and dried over anhydrous Na_2SO_4, and then concentrated to give the 2,5-dihydroxy-benzaldehyde derivatives **S7**, which was used for next step directly.

1.2.3 Procedures for the synthesis of dibromides chains

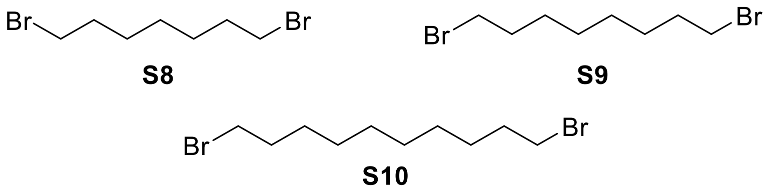

Known dibromides **S8-S10** were obtained from commercial suppliers and used without further purification.

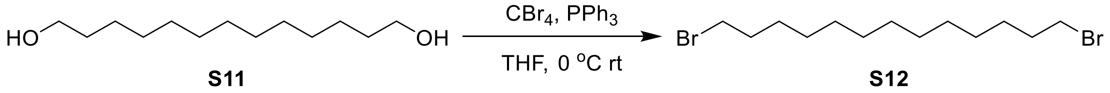

To a solution of **S11** (2.16 g, 10.0 mmol) in dry CH_2Cl_2 (200 mL) was addted CBr_4 (9.81 g, 30.0 mmol) and PPh_3 (7.86 g, 30.0 mmol) at 0 °C. The solution was stirred at room temperature overnight, then washed with saturated $NaHCO_3$ solution and brine, and dried over Na_2SO_4. The solvent was removed under reduced pressure, and the crude product was purified by flash column chromatography on the silica gel to give product **S12**.[2]

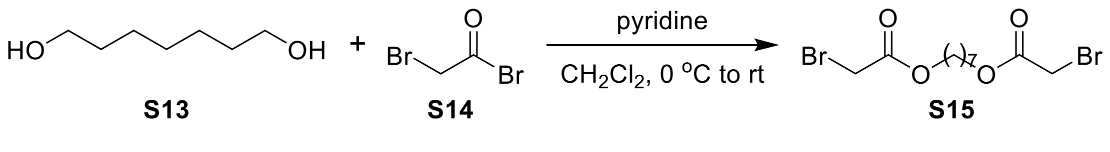

To a solution of heptane-1,7-diol **S13** (1.0 mL, 15.0 mmol) in dry CH_2Cl_2 (200 mL) was added pyridine (3.0 mL, 37.5 mmol) at 0 °C under argon atmosphere, and bromoacetyl bromide **S14** (4.0 mL, 45.0 mmol) was added dropwise to the reaction mixtures over 30 mins. Then, the reaction mixtures were stirred at 0 °C for 40 mins and warmed to room temperature for 1 h. The reaction

mixtures were quenched by H_2O (30 mL), and extracted with CH_2Cl_2 (3 × 50 mL). The combined organic layers was washed by brine, dried over anhydrous Na_2SO_4, concentrated to give a crude product, which was purified by flash column chromatography on the silica gel to give **S15**.[3]

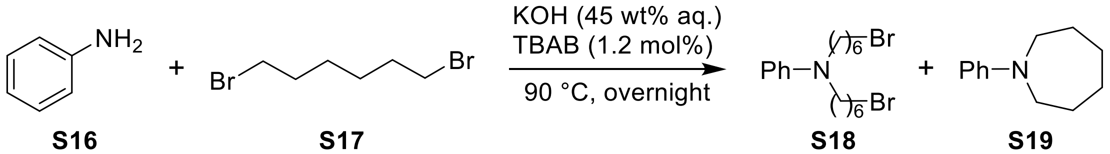

To a 100 mL flask was successively added aniline **S16** (1.0 g, 10.0 mmol), TBAB, (40 mg, 0.124 mmol), 1,6-dibromohexane **S17** (40 mL) and KOH aqueous solution (45 wt%, 30 mL). The mixture was stirred at 90 °C overnight. After cooling to room temperature, the reaction mixture was diluted with water (100 mL) and extracted with EtOAc (3 × 50 mL). The combined organic layers were dried over anhydrous Na_2SO_4. After filtration and removing EtOAc in vacuum, the remaining 1,6-dibromohexane was distilled out under vacuum. The residue was purified by flash column chromatography on silica gel affording a mixture of **S19** (0.94 mmol, 9.4% yield) and dibromides **S18** (4.60 mmol, 46% yield) as light brown oil. The total mass of **S18** and **S19** was 2.09 g, and the mole ratio of **S18** and **S19** was determined by ^{1}H NMR of the mixture.The procedure for the synthesis of dibromide **S18** was modified by the reported method.[4]

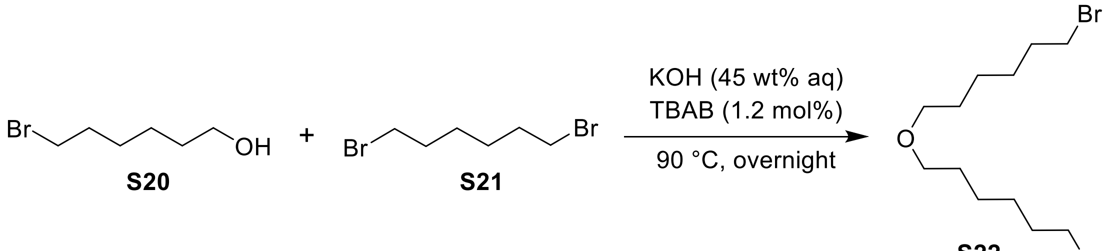

To a 100 mL-flask was successively added 6-bromohexan-1-ol **S20** (3.62 g, 20.0 mmol), TBAB (80 mg, 0.248 mmol, 0.012 eq), 1,6-dibromohexane (80 mL) and KOH aqueous solution (45 wt%, 60 mL). The mixture was stirred at 90 °C overnight. After cooling to room temperature, the reaction mixture was diluted with water (200 mL) and extracted with EtOAc (3 × 100 mL). The combined organic layers were dried over anhydrous Na_2SO_4. After filtration and removing EtOAc in vacuum, the remaining 1,6-dibromohexane was distilled out under vacuum. the residue was purified by flash column chromatography on silica gel affording the product **S22**.The procedure for the synthesis of dibromide **S22** was modified by the reported method.[4]

1.2.4 Procedures for the synthesis of macrocycle substrates 1a-1i, 4a, 4c, 4n, 4o

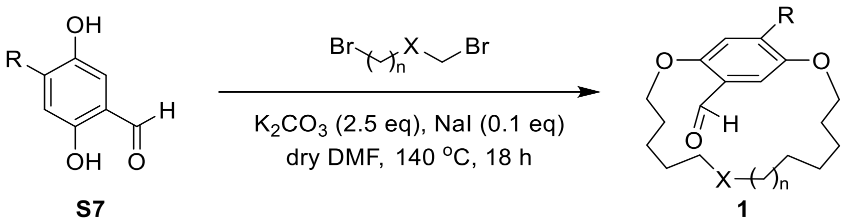

S7 (10.0 mmol) and dibromides (10.0 mmol) in DMF (25.0 mL) were slowly added to a suspension of K_2CO_3 (3.45 g, 25.0 mmol) and NaI (170 mg, 1.0 mmol) in DMF (150 mL) at 140 °C

over 18 h. The solvent was removed under reduced pressure. The aqueous phase was extracted with EtOAc (3 × 50 mL), the combined organic layers were washed water and brine, and dried over Na_2SO_4. The solvent was removed under reduced pressure, and the residue were purified by flash column chromatography on silica gel affording the product **1** (yield: 10~40%).

1.2.5 Procedures for the synthesis of macrocycle substrates 4m

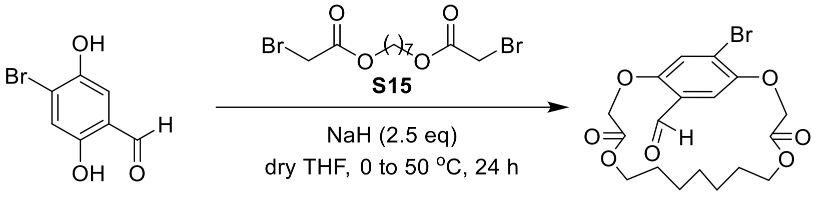

4-bromo-2,5-dihydroxybenzaldehyde **S7** (645 mg, 3.0 mmol) and NaH (60 mg, 7.5 mmol, 60% dispersion in mineral oil) were dissolved in dry THF (50 mL) at 0 °C. Then dibromides **S7** (1.3 g, 3.0 mmol) was slowly added to the reaction mixture. The reaction was quickly heated to 50 °C and stirred for 24 h. After cooling to room temperature, saturated NH_4Cl (5 mL) was added. Then the reaction mixture was diluted with water (20 mL) and extracted with EtOAc (3 × 20 mL). The combined organic layers were dried over anhydrous Na_2SO_4 and concentrated under vacuum, the residue was purified by flash column chromatography on silica gel to give the product **4m** (yield: 22%).

1.2.6 Procedures for the synthesis of macrocycle substrates 1k, 4f, 4g, 4h, 4i

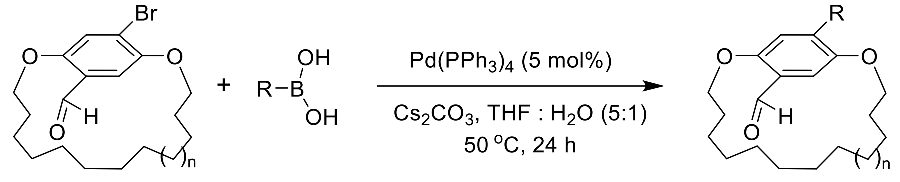

1 (1.0 mmol), boronic acid (3.0 mmol), $Pd(PPh_3)_4$ (57.8 mg, 0.05 mmol) and Cs_2CO_3 (977.5 mg, 3.0 mmol) were added to a dried flask. The flask was evacuated and backfilled with N_2 three times. Then the degassed THF:H_2O (12 mL, 5:1) was added to the reaction. The reaction was stirred for 24 h at 50 °C. After cooling to room temperature, diluted with water (20 mL) and extracted with EtOAc (3 × 20 mL). The combined organic layers were dried over anhydrous Na_2SO_4. After filtration and removing EtOAc in vacuum, the residue was purified by flash column chromatography on silica gel affording the product **1k, 4f, 4g, 4h, 4i** (yield: 80~95%).

1.2.7 Procedures for the synthesis of macrocycle substrates 4b and 4e

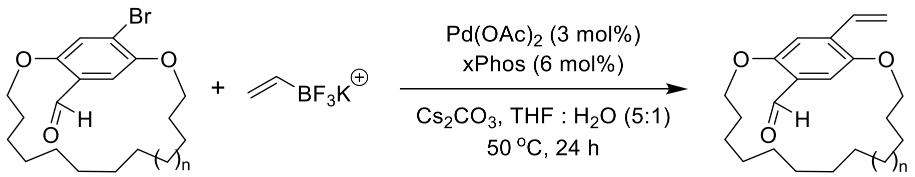

1 (1.0 mmol), Potassium vinyltrifluoroborate (446 mg, 3.0 mmol), $Pd(OAc)_2$ (5 mg, 0.03 mmol), xPhos (28.6 mg, 0.06 mmol) and Cs_2CO_3 (977.5 mg, 3.0 mmol) were added to a dried flask. The flask was evacuated and backfilled with N_2 three times. Then the degassed THF:H_2O (12 mL, 5:1) was added to the reaction. The reaction was stirred for 24 h at 50 °C. After cooling to room temperature, the reaction mixture was diluted with water (20 mL) and extracted with EtOAc (3 × 20 mL). The combined organic layers were dried over anhydrous Na_2SO_4. After filtration and removing EtOAc in vacuum, the residue was purified by flash column chromatography on silica gel to afford the product **4b** or **4e** (yield: 90%).

1.2.8 Procedures for the synthesis of macrocycle substrates 1l and 4d

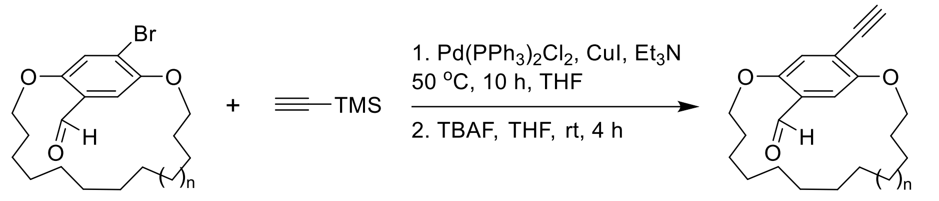

1a or **1e** **1l** or **4d**

1 (1.0 mmol), TMS-acetylene (3.0 mmol), $Pd(PPh_3)_2Cl_2$ (70.0 mg, 0.1 mmol) and CuI (19.0 mg, 0.1 mmol) were added to a flask. The flask was evacuated and backfilled with N_2 three times. Et_3N:THF (1:1, 3 mL) was added to the above flask. The reaction mixture was stirred for 10 h at 50 °C. The solution was then cooled to room temperature and quenched with water (5 mL). The crude mixture was diluted with ethyl acetate (10 mL), washed with brine (10 mL) for three times, dried over Na_2SO_4, the solvent was evaporated under reduced pressure. The residue was purified by column chromatography using silica gel to afford the product.

To the above product in THF (10 mL) was added TBAF (2.0 mL, 1.0 M in THF) at 0 °C. The reaction mixture was stirred at room temperature for 4 h. After completion, the reaction was quenched with NH_4Cl (aq), and the solution was extracted with ethyl acetate, the combined organic phase was washed with brine, dried over Na_2SO_4, the solvent was evaporated under reduced pressure. The residue was purified by column chromatography using silica gel to afford **1l** or **4d** (yield: 50~60%).

1.2.9 Procedures for the NHC-catalyzed enantioselective synthesis of planar chiral cyclophanes

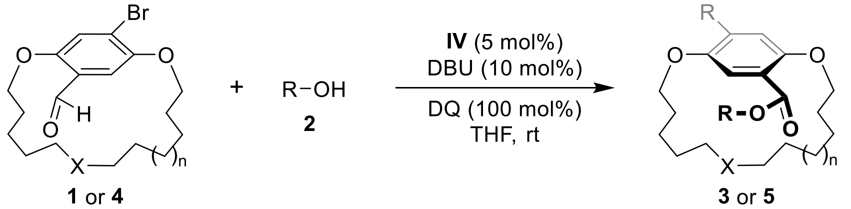

1 or **4** **3** or **5**

To a 15 mL schlenk tube equipped with a magnetic stirring bar was added macrocycles substrates **1** or **4** (0.10 mmol), **2** (0.4 mmol), NHC precursor **IV** or **V** (0.005 mmol), DBU (0.01 mmol) and DQ (0.1 mmol). The tube was closed with a septum, evacuated, and refilled with nitrogen (3 cycles). After that, freshly distilled THF (1.0 mL) was added to the reaction mixture and stirred for 10-72 h. Upon the completion (monitored by TLC), the solvent was evaporated, and the residue

was purified by silica gel column chromatography to afford the planar chiral cyclophanes **3** or **5**. **Note**: Racemic samples for the standard of chiral HPLC spectra were prepared using racemic NHC precursor **S23** as catalyst.

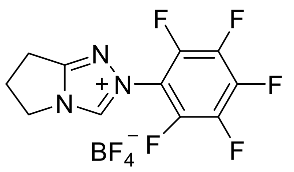

S23

1.3 Synthetic transformation of enantioenriched [14]paracyclophane 3ae

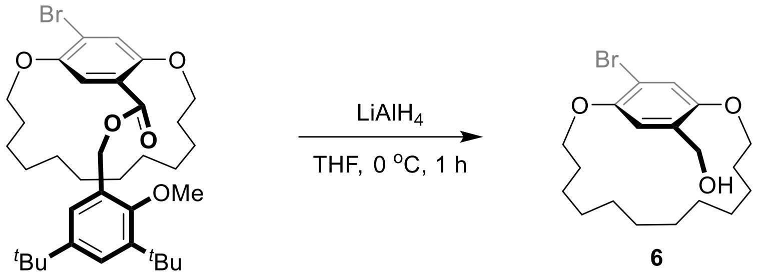

3ae **6**

To a solution of **3ae** (0.1 mmol) in anhydrous THF (1.0 mL) was added $LiAlH_4$ (0.4 mL, 2.5 M in THF) at 0 °C under N_2. Then, the reaction mixture was stirred at the same temperature for 1 h, and quenched with $NaHCO_3$ (aq). The solution was filtered, and the solid was washed by ethyl acetate. The combine organic layers were dried over anhydrous Na_2SO_4. The solvent was evaporated under reduced pressure, and the crude mixture was purified by column chromatography using silica gel to afford the corresponding product **6**. Colorless oil, 37.6 mg, 98% yield. **^{1}H NMR** (600 MHz, $CDCl_3$) δ 7.07 (s, 1H), 6.96 (s, 1H), 4.71 (d, J = 13.2 Hz, 1H), 4.51 (d, J = 13.2 Hz, 1H), 4.27 – 4.33 (m, 2H), 4.20 (ddd, J = 12.2, 8.5, 4.1 Hz, 1H), 4.11 (ddd, J = 12.1, 8.5, 4.1 Hz, 1H), 2.41 (s, 1H), 1.77 – 1.84 (m, 1H), 1.67 – 1.74 (m, 1H), 1.50 – 1.58 (m, 2H), 1.23 – 1.40 (m, 4H), 1.11 – 1.22 (m, 4H), 0.82 – 0.99 (m, 8H). **^{13}C NMR** (150 MHz, $CDCl_3$) δ 149.9, 148.6, 148.58, 130.4, 118.3, 116.3, 112.1, 69.5, 68.2, 61.6, 28.6, 28.1, 28.0, 27.6, 27.5, 27.13, 27.11, 26.92, 26.91, 24.03, 23.96. **HRMS** (ESI): m/z calculated for $C_{19}H_{29}BrNaO_3^+$ [M + Na]$^+$ 407.1192, found 407.1190. $[\alpha]_D^{25}$ = – 9.2 (c = 1.7, CH_2Cl_2). **HPLC**: Chiralpak IB N-5 column, 95:5 hexanes/isopropanol, 1.0 mL/min; t_R = 12.7 min (major), 17.2 min (minor), 91:9 er.

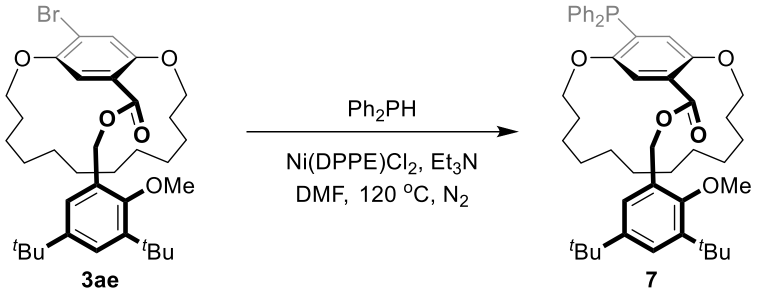

3ae **7**

A solution of Ni(DPPE)Cl_2 (11 mg, 0.01 mmol) and Ph_2PH (70 mg, 0.3 mmol) in DMF (1.0 mL) was stirred at 120 °C for 10 min under N_2. To this mixture was added a solution of **3ae** (0.1 mmol) and Et_3N (225 μL) in DMF (2 mL). Then, the mixture was heated at 120 °C for 6 h. The solution was then cooled to room temperature and quenched with water (15 mL). The crude mixture was diluted with ethyl acetate (30 mL), washed with brine (10 mL), dried over Na_2SO_4, the solvent was evaporated under reduced pressure. The crude mixture was purified by column chromatography using silica gel to afford the corresponding products **7**. Colorless oil, 44.2 mg, 61% yield. **^{1}H NMR** (400 MHz, $CDCl_3$) δ 7.29 – 7.39 (m, 14H), 4.40 – 4.45 (m, 2H), 4.29 – 4.34 (m, 1H), 4.05 – 4.12 (m, 1H), 3.85 – 3.88 (m, 5H), 1.41 (s, 9H), 1.30 (s, 9H), 1.18 – 1.24 (m, 5H), 0.86 – 1.13 (m, 15H). **^{13}C NMR** (100 MHz, $CDCl_3$) δ 166.3, 156.4, 153.09 (d, J = 63.2 Hz), 151.6, 145.8, 142.0, 134.5 (d, J = 81.6 Hz), 133.5 (d, J = 79.6 Hz), 129.2, 128.8 (d, J = 7.6 Hz), 128.7, 128.6, 128.5, 126.0, 124.6, 122.3, 122.2, 115.42, 115.40, 69.1, 68.4, 63.1, 62.8, 35.5, 34.7, 31.7, 31.2, 28.8, 28.7, 28.12, 28.07, 27.7, 27.13, 27.06, 26.7, 24.0, 23.8. **^{31}P NMR** (162 MHz, $CDCl_3$) δ –15.8. **HRMS** (ESI): m/z calculated for $C_{47}H_{61}NaO_5P^+$ $[M + Na]^+$ 759.4149, found 759.4140. $[\alpha]_D^{25}$ = 15.0 (c = 0.1, CH_2Cl_2). **HPLC**: Chiralpak IB N-5 column, 95:5 hexanes/isopropanol, 1.0 mL/min; t_R = 4.3 min (major), 8.5 min (minor), 90:10 er.

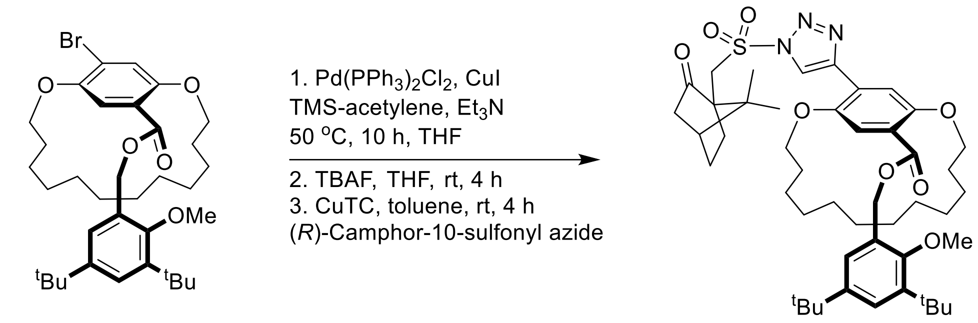

3ae (315 mg, 0.5 mmol), TMS-acetylene (150 mg, 1.5 mmol), $Pd(PPh_3)_2Cl$ (35.0 mg, 0.05 mmol) and CuI (9.5 mg, 0.05 mmol) were added to a flask. The flask was evacuated and backfilled with N_2 three times. Et_3N:THF (1:1, 20 mL) was added to the above flask. The reaction mixture was stirred for 10 h at 50 °C. The solution was then cooled to room temperature and quenched with water (15 mL). The crude mixture was extracted with ethyl acetate (20 mL), washed with brine (10 mL) for three times, dried over Na_2SO_4, the solvent was evaporated under reduced pressure. The crude mixture was purified by column chromatography using silica gel to afford the product (91% yield, 93:7 er.).

To the above product (78 mg, 0.12 mmol, 1.0 eq) in THF (10 mL) was added (TBAF·$3H_2O$) (45 mg, 0.144 mmol, 1.2 eq) at 0 °C. The reaction mixture was stirred at room temperature for 4 h. After completion, the reaction was quenched NH_4Cl (aq), and the solution was extracted with ethyl acetate, the combined organic phase was washed with brine, dried over Na_2SO_4, the solvent was evaporated under reduced pressure. The residue was purified by column chromatography using silica gel to afford the product (87% yield, 93:7 er.).

To the above product in toluene (5.0 mL) was successively added (*R*)-Camphor-10-sulfonyl azide (51 mg, 0.2 mmol),[5] Copper(I) thiophene-2-carboxylate (CuTC) (1.9 mg, 0.01 mmol). The reaction mixture was allowed to stir at room temperature for 4 h. Removing the volatiles in vacuum and purifying the residue on column chromatography on silica gel afforded the product **8**. White

solid, 80 mg, 96% yield, *dr* = 13:1 (determined by ^{1}H NMR). **^{1}H NMR** (600 MHz, $CDCl_3$) δ 8.68 (s, 1H), 8.09 (s, 1H), 7.52 (s, 1H), 7.38 (d, *J* = 2.5 Hz, 1H), 7.34 (d, *J* = 2.5 Hz, 1H), 5.46 (d, *J* = 12.4 Hz, 1H), 5.44 (d, *J* = 12.4 Hz, 1H), 4.47 – 4.55 (m, 2H), 4.26 – 4.30 (m, 1H), 4.19 – 4.23 (m, 1H), 3.99 (d, *J* = 14.9 Hz, 1H), 3.88 (s, 3H), 3.65 (d, *J* = 14.9 Hz, 1H), 2.39 (dt, *J* = 18.6, 3.9 Hz, 1H), 2.33 – 2.82 (m, 1H), 2.16 (t, *J* = 4.5 Hz, 1H), 2.05 – 2.11 (m, 1H), 1.96 (d, *J* = 18.6 Hz, 1H), 1.78 – 1.89 (m, 3H), 0.69 – 1.59 (m, 43H). **^{13}C NMR** (150 MHz, $CDCl_3$) δ 212.9, 166.0, 156.3, 152.2, 147.7, 145.8, 142.1, 142.0, 128.6, 125.9, 124.6, 123.5, 123.1, 121.9, 117.0, 115.4, 69.3, 68.4, 63.2, 62.7, 58.9, 53.7, 48.5, 42.8, 42.4, 35.4, 34.6, 31.6, 31.2, 28.5, 28.5, 28.0, 27.8, 27.53, 27.49, 27.12, 27.06, 26.7, 25.2, 23.9, 19.74, 19.71. **HRMS** (ESI): m/z calculated for $C_{47}H_{68}N_3O_8S^+$ [M + H]$^+$ 834.4722, found 834.4738. $[\alpha]_D^{25}$ = 7.3 (c = 2.00, CH_2Cl_2).

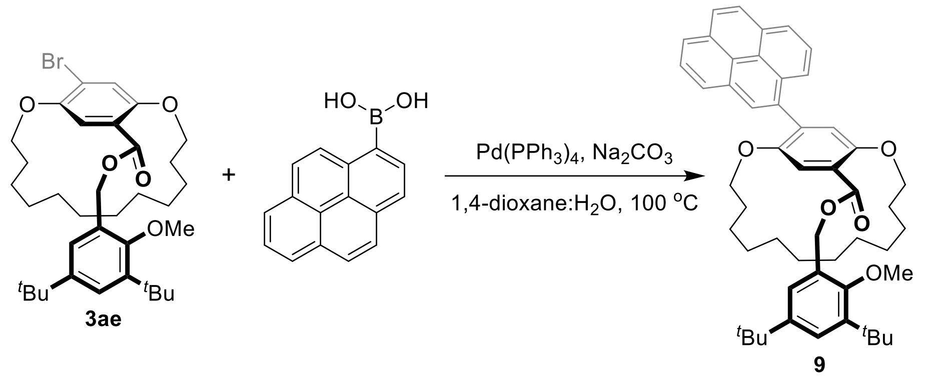

3ae (63 mg, 0.1 mmol), boronic acid (75.0 mg, 0.30 mmol), $Pd(PPh_3)_4$ (11.55 mg, 0.01 mmol) and Na_2CO_3 (31.8 mg, 0.30 mmol) were added to a flask. The flask was evacuated and backfilled with N_2 three times. Degassed 1,4-dioxane:H_2O (10:1, 2.0 mL) was added to the above flask. The reaction mixture was stirred for 3 h at 100 °C. The solution was then cooled to room temperature and quenched with water (15 mL). The crude mixture was extracted with ethyl acetate (50 mL), washed with brine (10 mL), dried over Na_2SO_4, the solvent was evaporated under reduced pressure. The crude mixture was purified by column chromatography using silica gel to afford the product **9**. Colorless oil, 51.2 mg, 68% yield. **^{1}H NMR** (600 MHz, $CDCl_3$) δ 8.17 – 8.27 (m, 3H), 8.11 – 8.14 (m, 3H), 8.00 – 8.06 (m, 3H), 7.87 – 7.97 (m, 1H), 7.70 (d, *J* = 32.4 Hz, 1H), 7.49 (dd, *J* = 4.2, 2.4 Hz, 1H), 7.41 (d, *J* = 2.4 Hz, 1H), 7.12 (d, *J* = 21.6 Hz, 1H), 5.52 – 5.58 (m, 2H), 4.34 – 4.42 (m, 1H), 4.08 – 4.22 (m, 2H), 3.96 (d, *J* = 1.8 Hz, 3H), 3.70 – 3.75 (m, 1H), 1.86 – 1.99 (m, 1H), 1.55 – 1.68 (m, 3H), 1.47 (s, 9H), 1.38 (s, 9H), 1.04 – 1.31 (m, 16H). **^{13}C NMR** (150 MHz, $CDCl_3$) δ 166.3, 166.2, 156.5, 156.4, 152.3, 151.7, 149.7, 149.4, 145.9, 142.1, 136.6, 136.3, 133.7, 133.3, 131.52, 131.47, 131.16, 131.12, 130.97, 130.95, 129.3, 128.84, 128.82, 128.79, 128.4, 127.73, 127.66, 127.63, 127.57, 127.5, 127.4, 127.2, 126.22, 126.16, 126.1, 126.0, 125.5, 125.3, 125.22, 125.16, 125.1, 125.0, 124.8, 124.72, 124.69, 124.54, 124.51, 121.3, 121.0, 120.5, 119.8, 118.2, 69.3, 69.0, 68.5, 63.2, 62.9, 62.8, 35.5, 34.7, 31.7, 31.3, 28.81, 28.80, 28.75, 28.4, 28.3, 28.1, 28.0, 27.9, 27.8, 27.6, 27.5, 27.3, 27.1, 27.0, 24.2, 24.1, 23.8, 23.7. **HRMS** (ESI): m/z calculated for $C_{51}H_{60}NaO_5^+$ [M + Na]$^+$ 775.4333, found 775.4343. $[\alpha]_D^{25}$ = 66.7 (c = 0.03, CH_2Cl_2). **HPLC**: Chiralpak IB N-5 column, 95:5 hexanes/isopropanol, 1.0 mL/min; t_R = 6.3 min (major), 9.4 min (minor), 93:7 er.

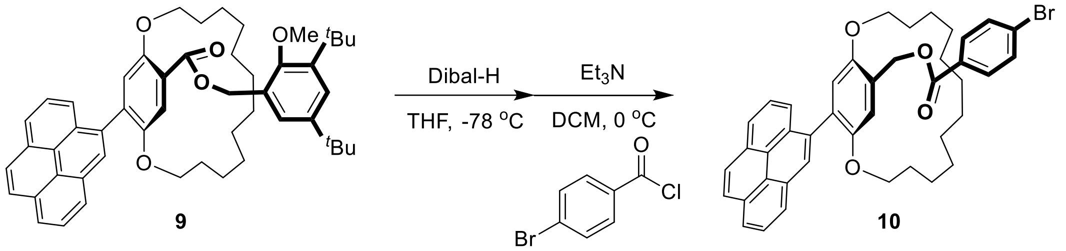

To a solution of **9** (0.5 mmol) in CH_2Cl_2 (10 mL) was added DIBAL–H (2.0 mmol, 1.0 M in toluene) slowly at – 78 °C under N_2. The reaction mixture was stirred at the same temperature for 2 h. After completion, the reaction was quenched HCl (1 M), and the solution was extracted with ethyl acetate, the combined organic phase was washed with brine, dried over Na_2SO_4, the solvent was evaporated under reduced pressure. The crude mixture was used directly for next step.

The above benzylic alcohol was dissolved in CH_2Cl_2 (10 mL). Then, the reaction mixture was cooled to 0 °C and was added Et_3N (101 mg, 1.0 mmol), 4-bromobenzoyl chloride (218 mg, 1.0 mmol) and DMAP (12.2 mg, 0.1 mmol). The reaction mixture was stirred at room temperature for 2 h. After completion, the reaction was quenched with $NaHCO_3$ (aq), and the solution was extracted with ethyl acetate, the combined organic phase was washed with brine, dried over Na_2SO_4, the solvent was evaporated under reduced pressure. The residue was purified by column chromatography using silica gel to afford the product, which was recrystallized by EtOAc to give the optically pure **10** (264 mg, 77% yield, 99% *ee*) as white solid. **^{1}H NMR** (600 MHz, $CDCl_3$) δ 8.14 – 8.26 (m, 3H), 8.11 (dd, *J* = 4.2, 1.2 Hz, 2H), 7.99 – 8.04 (m, 4H), 7.92 – 7.94 (m, 1H), 7.61 (d, *J* = 8.4 Hz, 2H), 7.31 (d, *J* = 41.4 Hz, 1H), 7.02 (d, *J* = 13.2 Hz, 1H), 5.50 – 5.59 (m, 2H), 4.35 – 4.42 (m, 1H), 4.13 – 4.19 (m, 1H), 4.00 – 4.06 (m, 1H), 3.61 – 3.66 (m, 1H), 1.94 – 2.06 (m, 1H), 1.57 – 1.65 (m, 1H), 1.33 – 1.39 (m, 3H), 1.20 – 1.30 (m, 5H), 0.97 – 1.15 (m, 10H). **^{13}C NMR** (150 MHz, $CDCl_3$) δ 166.1, 150.3, 149.9, 149.8, 149.4, 134.7, 134.0, 131.9, 131.58, 131.55, 131.48, 131.46, 131.3, 131.04, 130.9, 130.8, 129.6, 129.5, 129.51, 129.46, 128.9, 128.8, 128.22, 128.21, 127.7, 127.59, 127.56, 127.52, 127.49, 127.4, 127.3, 126.3, 126.13, 126.10, 125.5, 125.4, 125.3, 125.16, 125.15, 125.13, 125.10, 125.0, 124.9, 124.8, 124.6, 69.2, 68.8, 68.2, 67.9, 62.6, 62.5, 31.6, 30.4, 29.9, 28.81, 28.76, 28.3, 28.2, 28.1, 28.03, 27.99, 27.8, 27.7, 27.54, 27.46, 27.03, 26.95, 26.9, 24.2, 24.0, 23.9, 23.8. **HRMS** (ESI): m/z calculated for $C_{42}H_{41}BrNaO_4^+$ [M + Na]$^+$ 711.2080, found 711.2088. $[\alpha]_D^{25}$ = 24.0 (c = 0.1, CH_2Cl_2). **HPLC**: Chiralpak IB N-5 column, 95:5 hexanes/isopropanol, 1.0 mL/min; t_R = 6.1 min (major), 6.7 min (minor), >99:1 er.

1.4 Racemization experiment

1.The chiral macrocycle product **3ae** was dissolved in toluene (0.002 M) and heated to 110 °C, the *ee* value was then determined by chiral HPLC analysis every 6 h. No racemization of **3ae** was observed after 7 days.

2.

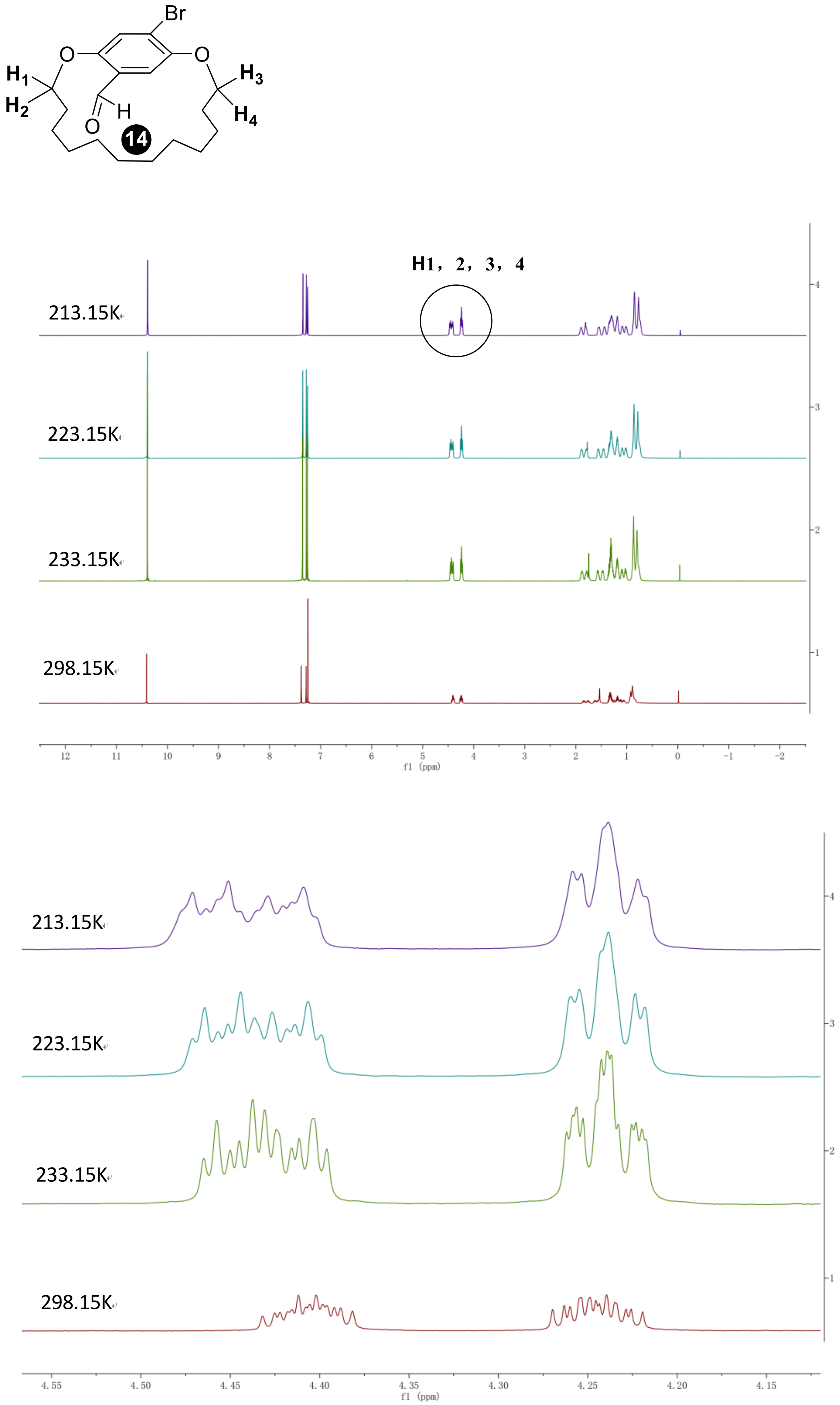

Supplementary Figure 1. The dynamic NMR of **1a**

1.5 Controlled experiments

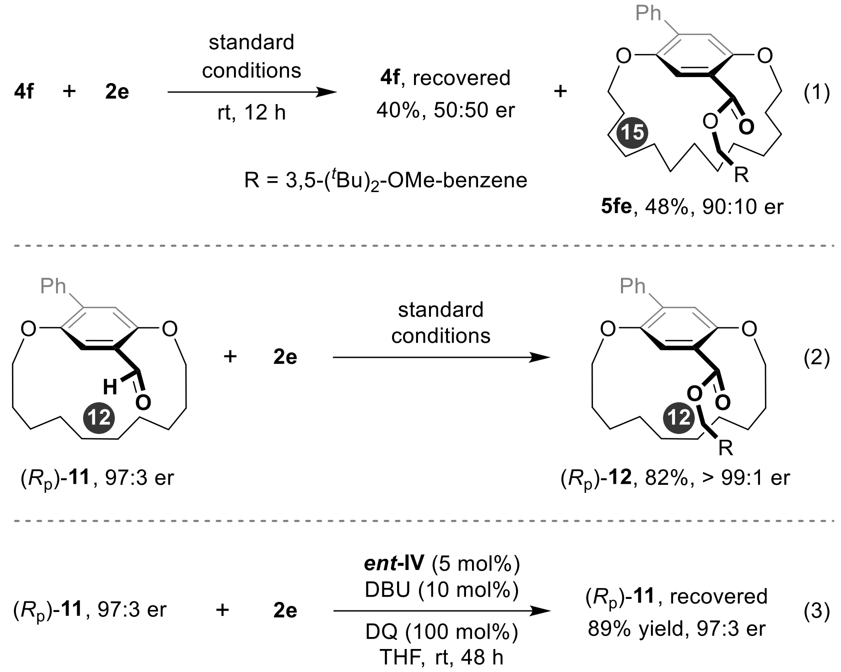

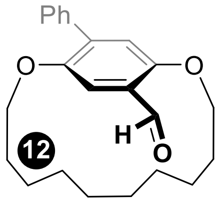

11, White solid, **^{1}H NMR** (400 MHz, $CDCl_3$) δ 10.49 (s, 1H), 7.54 – 7.56 (m, 3H), 7.37 – 7.47 (m, 3H), 7.09 (s, 1H), 4.53 – 4.58 (m, 1H), 4.20 – 4.32 (m, 2H), 3.86 – 3.93 (m, 1H), 1.86 – 1.94 (m, 1H), 1.60 – 1.69 (m, 2H), 1.20 – 1.35 (m, 3H), 1.01 – 1.16 (m, 3H), 0.65 – 0.95 (m, 7H). **^{13}C NMR** (100 MHz, $CDCl_3$) δ 189.5, 156.3, 150.2, 140.9, 137.7, 129.4, 128.4, 128.2, 126.4, 120.2, 117.4, 70.2, 69.8, 27.72, 27.70, 27.6, 27.51, 27.48, 27.3, 24.1, 24.0. **HRMS** (ESI): m/z calculated for $C_{23}H_{28}NaO_3^+$ [M + Na]$^+$ 375.1931, found 375.1939. $[\alpha]_D^{25}$ = – 64.5 (c = 0.33, CH_2Cl_2). **HPLC**: Chiralpak IB N-5 column, 95:5 hexanes/isopropanol, 1.0 mL/min; t_R = 4.9 min (major), 5.5 min (minor); 97:3 er.

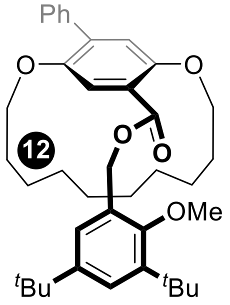

12, 48.6 mg, 82% yield, Colorless oil. **^{1}H NMR** (400 MHz, $CDCl_3$) δ 7.56 – 7.60 (m, 3H), 7.34 – 7.45 (m, 5H), 7.08 (s, 1H), 5.46 (s, 2H), 4.40 – 4.46 (m , 1H), 4.12 – 4.21 (m, 2H), 3.83 – 3.89 (m, 4H), 1.82 (s, 1H), 1.63 (s, 1H), 1.42 (s, 9H), 1.32 (s, 10H), 1.20 – 1.56 (m, 3H), 0.83 – 1.06 (m,

10H). **^{13}C NMR** (100 MHz, $CDCl_3$) δ 165.9, 156.3, 153.7, 149.6, 145.9, 142.0, 138.0, 137.7, 129.3, 128.8, 128.4, 127.9, 125.9, 124.6, 122.1, 121.6, 121.5, 70.6, 69.9, 63.1, 62.8, 35.5, 34.7, 31.7, 31.2, 28.0, 27.8, 27.7, 27.5, 27.3, 27.2, 24.0. **HRMS** (ESI): m/z calculated for $C_{39}H_{52}NaO_5^+$ [M + Na]$^+$ 623.3707, found 623.3700. $[\alpha]_D^{25}$ = 31.2 (c = 0.20, CH_2Cl_2). **HPLC**: Chiralpak IB N-5 column, 95:5 hexanes/isopropanol, 1.0 mL/min; t_R = 4.5 min (major), 5.8 min (minor);>99:1 er.

1.6 DFT Calculations

Density functional theory (DFT) study was performed using Gaussian 09 program.[6] All structural optimizations and frequency calculations were obtained using the B3LYP[7] functional with D3 dispersion correction[8] and the 6-31G(d,p)[9] basis set. Polarizable continuum model was adopted to account for the solvent effect. Single point energies was calculated by the M06-2X[10] functional with D3(0) dispersion correction upon the optimized structures, using basis set 6-311+G(d,p)[11] for all atoms. The transition states (TS) were further confirmed by performing intrinsic reaction coordinate (IRC) calculation. Atoms-in-molecules analyses (AIM)[12] and noncovalent interactions (NCI)[13] for transition states **TS-(*R*$_p$)-1a** and **TS-(*S*$_p$)-1a** were also performed with Multiwfn[14] to further display determining factors of stereoselectivity. All plots and molecular structures were showed by the VMD[15] and Cylview visualization package.[16]

The origin of stereoselectivity may be revealed by exploring the transition state of the reaction between NHC catalyst **IV** and [14]paracyclophane **1a**. The corresponding energy profile is summarized in **Supplementary Figure. 2**. For each diastereomeric pathway, we consider two orientations of the aldehyde carbonyl group, where the energetically favored one is indicated by the suffix **-1a** while the disfavored one is labeled by **-1a'**. Comparing **1a'** to the respective **1a** pathway, changing the orientation of the aldehyde carbonyl group leads to an increase of relative Gibbs free energy by 7.2 and 1.5 kcal/mol for the R_p and S_p transition state, respectively.

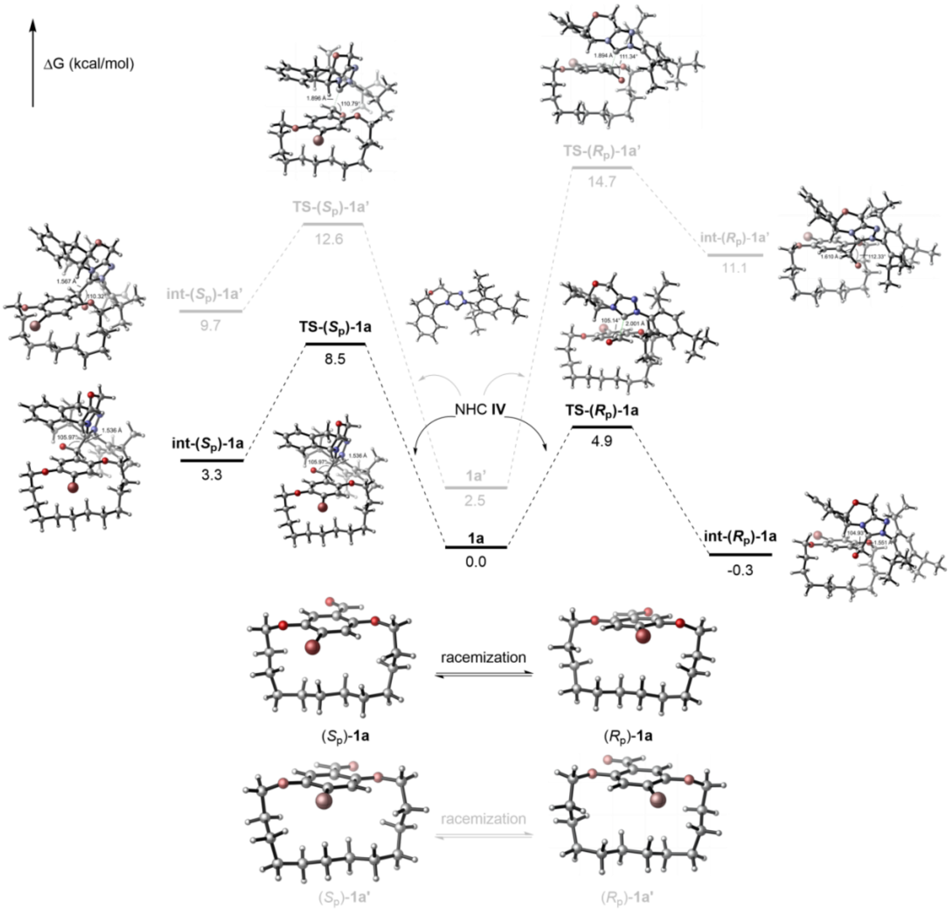

Supplementary Figure 2. Calculated energy profile of the addition of NHC catalyst **IV** to [14]paracyclophane **1a**. M06-2X-D3(0)/6-311+G(d,p)/PCM (THF)//B3LYP-D3/6-31G(d,p)/PCM (THF).

AIM analyses for transition states TS-(*R*$_p$)-1a and TS-(*S*$_p$)-1a.

The atoms-in-molecules (AIM) analyses for the key transition states **TS-(*R*$_p$)-1a** and **TS-(*S*$_p$)-1a** to further display determining factors of stereoselectivity. The value of Laplacian of electron densities ($\nabla^2\rho$) could be used to quantitatively measure the strength of the weak interactions. All the weak interactions with the values of Laplacian of electron densities were depicted in **Supplementary table 1**. Seven C–H…π interactions (2.74 Å, 2.77 Å, 2.87 Å, 3.08 Å, 3.15 Å, 3.21 Å and 3.41 Å), four C–H…O hydrogen bond interactions (2.36 Å, 2.50 Å, 2.69 Å, 2.53 Å, and 3.11 Å), two C–H…N hydrogen bond interactions (2.17 Å and 2.95 Å), one Lp…π interactions (3.27 Å), one C–H…Br halogen bond interactions (3.40 Å) and ten C–H…H–C van der Waals interaction can be found in **TS-(*R*$_p$)-1a**. For **TS-(*S*$_p$)-1a**, four C–H…π interactions (2.38 Å, 2.66 Å, 2.85 Å and 3.16 Å), three C–H…O hydrogen bond interactions (2.24 Å, 2.37 Å and 2.45 Å), one C–H…N hydrogen bond interactions (2.68 Å), two C–H…Br halogen bond interactions (3.09 Å and 3.16 Å) and night C–H…H–C van der Waals interaction can be found. Thus, the weak interactions in **TS-(*R*$_p$)-1a** are more than those observed in **TS-(*S*$_p$)-1a**, which leads to **TS-(*R*$_p$)-1a** has a lower energy barrier. AIM analyses of TS-(Rp)-1a, TS-(Sp)-1a and Laplacian of electron density (D represents distance in Å, ∇2ρ in 10-1 a.u.). The red, blue, green, pink and white balls represent oxygen, nitrogen, carbon, bromo and hydrogen atoms in three-dimensional structures, respectively. Bond critical points (BCPs) along the bond paths were shown as the small orange balls and yellow lines.

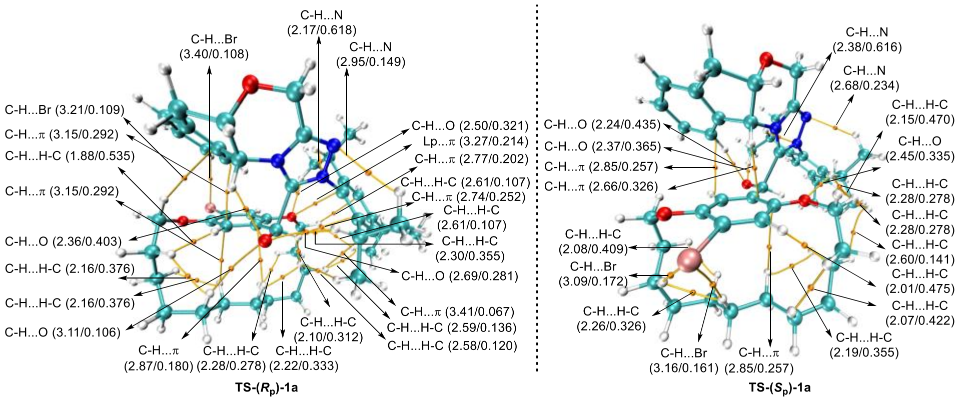

Supplementary table 1: AIM analyses of TS-(Rp)-1a, TS-(Sp)-1a

**TS-(R_p)-1a**			**TS-(S_p)-1a**		
($\nabla^2\rho$)	D (Å)	type	($\nabla^2\rho$)	D (Å)	type
0.618	2.17	C-H...N	0.616	2.38	C-H...π
0.535	1.88	C-H...H-C	0.475	2.01	C-H...H-C
0.403	2.36	C-H...O	0.470	2.15	C-H...H-C
0.386	2.12	C-H...H-C	0.435	2.24	C-H...O
0.376	2.16	C-H...H-C	0.422	2.07	CH...H-C
0.359	2.18	C-H...H-C	0.409	2.08	C-H...H-C
0.355	2.3	C-H...H-C	0.365	2.37	C-H...O
0.333	2.22	C-H...H-C	0.355	2.19	C-H...H-C
0.321	2.5	C-H...O	0.335	2.45	C-H...O
0.312	2.1	C-H...H-C	0.326	2.66	C-H...π
0.292	3.15	C-H...π	0.326	2.26	C-H...H-C
0.281	2.69	C-H...O	0.278	2.28	C-H...H-C
0.252	2.74	C-H...π	0.257	2.85	C-H...π
0.214	3.27	Lp...π	0.234	2.68	C-H...N
0.202	2.77	C-H...π	0.205	2.37	C-H...H-C
0.180	2.87	C-H...π	0.172	3.09	C-H...Br
0.149	2.95	C-H...N	0.161	3.16	C-H...Br
0.136	2.59	C-H...H-C	0.141	2.6	C-H...H-C
0.127	3.08	C-H...π	0.101	3.16	C-H...π
0.120	2.58	C-H...H-C	-	-	-
0.109	3.21	C-H...π	-	-	-
0.108	3.4	C-H...Br	-	-	-
0.107	2.61	C-H...H-C	-	-	-
0.106	3.11	C-H…O	-	-	-
0.067	3.41	C-H...π	-	-	-

NCI analyses for transition states TS-(R_p)-1a and TS-(S_p)-1a.

The NCI plots of the transition states further corroborate these noncovalent interactions using the Multiwfn programs. Some (not all) typical weak interactions were marked with arrows, which were consistent with the AIM analysis. Therefore, we can speculate that the weak interactions

largely stabilize the transition states. It is worth noting that the red dashed box contains a magnified image viewed from another angle.

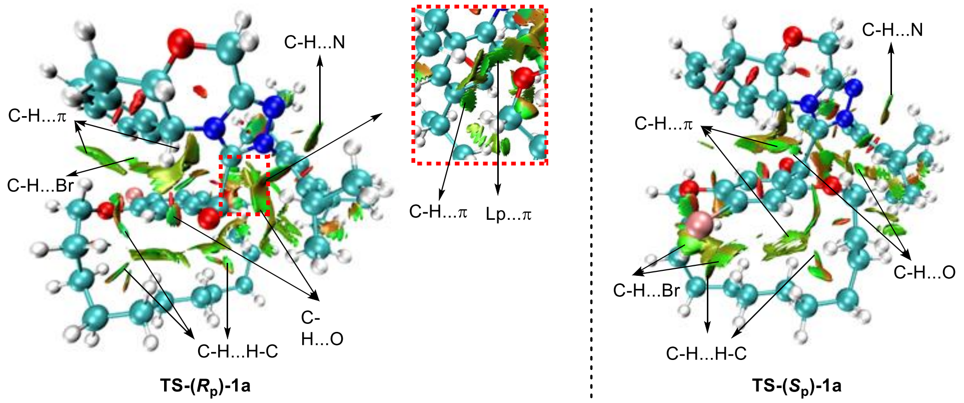

Supplementary Figure 3. NCI plots for **TS-(R_p)-1a** (left) and **TS-(S_p)-1a** (right) structures generated from wavefunction at B3LYP-D3/6-31G (d,p) level of theory with Multiwfn and plotted with VMD with density cutoff at 0.05 a.u. Colors reflect sign (λ_2)ρ on a scale of -0.04 au (blue) over 0 (green) to +0.02 (red). Accordingly, blue: strong attractive; green: weak attractive; and red: strong repulsive. Colour code: hydrogen (white), carbon (turquoise), nitrogen (blue), oxygen (red) bromo (pink).

2. Supplementary Note

2.1 Characterization data

2.1.1 Characterization data of unknown macrocycles substrates

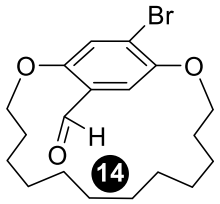

1a, White solid, yield: 30%. **^{1}H NMR** (400 MHz, $CDCl_3$) δ 10.41 (s, 1H), 7.38 (s, 1H), 7.28 (s, 1H), 4.38 – 4.42 (m, 2H), 4.21 – 4.26 (m, 2H), 1.72 – 1.87 (m, 2H), 1.51 – 1.66 (m, 2H), 1.26 – 1.36 (m, 4H), 1.03 – 1.22 (m, 4H), 0.88 – 0.92 (m, 8H). **^{13}C NMR** (100 MHz, $CDCl_3$) δ 189.1, 155.1, 149.0, 125.4, 121.8, 120.7, 113.0, 69.3, 69.2, 28.6, 28.10, 28.05, 27.60, 27.56, 27.0, 26.6, 24.1, 24.0. **HRMS** (ESI): m/z calculated for $C_{19}H_{27}BrNaO_3^+$ $[M + Na]^+$ 405.1036, found 405.1032.

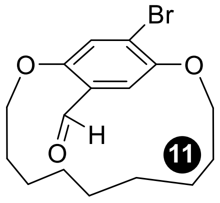

1b, White solid, yield: 10%. **^{1}H NMR** (600 MHz, $CDCl_3$) δ 10.40 (s, 1H), 7.42 (s, 1H), 7.32 (s, 1H), 4.42 – 4.46 (m, 2H), 4.23 – 4.28 (m, 2H), 1.73 – 1.80 (m, 1H), 1.53 – 1.69 (m, 3H), 1.19 – 1.28 (m,

2H), 1.04 – 1.12 (m, 4H), 0.85 – 0.98 (m, 2H), 0.58 – 0.75 (m, 4H).**^{13}C NMR** (150 MHz, $CDCl_3$) δ 189.1, 156.0, 149.8, 126.8, 123.3, 123.0, 115.8, 70.6, 70.3, 27.78, 27.75, 27.7, 27.49, 27.45, 27.1, 24.0, 23.6. **HRMS** (ESI): m/z calculated for $C_{16}H_{21}BrNaO_3^+$ $[M + Na]^+$ 363.0566, found 363.0560.

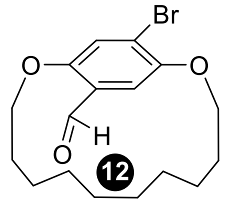

1c, White solid, yield: 34%. **^{1}H NMR** (400 MHz, $CDCl_3$) δ 10.41 (s, 1H), 7.43 (s, 1H), 7.33 (s, 1H), 4.42 – 4.47 (m, 2H), 4.24 – 4.29 (m, 2H), 1.59 – 1.67 (m, 4H), 1.22 – 1.26 (m, 2H), 1.05 – 1.12 (m, 4H), 0.89 – 0.94 (m, 2H), 0.63 – 0.73 (m, 4H). **^{13}C NMR** (100 MHz, $CDCl_3$) δ 189.1, 156.0, 149.8, 126.8, 123.3, 123.0, 115.8, 70.6, 70.3, 27.80, 27.76, 27.7, 27.5, 27.2, 24.0, 23.7. **HRMS** (ESI): m/z calculated for $C_{17}H_{23}BrNaO_3^+$ $[M + Na]^+$ 377.0723, found 377.0729.

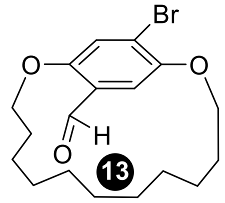

1d, White solid, yield: 33%. **1H NMR** (600 MHz, $CDCl_3$) δ 10.43 (s, 1H), 7.43 (s, 1H), 7.33 (s, 1H), 4.39 – 4.43 (m, 2H), 4.29 – 4.33 (m, 2H), 1.60 – 1.76 (m, 4H), 1.10 – 1.29 (m, 6H), 0.83 – 1.06 (m, 6H), 0.68 – 0.74 (m, 2H). **^{13}C NMR** (150 MHz, $CDCl_3$) δ 189.1, 156.3, 150.3, 126.2, 122.5, 122.1, 114.6, 70.3, 70.1, 28.71, 28.67, 28.6, 28.4, 27.3, 27.2, 24.4, 24.0. **HRMS** (ESI): m/z calculated for $C_{18}H_{25}BrNaO_3^+$ $[M + Na]^+$ 391.0879, found 391.0876.

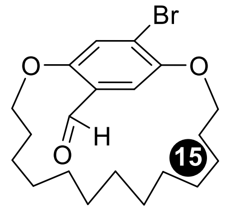

1e, White solid, yield: 30%. **^{1}H NMR** (600 MHz, $CDCl_3$) δ 10.40 (s, 1H), 7.36 (s, 1H), 7.27 (s, 1H), 4.30 – 4.34 (m, 2H), 4.18 – 4.23 (m, 2H), 1.60 –1.75 (m, 4H), 1.21 – 1.41(m, 6H), 0.12 – 0.85 (m, 12H). **^{13}C NMR** (150 MHz, $CDCl_3$) δ 189.0, 155.3, 149.4, 125.4, 121.8, 120.2, 112.5, 69.2, 68.9, 29.5, 28.40, 28.35, 28.21, 28.16, 27.71, 27.66, 27.5, 27.4, 24.1, 24.0 **HRMS** (ESI): m/z calculated for $C_{20}H_{29}BrNaO_3^+$ $[M + Na]^+$ 419.1192, found 419.1196.

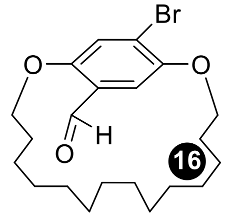

1f, Yellow solid, yield: 33%. **^{1}H NMR** (600 MHz, $CDCl_3$) δ 10.43 (s, 1H), 7.36 (s, 1H), 7.27 (s, 1H), 4.20 – 4.30 (m, 4H), 1.78 –1.83 (m, 4H), 0.93 – 1.51 (m, 20H). **^{13}C NMR** (150 MHz, $CDCl_3$) δ 189.0, 155.3, 149.3, 124.9, 121.4, 119.5, 111.8, 68.8, 68.5, 29.10, 29.09, 28.4, 28.3, 28.1, 28.0, 27.5, 27.2, 26.9, 24.0, 23.8. **HRMS** (ESI): m/z calculated for $C_{21}H_{31}BrNaO_3^+$ $[M + Na]^+$ 433.1349, found 433.1355.

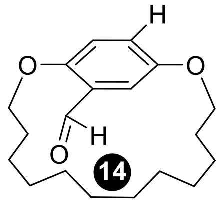

1g, Colorless liquid, yield: 15%. **^{1}H NMR** (600 MHz, $CDCl_3$) δ 10.46 (s, 1H), 7.36 (s, 1H), 7.14 (s, 1H), 6.98 (s, 1H), 4.21 – 4.31 (m, 4H), 1.63 – 1.70 (m, 4H), 0.87 – 1.31 (m, 16H). **^{13}C NMR** (150 MHz, $CDCl_3$) δ 189.9, 155.7, 152.3, 126.4, 125.6, 116.9, 113.6, 69.0, 68.3, 28.6, 28.5, 27.99, 27.97, 27.60, 27.55, 27.2, 26.9, 24.2, 24.0. **HRMS** (ESI): m/z calculated for $C_{19}H_{28}NaO_3^+$ $[M + Na]^+$ 327.1931, found 327.1934.

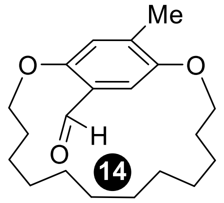

1h, White solid, yield: 11%. **^{1}H NMR** (600 MHz, $CDCl_3$) δ 10.42 (s, 1H), 7.30 (s, 1H), 6.86 (s, 1H), 4.38 – 4.44 (m, 2H), 4.12 – 4.22 (m, 2H), 2.28 (s, 3H), 1.78 – 1.83 (m, 2H), 1.49 – 1.62 (m, 2H), 1.27 – 1.33 (m, 4H), 1.04 – 1.19 (m, 4H), 0.87 – 0.91 (m, 6H), 0.82 – 0.85 (m, 2H). **^{13}C NMR** (150 MHz, $CDCl_3$) δ 189.7, 155.4, 150.5, 137.5, 124.4, 118.2, 110.9, 68.9, 68.0, 28.7, 28.6, 28.10, 28.09, 27.7, 27.6, 27.4, 26.7, 24.3, 24.1, 17.6. **HRMS** (ESI): m/z calculated for $C_{20}H_{30}BrNaO_3^+$ $[M + Na]^+$ 341.2087, found 341.2084.

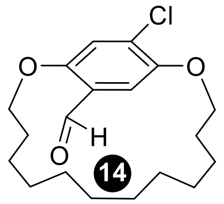

1i, White solid, yield: 15%. **^{1}H NMR** (400 MHz, $CDCl_3$) δ 10.41 (s, 1H), 7.43 (s, 1H), 7.11 (s, 1H), 4.38 – 4.44 (m, 2H), 4.22 – 4.29 (m, 2H), 1.53 – 1.86 (m, 4H), 0.86 – 1.38 (m, 16H). **^{13}C NMR** (100 MHz, $CDCl_3$) δ 189.0, 155.2, 148.1, 132.0, 124.9, 117.6, 113.8, 69.4, 69.1, 28.65, 28.62, 28.1, 27.6, 27.0, 26.6, 24.1, 23.9. **HRMS** (ESI): m/z calculated for $C_{19}H_{27}ClNaO_3^+$ $[M + Na]^+$ 361.1541, found 361.1547.

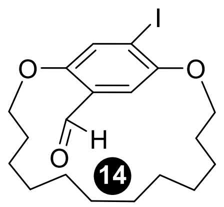

1j, Yellow solid, yield: 10%. **^{1}H NM**R (600 MHz, $CDCl_3$) δ 10.43 (s, 1H), 7.54 (s, 1H), 7.27 (s, 1H), 4.40 – 4.43 (m, 2H), 4.22 – 4.27 (m, 2H), 1.72 – 1.89 (m, 4H), 1.55 – 1.68 (m, 4H), 1.04 – 1.22 (m, 4H), 0.90 – 0.94 (m, 8H). **^{13}C NMR** (150 MHz, $CDCl_3$) δ 189.4, 161.3, 155.0, 127.0, 111.1, 97.7, 69.4, 69.3, 64.2, 29.6, 28.6, 28.2, 27.6, 27.5, 27.1, 27.0, 26.6, 26.0, 24.1. **HRMS** (ESI): m/z calculated for $C_{19}H_{27}INaO_3^+$ $[M + Na]^+$ 453.0897, found 453.0892.

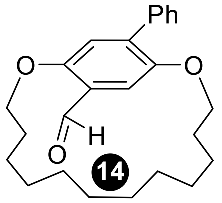

1k, Colorless liquid, yield: 12%. **^{1}H NMR** (600 MHz, $CDCl_3$) δ 10.50 (s, 1H), 7.53 – 7.55 (m, 2H), 7.48 (s, 1H), 7.44 (t, J = 7.8 Hz, 2H), 7.37 – 7.39 (m, 1H), 7.02 (s, 1H), 4.46 – 4.49 (m, 1H), 4.29 – 4.32 (m, 1H), 4.23 – 4.27 (m, 1H), 3.99 – 4.03 (m, 1H), 1.88 – 1.95 (m, 1H), 1.61 – 1.76 (m, 2H), 1.36 – 1.44 (m, 3H), 1.22 – 1.30 (m, 1H), 1.13 – 1.20 (m, 2H), 1.05 – 1.11 (m, 2H), 0.96 – 1.03 (m, 3H), 0.90 – 0.92 (m, 6H). **^{13}C NMR** (150 MHz, $CDCl_3$) δ 189.4, 155.3, 155.29, 149.3, 139.9, 137.8, 129.4, 128.2, 127.9, 118.0, 113.62, 113.57, 68.8, 68.6, 28.7, 28.2, 28.0, 27.7, 27.5, 27.21, 27.18, 26.79, 26.76, 24.24, 24.21, 23.9, 23.8. **HRMS** (ESI): m/z calculated for $C_{25}H_{32}NaO_3^+$ $[M + Na]^+$ 403.2244, found 403.2249.

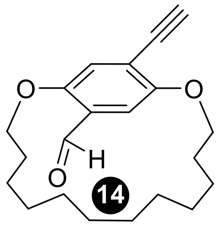

1l, Yellow solid, 57%. **^{1}H NMR** (600 MHz, $CDCl_3$) δ 10.44 (s, 1H), 7.38 (s, 1H), 7.14 (s, 1H), 4.39 – 4.44 (m, 2H), 4.21 – 4.28 (m, 2H), 3.47 (s, 1H) , 1.75 – 1.87 (m, 2H), 1.53 – 1.64 (m, 2H), 1.28 – 1.43 (m, 4H), 1.16 – 1.21 (m, 2H), 1.04 – 1.13 (m, 2H), 0.87 – 0.95 (m, 8H). **^{13}C NMR** (150 MHz, $CDCl_3$) δ 189.3, 154.6, 153.4, 126.5, 120.7, 120.2, 112.9, 84.7, 79.7, 69.1, 69.0, 28.7, 28.6, 28.1, 27.7, 27.6, 27.1, 26.8, 24.2, 24.0. **HRMS** (ESI): m/z calculated for $C_{21}H_{28}NaO_3^+$ $[M + Na]^+$ 351.1931, found 351.1937.

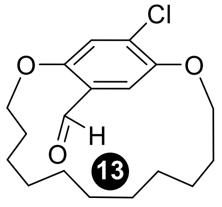

4a, White solid, yield: 23%. **^{1}H NMR** (600 MHz, $CDCl_3$) δ 10.42 (s, 1H), 7.47 (s, 1H), 7.14 (s, 1H), 4.38 – 4.42 (m, 2H), 4.28 – 4.33 (m, 2H), 1.61 – 1.73 (m, 4H), 1.13 – 1.26 (m, 6H), 0.94 – 1.06 (m, 3H), 0.83 – 0.89 (m, 3H), 0.68 – 0.72 (m, 2H). **^{13}C NMR** (150 MHz, $CDCl_3$) δ 188.9, 156.4, 149.3, 132.6, 125.6, 118.9, 115.2, 70.3, 70.0, 28.7, 28.5, 28.4, 27.27, 27.25, 27.1, 24.3, 24.0. **HRMS** (ESI): m/z calculated for $C_{18}H_{26}ClO_3^+$ $[M + H]^+$ 325.1565, found 325.1560.

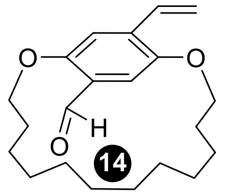

4b, Green oil, yield: 91%. **^{1}H NMR** (600 MHz, $CDCl_3$) δ 10.41 (s, 1H), 7.43 (s, 1H), 7.11 (s, 1H), 7.05 (dd, *J* = 17.8, 11.1 Hz, 1H), 5.80 (d, *J* = 18 Hz, 1H), 5.39 (d, *J* = 12.0 Hz, 1H), 4.36 – 4.46 (m, 2H), 4.11 – 4.22 (m, 2H), 1.71 – 1.85 (m, 2H), 1.46 – 1.61 (m, 2H), 1.22 – 1.31 (m, 4H), 1.00 –1.18 (m, 4H), 0.80 – 0.89 (m, 8H). **^{13}C NMR** (150 MHz, $CDCl_3$) δ 189.3, 155.3, 149.5, 135.5, 131.5, 125.6, 121.7, 117.6, 113.1, 112.6, 68.8, 68.4, 28.5, 28.0, 27.9, 27.6, 27.5, 27.1, 26.7, 24.1, 24.0. **HRMS** (ESI): m/z calculated for $C_{21}H_{30}NaO_3^+$ $[M + Na]^+$ 353.2087, found 353.2082.

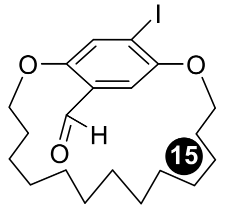

4c, Yellow solid, yield: 20%. **1H NMR** (600 MHz, $CDCl_3$) δ 10.43 (s, 1H), 7.53 (s, 1H), 7.25 (s, 1H), 4.31 – 4.36 (m, 2H), 4.20 – 4.24 (m, 2H), 1.63 – 1.78 (m, 4H), 1.40 – 1.46 (m, 1H), 1.32 – 1.38 (m, 3H), 1.24 – 1.31 (m, 3H), 1.12 – 1.17 (m, 1H), 1.05 – 1.11 (m, 2H), 0.96 – 1.02 (m, 4H), 0.87 – 0.94 (m, 4H). **^{13}C NMR** (150 MHz, $CDCl_3$) δ 189.4, 155.3, 151.7, 126.5, 126.3, 110.5, 97.7, 69.2, 68.9, 29.6, 28.5, 28.4, 28.3, 28.2, 27.7, 27.43, 27.41, 27.3, 24.2. **HRMS** (ESI): m/z calculated for $C_{20}H_{29}INaO_3^+$ $[M + Na]^+$ 467.1054, found 467.1050.

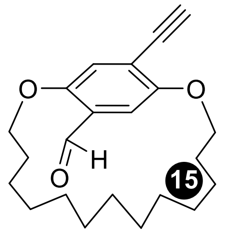

4d, Yellow oil, yield: 55%. **^{1}H NMR** (600 MHz, $CDCl_3$) δ 10.45 (s, 1H), 7.48 (s, 1H), 7.14 (s, 1H), 4.22 – 4.38 (m, 4H), 3.47 (m, 1H), 1.64 – 1.77 (m, 4H), 1.34 – 1.48 (m, 4H), 1.25 – 1.32 (m, 4H), 0.91 – 1.05 (m, 10H). **^{13}C NMR** (150 MHz, $CDCl_3$) δ 189.3, 154.8, 153.7, 126.5, 120.2, 112.3, 84.7, 79.7, 68.9, 68.7, 29.8, 29.6, 28.4, 28.24, 28.21, 27.59, 27.54, 27.51, 27.46, 24.2, 24.0. **HRMS** (ESI): m/z calculated for $C_{22}H_{30}NaO_3^+$ $[M + Na]^+$ 365.2087, found 365.2080.

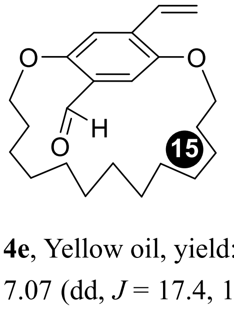

4e, Yellow oil, yield: 90%. **^{1}H NMR** (600 MHz, $CDCl_3$) δ 10.43 (s, 1H), 7.33 (s, 1H), 7.12 (s, 1H), 7.07 (dd, *J* = 17.4, 10.8 Hz, 1H), 5.81 (dd, *J* = 18.0, 1.2 Hz, 1H), 5.40 (dd, *J* = 10.8, 1.2 Hz, 1H),

4.38 (ddd, *J* = 11.4, 6.6, 4.2 Hz, 1H), 4.31 (ddd, *J* = 11.4, 7.2, 4.2 Hz, 1H), 4.20 (ddd, *J* = 11.4, 7.2, 4.2 Hz,1H), 4.13 (ddd, *J* = 11.4, 7.2, 4.2 Hz, 1H), 1.57 – 1.74 (m, 4H), 0.81 – 1.36 (m, 18H). ^{13}**C NMR** (150 MHz, $CDCl_3$) δ 189.3, 155.6, 149.9, 135.4, 131.5, 125.6, 117.5, 112.6, 112.0, 68.5, 68.2, 29.4, 28.4, 28.3, 28.1, 27.8, 27.5, 27.40, 27.36, 24.2, 24.0. **HRMS** (ESI): m/z calculated for $C_{22}H_{32}NaO_3^+$ $[M + Na]^+$ 367.2244, found 367.2250.

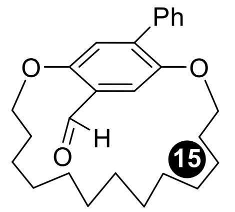

4f, Colorless solid, yield: 88%. ^{1}**H NMR** (600 MHz, $CDCl_3$) δ 10.51 (s, 1H), 7.37 – 7.54 (m, 6H), 7.02 (s, 1H), 4.41 – 4.43 (m, 1H), 4.24 – 4.27 (m, 2H), 4.01 – 4.05 (m, 1H), 0.94 – 1.81 (m, 22H) ^{13}**C NMR** (150 MHz, $CDCl_3$) δ 189.5, 155.4, 149.2, 134.0, 137.9, 129.3, 128.1, 128.0, 118.1, 113.5, 68.8, 68.7, 28.3, 28.2, 28.0, 27.8, 27.5, 27.2, 26.9, 24.1, 23.9, 23.8, 23.3. **HRMS** (ESI): m/z calculated for $C_{26}H_{34}NaO_3^+$ $[M + Na]^+$ 417.2400, found 417.2409.

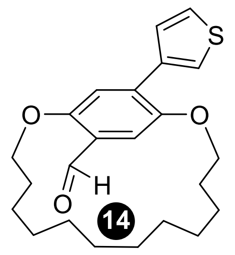

4g, Yellow solid, yield: 85%. ^{1}**H NMR** (600 MHz, $CDCl_3$) δ 10.47 (s, 1H), 7.77 (dd, *J* = 3.0, 1.2 Hz, 1H), 7.46 –7.47 (m, 2H), 7.37 (dd, *J* = 4.8, 3.0 Hz, 1H), 7.19 (s, 1H), 4.49 (ddd, *J* = 10.2, 6.0, 4.2 Hz, 1H), 4.41 (ddd, *J* = 10.2, 6.0, 4.2 Hz, 1H), 4.26 (ddd, *J* = 12.6, 9.0, 4.2 Hz, 1H), 4.15 (ddd, *J* = 12.0, 8.4, 3.6 Hz, 1H), 1.85 – 1.92 (m, 1H), 1.75 – 1.82 (m, 1H), 1.62 – 1.68 (m, 1H), 1.47 – 1.53 (m, 1H), 1.33 – 1.39 (m, 2H), 1.17 – 1.24 (m, 2H), 1.11 – 1.62 (m, 2H), 1.01 – 1.09 (m, 2H), 0.81 – 0.97 (m, 8H). ^{13}**C NMR** (150 MHz, $CDCl_3$) δ 189.4, 155.4, 149.3, 137.6,133.8, 128.4, 125.5, 125.0, 116.7, 113.3, 69.0, 68.6, 28.6, 28.2, 28.0, 27.7 27.5, 27.3, 26.8, 24.3, 24.0. **HRMS** (ESI): m/z calculated for $C_{23}H_{31}O_3S^+$ $[M + H]^+$ 387.1988, found 387.1984.

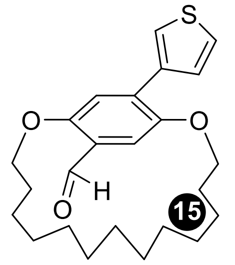

4h, Yellow solid, yield: 81%. ^{1}**H NMR** (600 MHz, $CDCl_3$) δ 10.48 (s, 1H), 7.77 (dd, *J* = 3.0, 1.8 Hz, 1H), 7.46 – 7.47 (m, 2H), 7.37 (dd, *J* = 4.8, 3.0 Hz, 1H), 7.19 (s, 1H), 4.42 (ddd, *J* = 11.4, 6.6, 3.6 Hz, 1H), 4.34 (ddd, *J* = 11.4, 6.6, 3.6 Hz, 1H), 4.24 (ddd, *J* = 11.4, 7.2, 3.6 Hz,1H), 4.16 (ddd,

J = 11.4, 7.8, 4.2 Hz, 1H), 1.75 – 1.82 (m, 1H), 1.67 – 1.72 (m, 2H), 1.54 – 1.61 (m, 1H) , 1.36 – 1.42 (m, 2H), 1.25 – 1.31 (m, 2H), 1.14 – 1.22 (m, 3H), 1.02 – 1.09 (m, 2H), 0.95 – 1.01 (m, 4H), 0.84 – 0.93 (m, 5H). **^{13}C NMR** (150 MHz, $CDCl_3$) δ 189.3, 155.6, 149.6, 137.5, 133.7, 128.4, 125.5, 125.0, 116.2, 112.5, 68.7, 68.4, 29.5, 28.4, 28.3, 28.2, 28.1, 27.9, 27.5, 27.44, 27.36, 24.3, 24.0. **HRMS** (ESI): m/z calculated for $C_{24}H_{32}NaO_3S^+$ $[M + Na]^+$ 423.1964, found 423.1960.

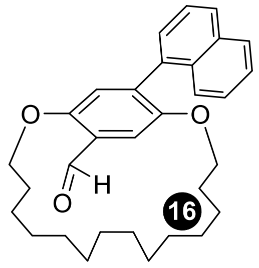

4i, Green oil, yield: 87%. **^{1}H NMR** (600 MHz, $CDCl_3$) δ 10.53 (s, 1H), 7.98 (s, 1H), 7.88 – 7.90 (m, 3H), 7.68 (dd, *J* = 9.0, 1.8 Hz, 1H), 7.52 (dd, *J* = 6.6, 3.6 Hz, 2H), 7.48 (s, 1H), 7.10 (s, 1H), 4.40 (ddd, *J* = 15.0, 7.2, 4.2 Hz, 1H), 4.20 – 4.27 (m, 2H), 4.05 (ddd, *J* = 10.8, 6, 4.8 Hz, 1H), 1.73 – 1.89 (m, 2H), 1.60 – 1.63 (m, 2H), 1.49 – 1.54 (m, 1H) , 1.37 – 1.43 (m, 2H), 0.84 – 1.36 (m, 17H). **^{13}C NMR** (150 MHz, $CDCl_3$) δ 189.5, 155.6, 149.8, 139.4, 135.6, 133.3, 132.9, 128.4, 128.3, 127.8, 127.6, 127.5, 126.4, 126.3, 117.2, 111.9, 68.3, 67.9, 29.3, 29.0, 28.5, 28.4, 28.2, 28.1, 27.6, 27.4, 27.3, 26.8, 24.2, 23.8. **HRMS** (ESI): m/z calculated for $C_{31}H_{38}NaO_3^+$ $[M + Na]^+$ 481.2713, found 481.2719.

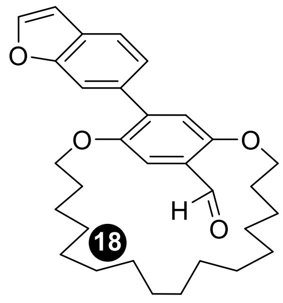

4l, Colorless oil, yield: 87%. **^{1}H NMR** (600 MHz, $CDCl_3$) δ 10.53 (s, 1H), 7.77 (d, *J* = 2.0 Hz, 1H), 7.65 (d, *J* = 2.0 Hz, 1H), 7.55 (d, *J* = 8.5 Hz, 1H), 7.49 (dd, *J* = 8.6, 1.8 Hz, 1H), 7.45 (s, 1H), 7.02 (s, 1H), 6.80 (d, *J* = 2.1 Hz, 1H), 4.22 (t, *J* = 5.7 Hz, 2H), 4.06 (t, *J* = 5.9 Hz, 2H), 1.80 (t, *J* = 6.4 Hz, 2H), 1.64 (t, *J* = 6.3 Hz, 2H), 1.46 – 1.51 (m, 2H), 1.29 (dt, *J* = 21.2, 6.9 Hz, 4H), 1.15 – 1.24 (m, 10H), 1.05 – 1.15 (m, 8H). **^{13}C NMR** (150 MHz, $CDCl_3$) δ 189.3, 155.7, 154.7, 149.8, 149.75, 145.6, 139.6, 132.6, 127.4, 126.1, 124.4, 122.2, 116.6, 111.2, 110.9, 106.8, 68.1, 67.8, 29.5, 29.4, 28.9, 28.7, 28.4, 28.20, 28.15, 28.0, 27.9, 27.8, 27.5, 27.1, 26.7, 24.4, 24.0. **HRMS** (ESI): m/z calculated for $C_{31}H_{40}O_4^+$ $[M + Na]^+$ 499.2819, found 499.2824.

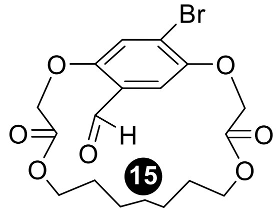

4m, White solid, yield: 22%. **^{1}H NMR** (600 MHz, $CDCl_3$) δ 10.43 (s, 1H), 7.27 (s, 1H), 7.21 (s, 1H), 4.77 – 4.84 (m, 4H), 4.23 – 4.36 (m, 1H), 4.23 – 4.27 (m, 1H), 3.97 – 4.01 (m, 2H), 1.37 – 1.51 (m, 4H), 0.92 – 1.01 (m, 4H), 0.78 – 0.89 (m, 2H). **^{13}C NMR** (150 MHz, $CDCl_3$) δ 188.3, 168.5, 168.4, 154.72, 154.70, 149.7, 125.1, 120.3, 119.0, 110.0, 66.5, 66.1, 65.5, 65.1, 28.1, 28.0, 27.7, 26.0, 25.8. **HRMS** (ESI): m/z calculated for $C_{18}H_{21}BrNaO_7^+$ $[M + Na]^+$ 451.0323, found 451.0331.

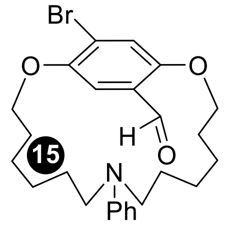

4n, White solid, yield: 20%. **^{1}H NMR** (600 MHz, $CDCl_3$) δ 10.44 (s, 1H), 7.42 (s, 1H), 7.33 (s, 1H), 7.15 (dd, *J* = 8.4, 7.2 Hz, 2H), 6.60 (t, *J* = 7.2 Hz, 1H), 6.51 (d, *J* = 7.8 Hz, 2H), 4.41 – 4.46 (m, 2H), 4.24 – 4.30 (m, 2H), 3.04 – 3.11 (m, 2H), 2.76 – 2.84 (m, 2H), 1.79 – 1.86 (m, 1H), 1.62 – 1.78 (m, 3H), 1.35 – 1.43 (m, 5H), 1.24 – 1.33 (m, 7H). **^{13}C NMR** (150 MHz, $CDCl_3$) δ 189.0, 155.2, 149.34, 149.32, 147.8, 129.3, 125.5, 121.8, 120.4, 115.4, 112.7, 111.9, 69.6, 69.2, 51.1, 51.0, 27.7, 27.4, 27.1, 26.6, 26.4, 25.7, 25.2, 25.1. **HRMS** (ESI): m/z calculated for $C_{25}H_{33}BrNO_3^+$ $[M + H]^+$ 474.1638, found 474.1643.

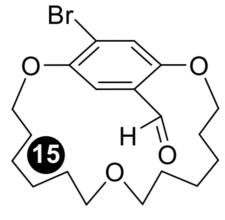

4o, White solid, yield: 30%. **^{1}H NMR** (600 MHz, $CDCl_3$) δ 10.41 (s, 1H), 7.38 (s, 1H), 7.28 (s, 1H), 4.23 – 4.34 (m, 4H), 3.04 – 3.13 (m, 4H), 1.65 – 1.77 (m, 4H), 1.31 – 1.44 (m, 6H), 1.05 – 1.28 (m, 6H). **^{13}C NMR** (150 MHz, $CDCl_3$) δ 188.9, 155.7, 149.5, 125.5, 122.0, 120.6, 113.1, 70.2, 69.9, 69.3, 69.1, 29.1, 27.8, 27.4, 24.4, 24.13, 24.06, 24.0. **HRMS** (ESI): m/z calculated for $C_{19}H_{28}BrO_4^+$ $[M + Na]^+$ 399.1165, found 399.1160.

2.1.2 Characterization data of [n]paracyclophanes

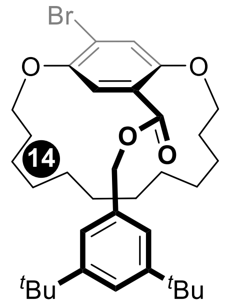

3aa, 46.8 mg, 76% yield, Colorless oil. **^{1}H NMR** (400 MHz, $CDCl_3$) δ 7.44 (s, 1H), 7.40 (t, *J* = 1.8 Hz, 1H), 7.29 (d, *J* = 1.8 Hz, 2H), 7.25 (s, 1H), 5.30 – 5.39 (m, 2H), 4.21 – 4.37 (m, 3H), 4.13 – 4.19 (m, 1H), 1.66 – 1.80 (m, 2H), 1.49 – 1.57 (m, 1H), 1.33 (s, 18H), 1.15 – 1.27 (m, 5H), 1.02 –

1.13 (m, 3H), 0.90 – 0.93 (m, 9H). **^{13}C NMR** (100 MHz, $CDCl_3$) δ 165.6, 152.7, 151.1, 148.5, 122.6, 122.4, 121.9, 121.2, 118.4, 118.0, 69.9, 69.6, 67.7, 35.0, 31.60, 31.59, 28.6, 28.13, 28.06, 27.52, 27.46, 27.4, 27.0, 24.0, 23.9. **HRMS** (ESI): m/z calculated for $C_{34}H_{49}BrNaO_4^+$ $[M + Na]^+$ 623.2706, found 623.2714. $[\alpha]_D^{25}$ = 18.9 (c = 0.45, CH_2Cl_2). HPLC: Chiralpak IB N-5 column, 95:5 hexanes/isopropanol, 1.0 mL/min; t_R = 4.6 min (major), 5.9 min (minor); 93:7 er.

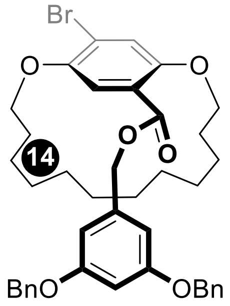

3ab, 48.8 mg, 72% yield, Colorless oil. **^{1}H NMR** (600 MHz, $CDCl_3$) δ 7.32 – 7.44 (m, 12H), 6.71 – 6.72 (m, 2H), 6.58 – 6.59 (m, 1H), 5.26 – 5.36 (m, 2H), 5.03 (d, *J* = 1.8 Hz, 4H), 4.16 – 4.38 (m, 4H), 1.70 – 1.80 (m, 2H), 1.58 – 1.62 (m, 1H), 1.23 – 1.44 (m, 5H), 1.03 – 1.21 (m, 4H), 0.92 – 0.93 (m, 8H). **^{13}C NMR** (150 MHz, $CDCl_3$) δ 165.5, 160.2, 152.6, 148.5, 138.5, 136.9, 128.7, 128.1, 127.6, 121.6, 120.8, 118.6, 118.2, 107.2, 101.7, 70.2, 69.7, 69.6, 66.7, 28.6, 28.1, 28.0, 27.5, 27.47, 27.2, 26.9, 24.0, 23.9. **HRMS** (ESI): m/z calculated for $C_{40}H_{46}BrO_6^+$ $[M + H]^+$ 701.2472, found 701.2468. $[\alpha]_D^{25}$ = 15.0 (c = 1.0, CH_2Cl_2). **HPLC**: Chiralpak IB N-5 column, 95:5 hexanes/isopropanol, 1.0 mL/min; t_R = 26.3 min (major), 39.8 min (minor), 93:7 er.

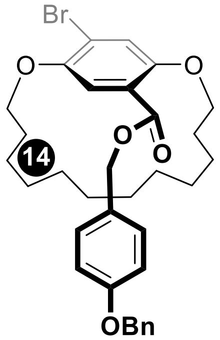

3ac, 40.5 mg, 68% yield, Colorless oil. **^{1}H NMR** (600 MHz, $CDCl_3$) δ 7.43 (d, *J* = 7.2 Hz, 2H), 7.37 – 7.40 (m, 5H), 7.31 – 7.34 (m, 1H), 7.23 (s, 1H), 6.96 (d, *J* = 9.0 Hz, 2H), 5.24 – 5.34 (m, 2H), 5.07 (s, 2H), 4.11 – 4.35 (m, 4H), 1.66 – 1.78 (m, 2H), 1.48 – 1.55 (m, 1H), 1.15 – 1.39 (m, 6H), 1.00 – 1.11 (m, 3H), 0.88 – 0.93 (m, 8H). **^{13}C NMR** (150 MHz, $CDCl_3$) δ 165.7, 158.9, 152.5, 148.5, 137.0, 130.2, 128.7, 128.1, 127.6, 121.6, 118.5, 118.2, 115.0, 70.2, 69.8, 69.6, 66.7, 28.56, 28.55, 28.1, 28.0, 27.52, 27.46, 27.2, 27.0, 24.0, 23.9. **HRMS** (ESI): m/z calculated for $C_{33}H_{40}BrO_5^+$ $[M + H]^+$ 595.2054, found 595.2060. $[\alpha]_D^{25}$ = 14.8 (c = 0.45, CH_2Cl_2). **HPLC**: Chiralpak IB N-5 column, 95:5 hexanes/isopropanol, 1.0 mL/min; t_R = 16.5 min (major), 26.3 min (minor), 88:12 er.

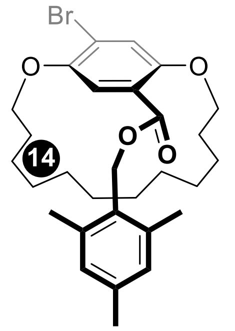

3ad, 33.7 mg, 61% yield, Colorless solid. **^{1}H NMR** (600 MHz, $CDCl_3$) δ 7.36 (s, 1H), 7.20 (s, 1H), 6.88 (s, 2H), 5.40 (d, *J* = 1.8 Hz , 2H), 4.30 – 4.34 (m, 1H), 4.21 – 4.28 (m, 2H), 4.08 – 4.12 (m, 1H), 2.41 (s, 6H), 2.28 (s, 3H), 1.64 – 1.78 (m, 2H), 1.55 – 1.61 (m, 1H), 1.42 – 1.49 (m, 1H), 0.98 – 1.39 (m, 8H), 0.82 – 0.93 (m, 8H). **^{13}C NMR** (150 MHz, $CDCl_3$) δ 166.1, 152.2, 148.4, 148.3, 138.4, 138.36, 129.2, 129.1, 121.3, 121.28, 121.2, 121.19, 118.2, 118.1, 69.6, 69.4, 61.8, 28.6, 28.49, 28.48, 28.0, 27.98, 27.97, 27.5, 27.4, 27.0, 26.9, 24.0, 23.9, 23.8, 23.79, 21.1, 19.69, 19.68. **HRMS** (ESI): m/z calculated for $C_{29}H_{39}B_rNaO_4^+$ [M + Na]$^+$ 553.1924, found 553.1920. $[\alpha]_D^{25}$ = 8.7 (c = 0.30, CH_2Cl_2). **HPLC**: Chiralpak IB N-5 column, 95:5 hexanes/isopropanol, 1.0 mL/min; t_R = 6.1 min (major), 8.4 min (minor), 93:7 er.

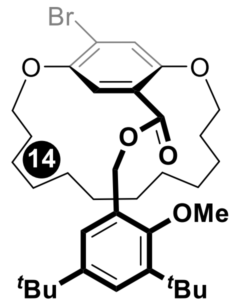

3ae, 47.2 mg, 78% yield, Colorless oil. **^{1}H NMR** (600 MHz, $CDCl_3$) δ 7.44 (s, 1H), 7.35 (q, *J* = 3.0 Hz, 2H), 7.25 (s, 1H), 5.43 (d, *J* = 3.0 Hz, 2H), 4.29 – 4.35 (m , 2H), 4.24 (ddd, *J* = 12.0 , 8.4, 4.2 Hz, 1H), 4.14 (ddd, *J* = 15.0, 7.2, 4.2 Hz, 1H), 3.86 (s, 3H), 1.70 – 1.78 (m, 2H), 1.50 – 1.61 (m, 2H), 1.41 (s, 9H), 1.28 – 1.34 (m, 11H), 1.16 – 1.26 (m, 3H), 1.02 – 1.15 (m, 3H), 0.85 – 0.96 (m, 8H). **^{13}C NMR** (150 MHz, $CDCl_3$) δ 165.7, 156.3, 152.6, 148.5, 145.9, 142.1, 128.6, 125.9, 124.7, 121.7, 121.1, 118.4, 118.0, 69.7, 69.5, 63.2, 62.7, 35.5, 34.6, 31.6, 31.2, 28.6, 28.1, 28.0, 27.5, 27.2, 26.9, 24.0, 23.9. **HRMS** (ESI): m/z calculated for $C_{35}H_{51}BrNaO_5^+$ [M + Na]$^+$ 653.2812, found 653.2819. $[\alpha]_D^{25}$ = 16.6 (c = 0.27, CH_2Cl_2). **HPLC**: Chiralpak IB N-5 column, 95:5 hexanes/isopropanol, 1.0 mL/min; t_R = 4.5 min (major), 5.8 min (minor), 95:5 er.

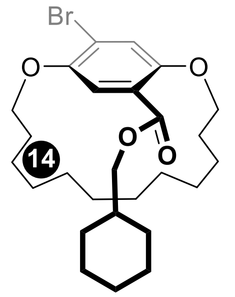

3af, 27.2 mg, 55% yield, Colorless oil. **^{1}H NMR** (600 MHz, $CDCl_3$) δ 7.39 (s, 1H), 7.23 (s, 1H), 4.31 – 4.37 (m , 2H), 4.23 – 4.27 (m, 1H), 4.15 – 4.19 (m, 1H), 4.09 – 4.14 (m, 2H), 1.72 – 1.84 (m, 7H), 1.66 – 1.71 (m, 1H), 1.54 – 1.62 (m, 2H), 1.35 – 1.43 (m, 2H), 1.23 – 1.34 (m, 4H), 1.14 – 1.22 (m, 3H), 1.02 – 1.12 (m, 4H), 0.90 – 0.97 (m, 8H). **^{13}C NMR** (150 MHz, $CDCl_3$) δ 166.0, 152.2, 148.4, 121.4, 118.2, 118.1, 70.4, 69.6, 69.5, 37.4, 29.9, 28.6, 28.1, 28.0, 27.6, 27.1, 27.0, 26.5, 25.87, 25.86, 23.98, 23.95. **HRMS** (ESI): m/z calculated for $C_{26}H_{39}BrNaO_4^+$ [M + Na]$^+$ 517.1924, found 517.1920. $[\alpha]_D^{25}$ = 10.5 (c = 0.73, CH_2Cl_2). **HPLC**: Chiralpak IB N-5 column, 95:5 hexanes/isopropanol, 1.0 mL/min; t_R = 5.2 min (major), 6.2 min (minor), 95:5 er.

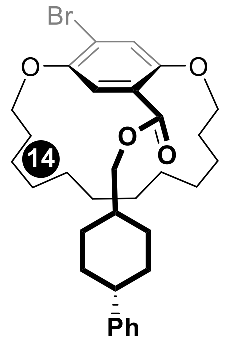

3ag, 38.7 mg, 68% yield, Colorless oil. **^{1}H NMR** (600 MHz, $CDCl_3$) δ 7.46 (s, 1H), 7.31 (t, J = 7.8 Hz, 2H), 7.27 (s, 1H), 7.24 – 7.25 (m , 2H), 7.20 (t, J = 7.2 Hz, 1H), 5.37 – 5.38 (m, 1H), 4.36 – 4.42 (m, 2H), 4.27 (ddd, J = 12.6, 8.4, 4.2 Hz, 1H), 4.22 (ddd, J = 12.0, 8.4, 3.6 Hz, 1H), 2.61 –2.65 (m, 1H), 2.13 – 2.19 (m, 2H), 1.86 – 1.98 (m, 2H), 1.72 – 1.85 (m, 6H), 1.53 – 1.65 (m, 2H), 1.07 – 1.46 (m, 9H), 0.92 – 0.98 (m, 9H). **^{13}C NMR** (150 MHz, $CDCl_3$) δ 165.2, 152.1, 148.2, 148.17, 147.3, 128.5, 126.8, 126.2, 121.8, 121.3, 117.9, 69.9, 69.4, 43.6, 30.6, 30.5, 28.8, 28.6, 28.5, 28.02, 27.96, 27.6, 27.5, 26.8, 26.7, 24.0, 23.9. **HRMS** (ESI): m/z calculated for $C_{32}H_{43}BrNaO_4^+$ [M + Na]$^+$ 593.2237, found 593.2242. $[\alpha]_D^{25}$ = 17.0 (c = 0.33, CH_2Cl_2). **HPLC**: Chiralpak IB N-5 column, 95:5 hexanes/isopropanol, 1.0 mL/min; t_R = 7.7 min (major), 12.6 min (minor), 97.5:2.5 er.

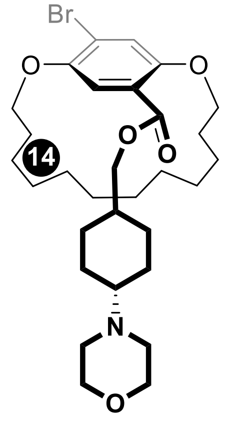

3ah, 41.0 mg, 78% yield, Colorless oil. **^{1}H NMR** (600 MHz, $CDCl_3$) δ 7.39 (s, 1H), 7.23 (s, 1H), 4.89 – 4.94 (m , 1H), 4.28 – 4.36 (m, 2H), 4.23 – 4.27 (m, 1H), 4.14 – 4.18 (m, 1H), 3.74 (s, 4H), 2.58 (s, 3H), 2.17 – 2.20 (m, 2H), 2.00 – 2.04 (m, 2H), 1.70 – 1.79 (m, 2H), 1.57 – 1.63 (m, 2H), 1.49 – 1.53 (m, 2H), 1.35 – 1.46 (m, 4H), 1.24 – 1.34 (m, 5H), 1.13 – 1.20 (m, 2H), 1.06 – 1.11 (m, 2H), 0.93 – 0.95 (m, 8H). **^{13}C NMR** (150 MHz, $CDCl_3$) δ 165.4, 152.3, 148.6, 121.6, 118.1, 69.8, 69.6, 50.1, 31.8, 30.5, 30.4, 29.83, 29.81, 28.6, 28.2, 28.1, 27.53, 27.51, 27.4, 27.1, 26.3, 24.1, 24.0.

HRMS (ESI): m/z calculated for $C_{30}H_{46}BrNNaO_5^+$ $[M + Na]^+$ 602.2452, found 602.2457. $[\alpha]_D^{25}$ = 6.1 (c = 0.31, CH_2Cl_2). **HPLC**: Chiralpak IB N-5 column, 95:5 hexanes/isopropanol, 1.0 mL/min; t_R = 20.9 min (major), 24.4 min (minor), 99:1 er.

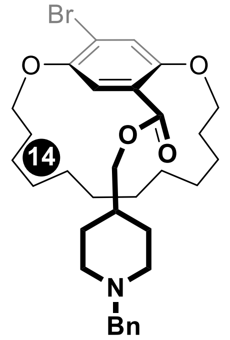

3ai, 27.0 mg, 48% yield, Colorless oil. **^{1}H NMR** (600 MHz, $CDCl_3$) δ 7.38 (s, 1H), 7.31 – 7.34 (m, 4H), 7.26 (s, 1H), 7.23 (s, 1H), 4.30 – 4.37 (m, 2H), 4.22 – 4.27 (m, 1H), 4.13 – 4.17 (m, 1H), 3.55 (s, 2H), 2.73 (s, 2H), 2.39 (s, 2H), 2.02 (s, 2H), 1.73 – 1.89 (m, 4H), 1.55 – 1.63 (m, 2H), 1.30 – 1.40 (m, 4H), 1.05 – 1.22 (m, 4H), 0.87 – 0.98 (m, 9H). **^{13}C NMR** (150 MHz, $CDCl_3$) δ 165.2, 152.3, 148.4, 129.3, 128.4, 121.6, 121.4, 118.2, 118.1, 69.6, 69.5, 63.2, 50.7, 28.6, 28.14, 28.06, 27.6, 27.2, 27.0, 24.1, 24.0. **HRMS** (ESI): m/z calculated for $C_{31}H_{42}BrNNaO_5^+$ $[M + Na]^+$ 594.2189, found 594.2180. $[\alpha]_D^{25}$ = 16.9 (c = 0.53, CH_2Cl_2). **HPLC**: Chiralpak IA column, 80:20 hexanes/isopropanol, 1.0 mL/min; t_R = 11.6 min (major), 16.1 min (minor), 93:7 er.

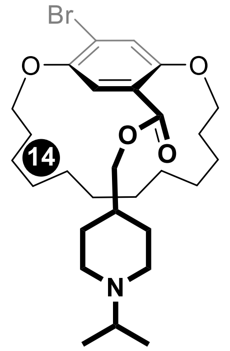

3aj, 26.7 mg, 51% yield, Colorless oil. **^{1}H NMR** (600 MHz, $CDCl_3$) δ 7.37 (d, J = 2.4 Hz, 1H), 7.21 (d, J = 2.4 Hz, 1H), 5.00 – 5.06 (m, 1H), 4.13 – 4.37 (m, 4H), 2.73 – 2.81 (m, 3H), 2.41 – 2.47 (m, 2H), 2.01 – 2.06 (m, 2H), 1.70 – 1.88 (m, 4H), 1.52 – 1.61 (m, 2H), 1.24 – 1.41 (m, 6H), 1.05 – 1.07 (m, 9H), 0.92 – 0.93 (m, 9H). **^{13}C NMR** (150 MHz, $CDCl_3$) δ 165.2, 152.2, 148.3, 121.6, 121.4, 118.1, 118.0, 69.6, 69.4, 54.7, 46.0, 31.3, 28.6, 28.56, 28.1, 28.0, 27.5, 27.1, 26.9, 24.0, 23.9, 18.5. **HRMS** (ESI): m/z calculated for $C_{28}H_{44}BrNNaO_4^+$ $[M + Na]^+$ 560.2346, found 560.2340. $[\alpha]_D^{25}$ = 8.7 (c = 0.67, CH_2Cl_2). **HPLC**: Chiralpak IB N-5 column, 90:10 hexanes/isopropanol, 1.0 mL/min; t_R = 5.7 min (major), 6.3 min (minor), 92.5:7.5 er.

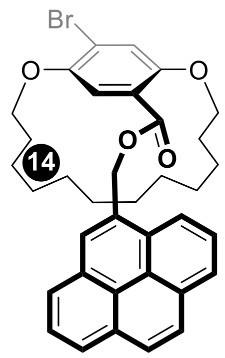

3ak, 57.5 mg, 94% yield, white solid. **^{1}H NMR** (600 MHz, $CDCl_3$) δ 8.41 (d, *J* = 9.0 Hz, 1H), 8.21 (dd, *J* = 7.2, 3.0 Hz, 2H), 8.15 – 8.18 (m, 3H), 8.02 – 8.10 (m, 3H), 7.43 (s, 1H), 7.19 (m, 1H), 6.04 – 6.16 (m, 2H), 4.26 – 4.29 (m, 1H), 4.16 – 4.23 (m, 2H), 4.04 – 4.08 (m, 1H), 1.65 – 1.72 (m, 1H), 1.47 – 1.53 (m, 1H), 1.21 – 1.41 (m, 4H), 1.05 – 1.14 (m, 2H), 0.93 – 1.01 (m, 2H), 0.84 – 0.91 (m, 3H), 0.73 – 0.79 (m, 7H). **^{13}C NMR** (150 MHz, $CDCl_3$) δ 165.7, 152.5, 148.4, 131.9, 131.3, 130.8, 129.8, 129.2, 128.2, 128.0, 127.9, 127.5, 126.2, 125.6, 125.5, 125.1, 124.8, 124.7, 123.3, 121.4, 120.9, 118.6, 118.2, 69.6, 69.5, 28.5, 28.4, 27.9, 27.5, 27.3, 26.9, 26.86, 26.8, 23.9, 23.7. **HRMS** (ESI): m/z calculated for $C_{36}H_{37}BrNaO_4^+$ [M + Na]$^+$ 635.1767, found 635.1762. $[\alpha]_D^{25}$ = 23.4 (c = 0.64, CH_2Cl_2). **HPLC**: Chiralpak IB N-5 column, 95:5 hexanes/isopropanol, 1.0 mL/min; t_R = 17.4 min (major), 33.6 min (minor), 79:21 er.

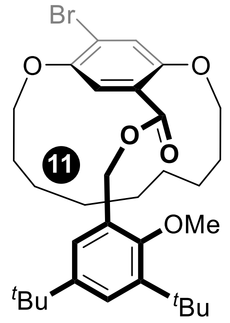

3be, 12.4 mg, 21% yield, Colorless oil. **^{1}H NMR** (600 MHz, $CDCl_3$) δ 7.58 (s, 1H), 7.37 (d, *J* = 2.4 Hz, 1H), 7.34 (d, *J* = 3.0 Hz, 1H), 7.33 (s, 1H), 5.44 (d, *J* = 1.2 Hz, 2H), 4.36 (ddd, *J* = 10.2, 7.2, 3.6 Hz, 1H), 4.24 – 4.27 (m, 1H), 4.18 – 4.22 (m, 2H), 3.85 (s, 3H), 1.75 – 1.82 (m, 1H), 1.63 – 1.69 (m, 1H), 1.49 – 1.61 (m, 2H), 1.41 (s, 9H), 1.30 (s, 9H), 0.72 – 1.03 (m, 10H). **^{13}C NMR** (150 MHz, $CDCl_3$) δ 165.2, 156.3, 151.3, 145.9, 142.1, 127.0, 125.8, 124.7, 123.9, 123.1, 120.9, 73.2, 72.4, 63.3, 62.8, 31.6, 29.9, 29.7, 27.9, 26.1, 26.0, 25.5, 25.0. **HRMS** (ESI): m/z calculated for $C_{32}H_{45}BrNaO_5^+$ [M + Na]$^+$ 611.2343, found 611.2340. $[\alpha]_D^{25}$ = 13.1 (c = 0.01, CH_2Cl_2). **HPLC**: Chiralpak IB N-5 column, 95:5 hexanes/isopropanol, 1.0 mL/min; t_R = 4.7 min (major), 6.0 min (minor); 86:14 er.

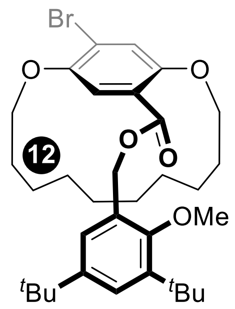

3ce, 25.3 mg, 42% yield, Colorless oil. **^{1}H NMR** (600 MHz, $CDCl_3$) δ 7.49 (s, 1H), 7.34 – 7.36 (m, 2H), 7.29 (s, 1H), 5.44 (s, 2H), 4.39 (ddd, *J* = 12.1, 6.1, 3.7 Hz, 1H), 4.33 (ddd, *J* = 12.3, 6.1, 3.7 Hz, 1H), 4.27 (ddd, *J* = 12.3, 8.7, 3.7 Hz, 1H), 4.14 (ddd, *J* = 12.3, 8.8, 3.5 Hz,1H), 3.85 (s, 3H), 1.65 – 1.73 (m, 2H), 1.48 – 1.62 (m, 2H), 1.41 (s, 9H), 1.30 (s, 9H), 1.21 – 1.26 (m, 2H), 1.07 – 1.20 (m, 4H), 0.87 – 1.02 (m, 3H), 0.72 – 0.77 (m, 4H). **^{13}C NMR** (150 MHz, $CDCl_3$) δ 165.5, 156.3, 153.7, 145.9, 142.1, 128.6, 125.8, 124.7, 124.6, 120.5, 119.6, 71.1, 70.4, 63.3, 62.7, 35.5, 34.7, 31.7, 31.2, 28.1, 27.71, 27.67, 27.5, 27.4, 27.3, 24.0, 23.7. **HRMS** (ESI): m/z calculated for $C_{33}H_{47}BrNaO_5^+$ $[M + Na]^+$ 625.2499, found 625.2490. $[\alpha]_D^{25}$ = 38.3 (c = 0.48, CH_2Cl_2). **HPLC**: Chiralpak IB N-5 column, 95:5 hexanes/isopropanol, 1.0 mL/min; t_R = 4.5 (major), 6.2 min (minor), 91.5:8.5 er.

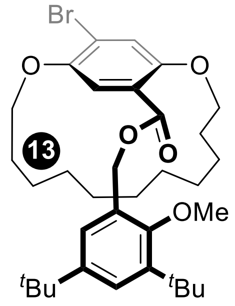

3de, 37.6 mg, 61% yield, Colorless oil. **^{1}H NMR** (600 MHz, $CDCl_3$) δ 7.48 (s, 1H), 7.34 – 7.36 (m, 2H), 7.28 (s, 1H), 5.43 (d, *J* = 2.4, 2H), 4.27 – 4.37 (m, 3H), 4.19 – 4.23 (m, 1H), 3.86 (s, 3H), 1.55 – 1.72 (m, 4H), 1.41 (s, 9H), 1.31 (s, 9H), 1.21 – 1.26 (m, 2H), 1.05 – 1.20 (m, 4H), 0.85 – 1.04 (m, 6H), 0.72 – 0.78 (m, 2H). **^{13}C NMR** (150 MHz, $CDCl_3$) δ 165.6, 156.3, 153.6, 149.5, 145.9, 128.6, 125.9, 124.7, 122.9, 121.9, 119.2, 119.0, 70.5, 70.3, 63.3, 62.7, 35.5, 34.7, 31.6, 31.2, 28.73, 28.67, 28.6, 28.3, 27.31, 27.29, 27.1, 24.2, 24.1. **HRMS** (ESI): m/z calculated for $C_{34}H_{49}BrNaO_5^+$ $[M + Na]^+$ 639.2656, found 639.2661. $[\alpha]_D^{25}$ = 20.0 (c = 0.2, CH_2Cl_2). **HPLC**: Chiralpak IB N-5 column, 95:5 hexanes/isopropanol, 1.0 mL/min; t_R = 4.5 min (major), 6.1 min (minor), 95:5 er.

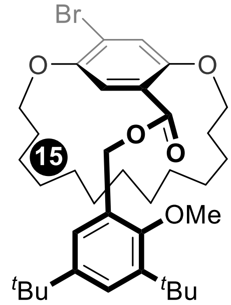

3ee, 45.3 mg, 72% yield, Colorless oil. **^{1}H NMR** (600 MHz, $CDCl_3$) δ 7.45 (s, 1H), 7.35 (q, J = 2.4Hz, 2H), 7.24 (s, 1H), 5.42 (d, J = 3.6 Hz, 2H), 4.20 – 4.29 (m, 3H), 4.12 – 4.16 (m, 1H), 3.86 (s, 3H), 1.57 – 1.69 (m, 4H), 1.41 (s, 9H), 1.32 – 1.37 (m, 2H), 1.31 (s, 9H), 1.18 – 1.29 (m, 4H), 0.91 – 1.14 (m, 12H). **^{13}C NMR** (150 MHz, $CDCl_3$) δ 165.7, 152.8, 148.8, 145.9, 142.1, 128.6, 125.9, 124.7, 121.0, 120.9, 118.4, 117.5, 69.5, 69.4, 63.2, 62.7, 35.5, 31.6, 31.2, 29.5, 28.5, 28.4, 28.21, 28.18, 27.8, 27.7, 27.45, 27.36, 24.1, 23.9. **HRMS** (ESI): m/z calculated for $C_{36}H_{53}BrNaO_5^+$ $[M + Na]^+$ 667.2969, found 667.2979. $[\alpha]_D^{25}$ = 5.6 (c = 0.36, CH_2Cl_2). **HPLC**: Chiralpak IB N-5 column, 95:5 hexanes/isopropanol, 1.0 mL/min; t_R = 4.5 min (major), 5.7 min (minor), 95:5 er.

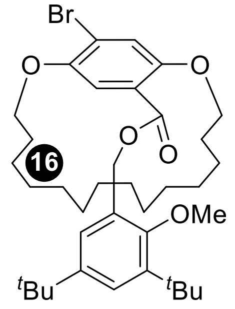

3fe, 46 mg, 70% yield, Colorless oil. **^{1}H NMR** (600 MHz, $CDCl_3$) δ 7.52 (s, 1H), 7.35 (d, J = 4.8 Hz, 2H), 7.10 (s, 1H), 5.43 (d, J = 3.0 Hz, 2H), 4.27– 4.36 (m, 3H), 4.19 – 4.23 (m, 1H), 3.85 (s, 3H), 1.58 – 1.70 (m, 4H), 1.41 (s, 9H), 1.31 (s, 11H), 0.85 – 1.26 (m, 16H), 0.73 – 0.80 (m, 2H). **^{13}C NMR** (150 MHz, $CDCl_3$) δ 165.5, 156.3, 153.6, 148.5, 145.9, 142.1, 128.6, 125.9, 124.7, 121.2, 120.0, 119.9, 70.5, 70.4, 63.3, 62.8, 35.5, 31.7, 28.6, 28.4, 27.3, 27.2, 24.2, 24.1. **HRMS** (ESI): m/z calculated for $C_{37}H_{55}BrNaO_5^+$ $[M + Na]^+$ 681.3125, found 681.3129.

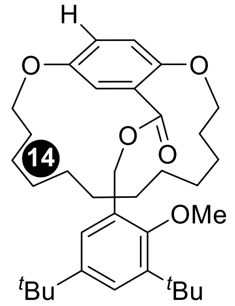

3ge, 33.1 mg, 60% yield, Colorless oil. **^{1}H NMR** (600 MHz, $CDCl_3$) δ 7.34 – 7.39 (m, 3H), 7.03 (d, J = 9.6 Hz, 1H), 6.96 (d, J = 9.0 Hz, 1H), 5.42 (s, 2H), 4.17 – 4.24 (m, 4H), 3.87 (s, 3H), 1.58 – 1.65 (m, 4H), 1.41 (s, 9H), 1.30 – 1.35 (m, 11H), 1.25 – 1.29 (m, 2H), 1.16 – 1.14 (m, 4H), 0.91 – 0.92 (m, 8H). **^{13}C NMR** (150 MHz, $CDCl_3$) δ 166.3, 156.4, 152.6, 151.8, 145.9, 142.0, 128.8, 126.1, 124.7, 122.5, 122.0, 118.8, 118.0, 77.4, 69.6, 68.5, 63.0, 62.8, 35.5, 34.7, 31.7, 31.2, 28.6, 28.5, 28.00, 27.98, 27.6, 27.5, 27.4, 27.2, 24.1, 24.0. **HRMS** (ESI): m/z calculated for $C_{35}H_{52}NaO_5^+$ $[M + Na]^+$ 575.3707, found 575.3718.

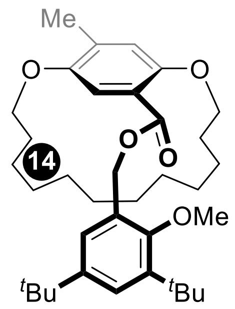

3he, 39.4 mg, 66% yield, Colorless oil. **^{1}H NMR** (600 MHz, $CDCl_3$) δ 7.38 (d, J = 3.0 Hz,1H), 7.37 (s, 1H), 7.33 (d, J = 2.4 Hz, 1H), 6.84 (s, 1H), 5.42 (s, 2H), 4.29 – 4.34 (m, 2H), 4.09 – 4.14 (m, 2H), 3.87 (s, 3H), 2.25 (s, 3H), 1.67 – 1.80 (m, 2H), 1.47 – 1.58 (m, 2H), 1.41 (s, 9H), 1.31 (s, 11H), 1.15 – 1.23 (m, 2H), 0.86 – 1.12 (m, 12H). **^{13}C NMR** (150 MHz, $CDCl_3$) δ 166.3, 156.3, 152.6, 150.0, 145.8, 142.0, 134.0, 129.0, 125.9, 124.5, 119.8, 119.2, 116.1, 69.6, 68.2, 62.8, 62.7, 35.5, 34.7, 31.7, 31.2, 28.6, 28.5, 28.14, 28.05, 27.60, 27.58, 27.5, 27.0, 24.1, 17.0. **HRMS** (ESI): m/z calculated for $C_{36}H_{54}NaO_5^+$ $[M + Na]^+$ 589.3863, found 589.3859. $[\alpha]_D^{25}$ = 19.4 (c = 0.067, CH_2Cl_2). **HPLC**: Chiralpak IB N-5 column, 95:5 hexanes/isopropanol, 1.0 mL/min; t_R = 4.9 min (major), 5.7 min (minor); 95:5 er.

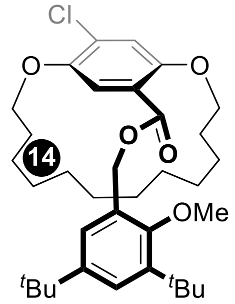

3ie, 33.5 mg, 57% yield, Colorless oil. **^{1}H NMR** (600 MHz, $CDCl_3$) δ 7.49 (s, 1H), 7.35 (s, 2H), 7.07 (s, 1H), 5.43 (d, J = 3.3 Hz, 2H), 4.28 – 4.34 (m, 2H), 4.23 – 4.26 (m, 1H), 4.14 – 4.17 (m, 1H), 3.86 (s, 3H), 1.69 – 1.78 (m, 2H), 1.52 – 1.60 (m, 2H), 1.41 (s, 9H), 1.30 (s, 10H), 1.04 – 1.26 (m, 6H), 0.93 (s, 9H). **^{13}C NMR** (150 MHz, $CDCl_3$) δ 165.6, 156.3, 152.7, 147.5, 145.9, 142.1, 129.0, 128.7, 125.9, 124.7, 120.5, 118.8, 118.6, 69.7, 69.6, 63.2, 62.7, 35.5, 34.7, 31.7, 31.2, 28.60, 28.57, 28.10, 28.06, 27.6, 27.5, 27.3, 27.0, 24.0, 23.9. **HRMS** (ESI): m/z calculated for $C_{35}H_{51}ClNaO_5^+$ $[M + Na]^+$ 609.3317, found 609.3310. $[\alpha]_D^{25}$ = 5.8 (c = 0.33, CH_2Cl_2). **HPLC**: Chiralpak IB N-5 column, 95:5 hexanes/isopropanol, 1.0 mL/min; t_R = 4.6 min (major), 6.1 min (minor), 95:5 er.

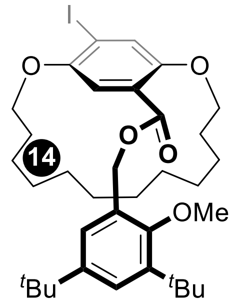

3je, 40.3 mg, 61% yield, Colorless oil. **^{1}H NMR** (600 MHz, $CDCl_3$) δ 7.47 (s, 1H), 7.33 – 7.36 (m, 2H), 7.31 (s, 1H), 5.42 (d, *J* = 2.4 Hz, 2H), 4.28 – 4.34 (m, 2H), 4.20 – 4.24 (m, 1H), 4.10 – 4.14 (m, 1H), 3.85 (s, 3H), 1.69 – 1.77 (m, 2H), 1.49 – 1.63 (m, 2H), 1.41 (s, 10H), 1.30 (s, 9H), 1.17 – 1.25 (m, 3H), 1.02 – 1.14 (m, 3H), 0.87 – 0.98 (m, 9H).**^{13}C NMR** (150 MHz, $CDCl_3$) δ 165.9, 156.3, 152.6, 151.0, 145.9, 142.1, 128.6, 128.0, 125.9, 124.7, 122.1, 116.0, 93.6, 69.9, 69.5, 63.3, 62.7, 35.5, 34.7, 31.7, 31.2, 28.64, 28.6, 28.2, 28.1, 27.6, 27.5, 27.3, 26.9, 24.1, 24.0. **HRMS** (ESI): m/z calculated for $C_{35}H_{51}INaO_5^+$ $[M + Na]^+$ 701.2673, found 701.2663. $[\alpha]_D^{25}$ = 4.6 (c = 0.37, CH_2Cl_2). **HPLC**: Chiralpak IB N-5 column, 95:5 hexanes/isopropanol, 1.0 mL/min; t_R = 4.9 min (major), 7.0 min (minor), 95:5 er.

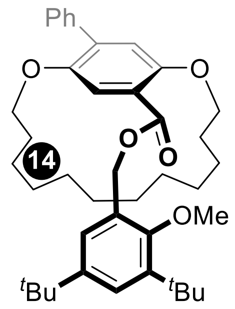

3ke, 28.2 mg, 45% yield, Colorless oil. **^{1}H NMR** (600 MHz, $CDCl_3$) δ 7.50 – 7.56 (m, 3H), 7.39 – 7.44 (m, 3H), 7.33 –7.38 (m, 2H), 7.01 (s, 1H), 5.45 (d, *J* = 2.4 Hz, 2H), 4.36 – 4.40 (m, 1H), 4.14 – 4.21 (m, 2H), 3.94 – 3.98 (m, 1H), 3.89 (s, 3H), 1.77 – 1.84 (m, 1H), 1.64 –1.72 (m, 1H), 1.53 – 1.59 (m,1H), 1.42 (s, 9H), 1.32 (s, 9H), 1.01 – 1.21 (m, 7H), 0.83 – 1.01 (m, 10H). **^{13}C NMR** (150 MHz, $CDCl_3$) δ 166.1, 156.4, 152.6, 148.7, 145.8, 142.0, 137.2, 129.5, 128.9, 128.2, 127.7, 126.0, 124.6, 119.2, 118.8, 69.4, 69.0, 63.0, 62.8, 35.5, 34.7, 31.7, 31.3, 28.64, 28.6, 28.2, 28.0, 27.6, 27.5, 27.4, 27.1, 24.0, 23.9. **HRMS** (ESI): m/z calculated for $C_{41}H_{56}NaO_5^+$ $[M + Na]^+$ 651.4020, found 651.4029. $[\alpha]_D^{25}$ = 13.5 (c = 0.48, CH_2Cl_2). **HPLC**: Chiralpak IB N-5 column, 95:5 hexanes/isopropanol, 1.0 mL/min; t_R = 4.1 min (major), 5.6 min (minor), 95:5 er.

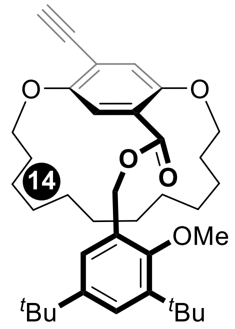

3le, 35.6 mg, 62% yield, Colorless oil. **^{1}H NMR** (400 MHz, $CDCl_3$) δ 7.41(s, 1H), 7.35 (d, *J* = 2.8 Hz, 2H), 7.11 (s, 1H), 5.43 (s, 2H), 4.23 – 4.37 (m, 3H), 4.10 – 4.16 (m, 1H), 3.86 (s, 3H), 3.38 (s, 1H), 1.67 – 1.81 (m, 2H), 1.48 – 1.63 (m, 3H), 1.42 (s, 9H), 1.31 (s, 10H), 1.02 – 1.27 (m, 6H), 0.93 (s, 8H). **^{13}C NMR** (100 MHz, $CDCl_3$) δ 165.8, 156.3, 153.0, 151.8, 145.9, 142.1, 126.0, 124.7, 122.8, 121.6, 117.8, 83.3, 69.5, 69.3, 63.3, 62.8, 35.5, 34.6, 31.6, 31.2, 28.6, 28.57, 28.08, 28.06, 27.6, 27.5, 27.2, 27.1, 24.0, 23.9. **HRMS** (ESI): m/z calculated for $C_{37}H_{52}NaO_5^+$ $[M + Na]^+$ 599.3707, found 599.3700. $[\alpha]_D^{25}$ = 42.7 (c = 0.05, CH_2Cl_2). **HPLC**: Chiralpak IB N-5 column, 95:5

hexanes/isopropanol, 1.0 mL/min; t_R = 7.1 min (major), 9.6 min (minor), 95:5 er.

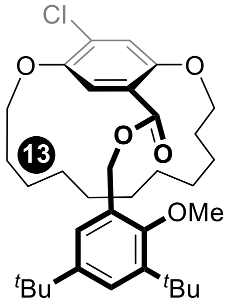

5ae, 34.2 mg, 60% yield, Colorless oil. ^{1}H NMR (600 MHz, $CDCl_3$) δ 7.43 (s, 1H), 7.35 (d, J = 2.4 Hz, 2H), 7.22 (s,1H), 5.22 (d, J = 1.2 Hz, 2H), 4.11 – 4.21 (m, 4H), 3.86 (s, 3H), 1.63 – 1.77 (m, 4H), 1.52 – 1.58 (m, 1H),1.41 (s, 9H), 1.31 (s, 9H), 1.16 – 1.25 (m, 5H), 1.06 – 1.14 (m, 4H), 1.01 – 1.05 (m, 4H). **^{13}C** NMR (150 MHz, $CDCl_3$) δ 165.7, 156.3, 152.7, 148.6, 145.9, 142.1, 128.7, 125.9, 124.7, 120.3, 118.0, 116.8, 69.0, 63.2, 62.7, 35.5, 34.6, 31.7, 31.2, 29.1, 29.00, 28.95, 28.41, 28.35, 28.2, 28.0, 27.5, 27.4, 27.0, 26.9, 24.0, 23.7. **HRMS** (ESI): m/z calculated for $C_{34}H_{49}ClNaO_5^+$ $[M + Na]^+$ 595.3161, found 595.3169. $[\alpha]_D^{25}$ = 14.0 (c = 0.33, CH_2Cl_2). **HPLC**: Chiralpak IB N-5 column, 95:5 hexanes/isopropanol, 1.0 mL/min; t_R = 4.6 min (major), 6.7min (minor), 95:5 er.

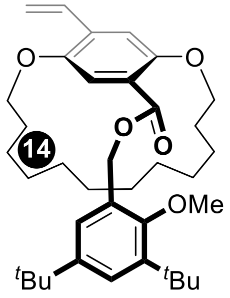

5be, 38.1 mg, 66% yield, Colorless oil. **^{1}H NMR** (600 MHz, $CDCl_3$) δ 7.42 (s, 1H), 7.38 (d, J = 2.4 Hz, 1H), 7.33 – 7.35 (m, 1H), 7.13 (s, 1H), 7.05 (dd, J = 18.0, 11.4 Hz, 1H), 5.78 (dd, J = 17.4, 1.2 Hz, 1H), 5.43 (s, 2H), 5.37 (dd, J = 11.4, 1.2 Hz, 1H), 4.34 – 4.38 (m, 2H), 4.11 – 4.16 (m, 2H), 3.86 (s, 3H), 1.71 – 1.77 (m, 2H), 1.49 – 1.59 (m, 2H), 1.41 (s, 9H), 1.31 (s, 9H), 1.01 – 1.27 (m, 7H), 0.88 – 0.93 (m, 9H). **^{13}C NMR** (150 MHz, $CDCl_3$) δ 166.0, 156.3, 152.7, 149.1, 132.9, 131.4, 128.9, 125.9, 124.6, 117.7, 116.7, 114.9, 69.7, 68.8, 63.0, 62.7, 35.5, 34.7, 31.7, 31.2, 28.5, 28.1, 28.0, 27.6, 27.5, 27.1, 24.1. **HRMS** (ESI): m/z calculated for $C_{37}H_{55}O_5^+$ $[M + H]^+$ 579.4044, found 579.4040. $[\alpha]_D^{25}$ = 19.0 (c = 0.1, CH_2Cl_2). **HPLC**: Chiralpak IB N-5 column, 95:5 hexanes/isopropanol, 1.0 mL/min; t_R = 5.0 min (major), 6.5 min (minor), 90:10 er.

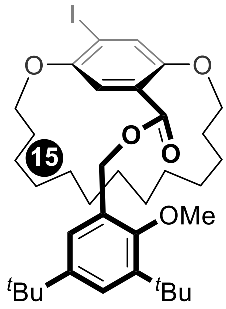

5ce, 41.5 mg, 60% yield, Colorless oil. **^{1}H NMR** (600 MHz, $CDCl_3$) δ 7.47 (s, 1H), 7.34 – 7.35 (m, 2H), 7.31 (s, 1H), 5.42 (d, J = 2.4 Hz, 2H), 4.18 – 4.27 (m, 3H), 4.11 – 4.15 (m, 1H), 3.85 (s, 3H), 1.57 – 1.69 (m, 4H), 1.43 – 1.51 (m, 1H), 1.41 (s, 9H), 1.30 (s, 9H), 0.91 – 1.30 (m, 17H). **^{13}C NMR** (150 MHz, $CDCl_3$) δ 165.9, 156.3, 152.8, 151.2, 145.9, 142.1, 128.6, 127.3, 125.9, 124.7, 124.6, 122.0, 121.9, 115.5, 93.5, 69.5, 69.4, 63.2, 62.7, 35.5, 34.7, 31.7, 31.6, 31.20, 31.19, 29.5, 28.5, 28.4, 28.3, 28.2, 27.9, 27.6, 27.4, 27.36, 24.3, 24.0. **HRMS** (ESI): m/z calculated for $C_{36}H_{53}INaO_5^+$ $[M + Na]^+$ 715.2830, found 715.2820. $[\alpha]_D^{25}$ = 6.6 (c = 0.17, CH_2Cl_2). **HPLC**: Chiralpak IB N-5 column, 95:5 hexanes/isopropanol, 1.0 mL/min; t_R = 4.9 min (major), 6.8 min (minor), 95:5 er.

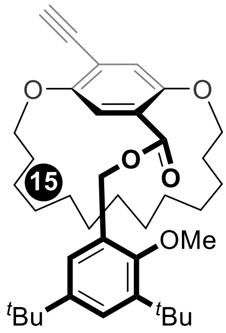

5de, 36.4 mg, 61% yield, Colorless oil. **^{1}H NMR** (400 MHz, $CDCl_3$) δ 7.41 (s, 1H), 7.34 – 7.36 (m, 2H), 7.10 (s, 1H), 5.42 (s, 2H), 4.21 – 4.32 (m, 3H), 4.11– 4.15 (m, 1H), 3.86 (s, 3H), 3.38 (s, 1H), 1.56 – 1.71 (m, 4H), 1.42 – 1.51 (m, 1H), 1.41 (s, 9H), 1.30 (s, 9H), 1.18 – 1.28 (m, 4H), 0.92 – 1.14 (m, 13H). **^{13}C NMR** (100 MHz, $CDCl_3$) δ 165.6, 156.3, 152.8, 147.7, 145.8, 142.1, 128.9, 128.6, 126.0, 124.7, 120.1, 118.2, 117.8, 69.5, 69.3, 63.2, 62.7, 35.5, 34.6, 31.6, 31.2, 29.5, 28.4, 28.37, 28.2, 28.1, 27.7, 27.6, 27.4, 27.3, 24.0, 23.9. **HRMS** (ESI): m/z calculated for $C_{38}H_{54}NaO_5^+$ $[M + Na]^+$ 613.3863, found 613.3860. $[\alpha]_D^{25}$ = 18.3 (c = 0.30, CH_2Cl_2). **HPLC**: Chiralpak IB N-5 column, 95:5 hexanes/isopropanol, 1.0 mL/min; t_R = 6.8 min (major), 9.1 min (minor), 95:5 er.

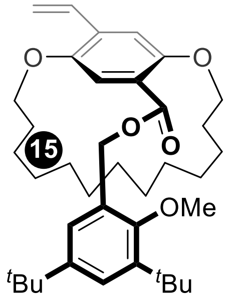

5ee, 33.8 mg, 57% yield, Colorless oil. **^{1}H NMR** (600 MHz, $CDCl_3$) δ 7.42 (s, 1H), 7.38 (d, J = 2.4 Hz, 1H), 7.34 (d, J = 2.4 Hz, 1H), 7.13 (s, 1H), 7.06 (dd, J = 18.0, 11.4 Hz, 1H), 5.78 (dd, J = 17.4, 1.2 Hz, 1H), 5.43 (s, 2H), 5.37 (dd, J = 10.8, 1.2 Hz, 1H), 4.29 – 4.33 (m, 1H), 4.23 – 4.27 (m, 1H), 4.12 – 4.16 (m, 2H), 3.87 (s, 3H), 1.60 – 1.69 (m, 4H), 1.41 (s, 9H), 1.31 (s, 9H), 0.88 – 1.28 (m, 18H). **^{13}C NMR** (150 MHz, $CDCl_3$) δ 166.1, 156.3, 152.9, 149.4, 145.8, 142.0, 132.8, 131.5, 128.9, 125.9, 124.6, 121.0, 117.1, 116.6, 114.1, 69.3, 68.6, 63.0, 62.7, 35.5, 34.7, 31.7, 31.2, 29.4, 28.4, 28.2, 28.1, 28.0, 27.8, 27.4, 24.2, 24.1. **HRMS** (ESI): m/z calculated for $C_{38}H_{56}NaO_5^+$ $[M + Na]^+$ 615.4020, found 615.4029. $[\alpha]_D^{25}$ = 35.7 (c = 0.14, CH_2Cl_2). **HPLC**: Chiralpak IB N-5 column, 95:5 hexanes/isopropanol, 1.0 mL/min; t_R = 5.1 min (major), 6.8 min (minor), 95.5:4.5 er.

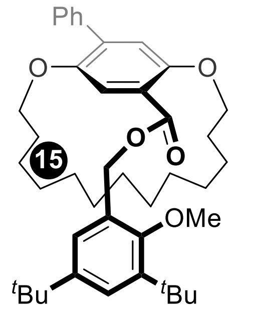

5fe, 39.8 mg, 62% yield, Colorless oil. **^{1}H NMR** (600 MHz, $CDCl_3$) δ 7.53 (d, *J* = 6.6 Hz, 3H), 7.40 – 7.43 (m, 3H), 7.34 – 7.37 (m, 2H), 7.01 (s, 1H), 5.45 (d, *J* = 1.8 Hz, 2H), 4.32 – 4.43 (m, 1H), 4.13 – 4.18 (m, 2H), 3.97 – 4.01 (m, 1H), 3.89 (s, 3H), 1.46 – 1.72 (m, 4H), 1.42 (s, 9H), 1.32 (s, 9H), 0.93 – 1.27 (m, 18H). **^{13}C NMR** (150 MHz, $CDCl_3$) δ 166.1, 156.4, 152.9, 149.1, 145.8, 142.0, 138.0, 137.1, 129.6, 128.9, 128.1, 127.7, 126.0, 124.6, 120.6, 118.6, 118.0, 69.3, 68.9, 63.0, 62.8, 35.5, 31.7, 31.3, 29.5, 28.6, 28.4, 28.3, 28.2, 28.1, 27.8, 27.6, 27.4, 24.2, 24.1. **HRMS** (ESI): m/z calculated for $C_{42}H_{58}NaO_5^+$ $[M + Na]^+$ 665.4176, found 665.4186. $[\alpha]_D^{25}$ = 10.0 (c = 0.1, CH_2Cl_2). **HPLC**: Chiralpak IB N-5 column, 95:5 hexanes/isopropanol, 1.0 mL/min; t_R = 4.1 min (major), 6.0 min (minor), 90:10 er.

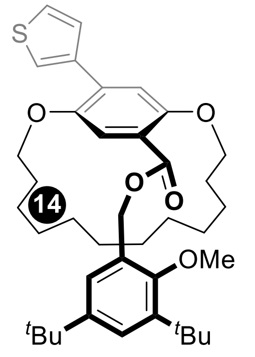

5ge, 50.5 mg, 79% yield, Colorless oil. **^{1}H NMR** (600 MHz, $CDCl_3$) δ 7.72 – 7.73 (m, 1H), 7.53 (d, *J* = 4.8 Hz, 1H), 7.44 – 7.46 (m, 1H), 7.40 (d, *J* = 4.2 Hz, 1H), 7.34 – 7.37 (m, 2H), 7.19 (s, 1H) 5.45 (d, *J* = 4.8 Hz, 2H), 4.28 – 4.41 (m, 2H), 4.09 – 4.20 (m, 2H), 3.89 (s, 3H), 1.70-1.81 (m, 2H), 1.48 – 1.60 (m, 2H), 1.42 (s, 9H), 1.32 (s, 9H), 1.04 – 1.22 (m, 7H), 0.89 – 0.91 (m, 9H).**^{13}C NMR** (150 MHz, $CDCl_3$) δ 152.9, 149.1, 142.0, 137.7, 131.1, 128.9, 128.5, 126.0, 124.80, 124.79, 124.6, 120.2, 117.7, 117.6, 69.5, 68.8, 63.0, 34.7, 31.7, 31.3, 29.4, 28.5, 28.4, 28.3, 28.1, 27.8, 27.5, 27.4, 24.21, 24.2. **HRMS** (ESI): m/z calculated for $C_{39}H_{55}O_5S^+$ $[M + H]^+$ 635.3765, found 635.3760. $[\alpha]_D^{25}$ = 1.0 (c = 0.1, CH_2Cl_2). **HPLC**: Chiralpak IB N-5 column, 95:5 hexanes/isopropanol, 1.0 mL/min; t_R = 5.9 min (major), 10.2 min (minor), 95:5 er

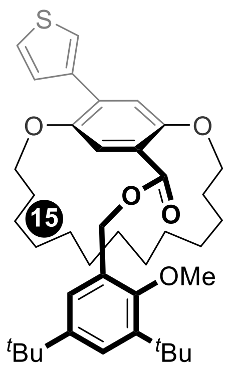

5he, 37.6 mg, 58% yield, Colorless oil. **^{1}H NMR** (600 MHz, $CDCl_3$) δ 7.73 (dd, J = 3.0, 1.2 Hz, 1H), 7.53 (s, 1H), 7.46 (dd, J = 4.8, 1.2 Hz, 1H), 7.40 (d, J = 2.4 Hz, 1H), 7.35 – 7.36 (m, 2H), 7.18 (s, 1H), 5.45 (s, 2H), 4.31 – 4.35 (m, 1H), 4.23 – 4.27 (m, 1H), 4.11 – 4.19 (m, 2H), 3.89 (s, 3H), 1.58 – 1.69 (m, 4H), 1.42 (s, 9H), 1.32 (s, 9H), 1.14 – 1.29 (m, 5H), 0.87 – 1.13 (m, 13H). **^{13}C NMR** (150 MHz, $CDCl_3$) δ 166.1, 156.4, 152.9, 149.1, 142.0, 137.7, 131.1, 128.9, 128.5, 126.0, 124.81, 124.79, 124.6, 120.2, 117.7, 117.6, 69.5, 68.8, 63.0, 62.8, 35.5, 34.7, 31.7, 31.3, 29.4, 28.5, 28.4, 28.3, 28.1, 27.8, 27.5, 27.4. **HRMS** (ESI): m/z calculated for $C_{40}H_{55}NaO_5S^+$ $[M + Na]^+$ 671.3741, found 671.3749. $[\alpha]_D^{25}$ = 16.9 (c = 0.26, CH_2Cl_2). **HPLC**: Chiralpak IB N-5 column, 95:5 hexanes/isopropanol, 1.0 mL/min; t_R = 5.4 min (major), 9.2 min (minor), 90:10 er.

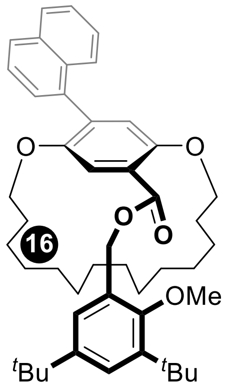

5ie, 48.1 mg, 68% yield, Colorless oil. **^{1}H NMR** (600 MHz, $CDCl_3$) δ 7.99 (d, J = 1.8 Hz, 1H), 7.87 – 7.90 (m, 3H), 7.69 (dd, J = 8.5, 1.7 Hz, 1H), 7.56 (s, 1H), 7.50 – 7.52 (m, 2H), 7.43 (d, J = 3.0 Hz, 1H), 7.43 (d, J = 3.0 Hz, 1H), 7.10 (s, 1H), 5.48 (s, 2H), 4.30 – 4.34 (m, 1H), 4.10 – 4.20 (m, 2H), 3.99 – 4.03 (m, 1H), 3.91 (s, 3H), 1.57 – 1.78 (m, 4H), 1.44 (s, 9H), 1.34 (s, 9H), 1.05 – 1.25 (m, 20H). **^{13}C NMR** (150 MHz, $CDCl_3$) δ 166.1, 152.8, 149.2, 145.8, 142.0, 136.6, 133.4, 132.8, 128.9, 128.4, 128.3, 127.8, 127.78, 127.4, 126.3, 126.2, 126.0, 124.6, 120.3, 118.4, 117.2, 69.0, 68.3, 63.0, 62.8, 35.5, 34.7, 31.7, 31.3, 29.8, 29.3, 28.9, 28.6, 28.4, 28.2, 28.1, 27.7, 27.6, 27.4, 26.8, 24.0. **HRMS** (ESI): m/z calculated for $C_{47}H_{62}NaO_5^+$ $[M + Na]^+$ 729.4489, found 729.4482. $[\alpha]_D^{25}$ = 3.1 (c = 0.62, CH_2Cl_2). **HPLC**: Chiralpak IB N-5 column, 95:5 hexanes/isopropanol, 1.0 mL/min; t_R = 5.1 min (major), 8.7 min (minor), 93:7 er.

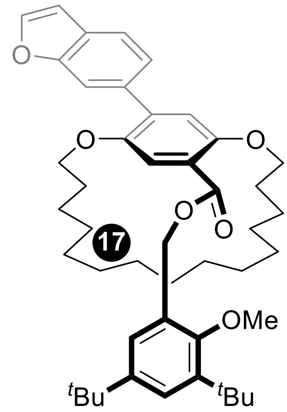

5je, 48.7 mg, 68% yield, Colorless oil. **¹H NMR** (600 MHz, $CDCl_3$) δ 7.75 (d, *J* = 1.4 Hz, 1H), 7.55 (d, *J* = 7.3 Hz, 1H), 7.48 (dd, *J* = 8.6, 1.8 Hz, 2H), 7.42 (d, *J* = 2.6 Hz, 1H), 7.36 (d, *J* = 2.5 Hz, 1H), 7.04 (s, 1H), 6.82 – 6.80 (m, 1H), 5.47 (d, *J* = 1.7 Hz, 2H), 4.26 (ddd, *J* = 11.0, 7.4, 4.8 Hz, 1H), 4.15 (qd, *J* = 6.4, 5.0 Hz, 1H), 4.02 – 4.09 (m, 1H), 3.99 (dt, *J* = 10.6, 5.3 Hz, 1H), 3.90 (s, 3H), 1.45 – 1.77 (m, 6H), 1.43 (s, 9H), 1.33 (s, 9H), 1.19 – 1.24 (m, 3H), 1.05 – 1.15 (m, 17H). **¹³C NMR** (150 MHz, $CDCl_3$) δ 166.1, 156.3, 154.6, 153.1, 149.2, 145.8, 145.5, 142.0, 137.2, 132.7, 128.9, 127.4, 126.2, 126.0, 124.6, 122.2, 118.3, 117.3, 110.9, 106.9, 69.1, 68.7, 62.9, 62.8, 35.5, 34.7, 31.7, 31.2, 29.5, 29.1, 28.9, 28.5, 28.46, 28.33, 28.3, 28.25, 28.0, 27.6, 27.2, 24.5, 24.45. **HRMS** (ESI): m/z calculated for $C_{46}H_{62}NaO_6^+$ [M + Na]$^+$ 733.4439, found 733.4433. $[\alpha]_D^{25}$ = 3.7 (c = 0.19, CH_2Cl_2). **HPLC**: Chiralpak IB N-5 column, 95:5 hexanes/isopropanol, 1.0 mL/min; t_R = 5.2 min (major), 8.9 min (minor), 90:10 er.

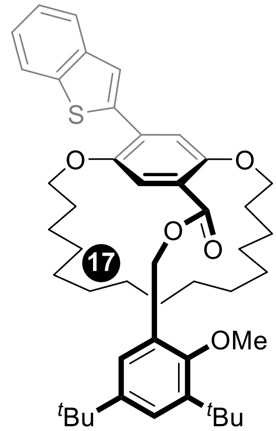

5ke

5ke, 51.6 mg, 71% yield, Colorless oil. **¹H NMR** (600 MHz, $CDCl_3$) δ 7.79 – 7.89 (m, 3H), 7.57 (s, 1H), 7.31 – 7.44 (m, 5H), 5.47 (s, 2H), 4.15 – 4.34 (m, 4H), 3.90 (s, 3H), 1.81 – 1.88 (m, 1H), 1.67 – 1.79 (m, 3H), 1.46 – 1.54 (m, 3H), 1.43 (s, 9H), 1.33 (s, 9H), 0.94 – 1.27 (m, 19H). **¹³C NMR** (150 MHz, $CDCl_3$) δ 165.8, 156.3, 152.9, 148.9, 145.8, 142.1, 140.4, 140.0, 139.1, 128.8, 128.7, 125.9, 124.7, 124.6, 124.4, 124.0, 123.9, 122.0, 116.9, 116.2, 69.3, 68.8, 63.1, 62.8, 35.5, 34.7, 31.7, 31.2, 29.3, 29.1, 28.8, 28.4, 28.37, 28.2, 28.19, 28.15, 27.9, 27.4, 27.2, 24.6, 24.4. **HRMS** (ESI): m/z calculated for $C_{46}H_{62}NaO_5S^+$ [M + Na]$^+$ 749.4210, found 749.4217. $[\alpha]_D^{25}$ = 9.1 (c = 0.1, CH_2Cl_2). **HPLC**: Chiralpak IB N-5 column, 95:5 hexanes/isopropanol, 1.0 mL/min; t_R = 5.9 min (major), 7.0 min (minor), 90:10 er.

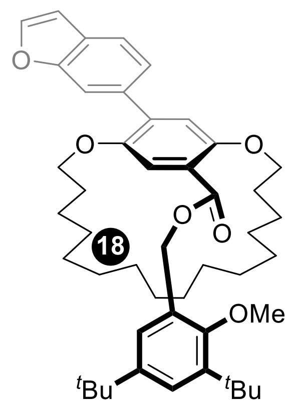

5le, 50.8 mg, 69% yield, Colorless oil. **^{1}H NMR** (600 MHz, $CDCl_3$) δ 7.75 (d, *J* = 1.7 Hz, 1H), 7.66 (d, *J* = 2.3 Hz, 1H), 7.52 – 7.56 (m, 2H), 7.49 (dd, *J* = 8.6, 1.8 Hz, 1H), 7.42 (d, *J* = 2.5 Hz, 1H), 7.36 (d, *J* = 2.6 Hz, 1H), 7.02 (s, 1H), 6.81 (dd, *J* = 2.4, 0.9 Hz, 1H), 5.47 (d, *J* = 2.1 Hz, 2H), 4.22 (ddd, *J* = 10.7, 7.6, 5.3 Hz, 1H), 4.14 (dt, *J* = 11.2, 5.8 Hz, 1H), 3.98 – 4.03 (m, 2H), 3.90 (s, 3H), 1.60 – 1.82 (m, 3H), 1.50 – 1.57 (m, 2H), 1.43 (s, 9H), 1.33 (s, 9H), 1.15 – 1.26 (m, 8H), 1.13 (s, 13H). **^{13}C NMR** (150 MHz, $CDCl_3$) δ 166.1, 156.3, 154.6, 152.8, 149.2, 145.8, 145.5, 142.0, 136.9, 132.7, 128.9, 127.4, 126.2, 125.9, 124.6, 122.2, 119.6, 117.9, 116.6, 110.9, 106.9, 68.8, 68.3, 62.9, 62.7, 35.5, 34.7, 31.7, 31.2, 29.4, 29.35, 29.0, 28.6, 28.58, 28.2, 28.14, 28.1, 27.9, 27.8, 27.4, 26.8, 24.3, 24.1. **HRMS** (ESI): m/z calculated for $C_{47}H_{64}NaO_6^+$ [M + Na]$^+$ 747.4595, found 747.4599. **HPLC**: Chiralpak IB N-5 column, 95:5 hexanes/isopropanol, 1.0 mL/min; t_R = 4.9 min (major), 8.2 min (minor), 76:24 er.

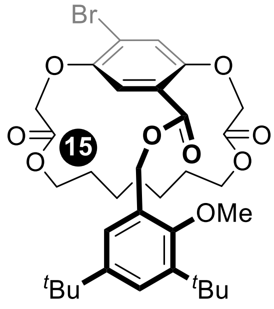

5me, 17.2 mg, 26% yield, Colorless oil. **^{1}H NMR** (600 MHz, $CDCl_3$) δ 7.33 – 7.35 (m, 2H), 7.32 (s, 1H), 7.11 (s, 1H), 5.39 – 5.47 (m, 2H), 4.69 – 4.82 (m, 4H), 4.31 – 4.35 (m, 1H), 4.22 – 4.26 (m, 1H), 3.95 – 3.99 (m, 1H), 3.89 – 3.93 (m, 1H), 3.86 (s, 3H), 1.43 –1.51 (m, 4H), 1.41 (s, 9H), 1.30 (s, 9H), 0.93 – 1.08 (m, 4H), 0.83 – 0.91 (m, 2H). **^{13}C NMR** (150 MHz, $CDCl_3$) δ 189.5, 168.9, 168.6, 165.1, 156.4, 152.1, 148.7, 142.2, 128.4, 126.0, 124.9, 120.4, 118.6, 114.9, 66.4, 66.3, 65.2, 63.6, 62.8, 35.5, 34.6, 31.6, 31.2, 28.1, 28.06, 27.9, 25.90, 25.89. **HRMS** (ESI): m/z calculated for $C_{33}H_{43}BrNaO_9^+$ [M + Na]$^+$ 685.1983, found 685.1980. $[\alpha]_D^{25}$ = 3.0 (c = 0.1, CH_2Cl_2). **HPLC**: Chiralpak IB N-5 column, 95:5 hexanes/isopropanol, 1.0 mL/min; t_R = 19.7 min (major), 22.5 min (minor), 6.5:93.5 er.

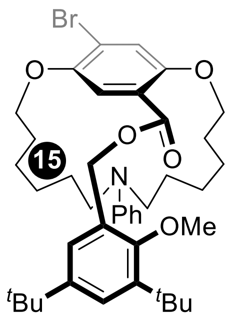

5ne, 11.8 mg, 16% yield, Colorless oil. **^{1}H NMR** (600 MHz, $CDCl_3$) δ 7.47 (s, 1H), 7.34 (s, 2H), 7.28 (s, 1H), 7.15 (dd, *J* = 8.8, 7.2 Hz, 2H), 6.59 (t, *J* = 7.1 Hz, 1H), 6.51 (d, *J* = 8.3 Hz, 2H), 5.42 (s, 2H), 4.34 (dddd, *J* = 18.3, 11.1, 6.6, 3.9 Hz, 2H), 4.25 (ddd, *J* = 12.0, 7.6, 4.1 Hz, 1H), 4.16 (ddd, *J* = 11.9, 7.7, 4.3 Hz, 1H), 3.84 (s, 3H), 3.07 (ddt, *J* = 14.9, 10.5, 5.6 Hz, 2H), 2.84 (dddd, *J* = 18.9, 15.0, 9.8, 5.8 Hz, 2H), 1.64 – 1.74 (m, 3H), 1.41 (s, 10H), 1.33 – 1.38 (m, 3H), 1.30 (s, 11H), 1.15 – 1.24 (m, 7H). **^{13}C NMR** (150 MHz, $CDCl_3$) δ 165.7, 156.3, 152.6, 148.7, 147.8, 145.9, 142.2, 129.3, 128.5, 125.9, 124.8, 121.2, 121.1, 118.4, 117.6, 115.3, 111.8, 69.7, 69.6, 63.4, 62.7, 51.1, 51.0, 35.5, 31.7, 31.2, 27.7, 27.6, 26.5, 25.7, 25.5, 25.1, 24.9. **HRMS** (ESI): m/z calculated for $C_{41}H_{56}BrNNaO_5^+$ $[M + Na]^+$ 744.3234, found 744.3245. $[\alpha]_D^{25}$ = 2.7 (c = 0.15, CH_2Cl_2). **HPLC**: Chiralpak IB N-5 column, 95:5 hexanes/isopropanol, 1.0 mL/min; t_R = 6.6 min (minor), 8.9 min (major), 29:71 er.

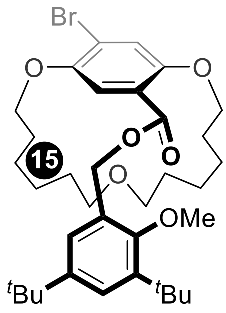

5oe, 43.3 mg, 67% yield, Colorless oil. **^{1}H NMR** (600 MHz, $CDCl_3$) δ 7.44 (s, 1H), 7.35 (s, 2H), 7.25 (s, 1H), 5.42 (s, 2H), 4.14 – 4.26 (m, 4H), 3.85 (s, 3H), 3.11 (s, 4H), 1.63 – 1.73 (m, 4H), 1.44 – 1.51 (m, 1H), 1.41 (s, 9H), 1,35 – 1.38 (m, 3H), 1.31 (s, 9H), 1.12 – 1.26 (m, 8H). **^{13}C NMR** (150 MHz, $CDCl_3$) δ 165.6, 156.3, 153.1, 149.0, 145.9, 142.1, 128.6, 125.9, 124.8, 121.5, 121.2, 118.5, 117.9, 70.1, 69.6, 69.5, 63.3, 62.7, 35.5, 34.7, 31.7, 31.2, 29.1, 27.9, 27.7, 24.3, 24.1, 24.04, 23.95. **HRMS** (ESI): m/z calculated for $C_{35}H_{51}BrNaO_6^+$ $[M + Na]^+$ 669.2761, found 669.2755. $[\alpha]_D^{25}$ = 60.0 (c = 0.05, CH_2Cl_2). **HPLC**: Chiralpak IB N-5 column, 95:5 hexanes/isopropanol, 1.0 mL/min; t_R = 5.2 min (major), 7.3 min (minor), 94:6 er.

2.2 X-Ray report

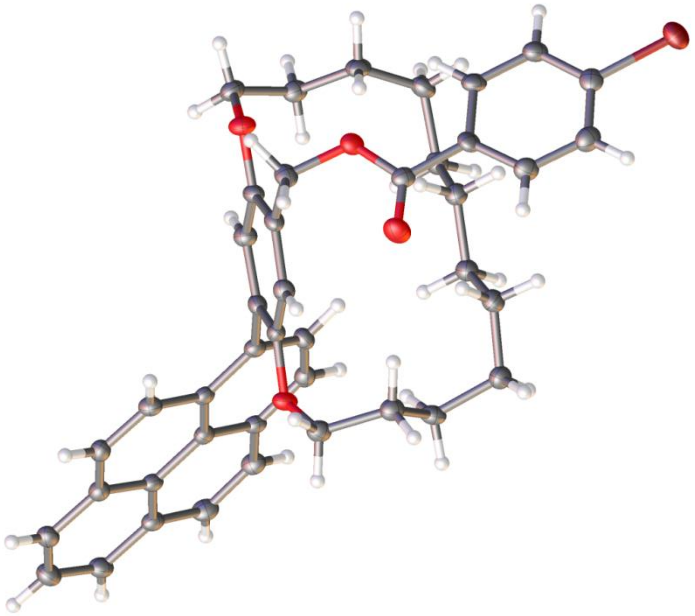

Supplementary Figure 4. X-ray structure of **(R_p)-10** (CCDC No. 2256859).

Supplementary table 2: Single crystal data of (R_p)-10

Identification code	**10**
Empirical formula	$C_{42}H_{41}BrO_4$
Formula weight	689.66
Temperature/K	100k
Crystal system	monoclinic
Space group	$P2_1$
a/Å	9.9792(2)
b/Å	7.75970(10)
c/Å	22.2109(5)
α/°	90
β/°	101.912(2)
γ/°	90
Volume/Å^3	1682.88(6)
Z	2
ρ_{calc}g/cm^3	1.361
μ/mm^{-1}	1.999
F(000)	720.0
Crystal size/mm^3	0.2 × 0.05 × 0.05
Radiation	Cu Kα (λ = 1.54184)
2Θ range for data collection/	8.136 to 154.424
Index ranges	$-12 \leq h \leq 12$, $-9 \leq k \leq 9$, $-27 \leq l \leq 25$

Reflections collected	19605
Independent reflections	6780 [R_{int} = 0.0550, R_{sigma} = 0.0559]
Data/restraints/parameters	6780/1/424
Goodness-of-fit on F^2	1.110
Final R indexes [I>=2σ (I)]	R_1 = 0.0452, wR_2 = 0.1159
Final R indexes [all data]	R_1 = 0.0484, wR_2 = 0.1180
Largest diff. peak/hole / e $Å^{-3}$	0.35/-0.53
Flack parameter	-0.016(19)

2.3 Copies of NMR spectra

Supplementary Figure 5. ^{1}H NMR and ^{13}C NMR spectrum of compound of **1b**

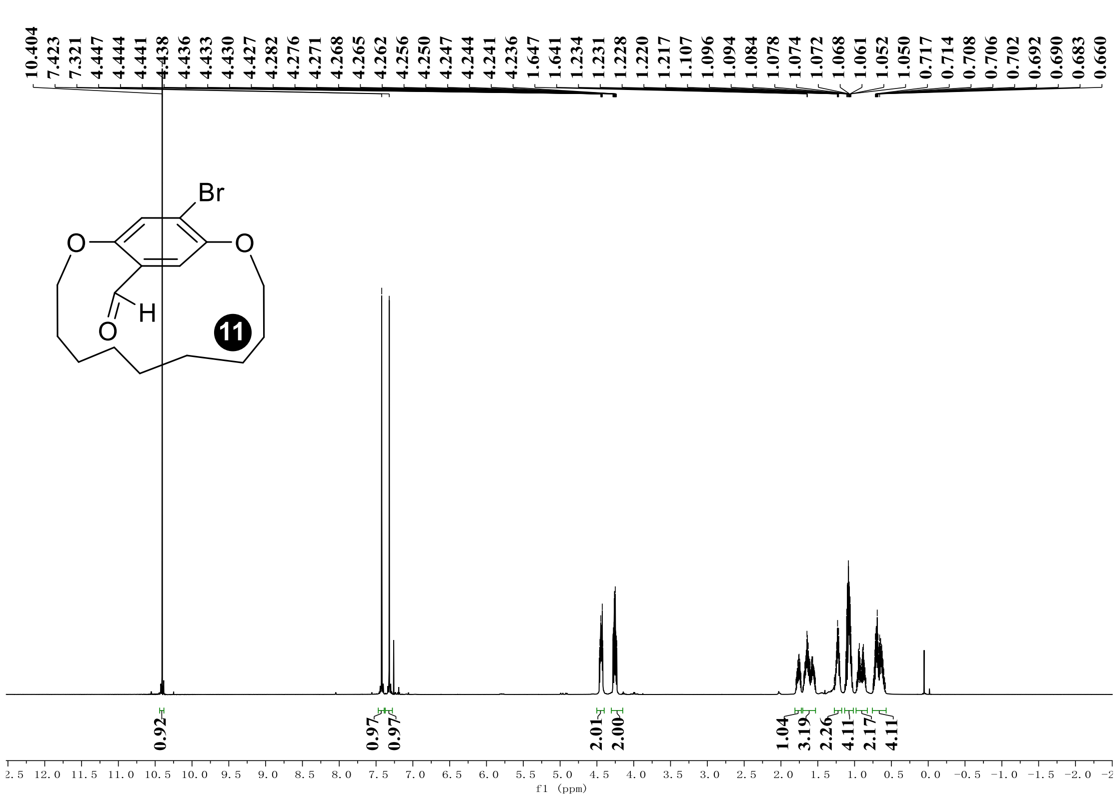

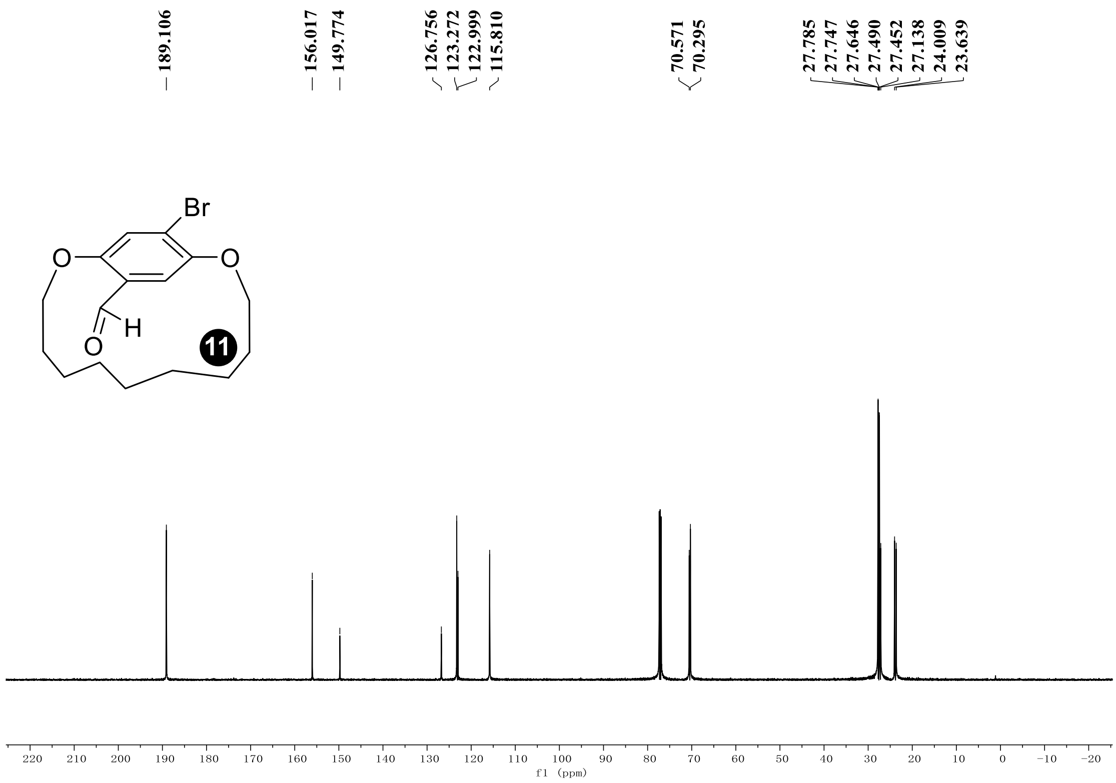

Supplementary Figure 6. ^{1}H NMR and ^{13}C NMR spectrum of compound of **1c**

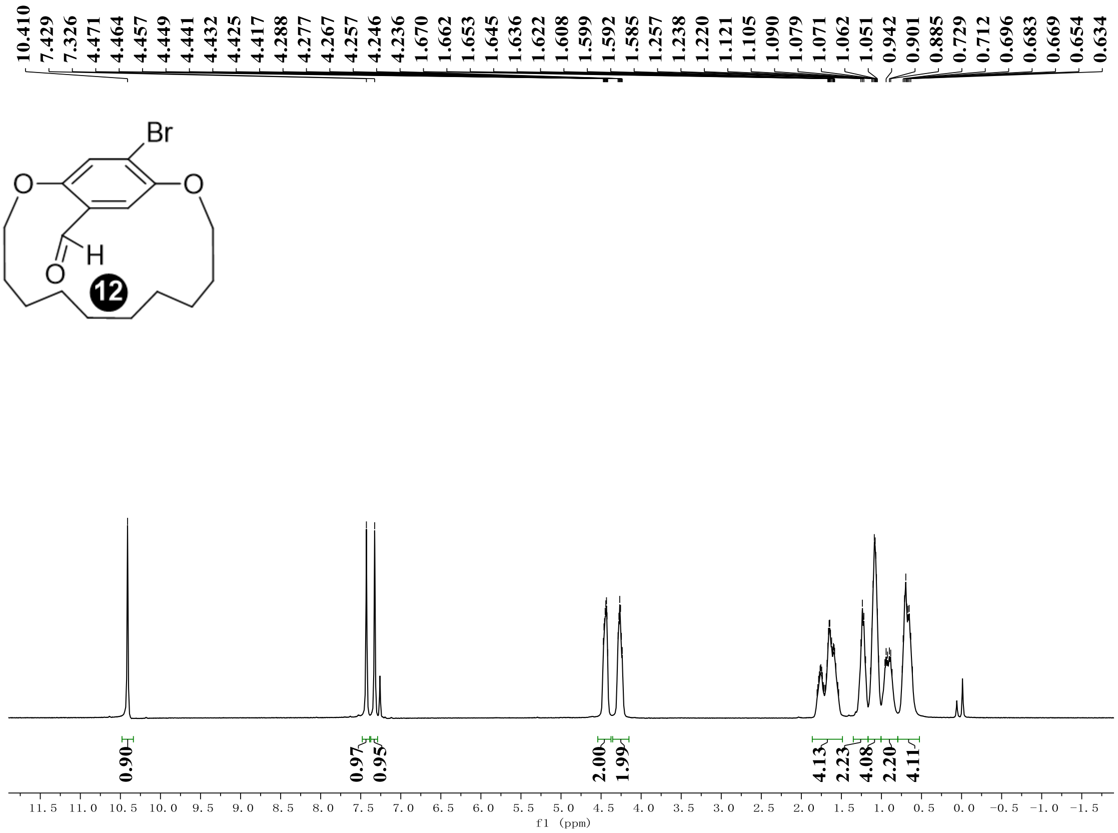

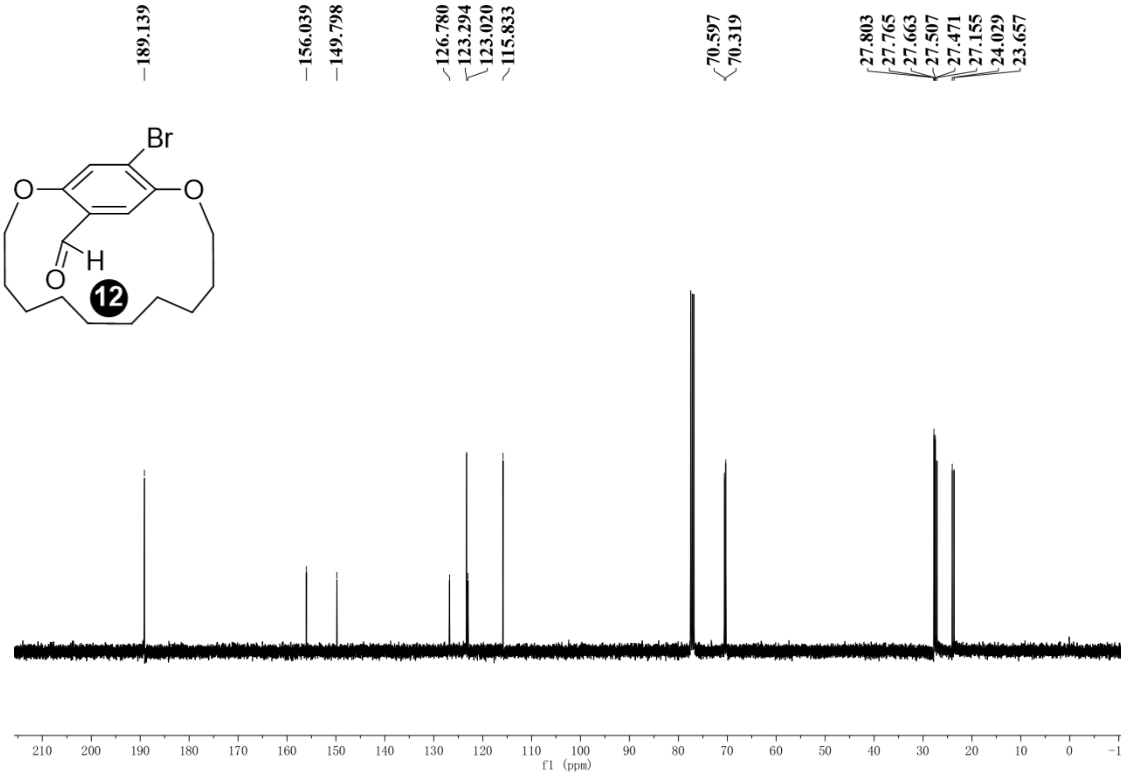

Supplementary Figure 7. ^{1}H NMR and ^{13}C NMR spectrum of compound of **1d**

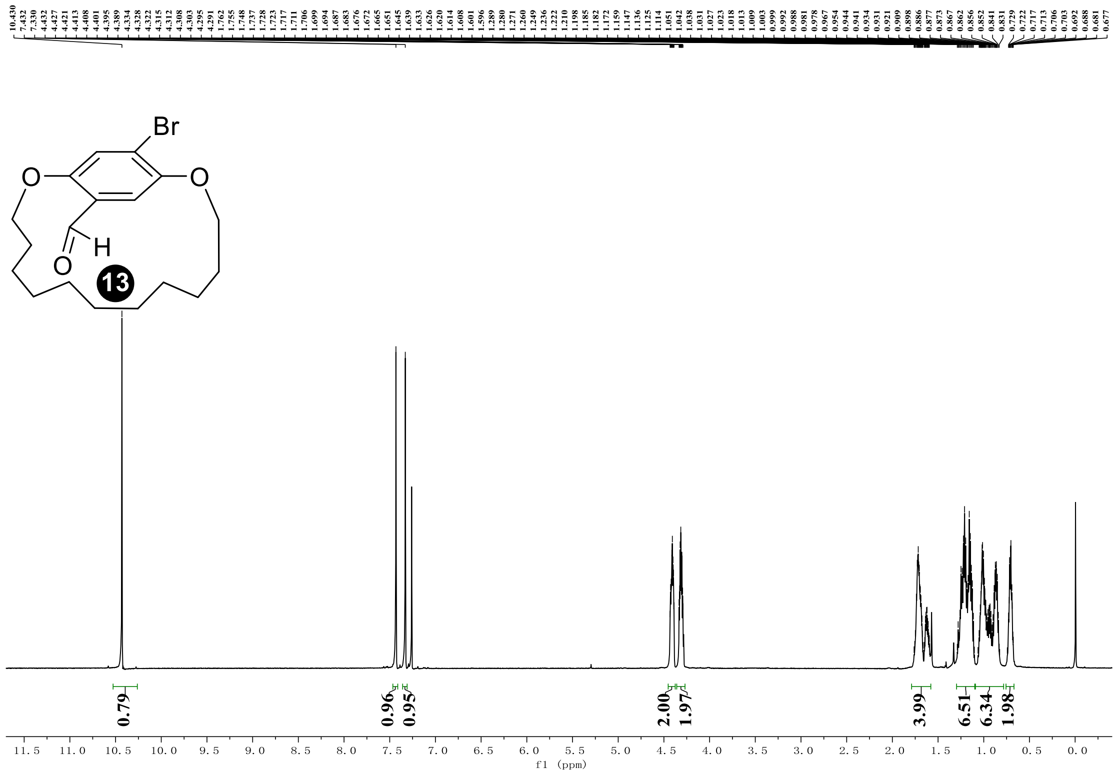

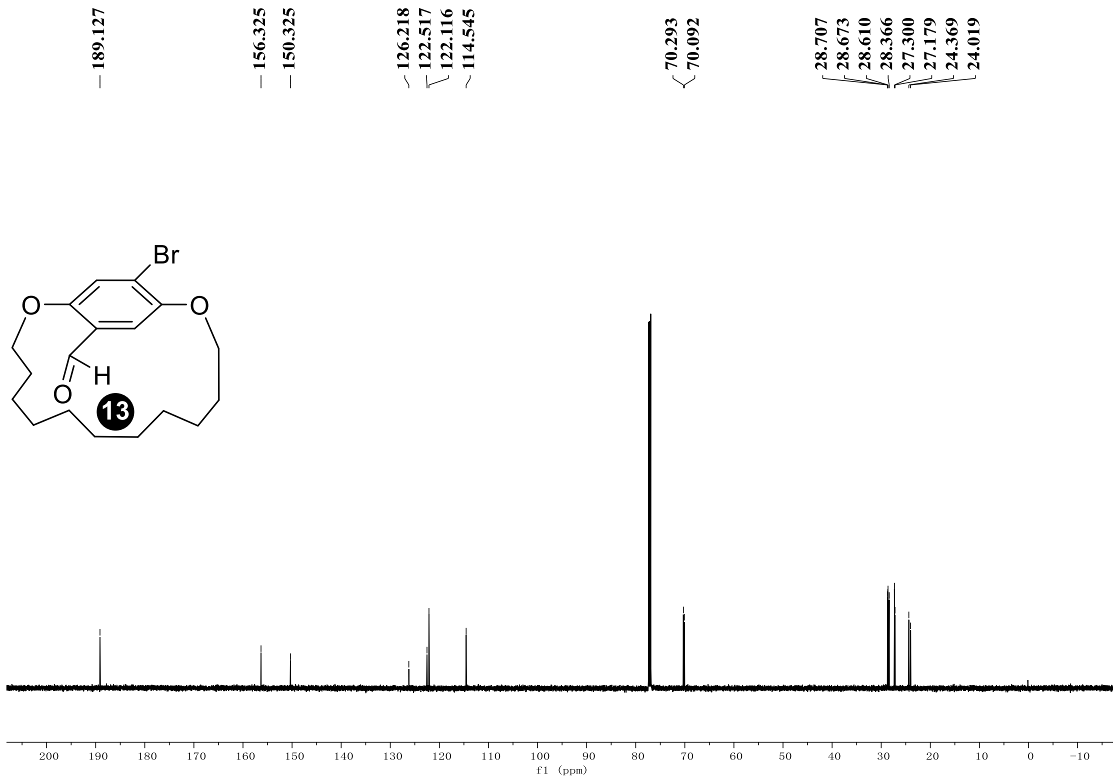

Supplementary Figure 8. ^{1}H NMR and ^{13}C NMR spectrum of compound of **4a**

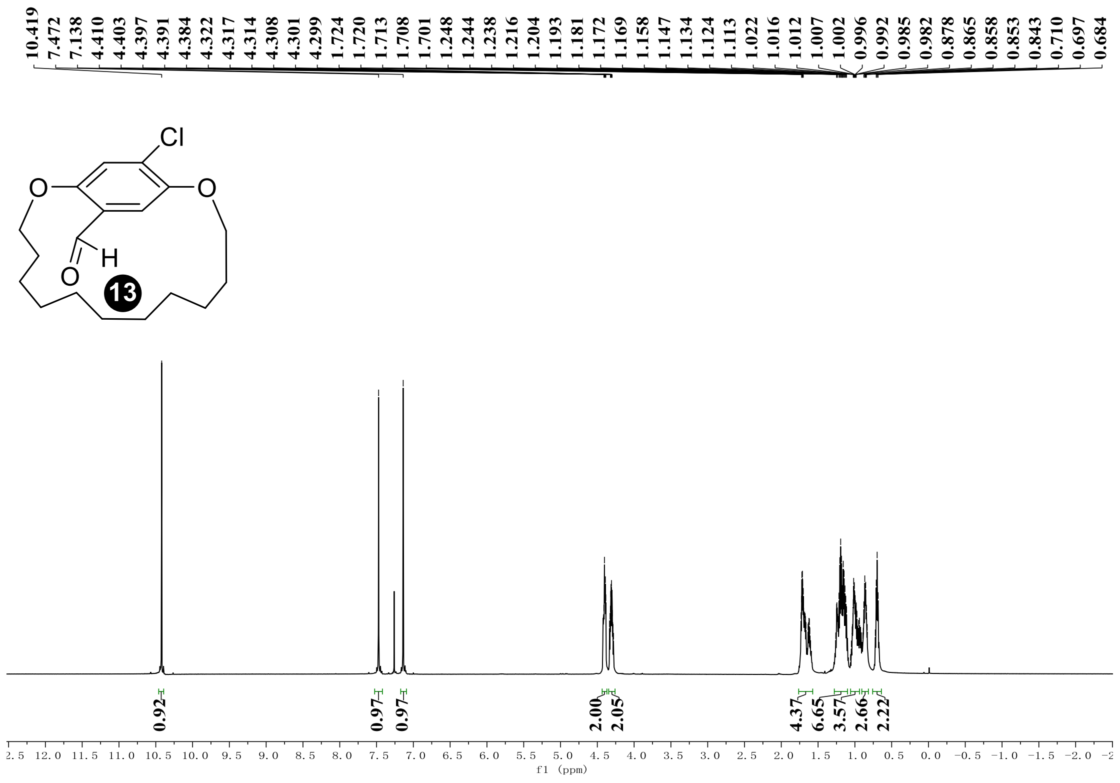

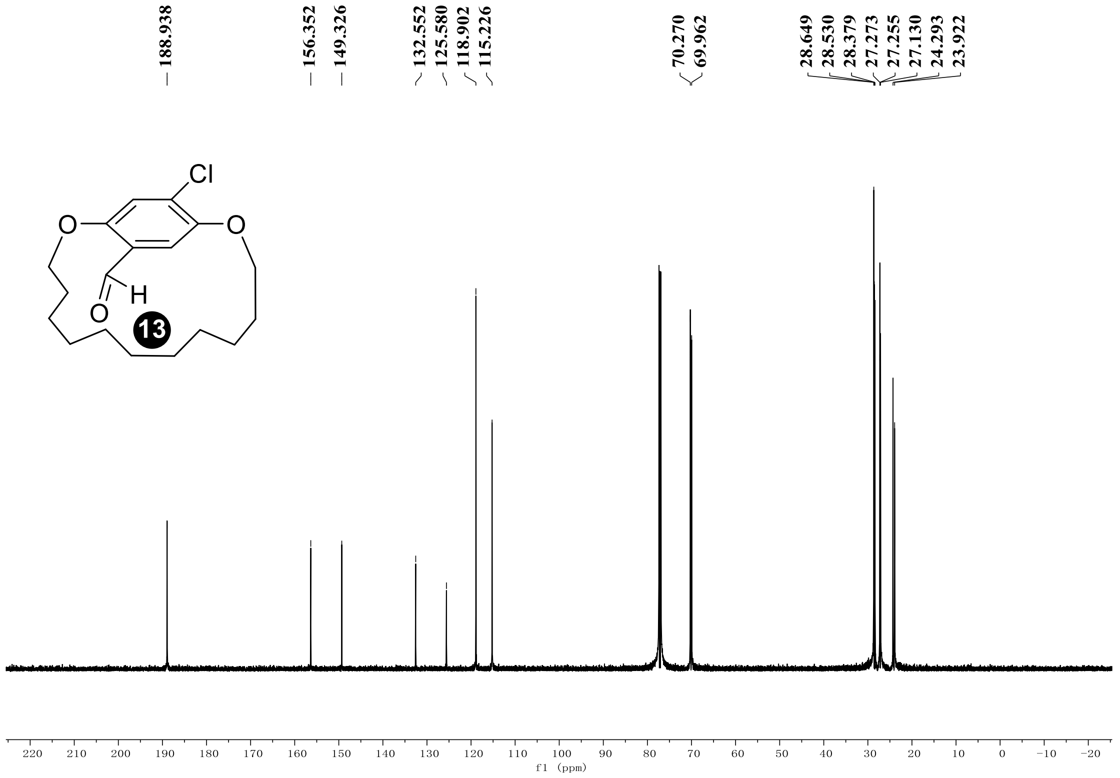

Supplementary Figure 9. ^{1}H NMR and ^{13}C NMR spectrum of compound of **1a**

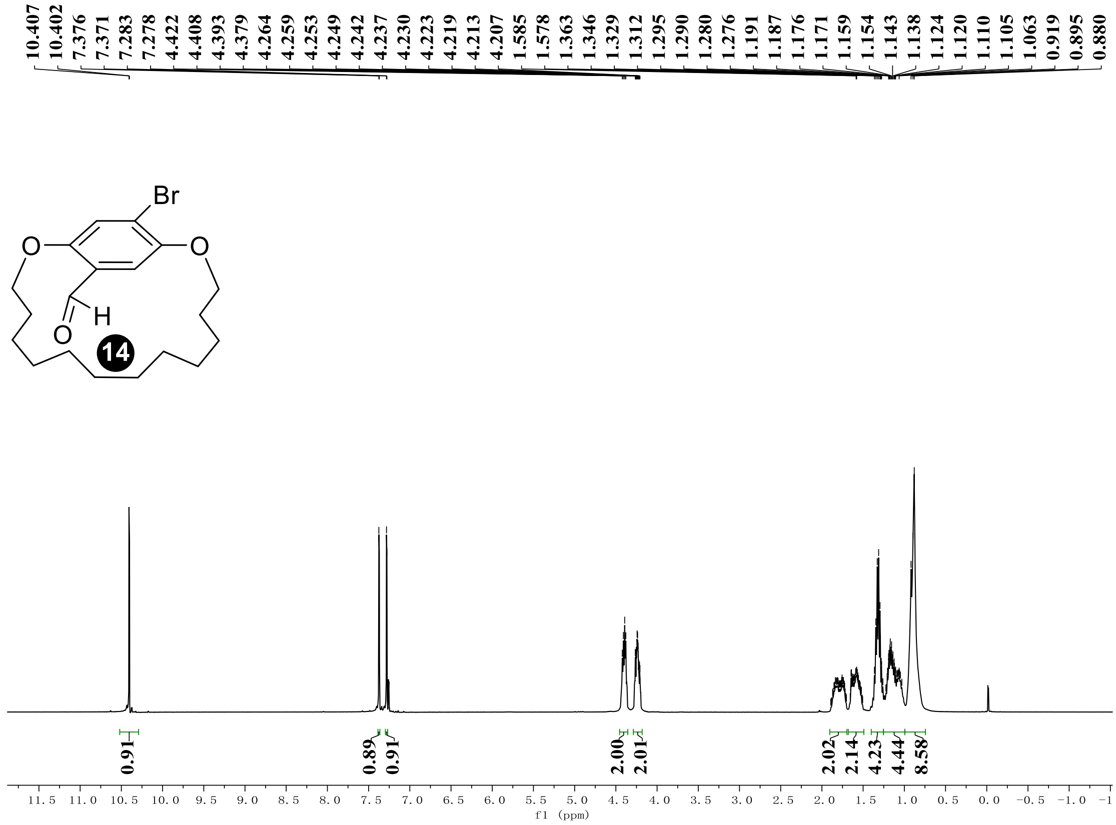

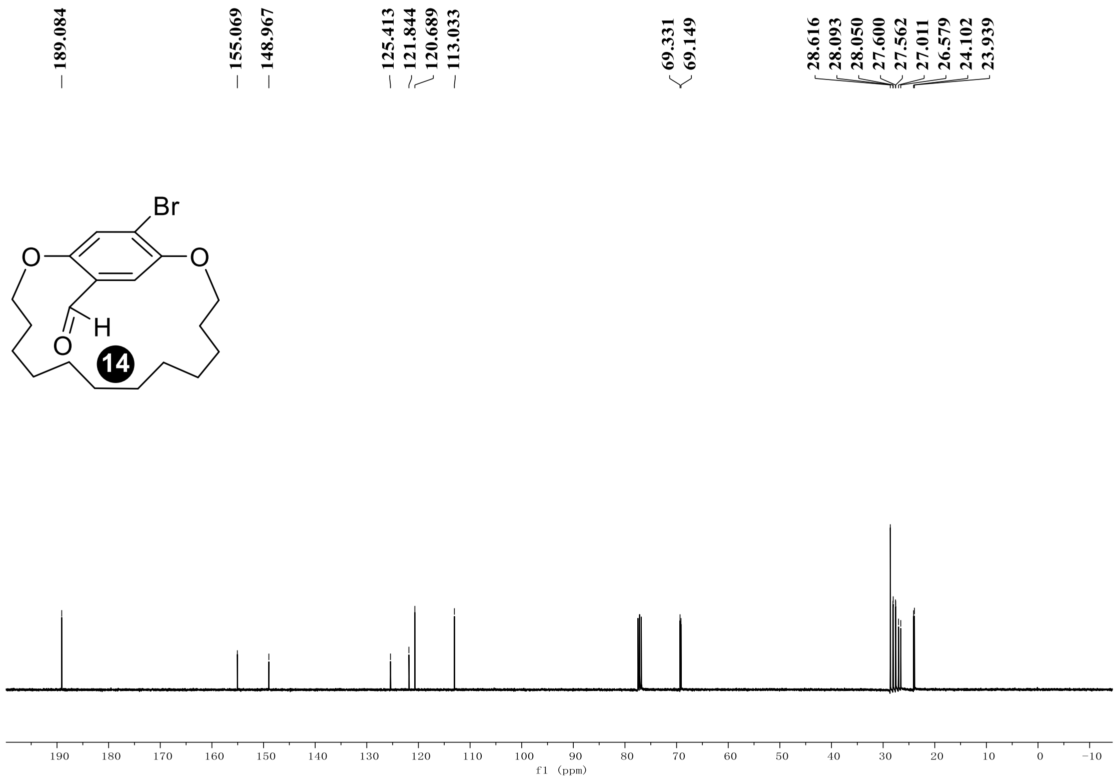

Supplementary Figure 10. ^{1}H NMR and ^{13}C NMR spectrum of compound of **1h**

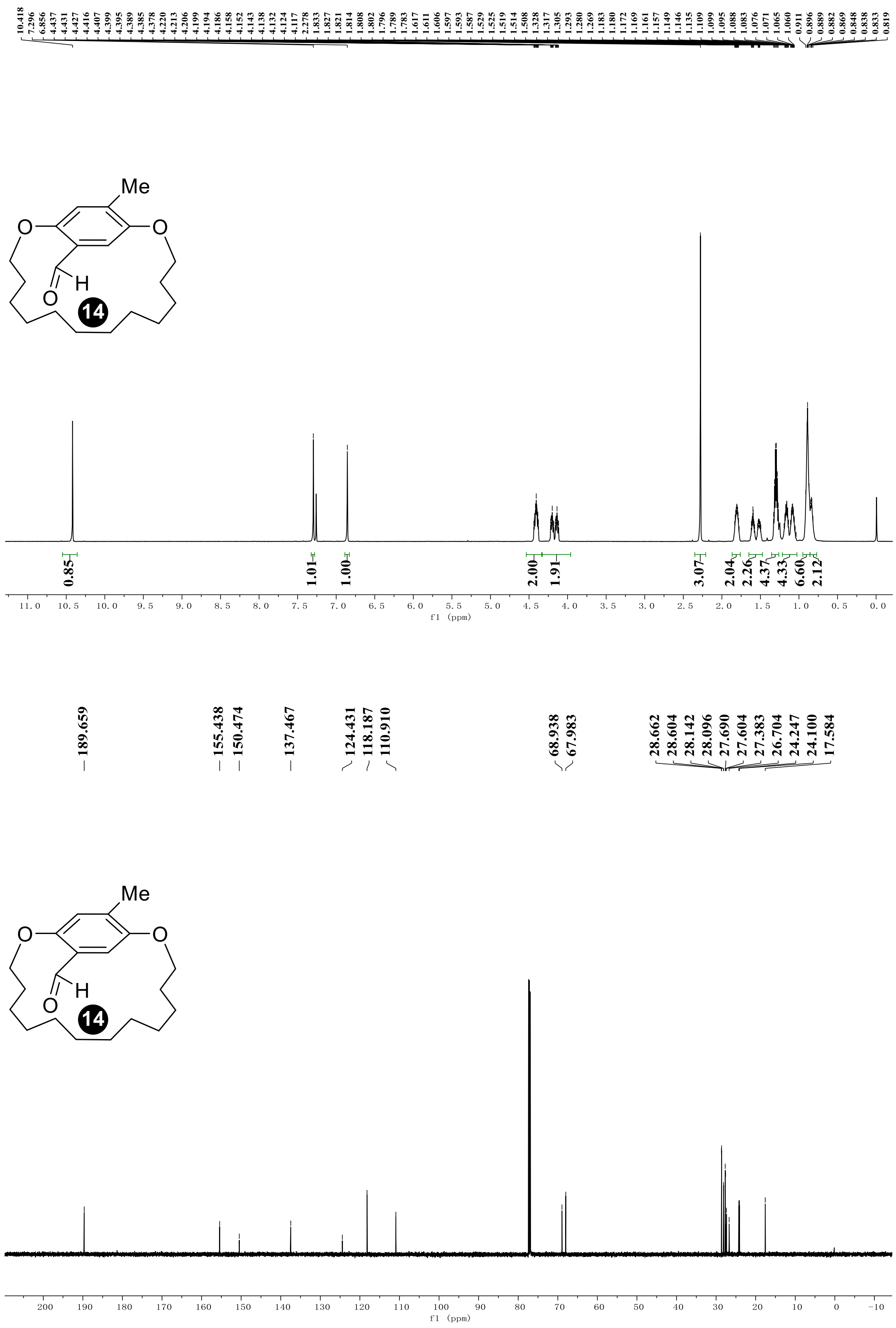

Supplementary Figure 11. ^{1}**H NMR and** ^{13}**C NMR** spectrum of compound of **1k**

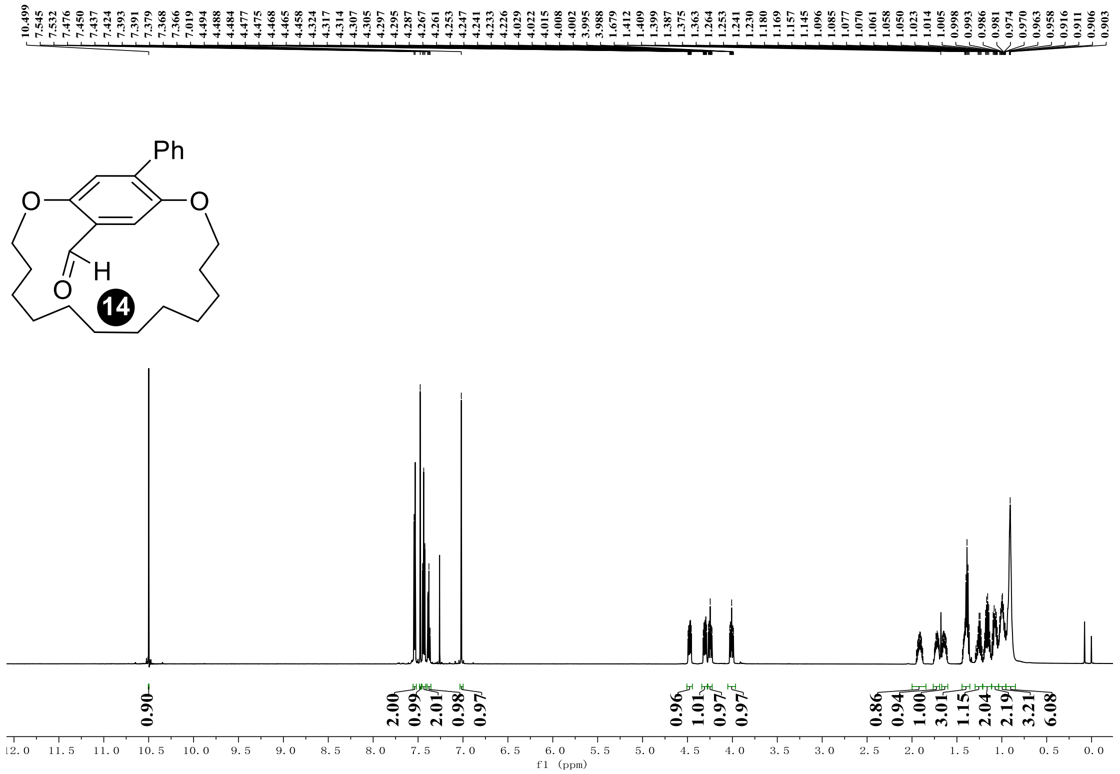

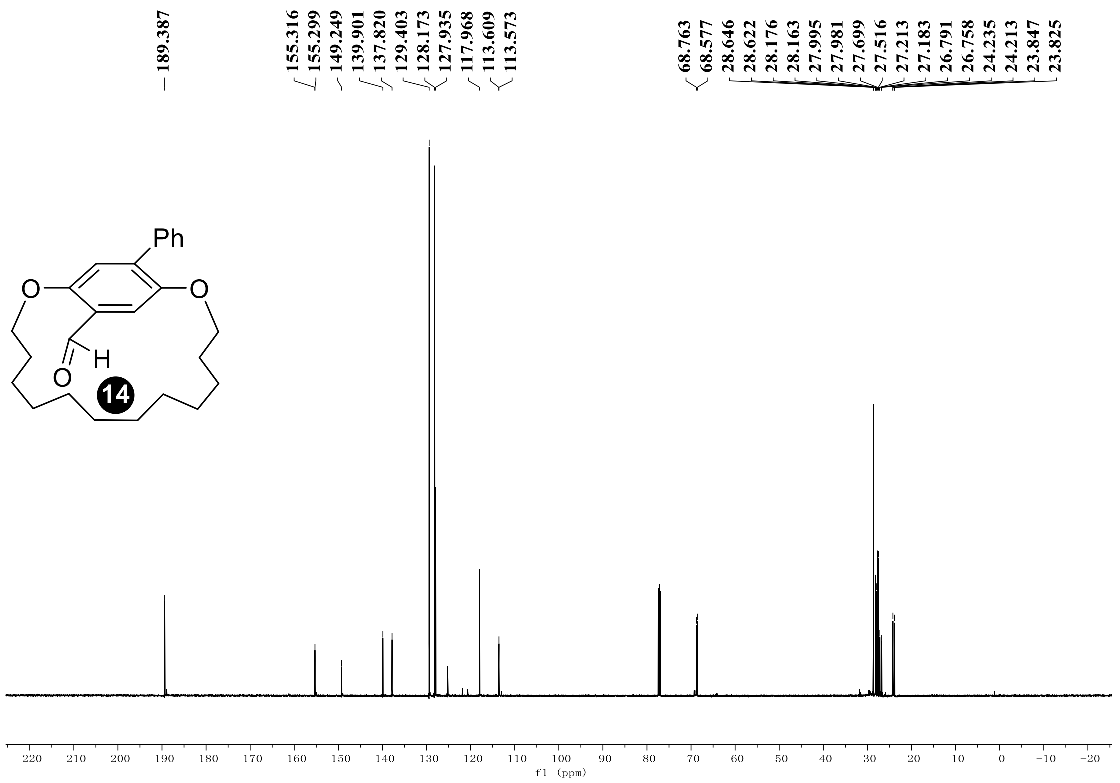

Supplementary Figure 12. **^{1}H NMR and ^{13}C NMR** spectrum of compound of **4c**

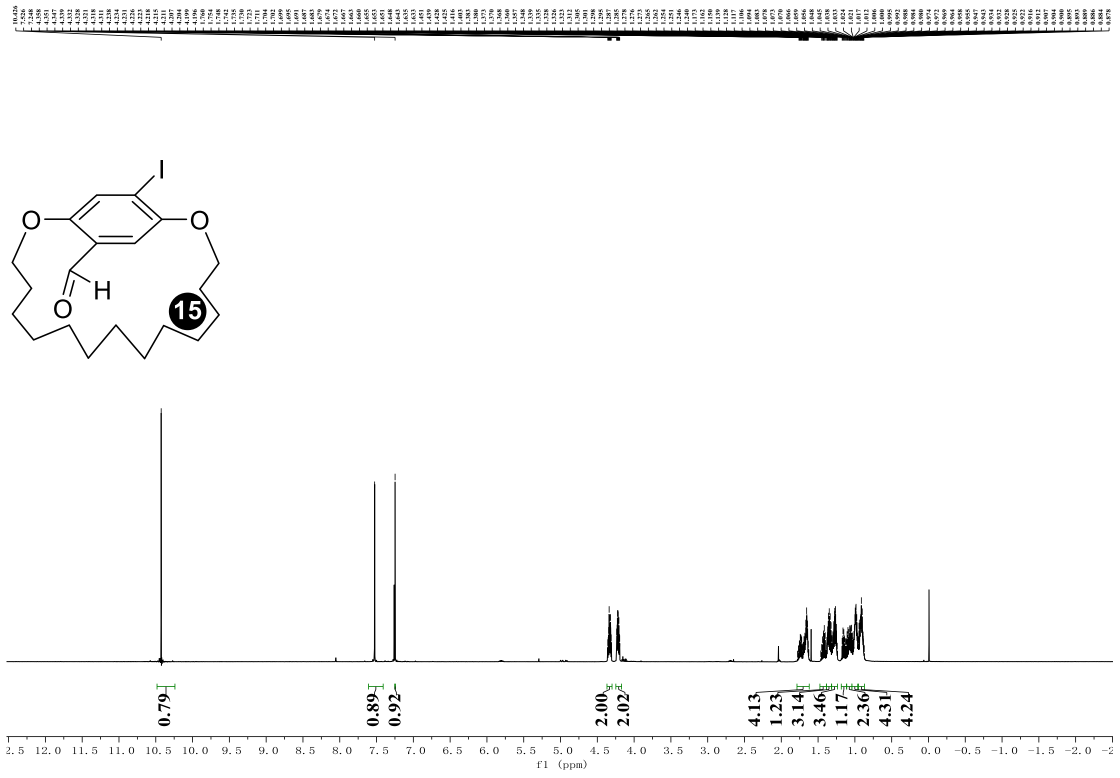

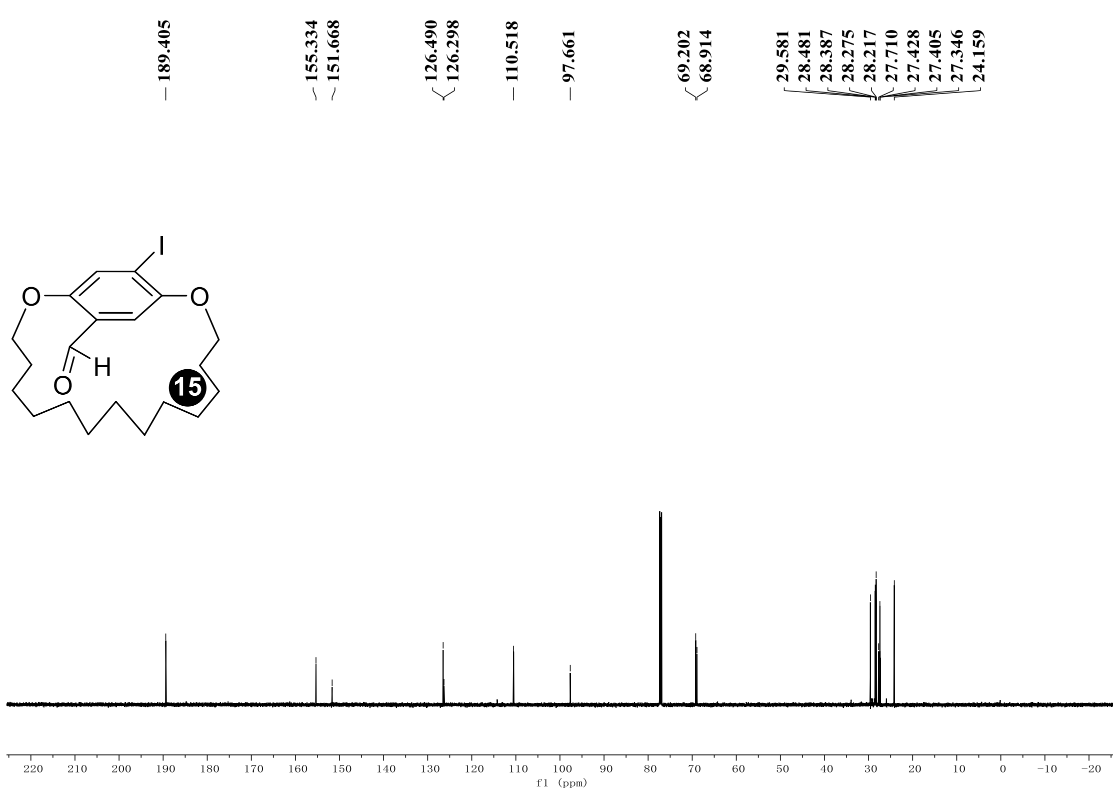

Supplementary Figure 13. ¹H NMR and ¹³C NMR spectrum of compound of **4d**

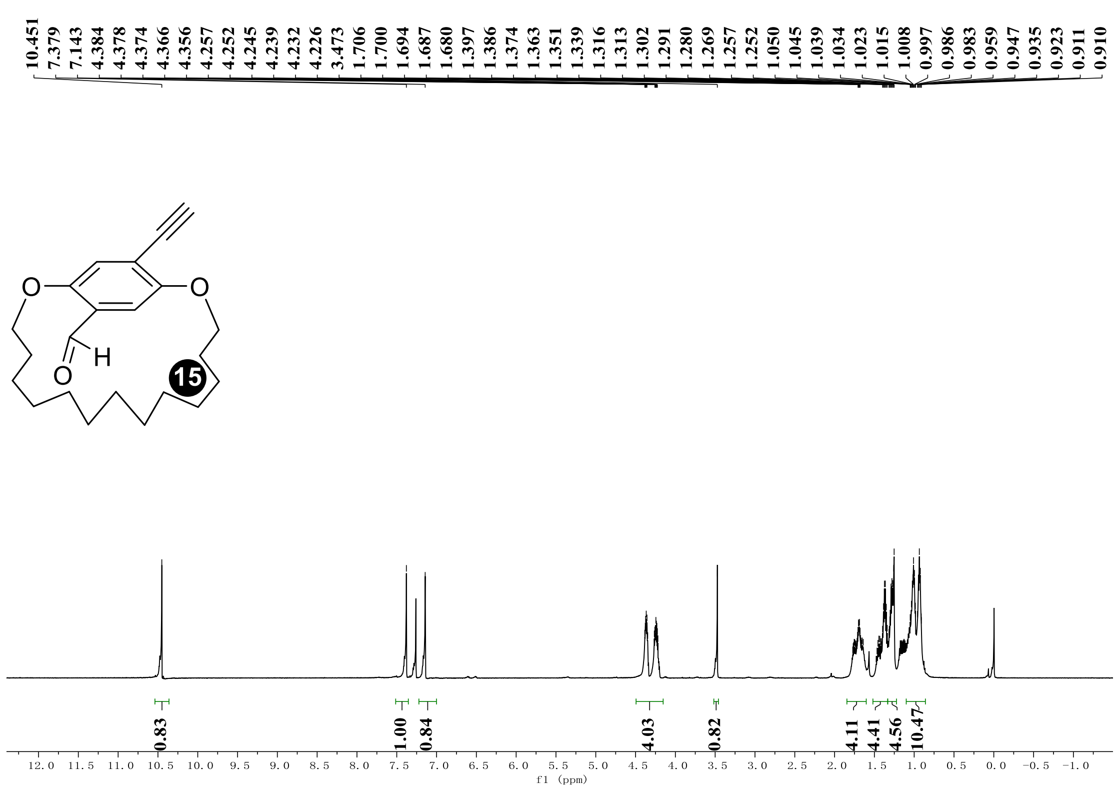

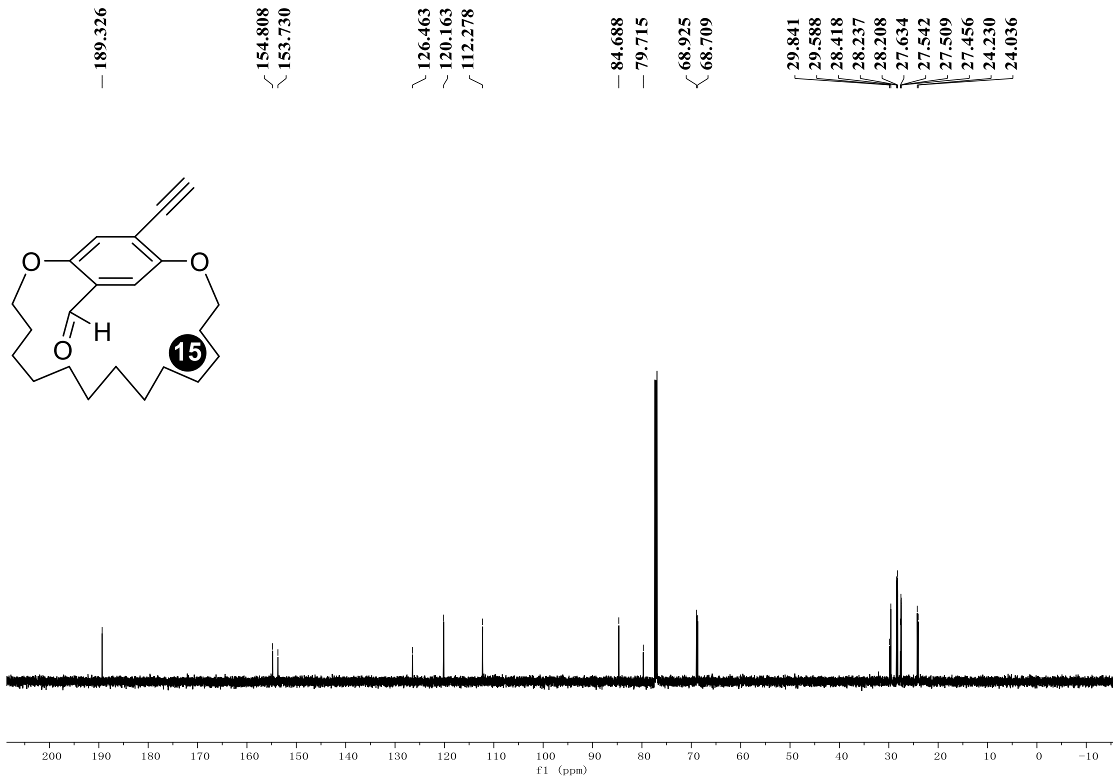

Supplementary Figure 14. ^{1}H NMR and ^{13}C NMR spectrum of compound of **4h**

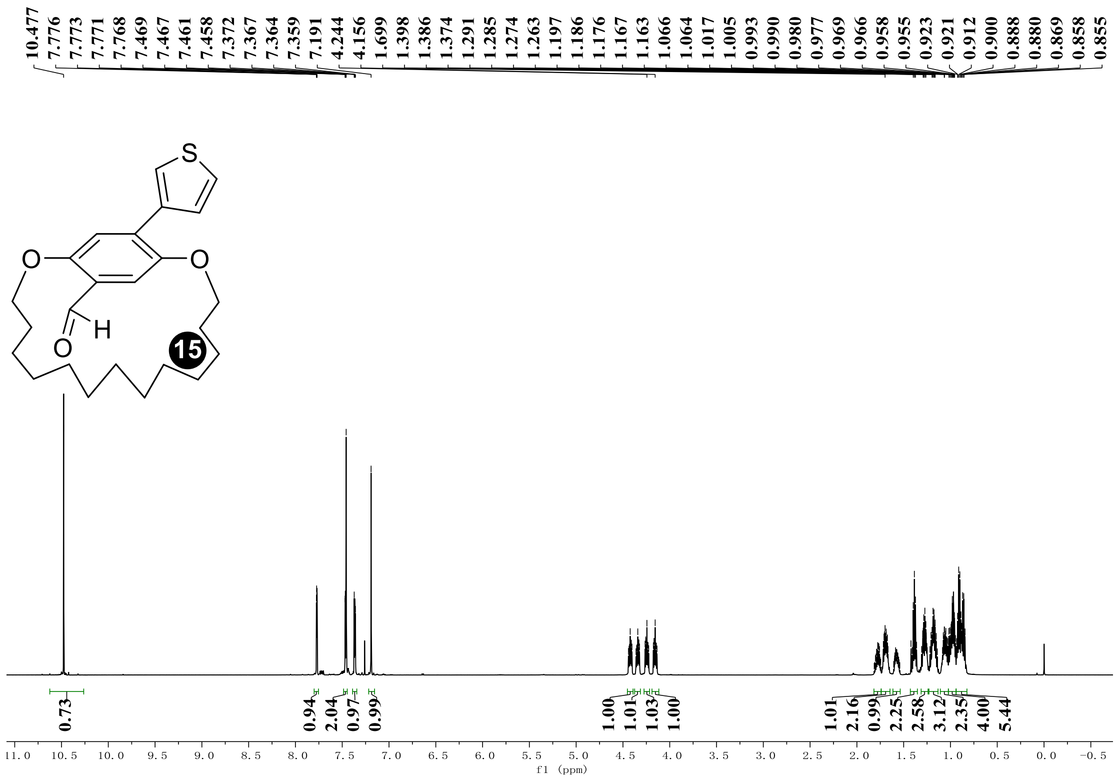

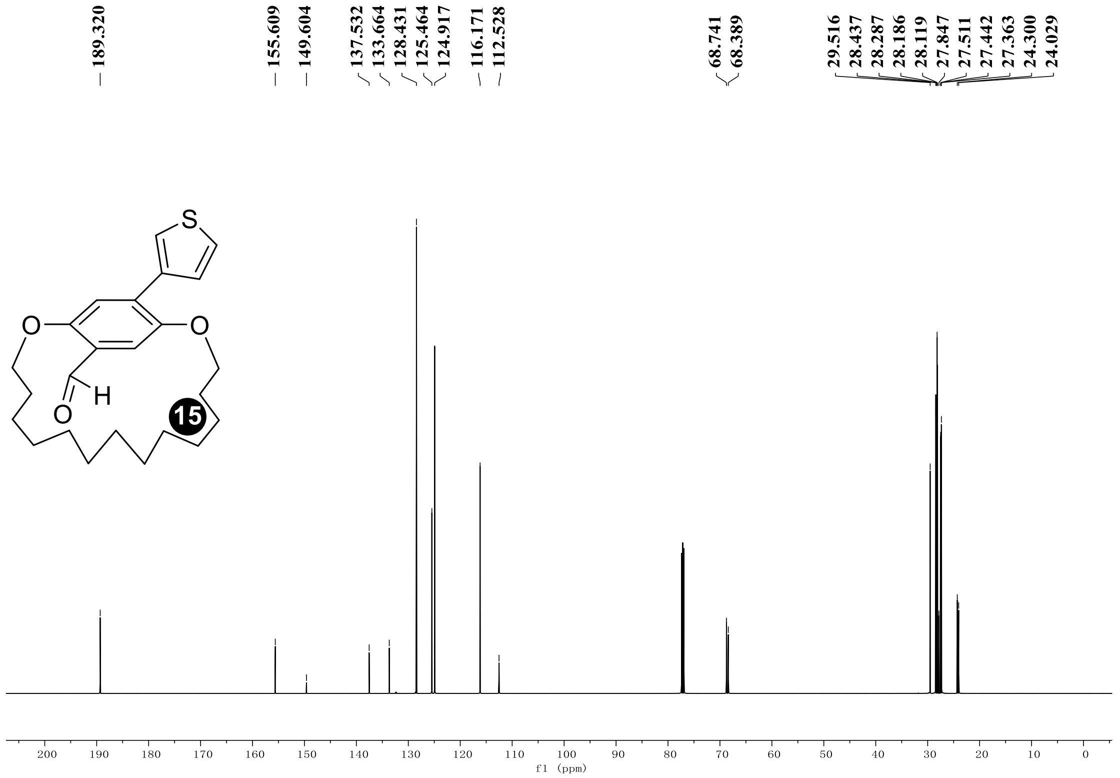

Supplementary Figure 15. ¹H NMR and ¹³C NMR spectrum of compound of **4m**

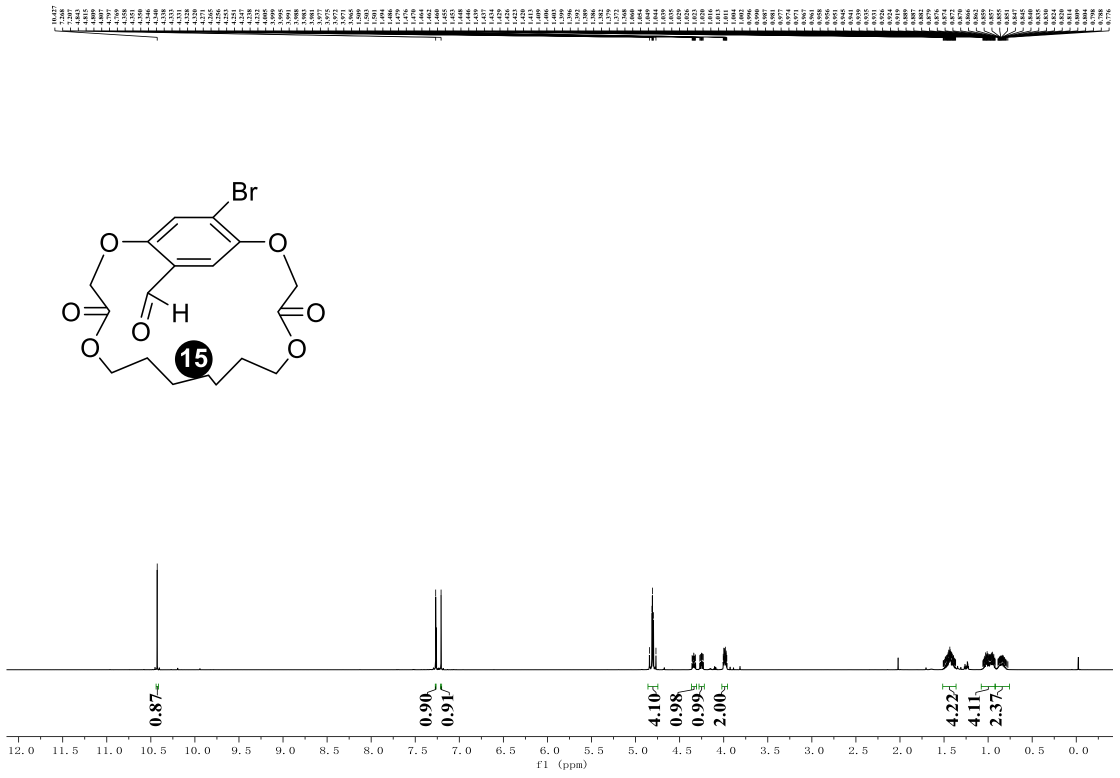

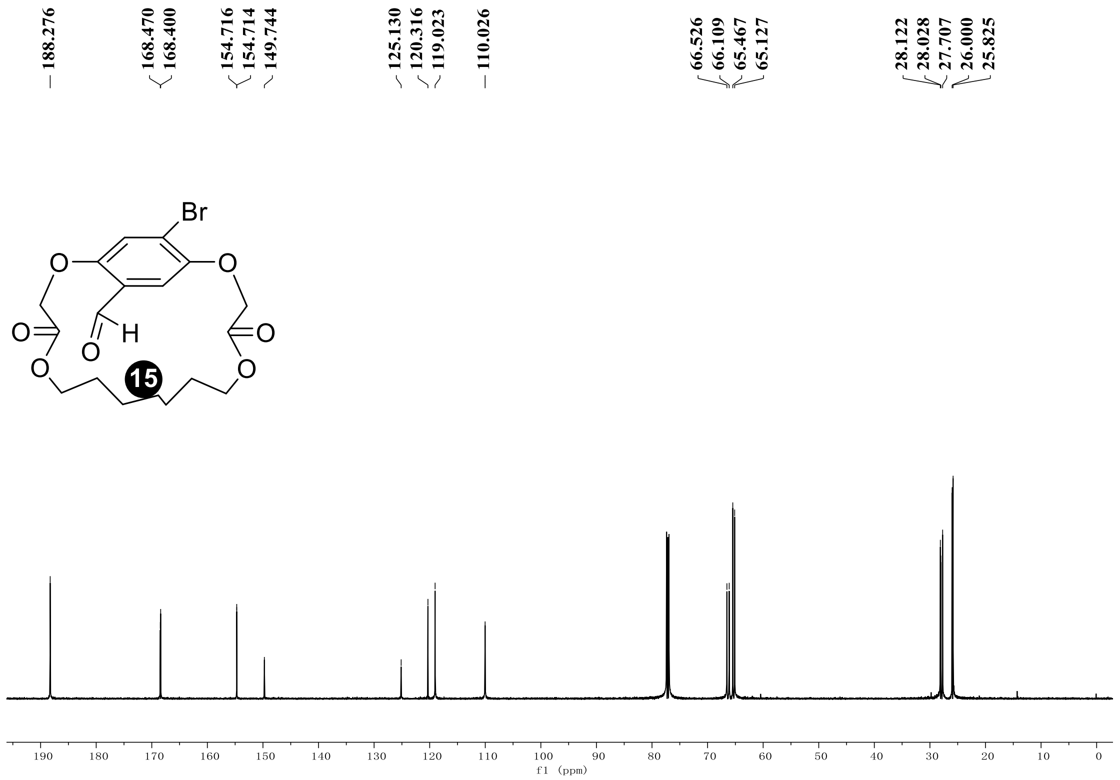

Supplementary Figure 16. ^{1}H NMR and ^{13}C NMR spectrum of compound of **4n**

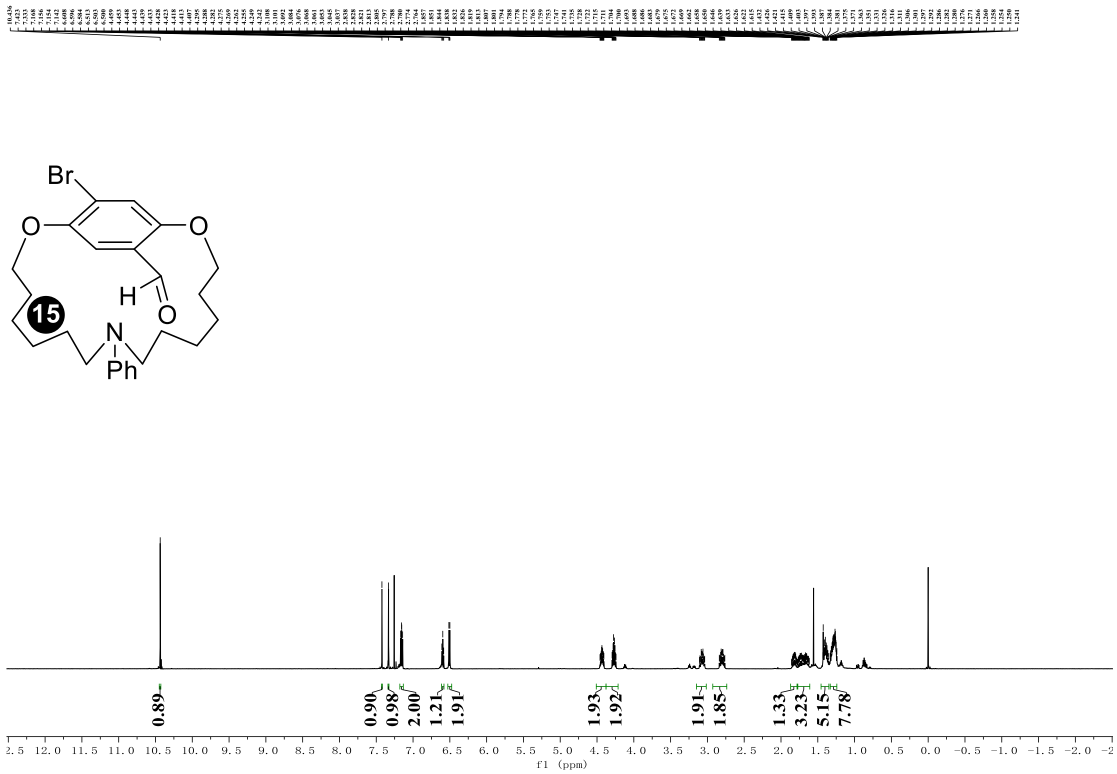

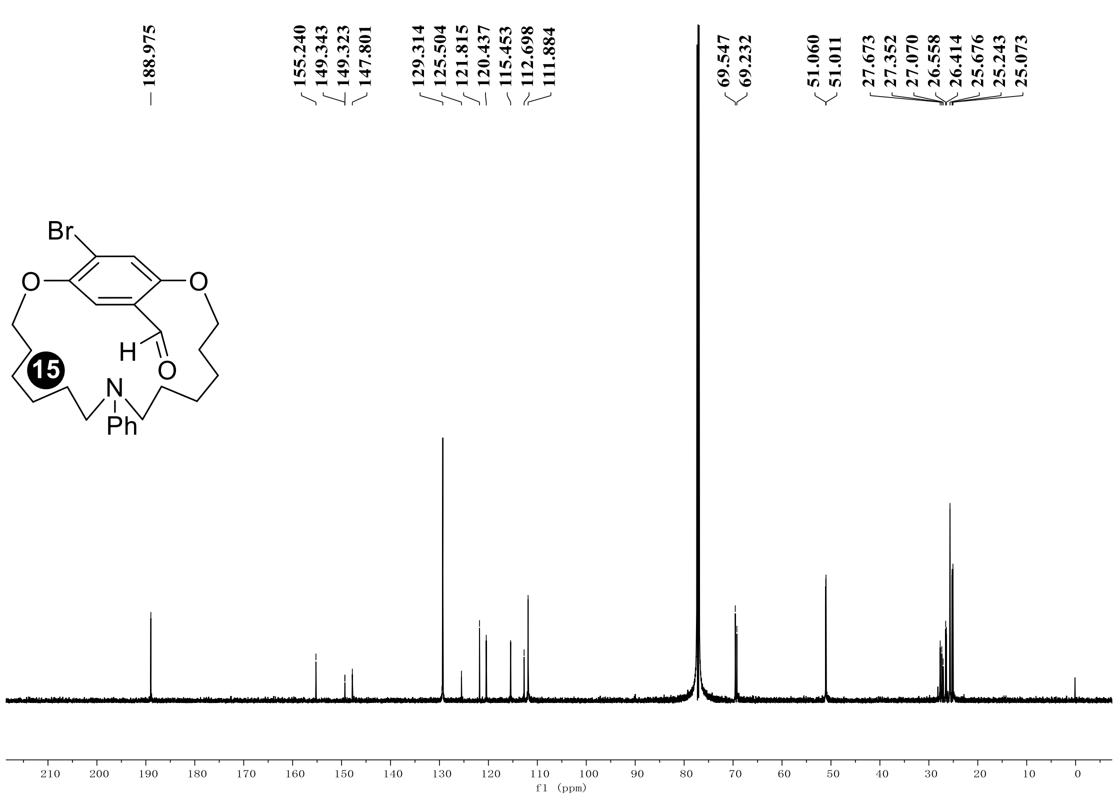

Supplementary Figure 17. ^{1}H NMR and ^{13}C NMR spectrum of compound of **4o**

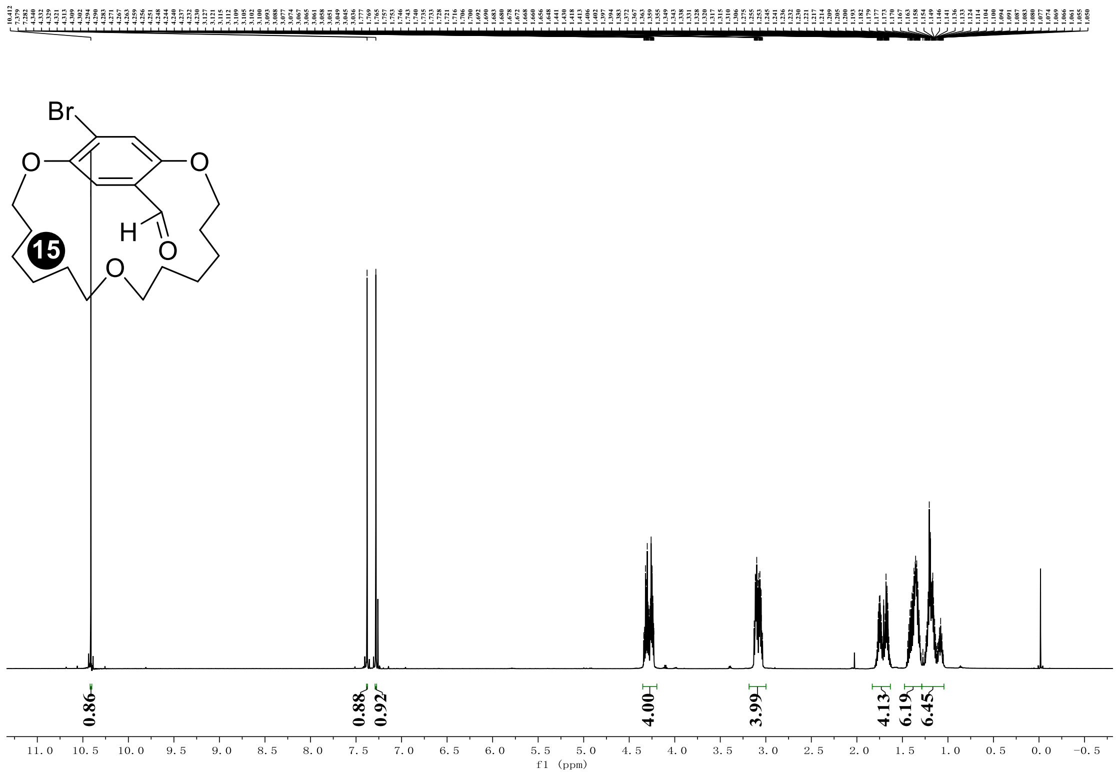

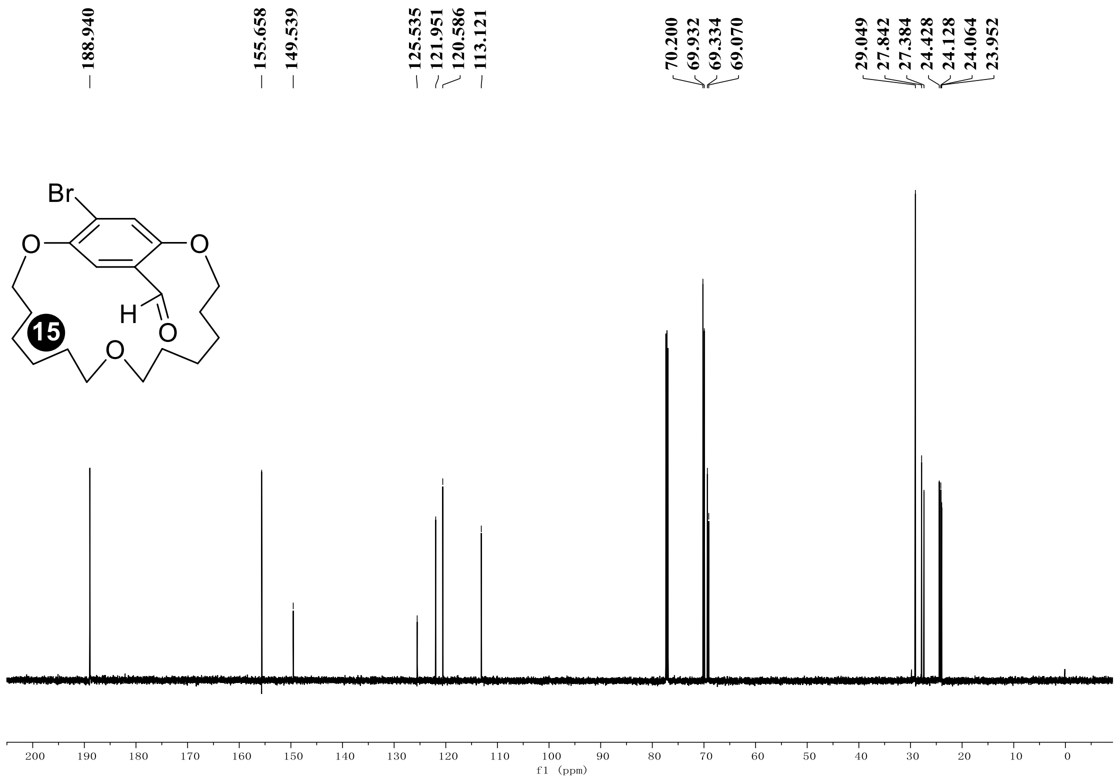

Supplementary Figure 18. ^{1}**H NMR and** ^{13}**C NMR** spectrum of compound of **11**

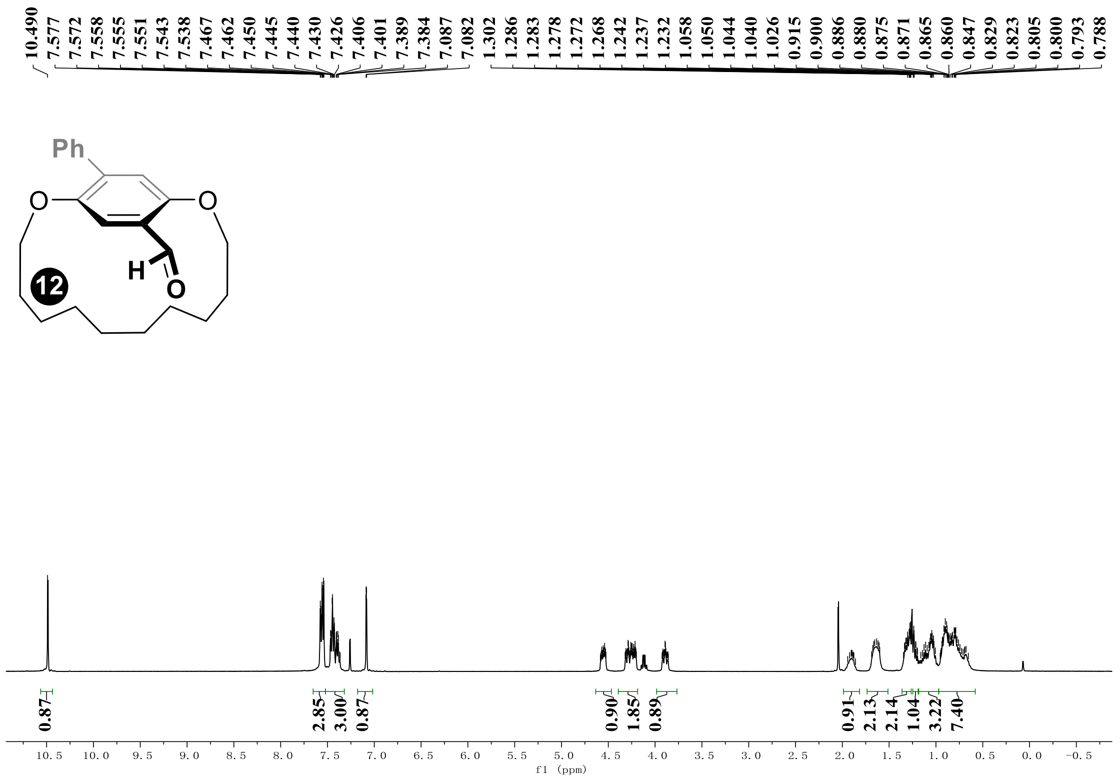

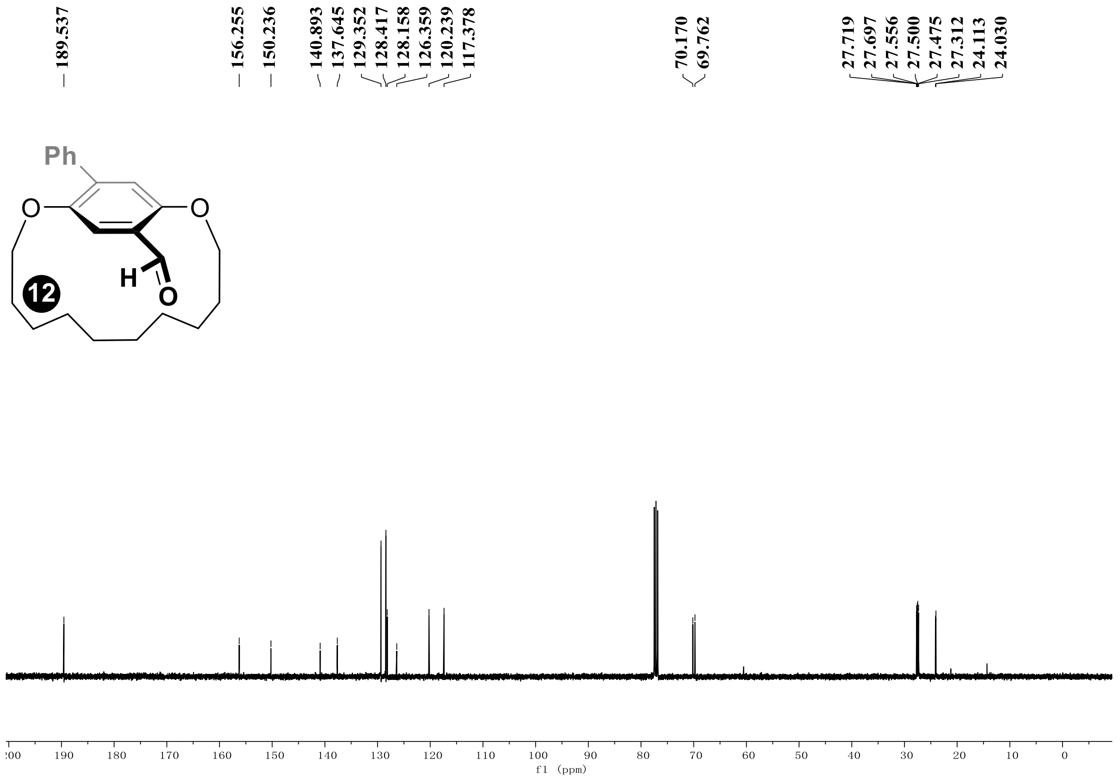

Supplementary Figure 19. ^{1}H NMR and ^{13}C NMR spectrum of compound of **4l**

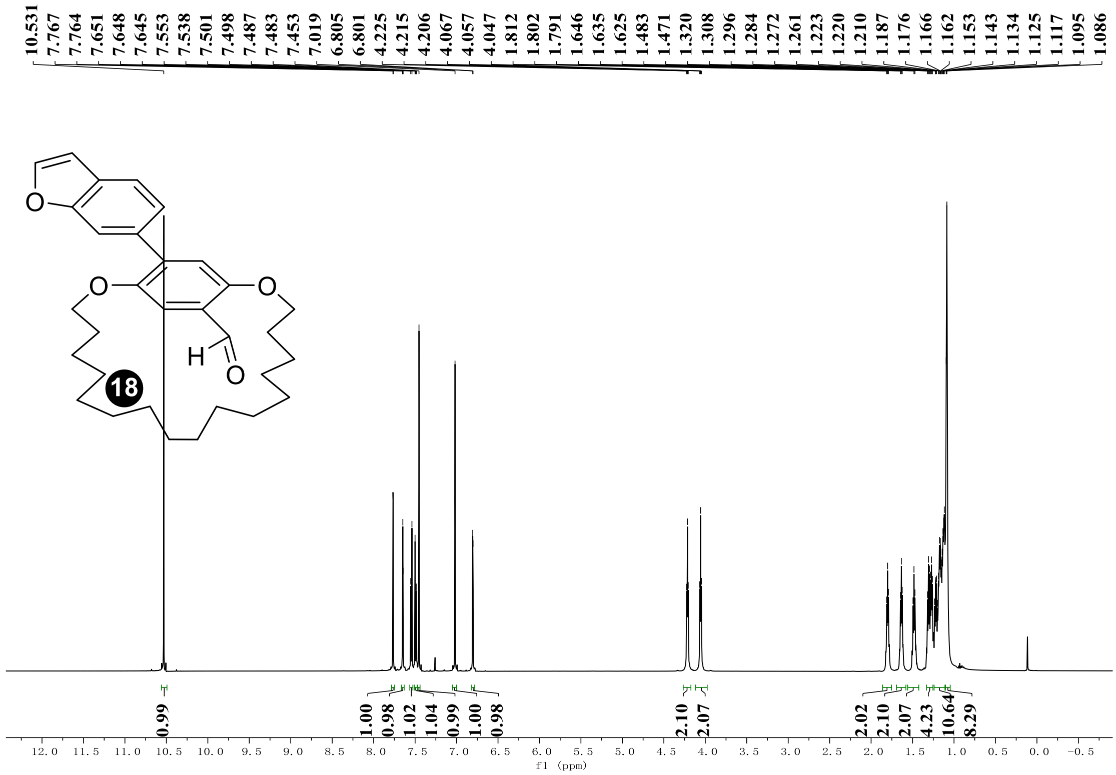

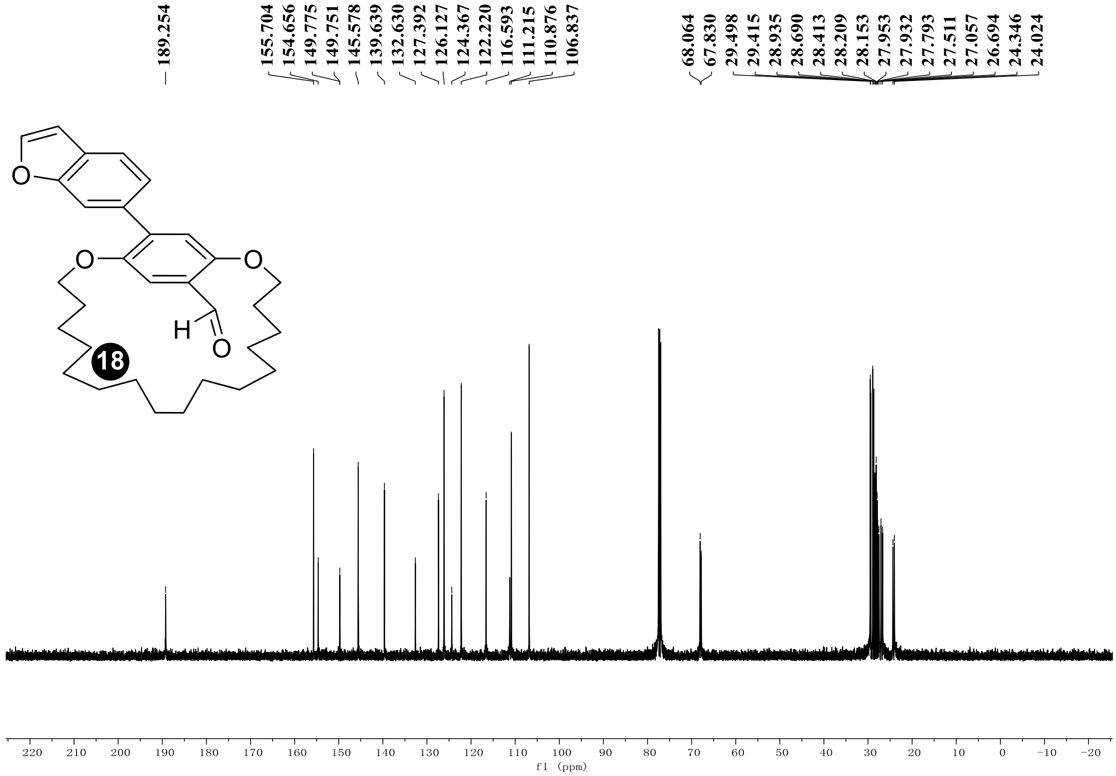

Supplementary Figure 20. ^{1}H NMR and ^{13}C NMR spectrum of compound of **3aa**

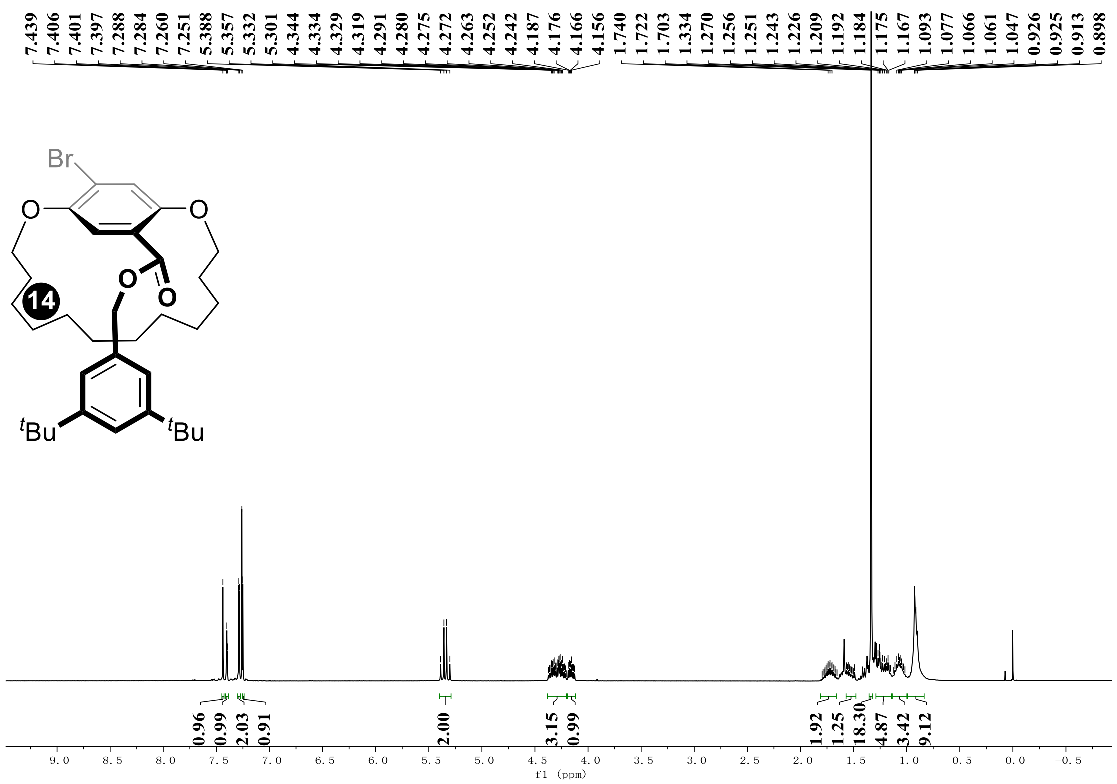

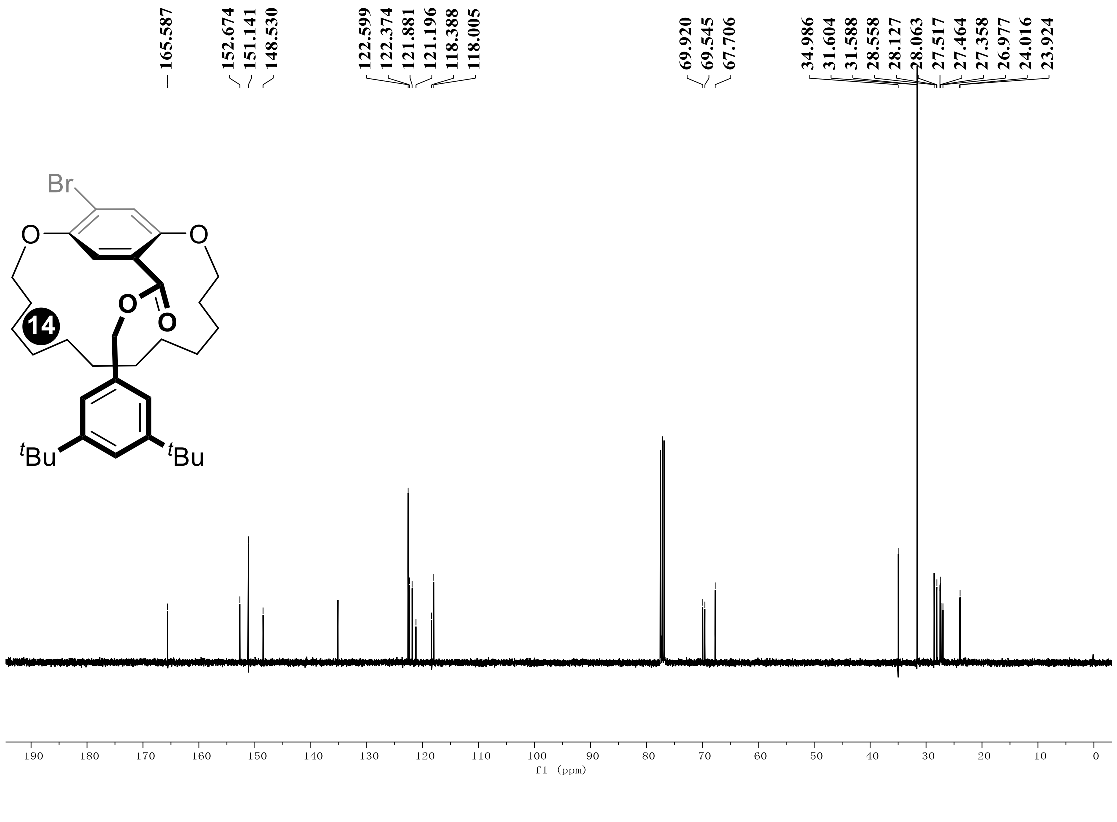

Supplementary Figure 21. ^{1}H NMR and ^{13}C NMR spectrum of compound of **3ab**

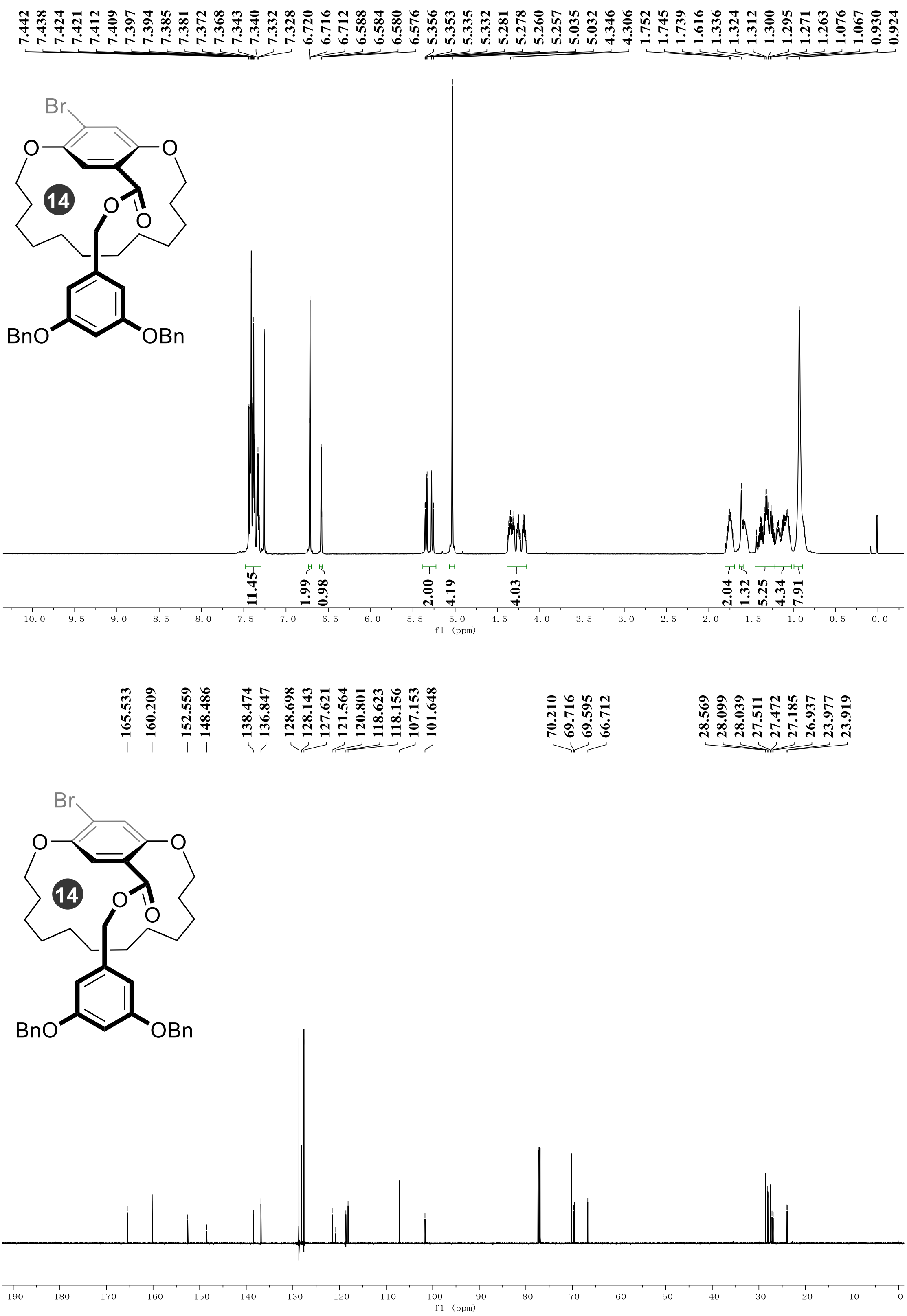

Supplementary Figure 22. ^{1}**H NMR and** ^{13}**C NMR** spectrum of compound of **3ac**

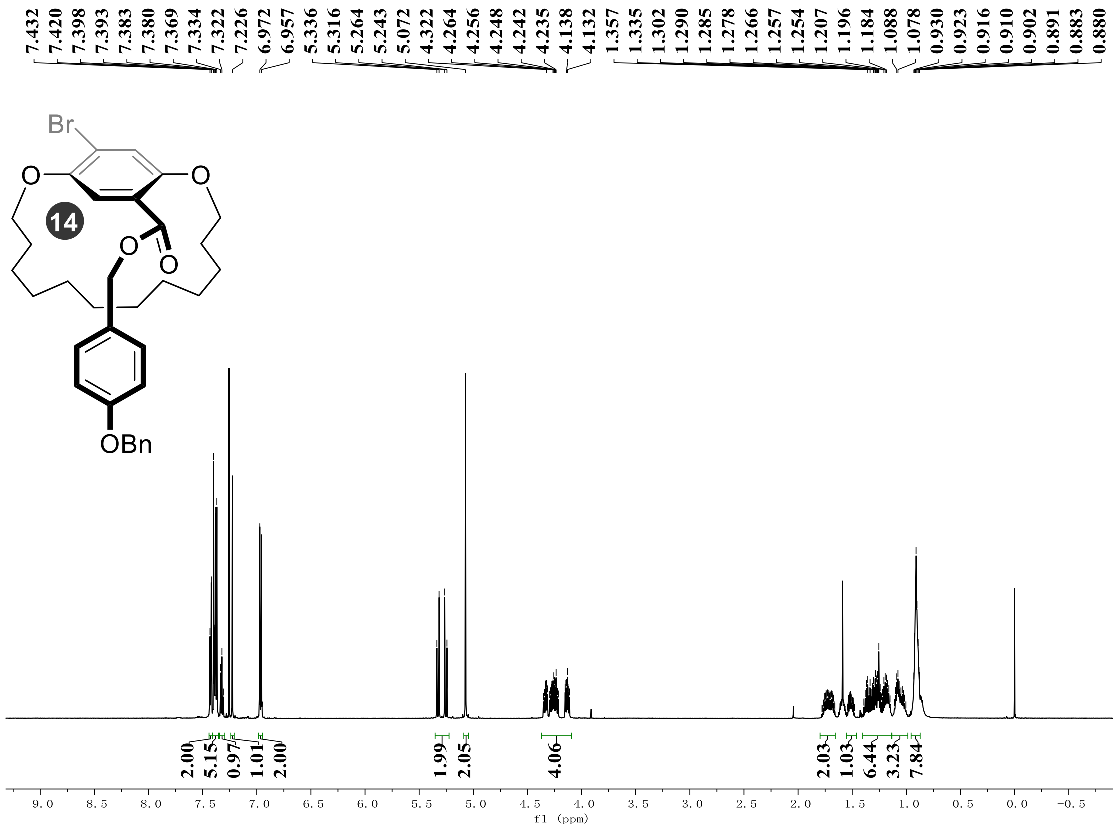

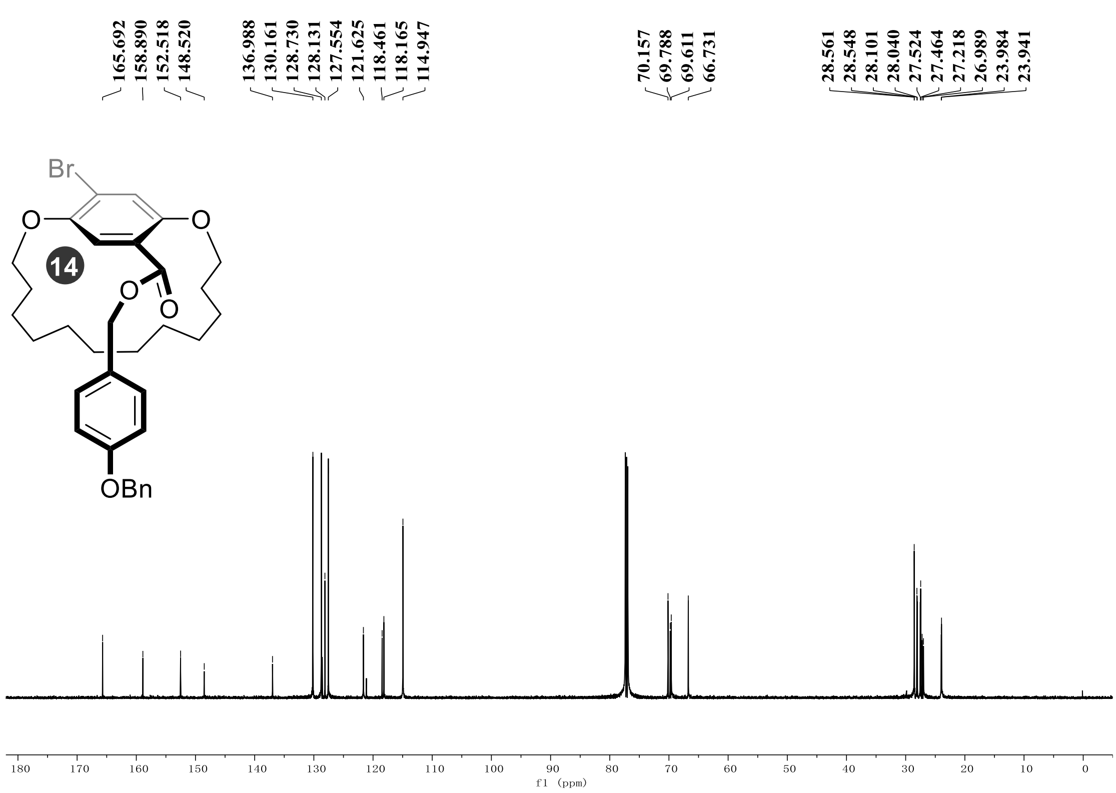

Supplementary Figure 23. ^{1}H NMR and ^{13}C NMR spectrum of compound of **3ad**

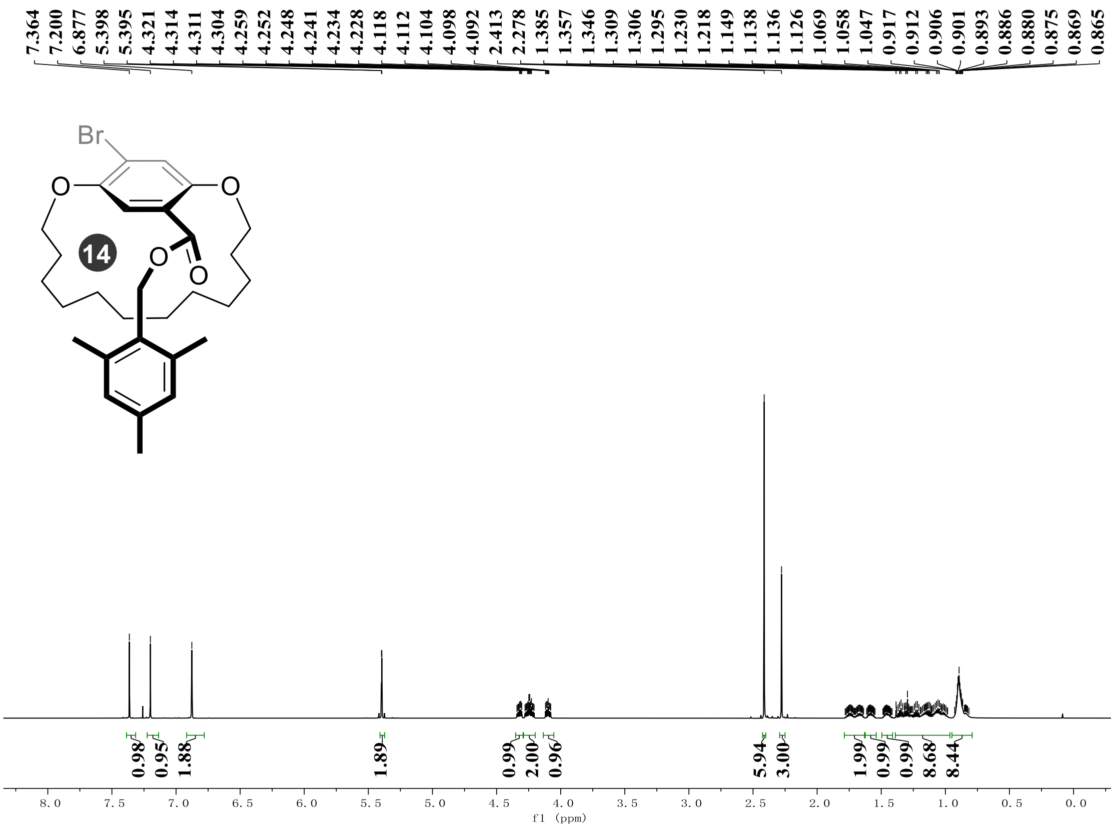

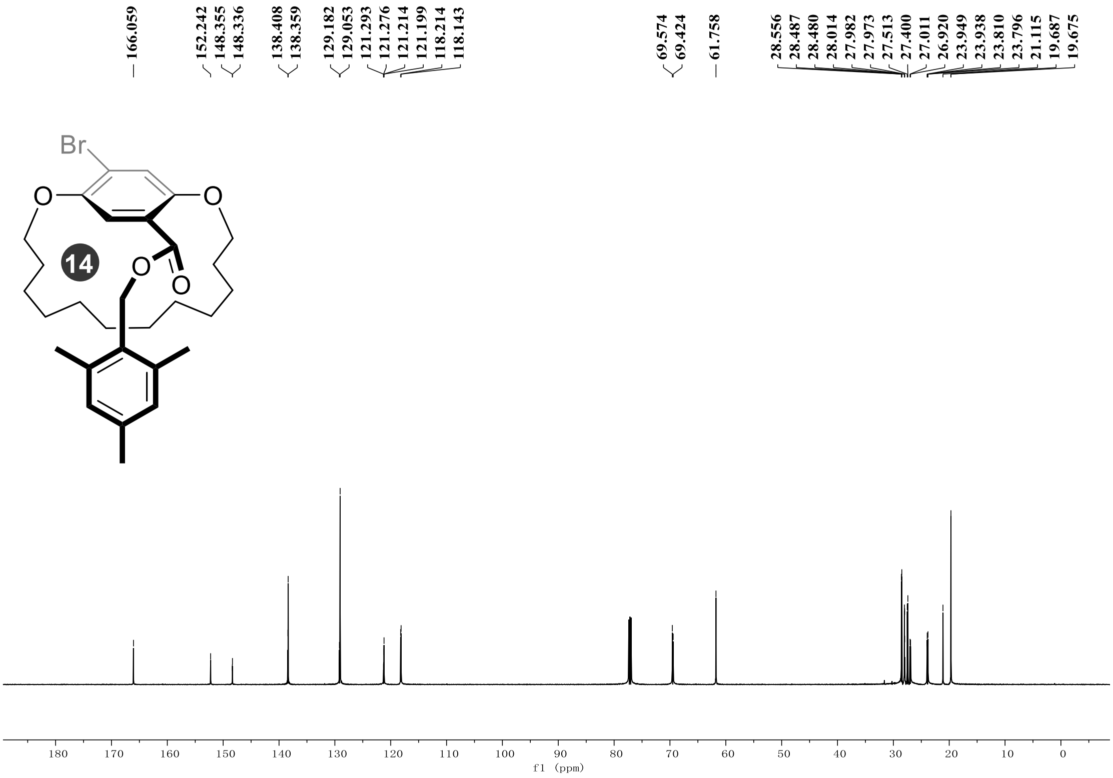

Supplementary Figure 24. ^{1}**H NMR and** ^{13}**C NMR** spectrum of compound of **3ae**

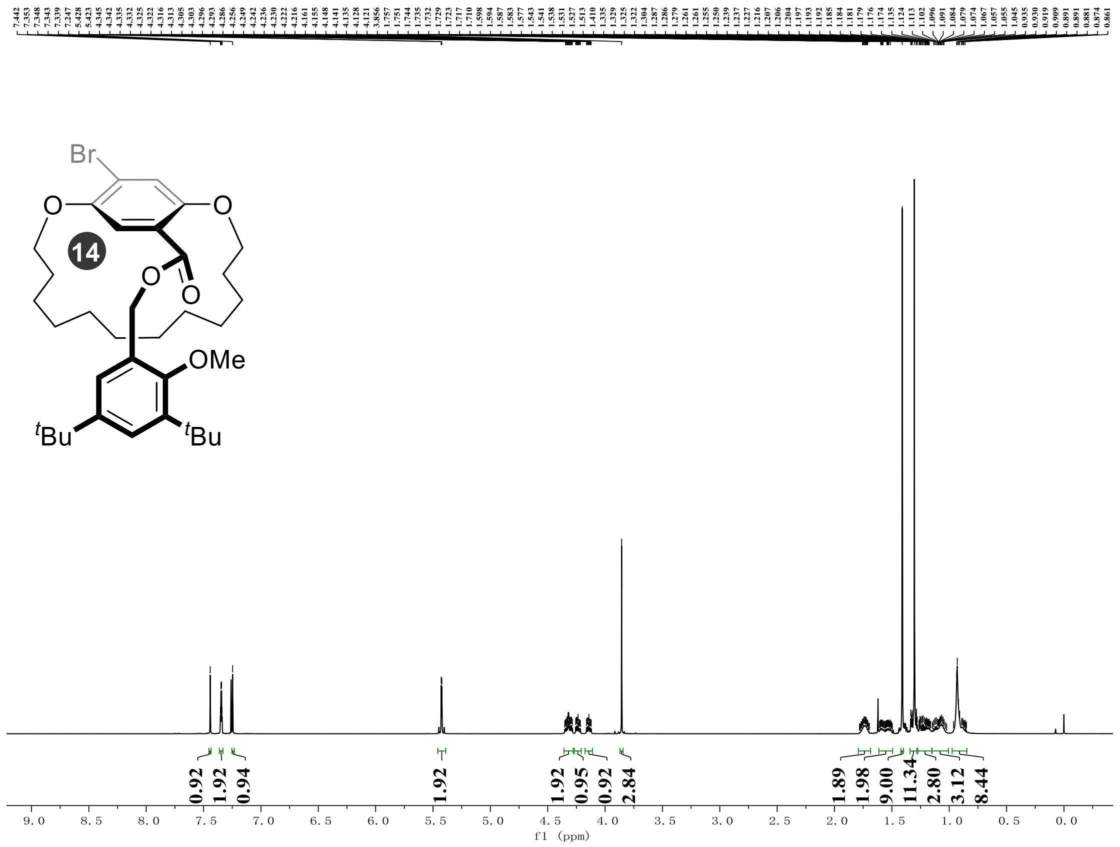

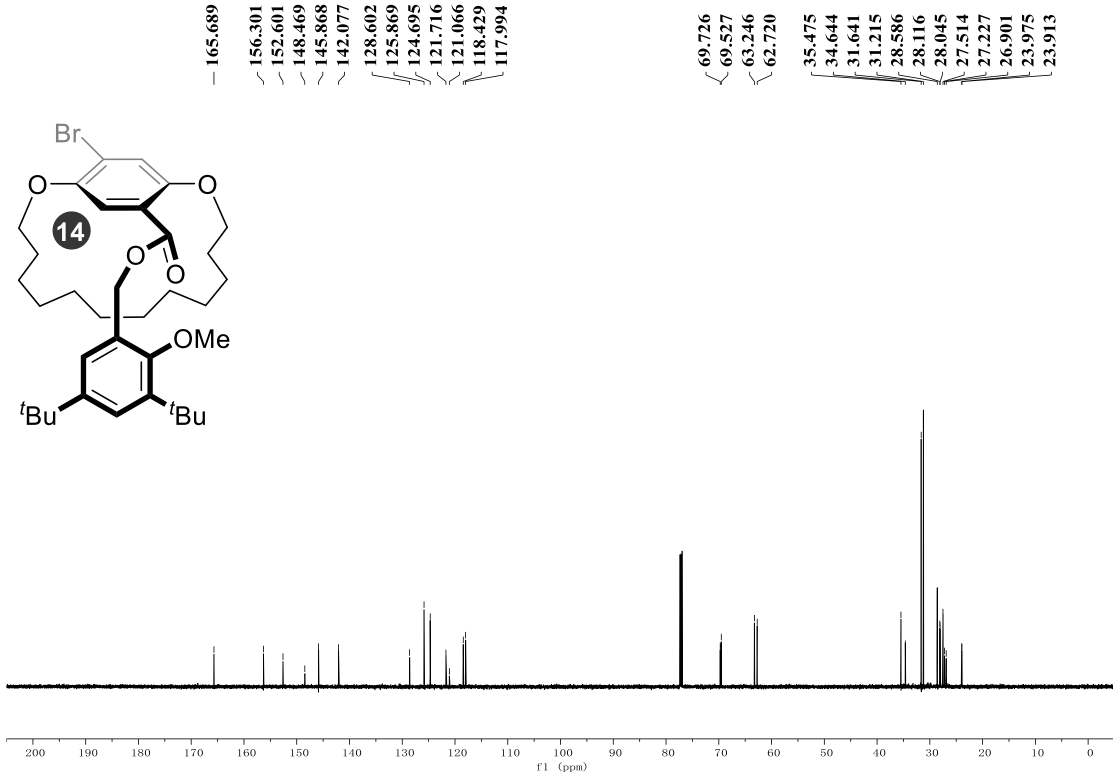

Supplementary Figure 25. ^{1}H NMR and ^{13}C NMR spectrum of compound of **3af**

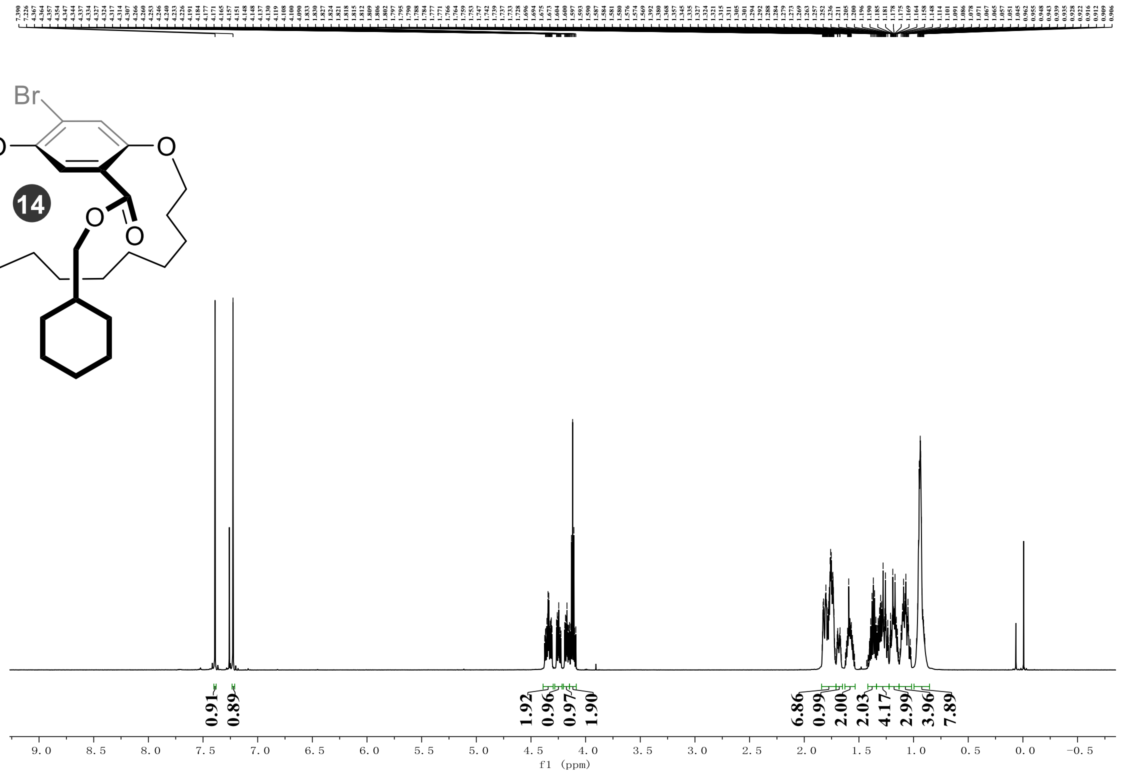

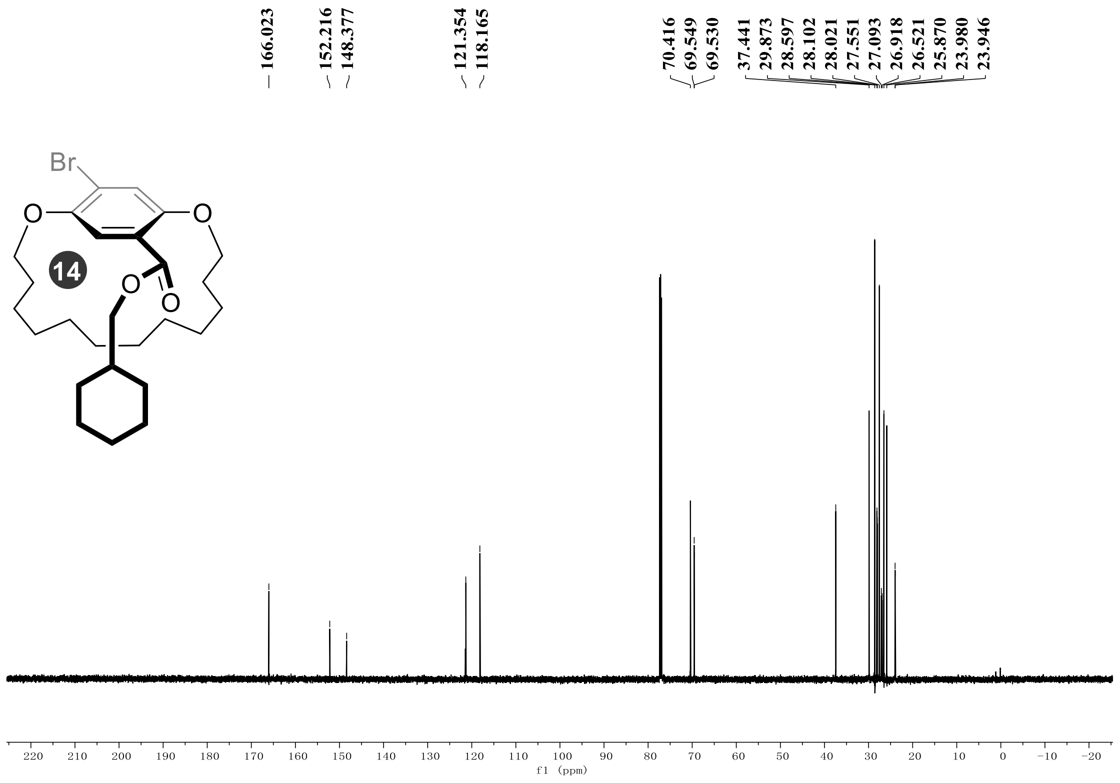

Supplementary Figure 26. ^{1}H NMR and ^{13}C NMR spectrum of compound of **3ag**

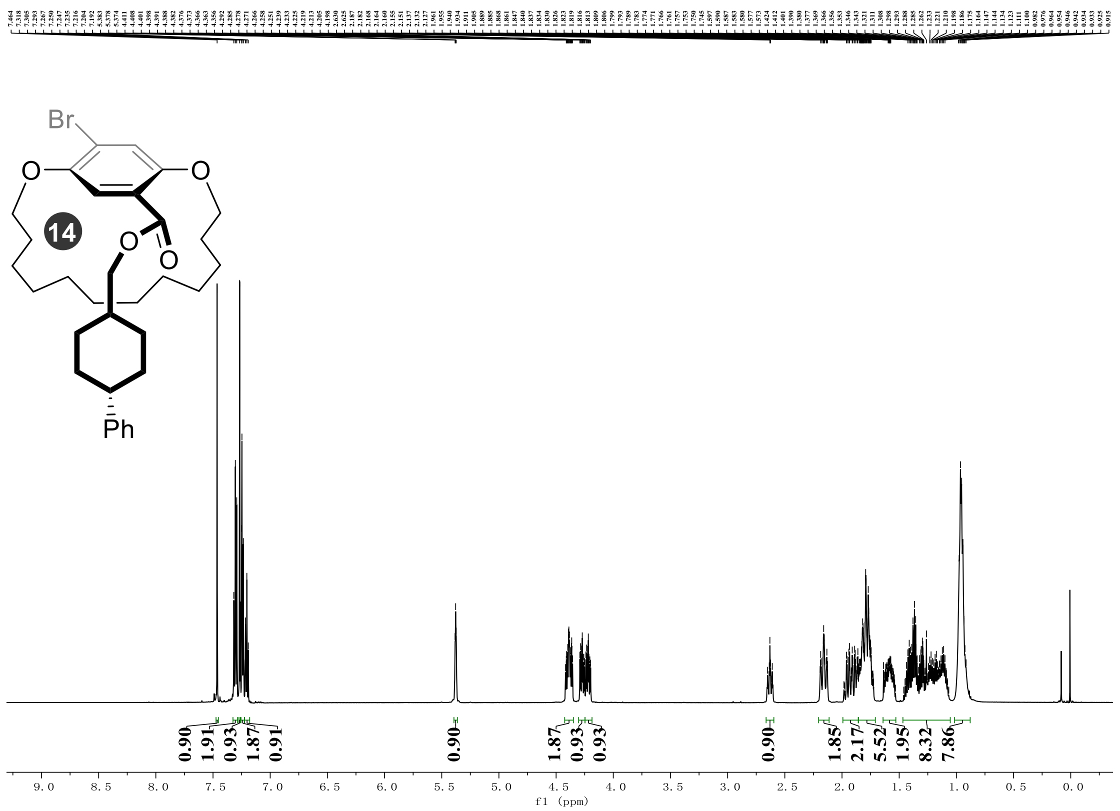

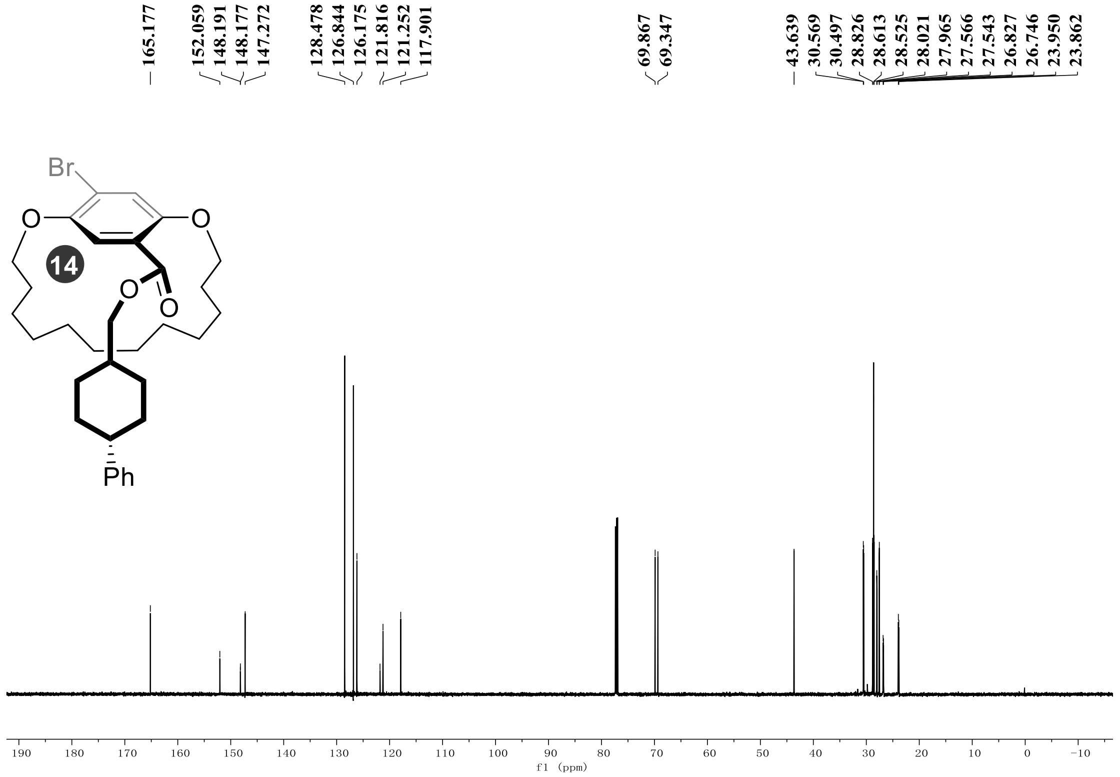

Supplementary Figure 27. ^{1}H NMR and ^{13}C NMR spectrum of compound of **3ah**

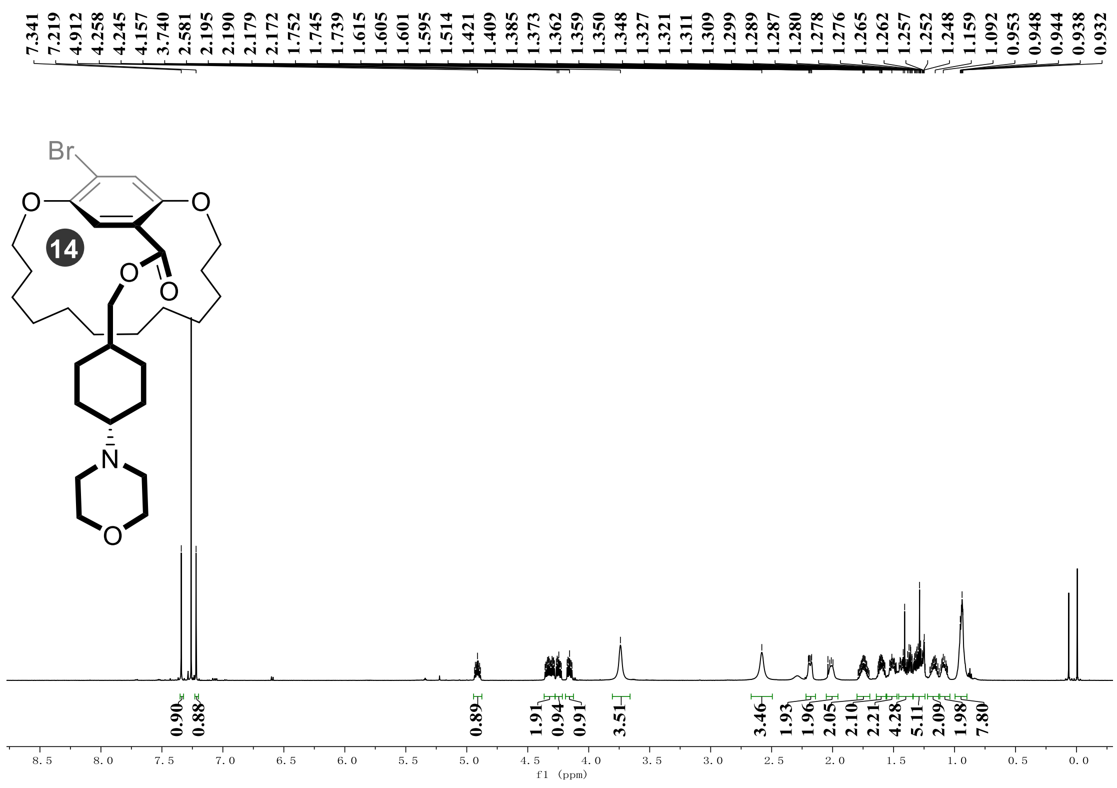

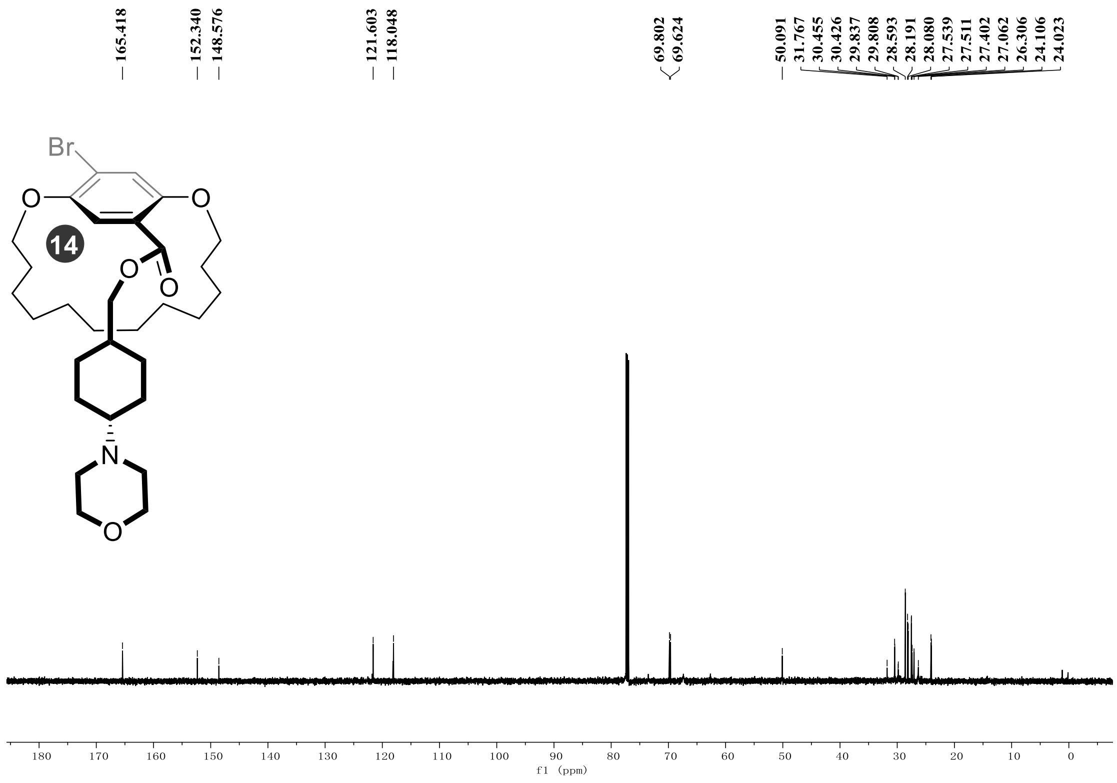

Supplementary Figure 28. ^{1}H NMR and ^{13}C NMR spectrum of compound of **3ai**

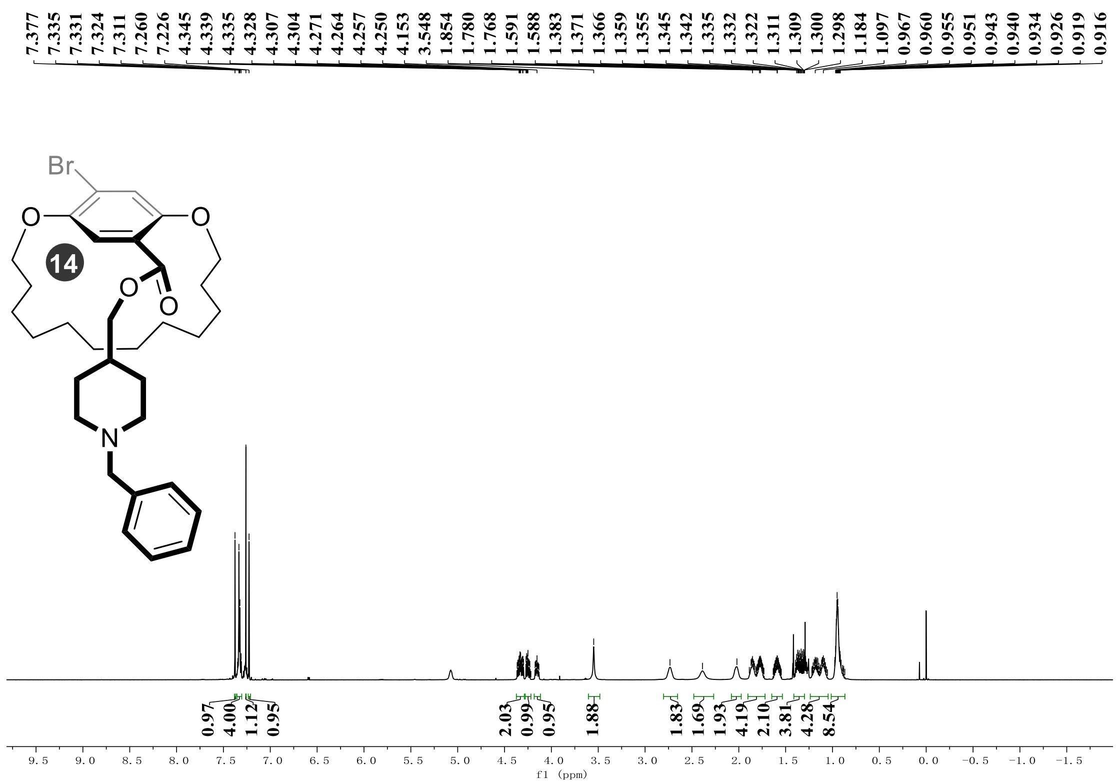

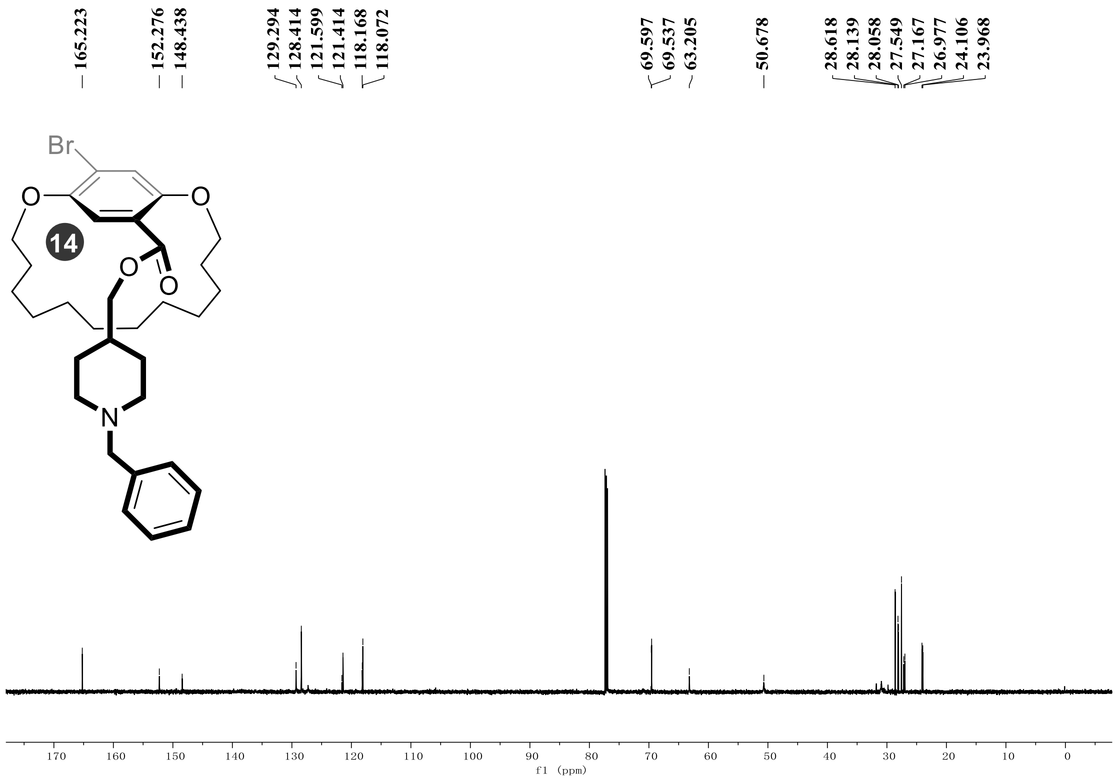

Supplementary Figure 29. ^{1}H NMR and ^{13}C NMR spectrum of compound of **3aj**

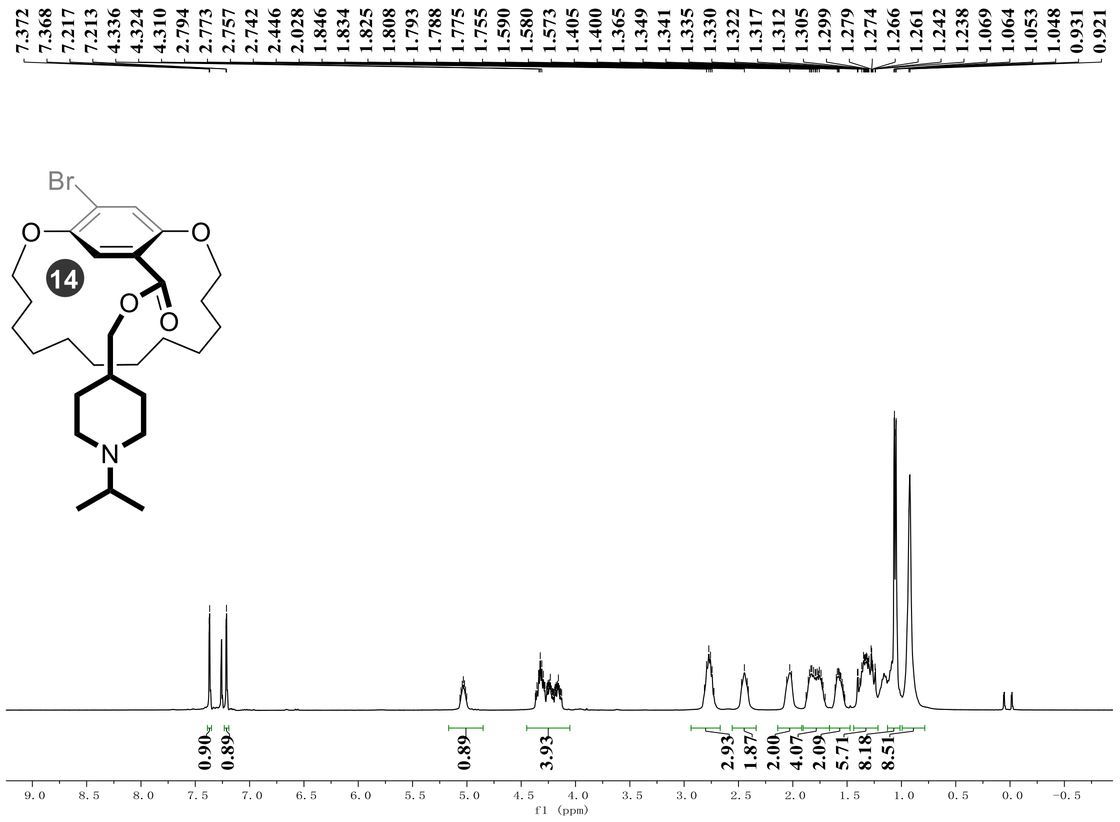

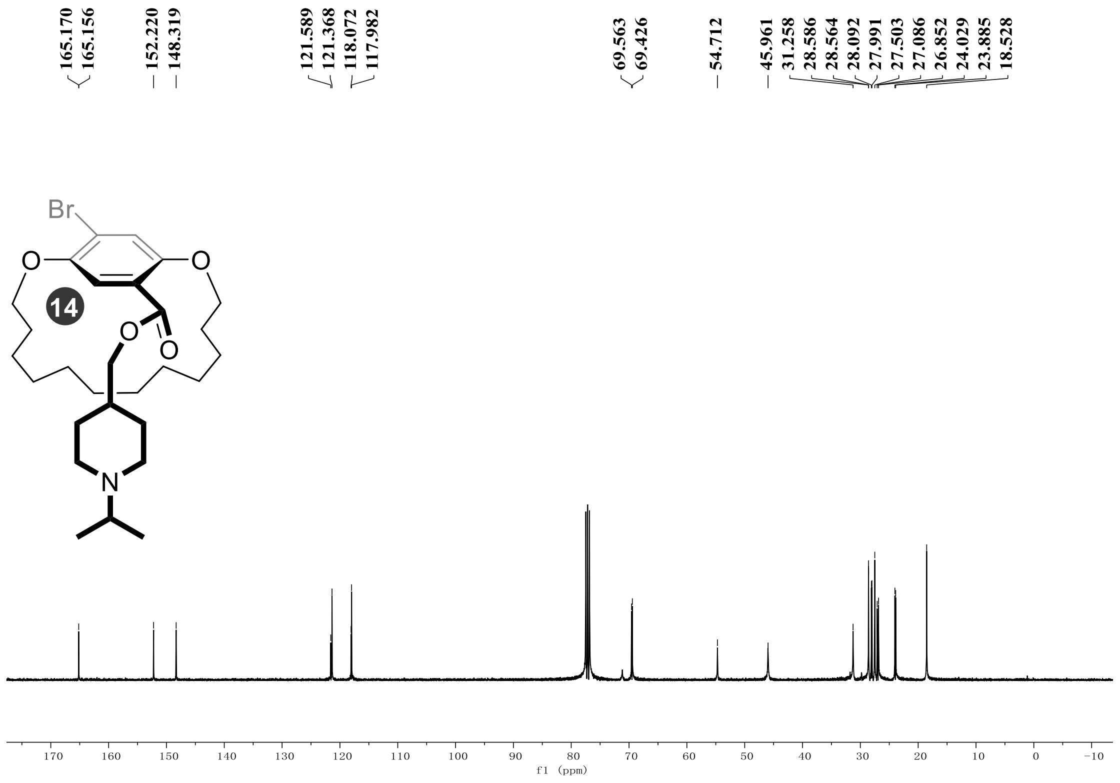

Supplementary Figure 30. ^{1}H NMR and ^{13}C NMR spectrum of compound of **3ak**

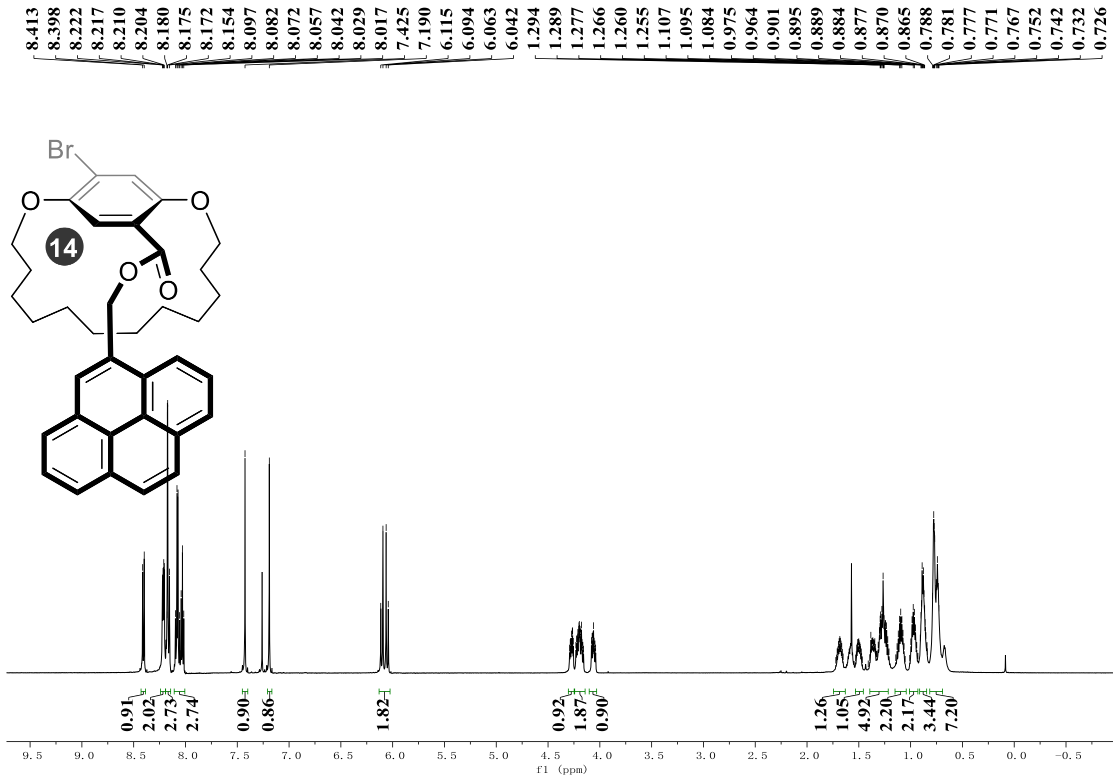

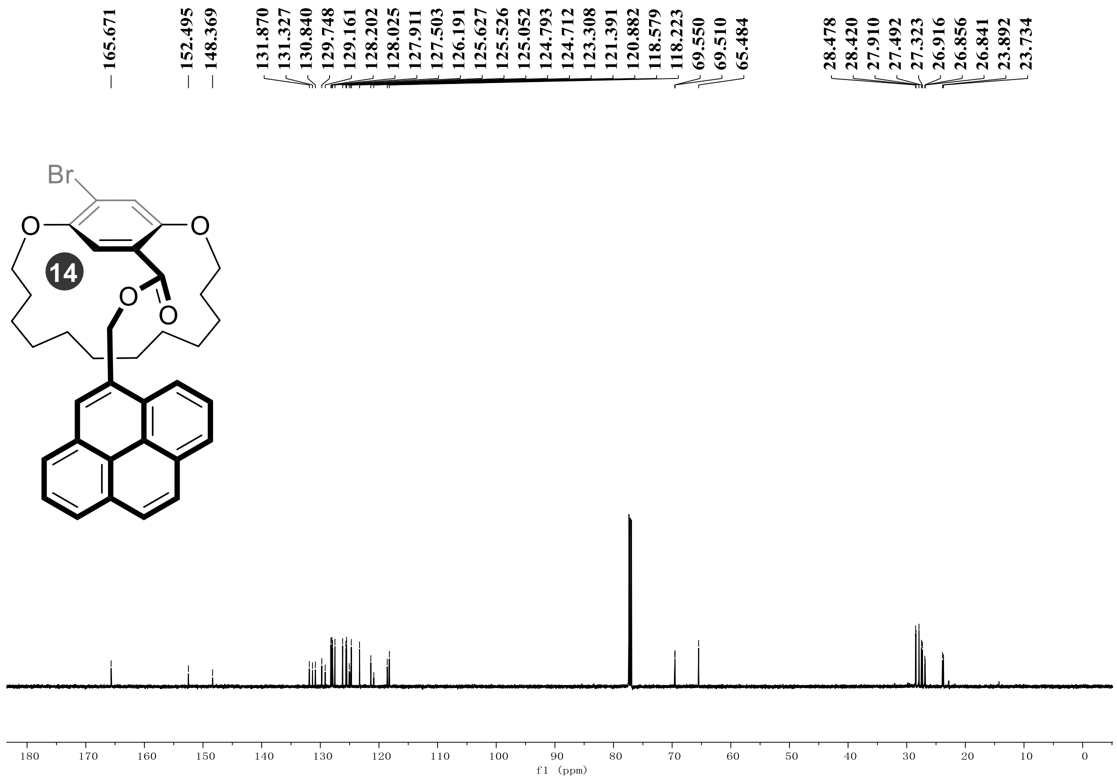

Supplementary Figure 31. ^{1}H NMR and ^{13}C NMR spectrum of compound of **3be**

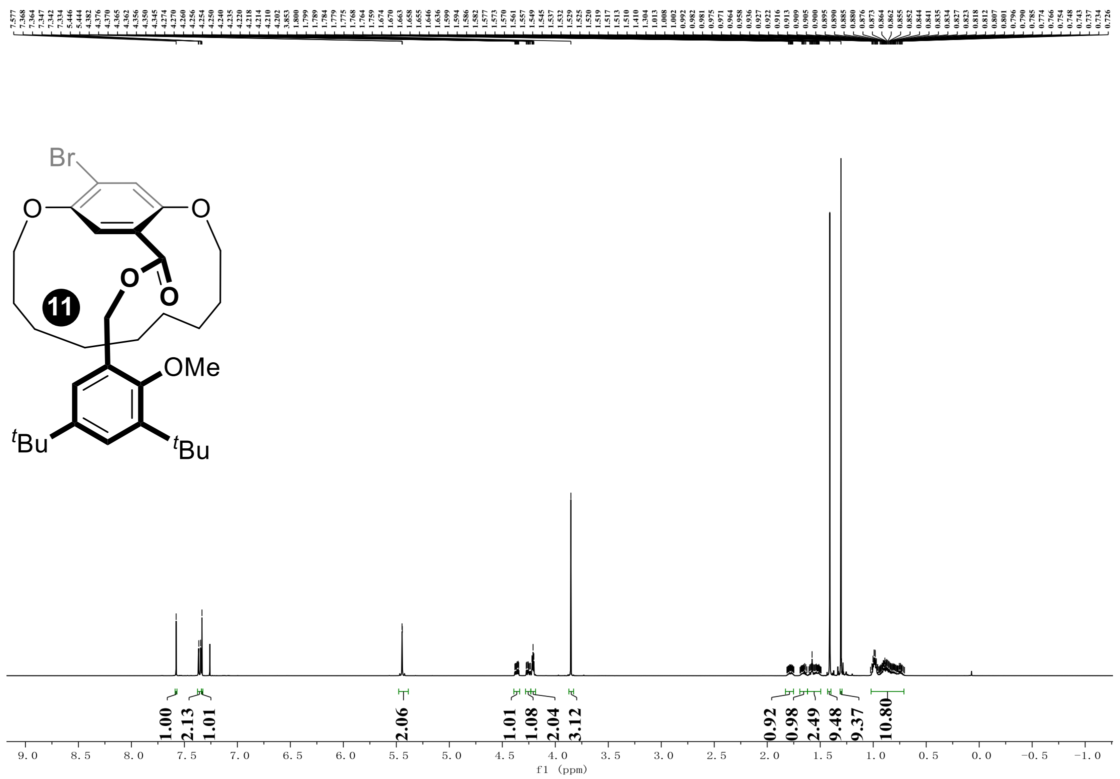

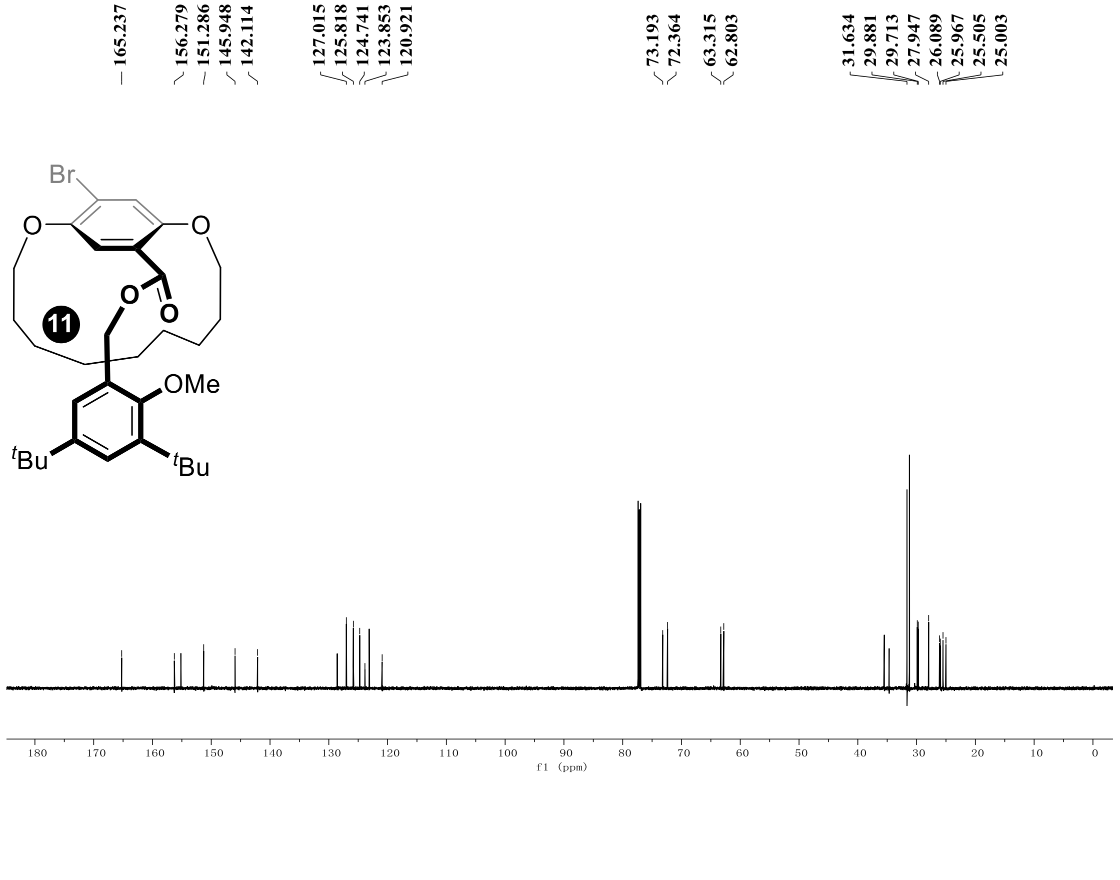

Supplementary Figure 32. ^{1}H NMR and ^{13}C NMR spectrum of compound of **3ce**

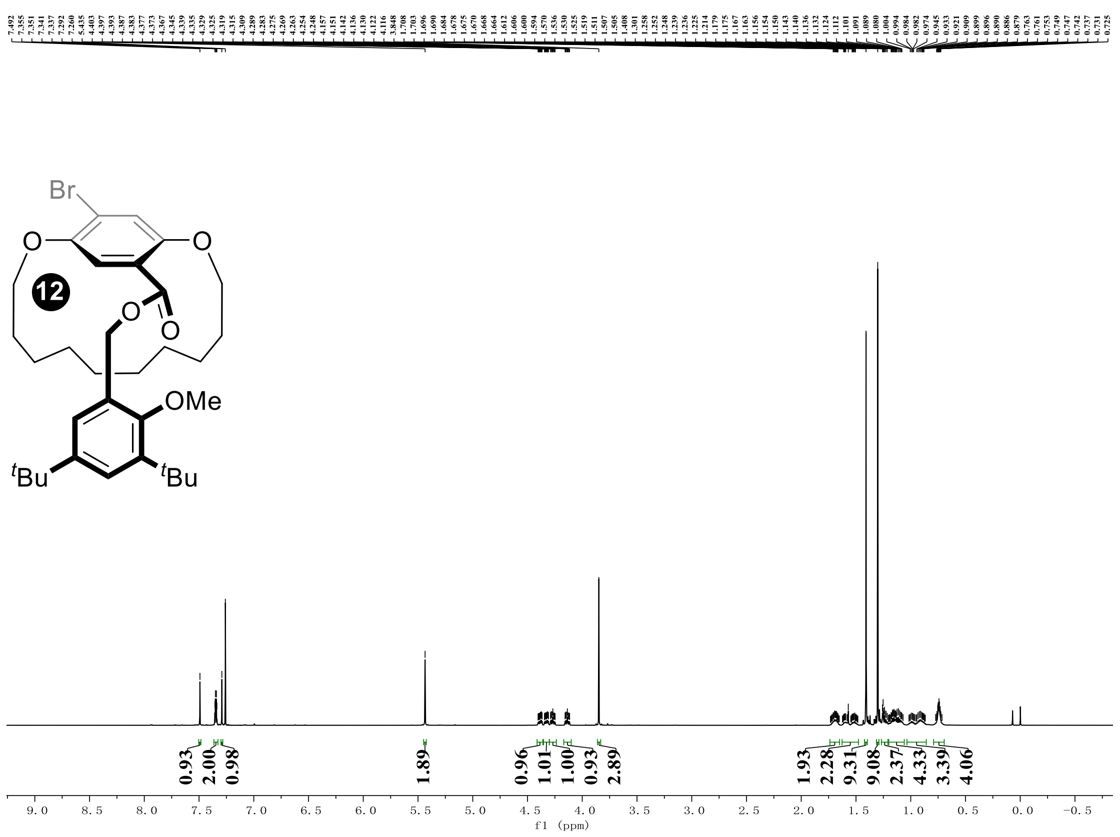

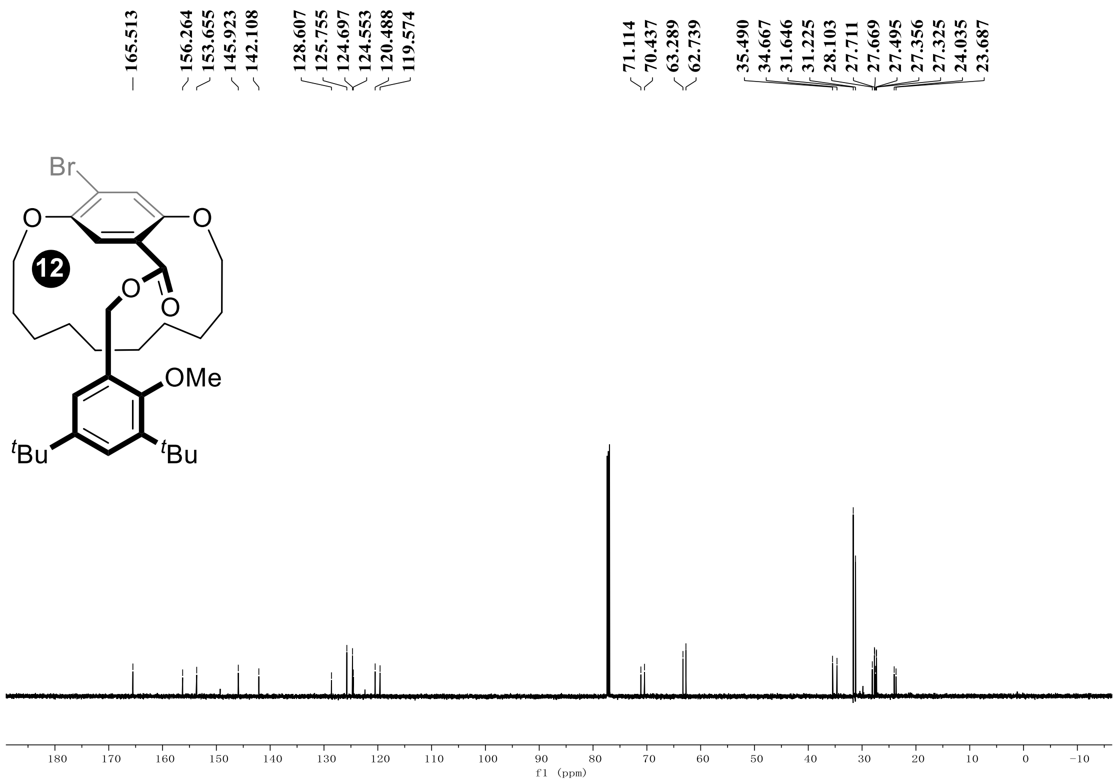

Supplementary Figure 33. ^{1}H NMR and ^{13}C NMR spectrum of compound of **3de**

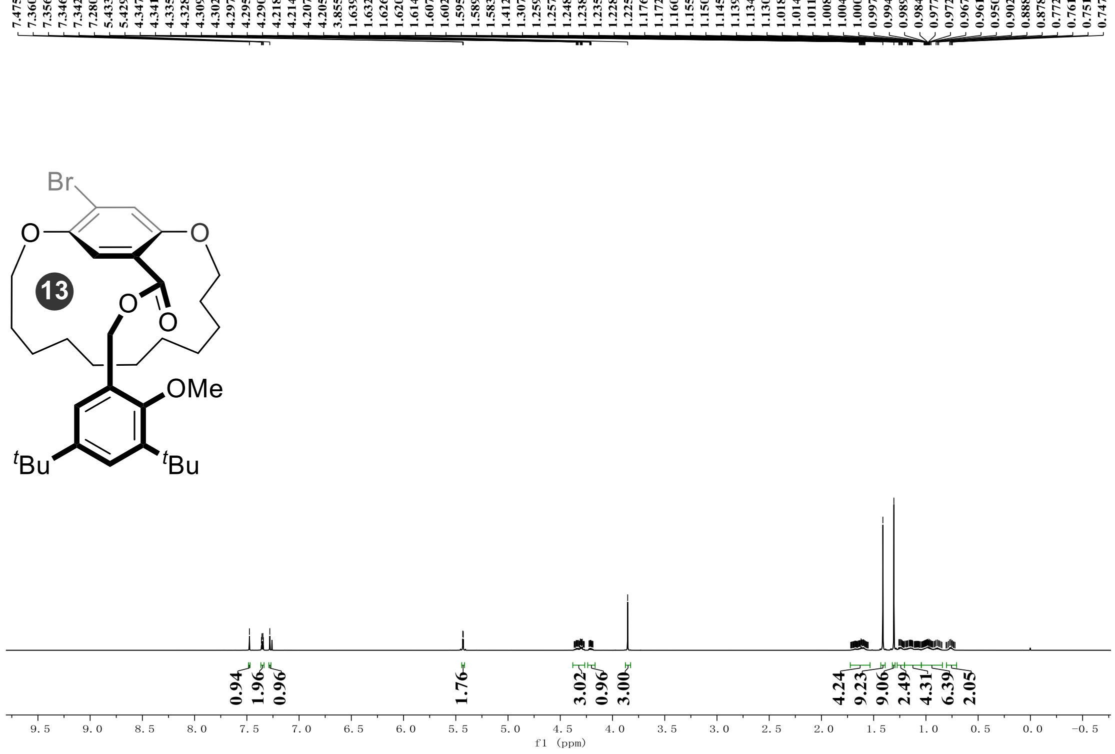

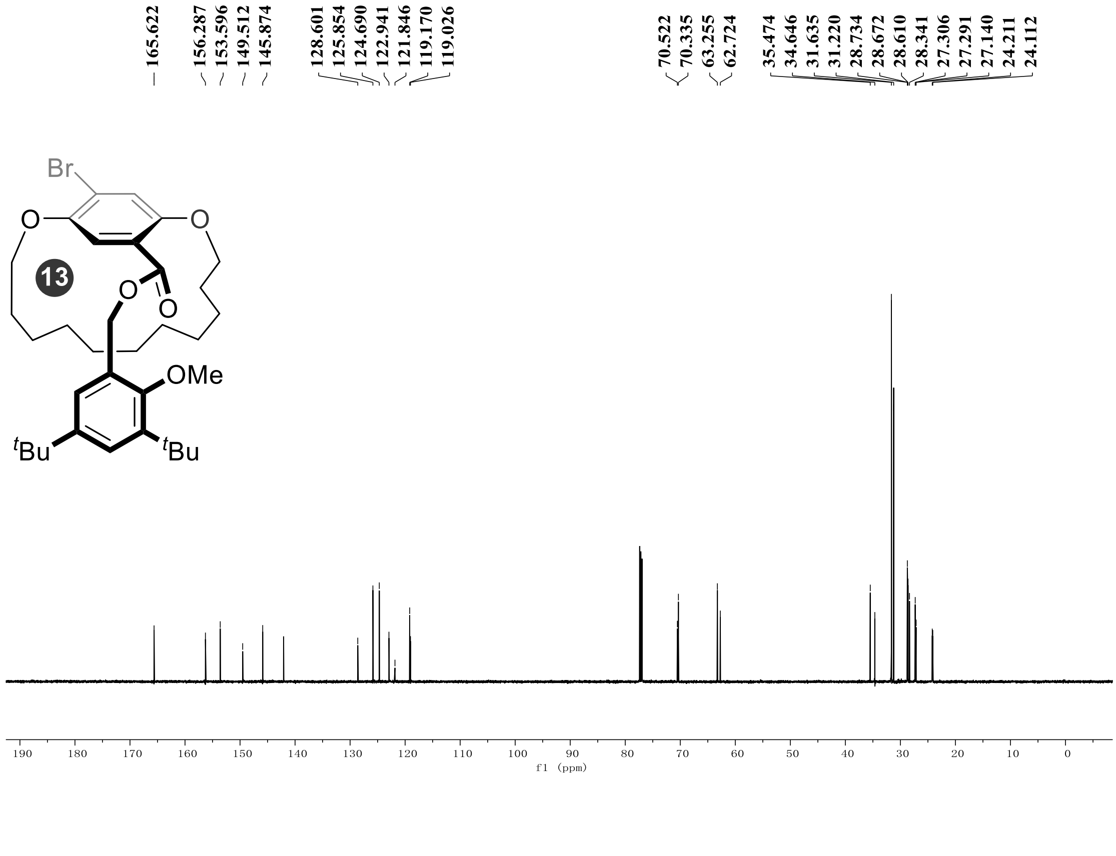

Supplementary Figure 34. ^{1}H NMR and ^{13}C NMR spectrum of compound of **3ee**

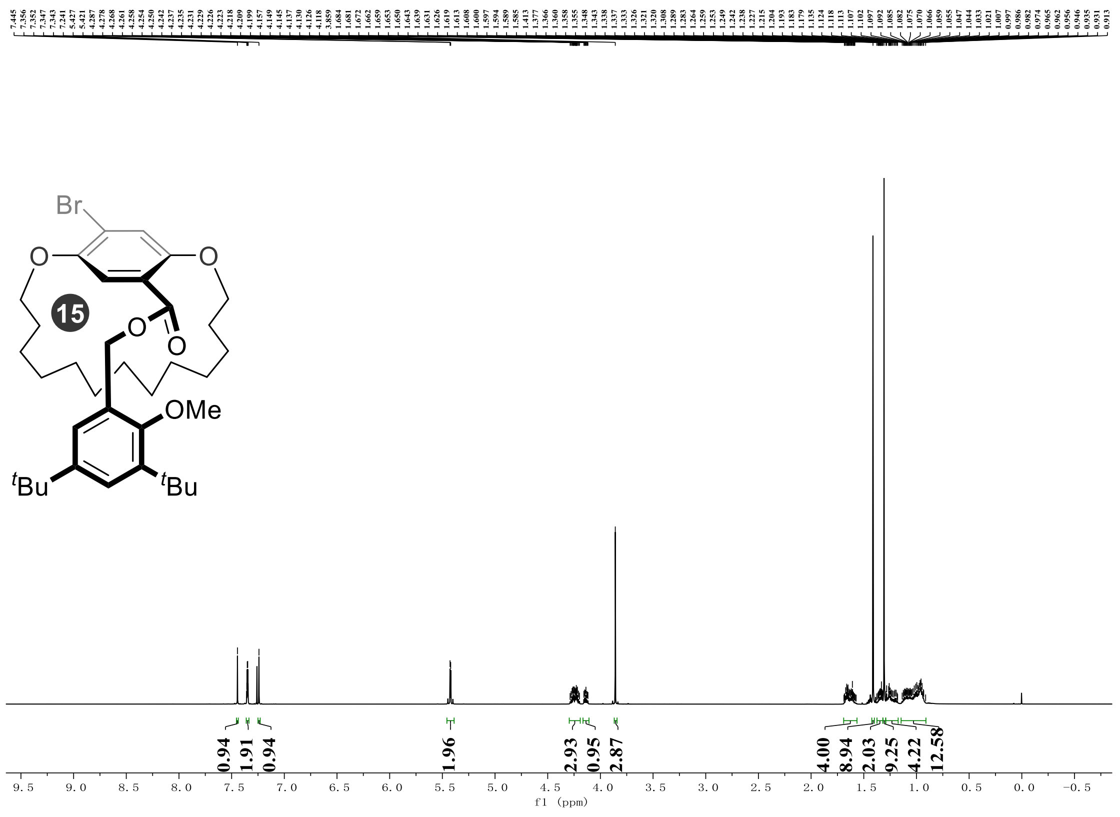

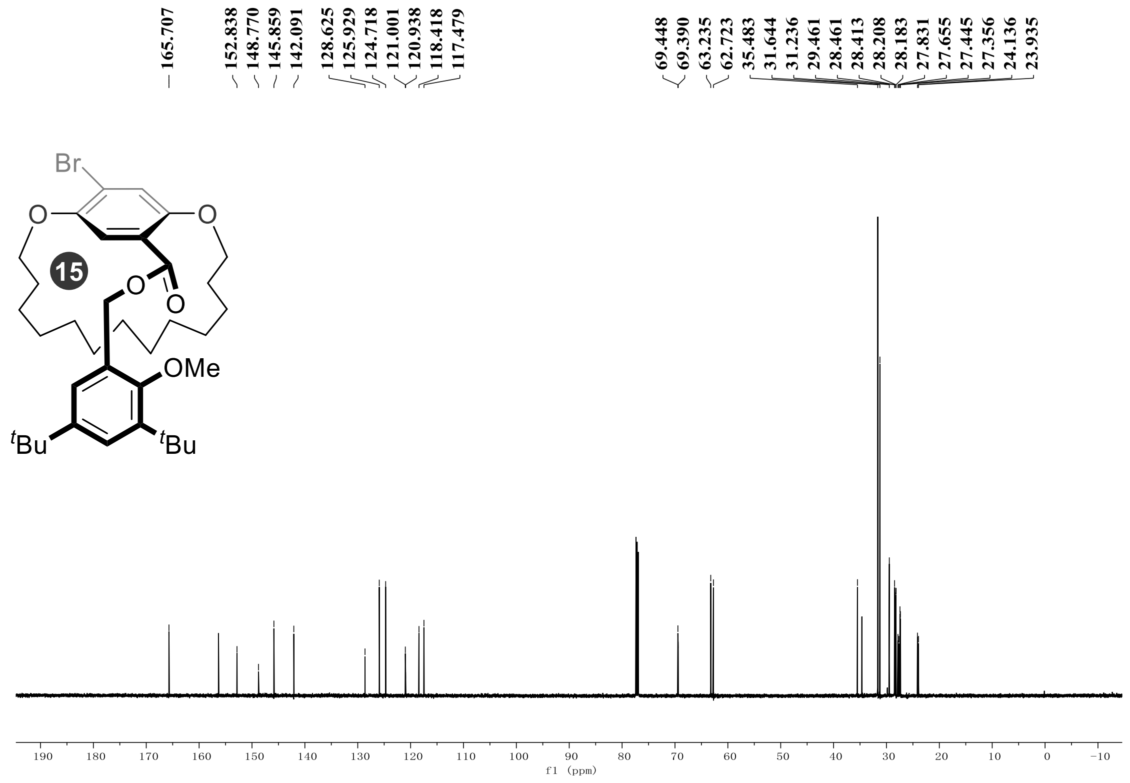

Supplementary Figure 35. ^{1}H NMR and ^{13}C NMR spectrum of compound of **3fe**

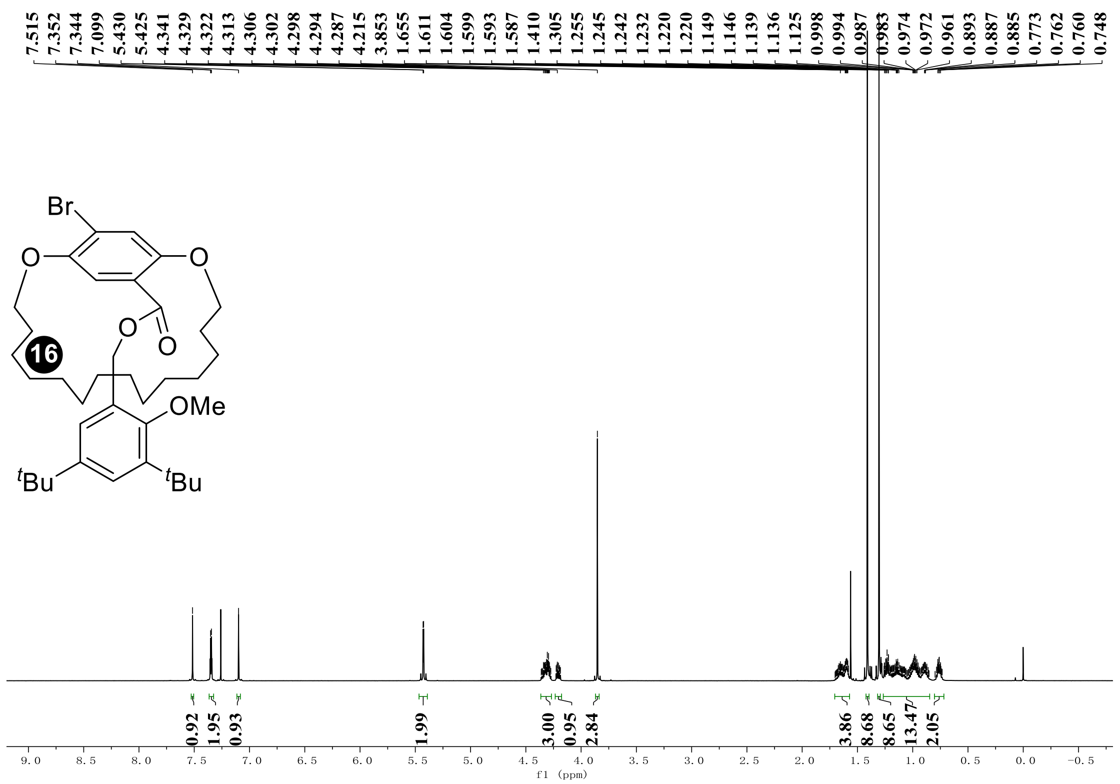

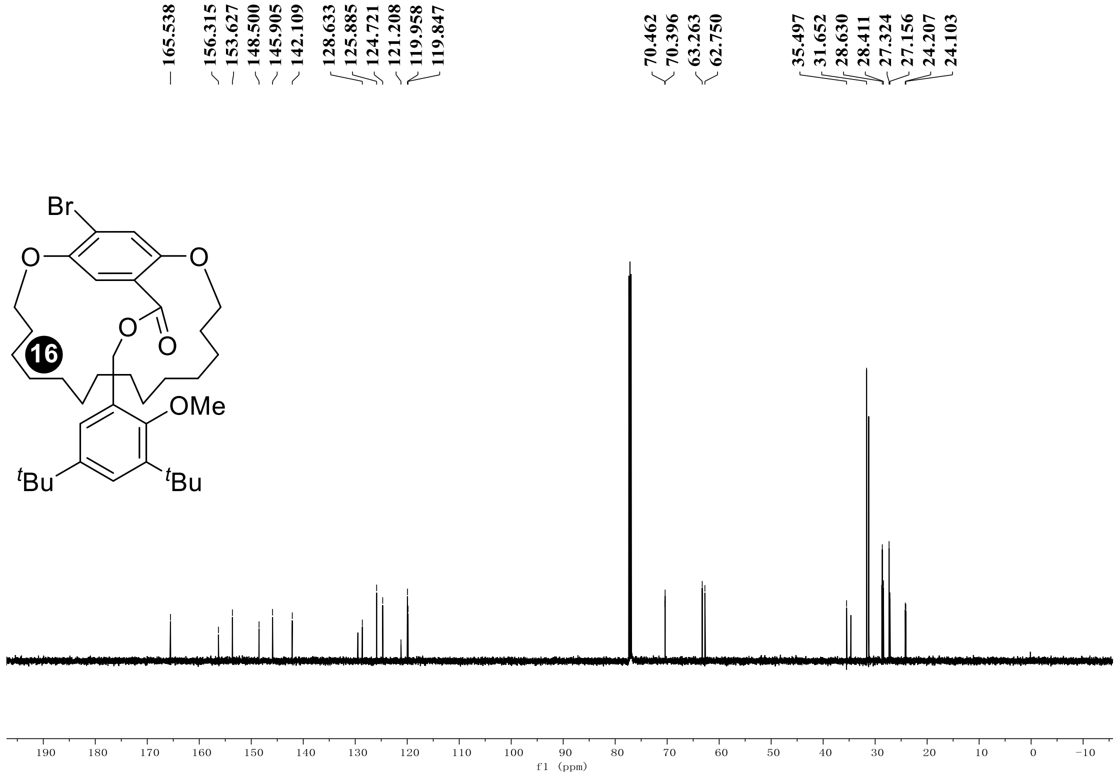

Supplementary Figure 36. ^{1}H NMR and ^{13}C NMR spectrum of compound of **3ge**

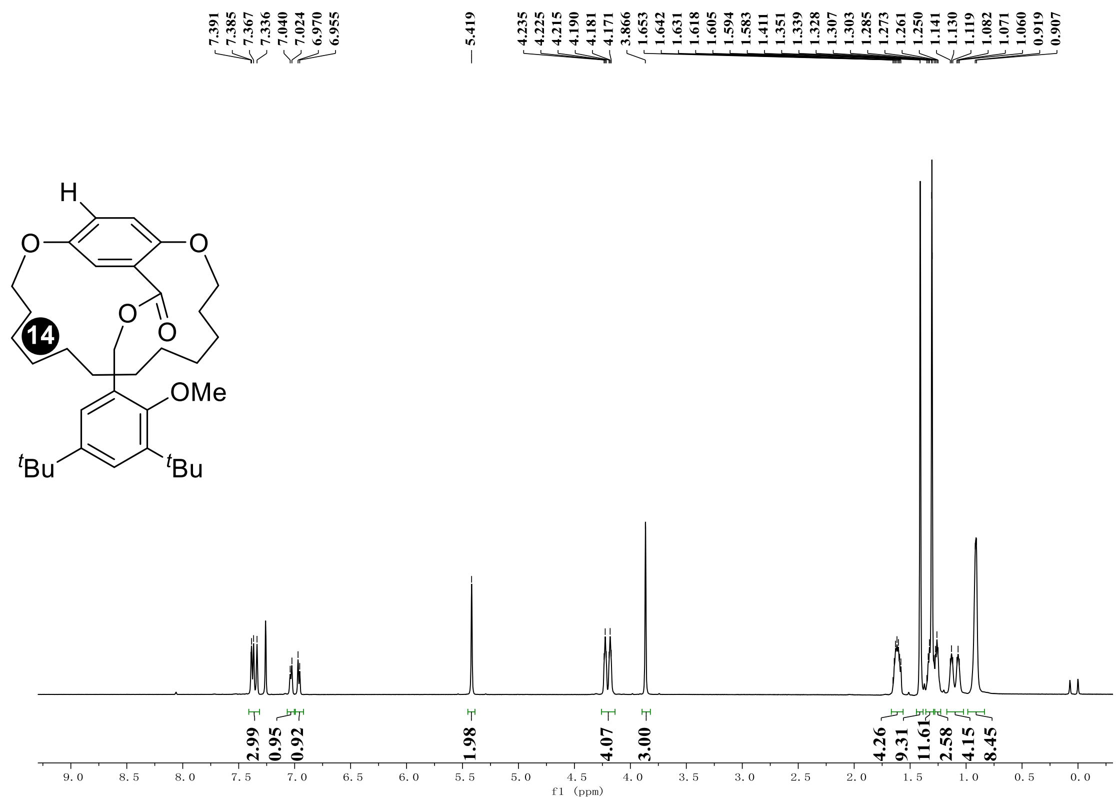

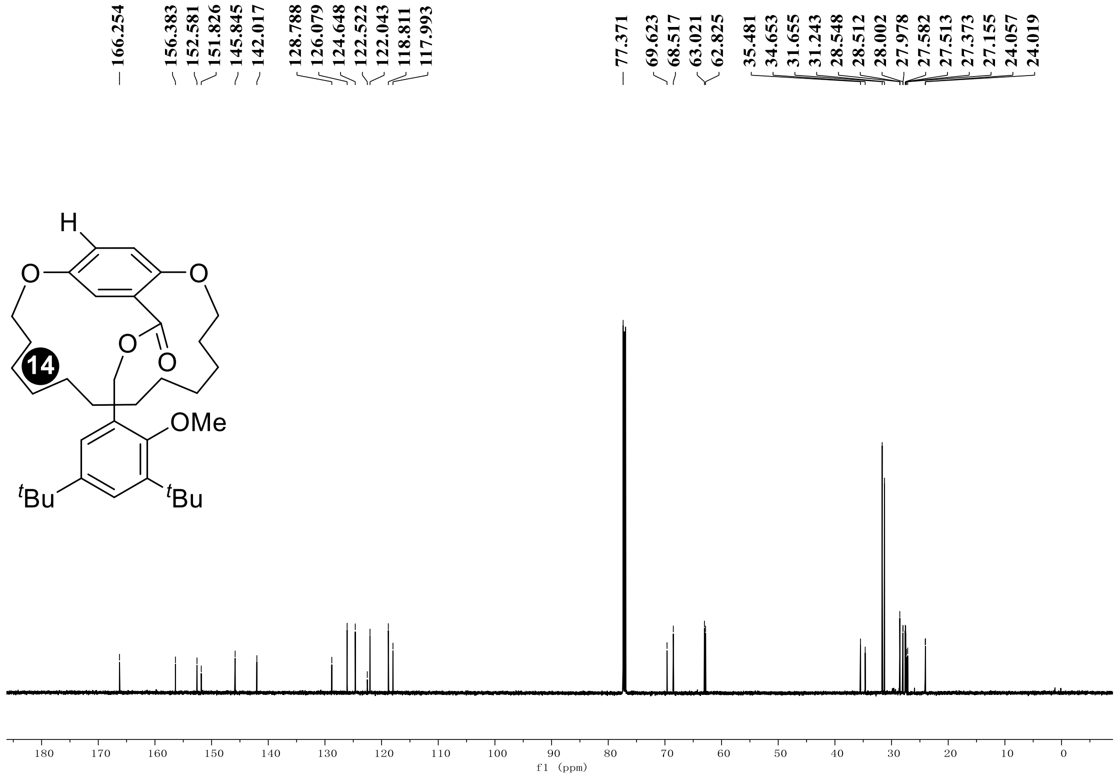

Supplementary Figure 37. ^{1}H NMR and ^{13}C NMR spectrum of compound of **3he**

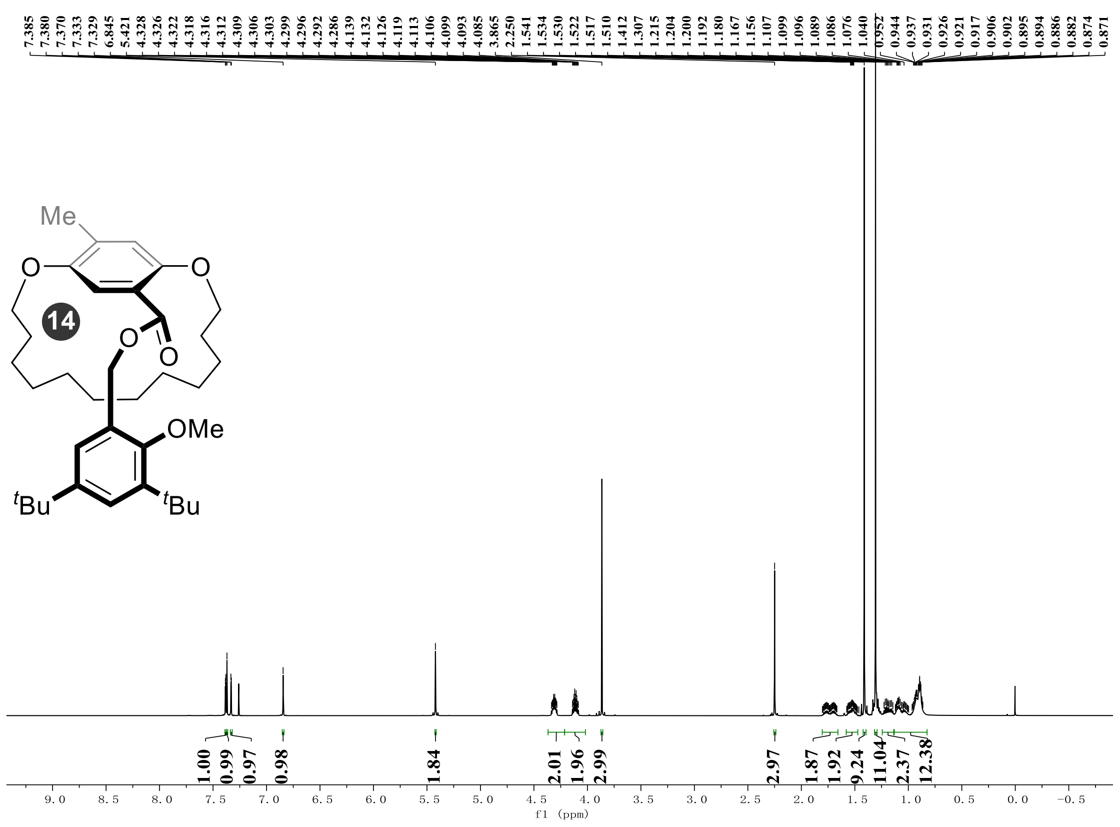

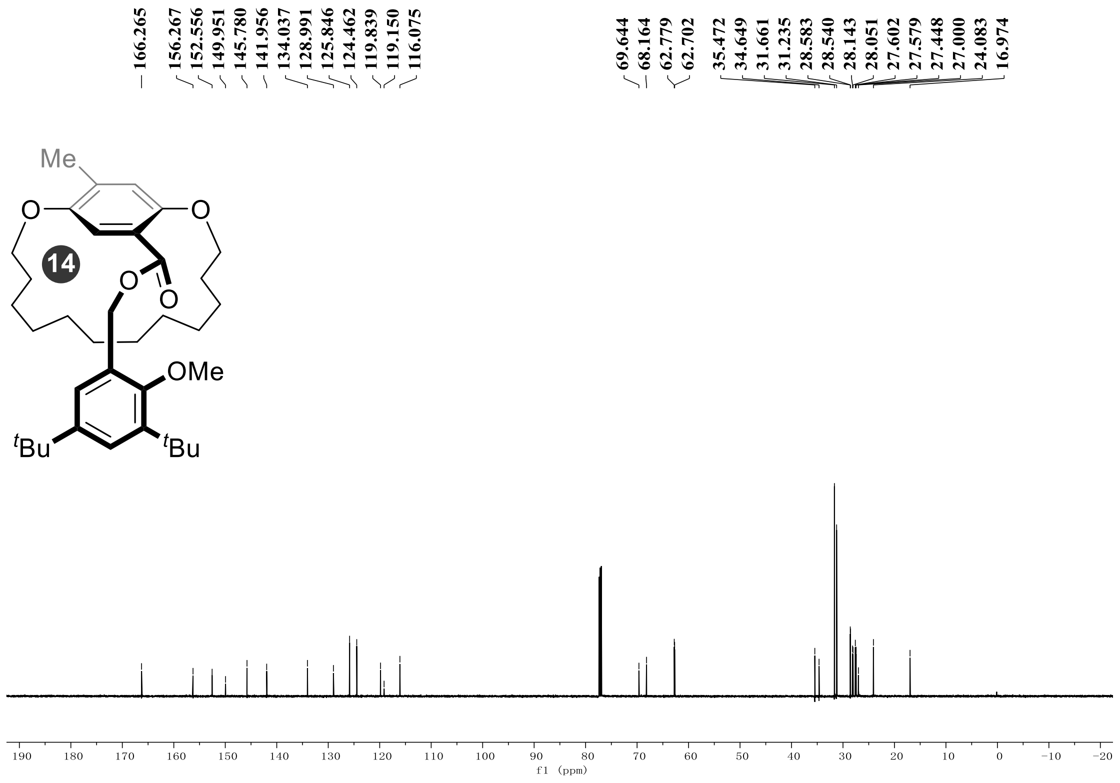

Supplementary Figure 38. ^{1}H NMR and ^{13}C NMR spectrum of compound of **3ie**

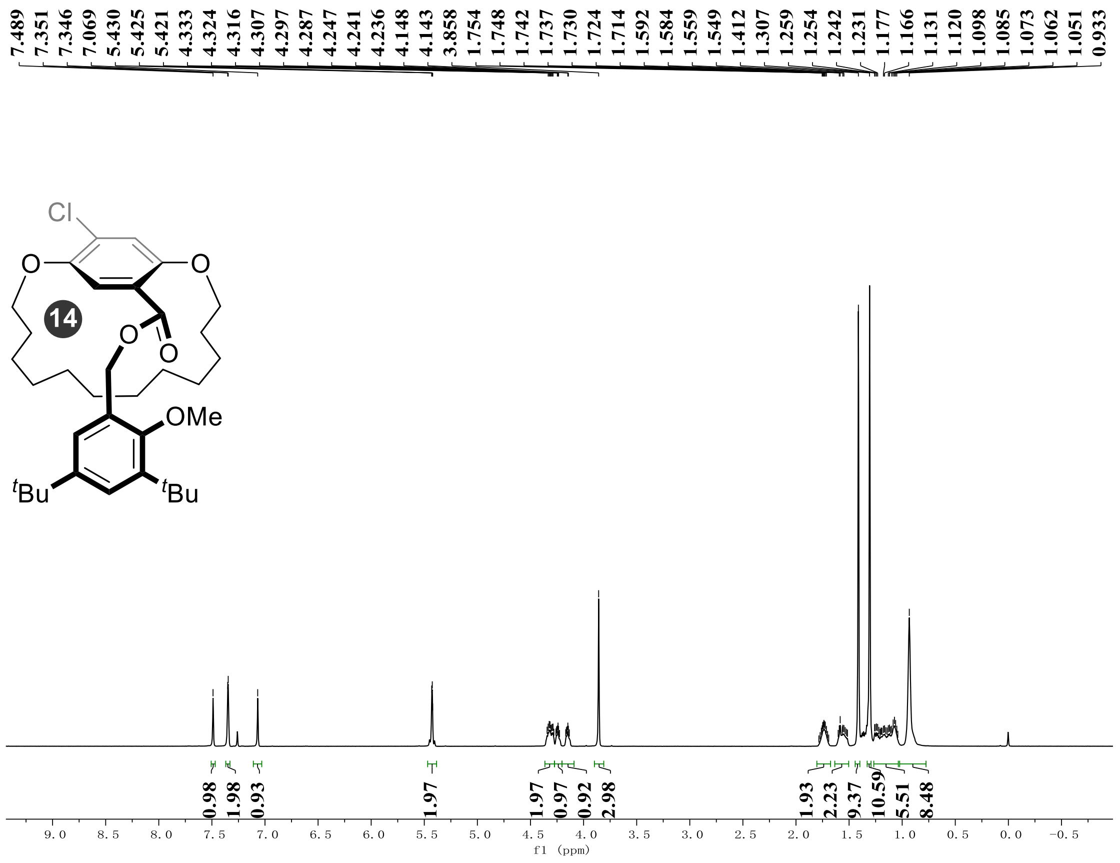

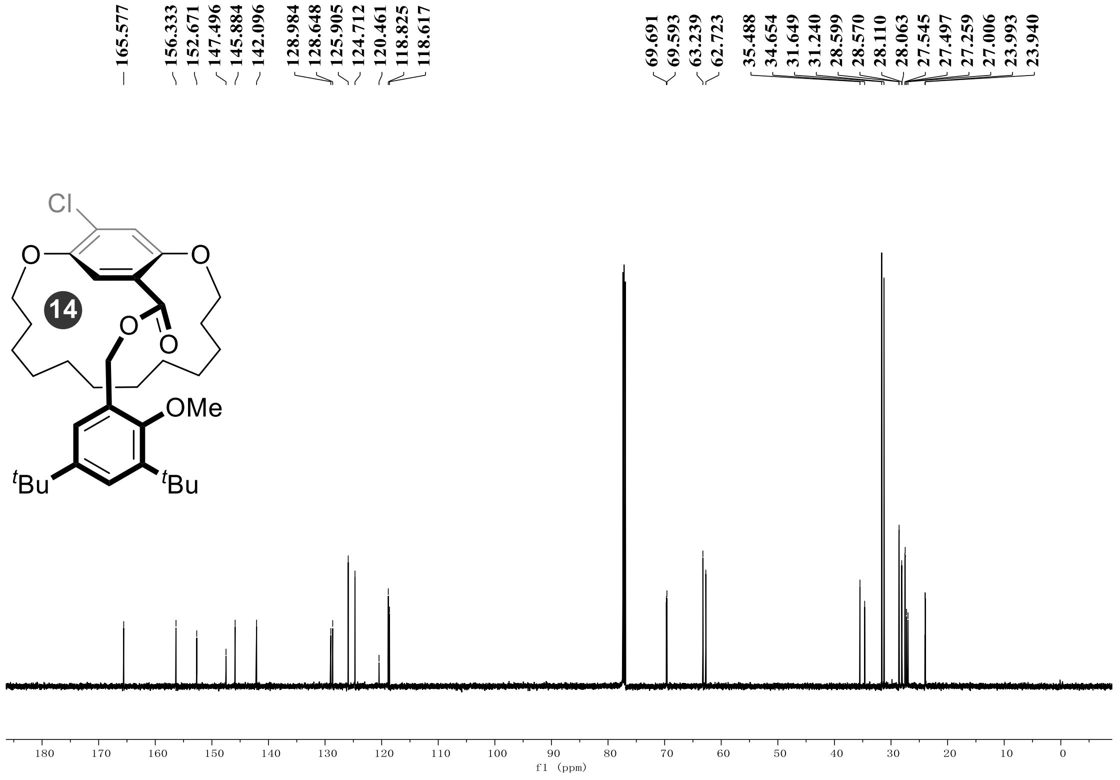

Supplementary Figure 39. ^{1}H NMR and ^{13}C NMR spectrum of compound of **3je**

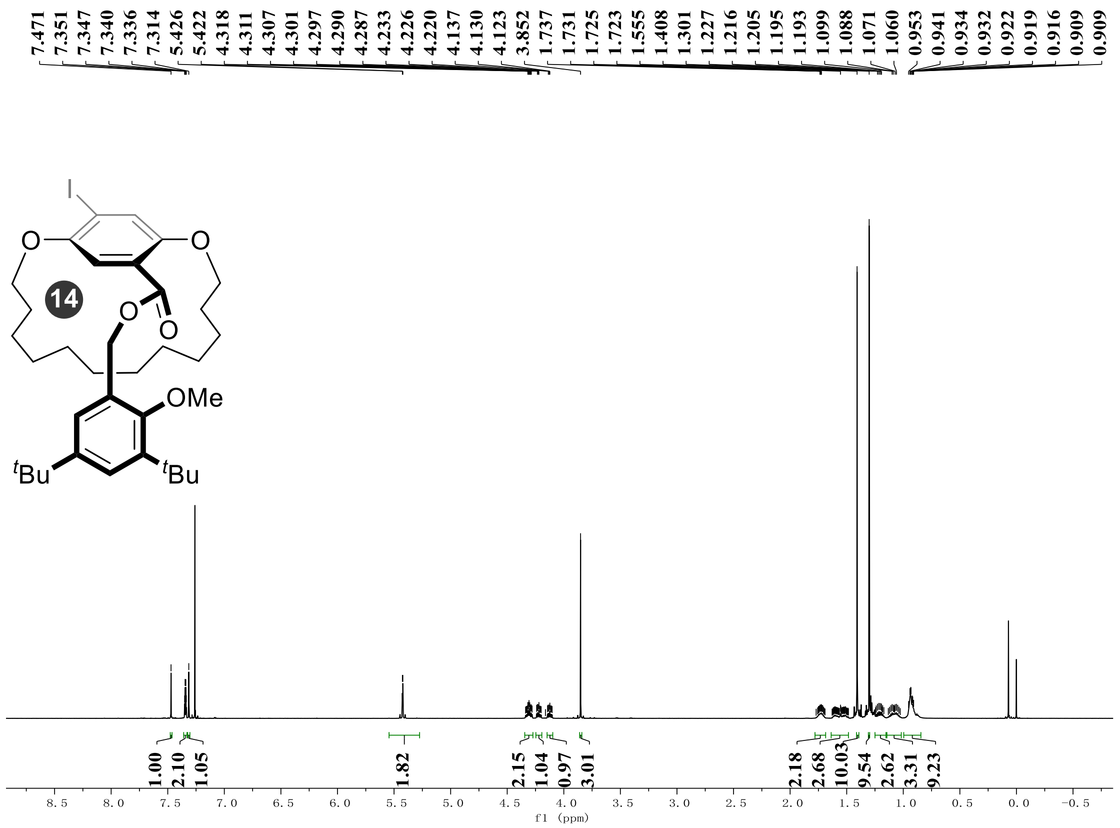

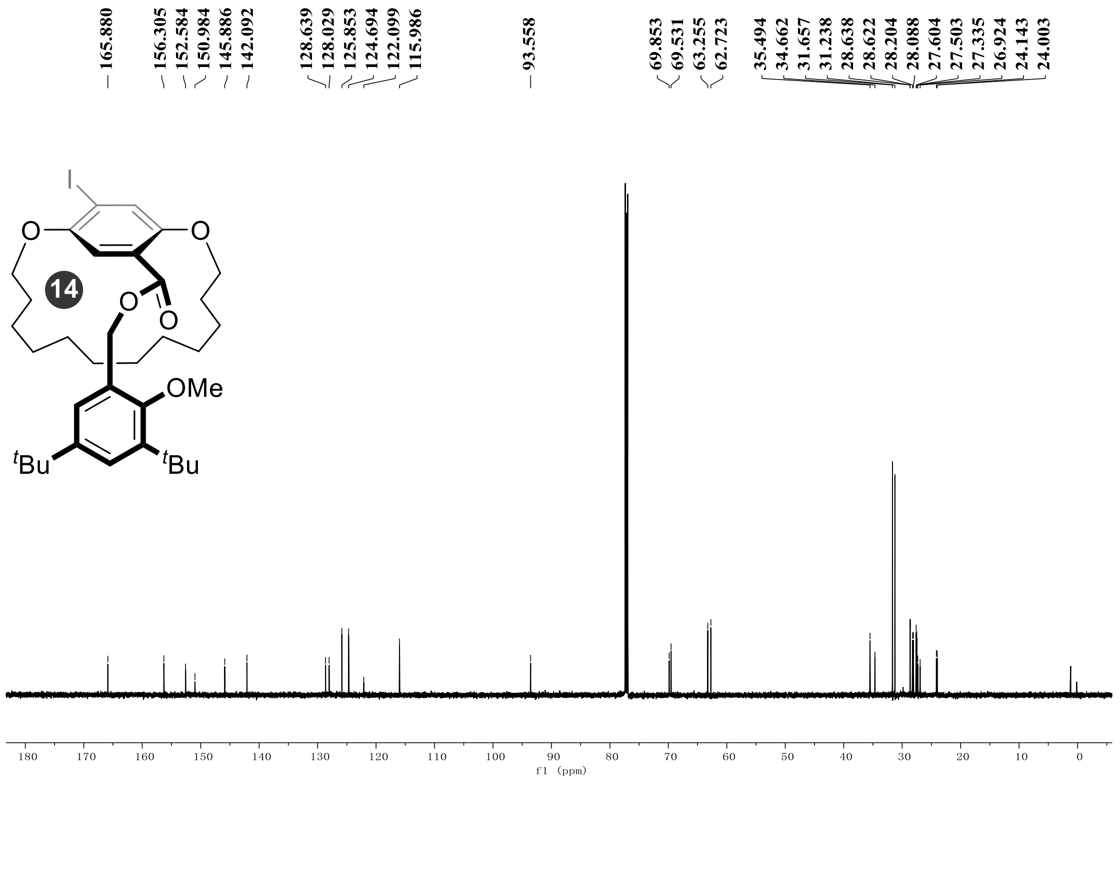

Supplementary Figure 40. ^{1}H NMR and ^{13}C NMR spectrum of compound of **3ke**

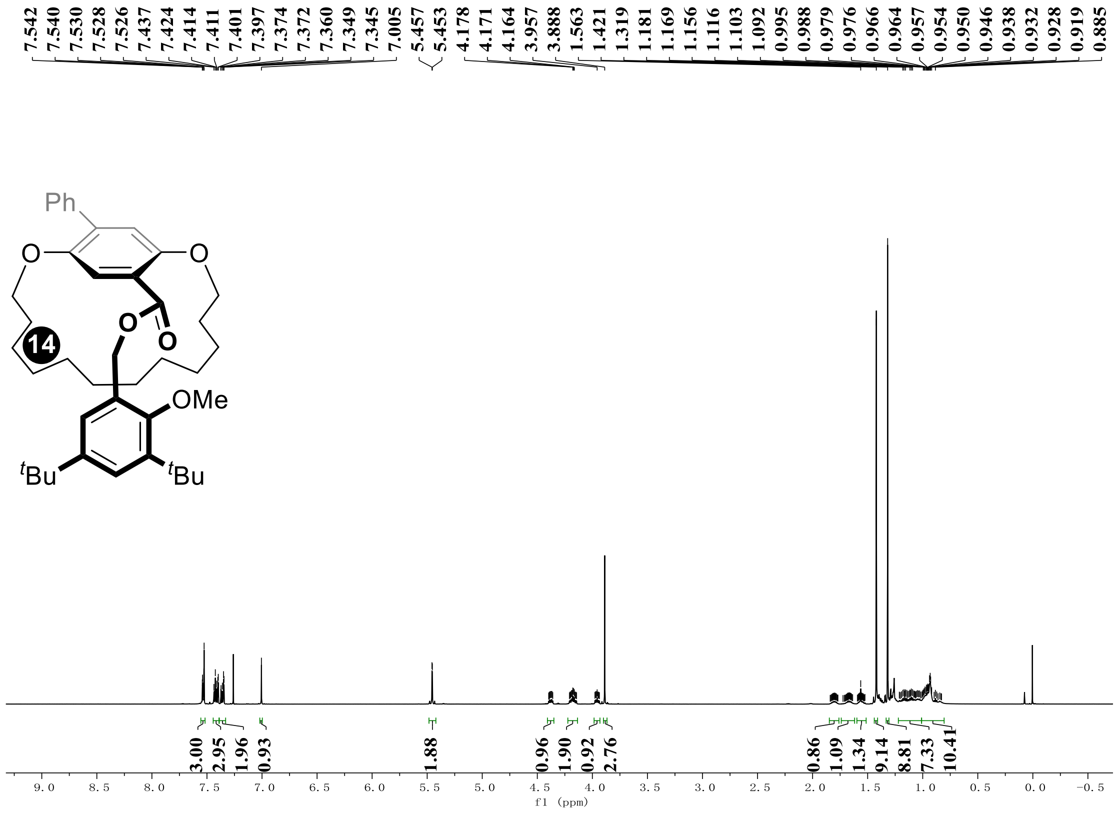

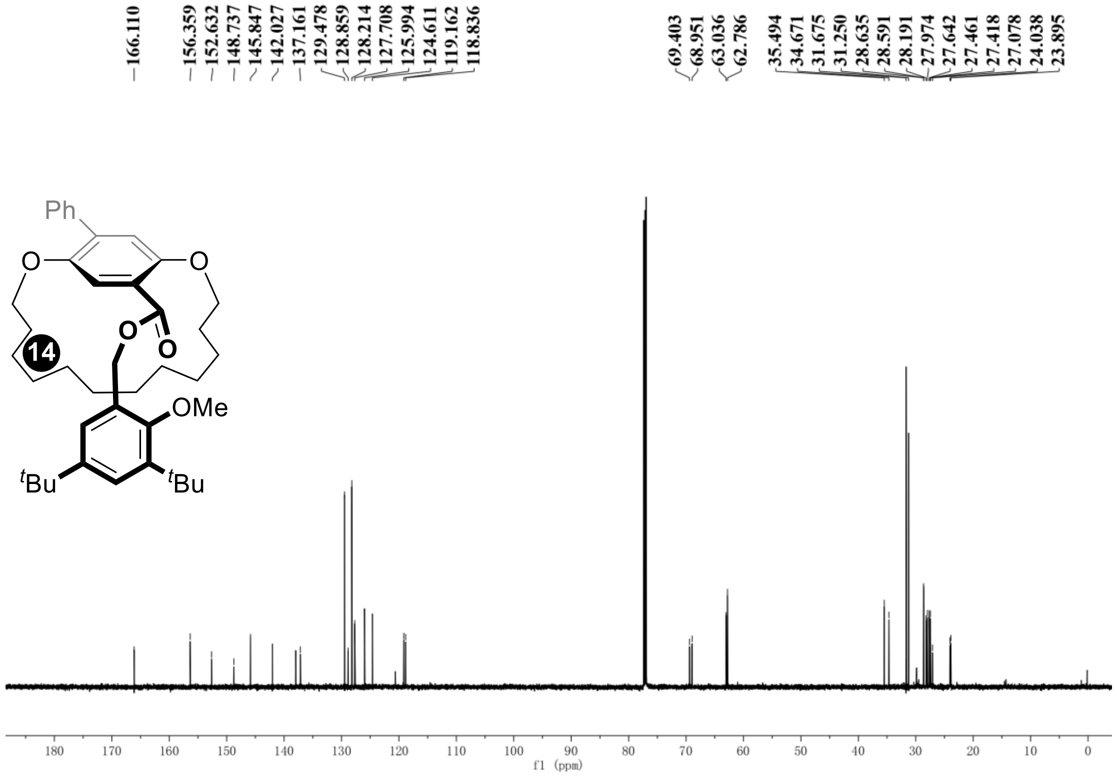

Supplementary Figure 41. ^{1}H NMR and ^{13}C NMR spectrum of compound of **3le**

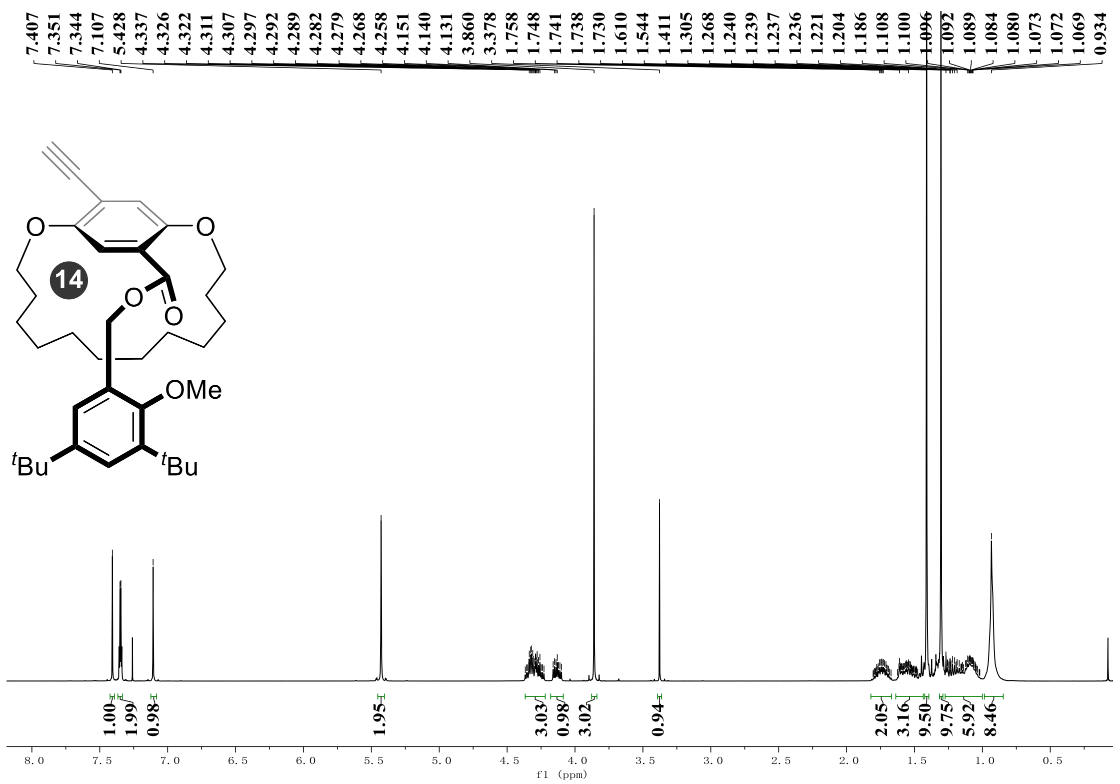

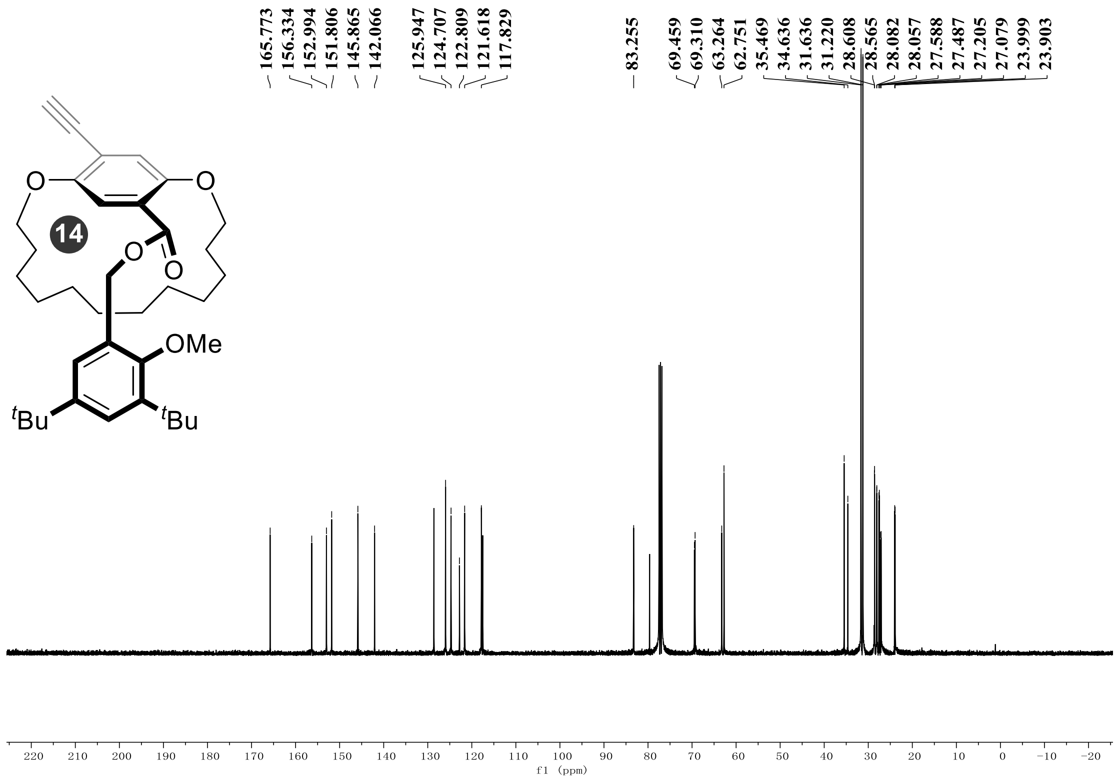

Supplementary Figure 42. ^{1}H NMR and ^{13}C NMR spectrum of compound of **5ae**

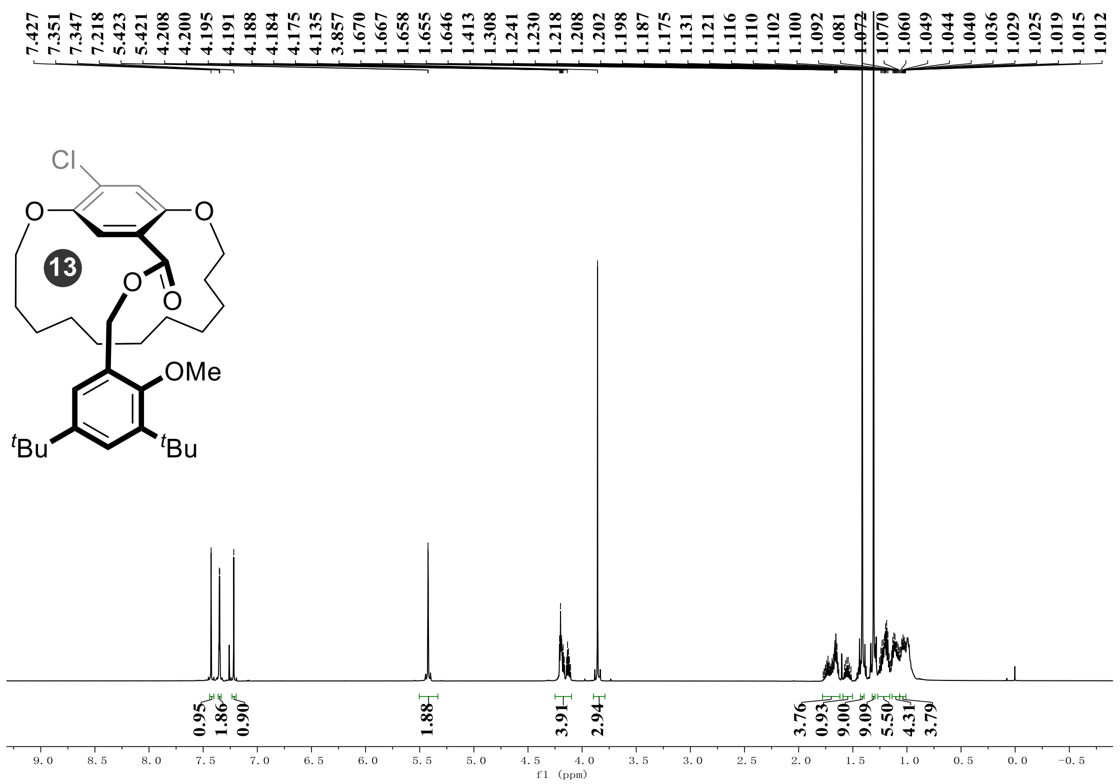

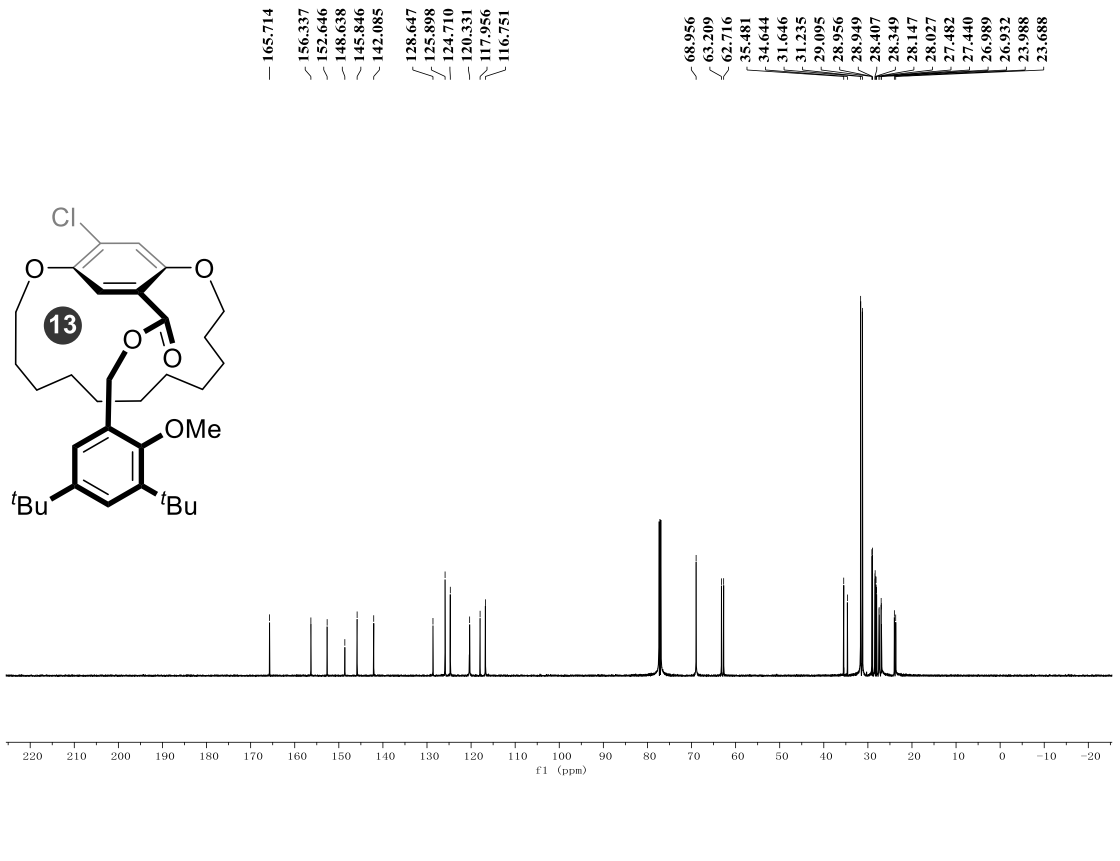

Supplementary Figure 43. ^{1}H NMR and ^{13}C NMR spectrum of compound of **5be**

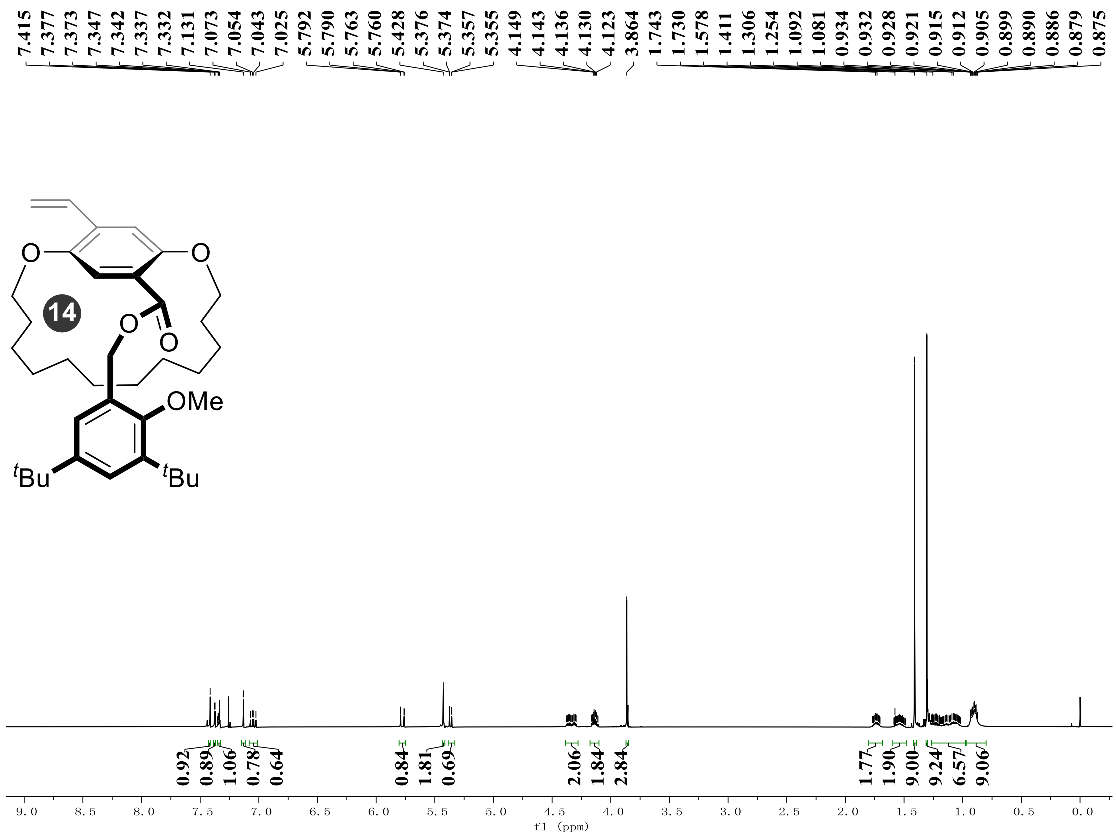

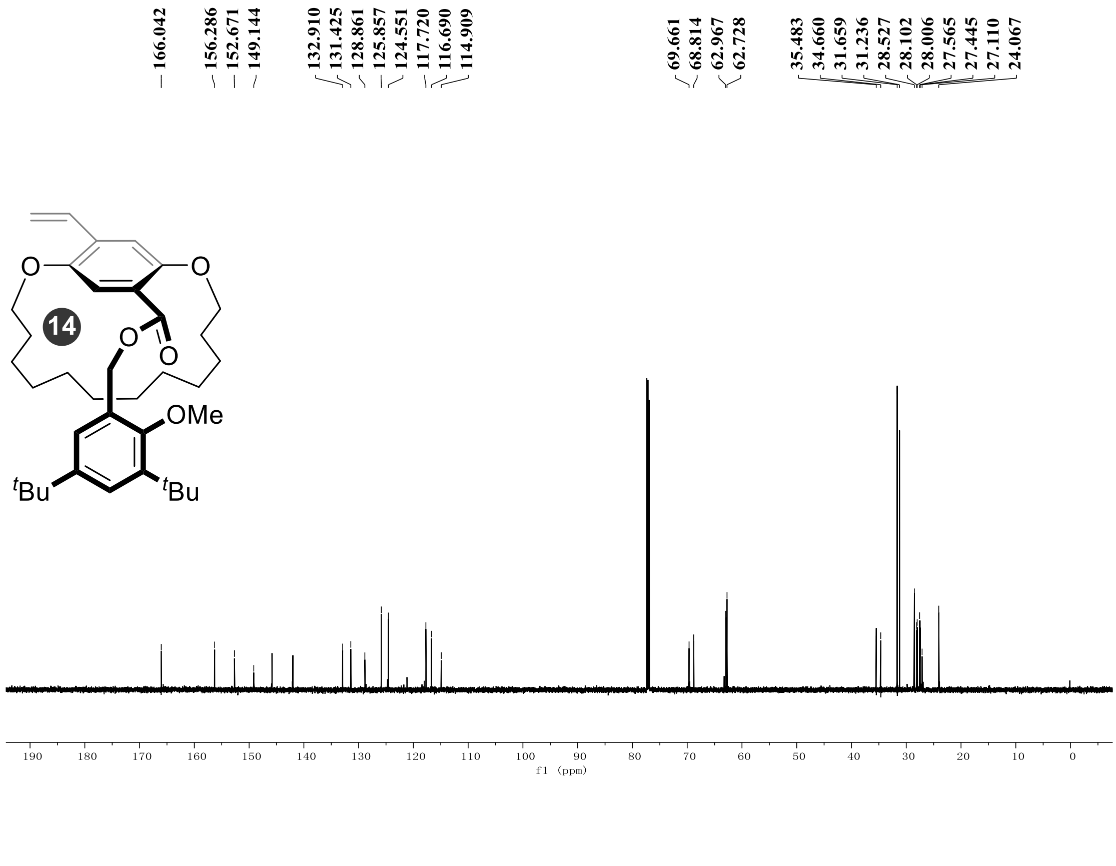

Supplementary Figure 44. ^{1}H NMR and ^{13}C NMR spectrum of compound of **5ce**

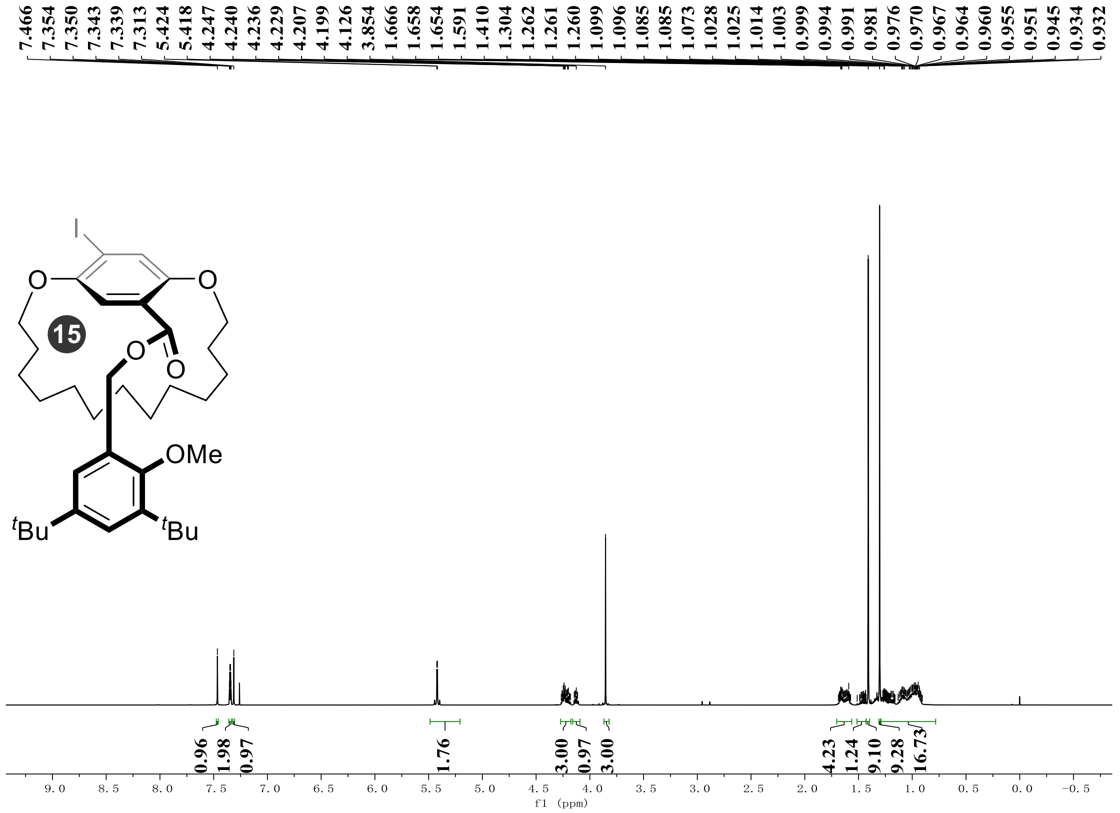
7.466 7.354 7.350 7.343 7.339 7.313 5.424 5.418 4.247 4.240 4.236 4.229 4.207 4.199 4.126 3.854 1.666 1.658 1.654 1.591 1.410 1.304 1.262 1.261 1.260 1.099 1.096 1.085 1.073 1.028 1.025 1.014 1.003 0.999 0.994 0.991 0.981 0.976 0.970 0.967 0.964 0.960 0.955 0.951 0.945 0.934 0.932
I
O
O
15
O
O
OMe
^{t}Bu
^{t}Bu
0.96 1.98 0.97 1.76 3.00 0.97 3.00 4.23 1.24 9.10 9.28 16.73
9.0 8.5 8.0 7.5 7.0 6.5 6.0 5.5 5.0 4.5 4.0 3.5 3.0 2.5 2.0 1.5 1.0 0.5 0.0 -0.5
f1 (ppm)

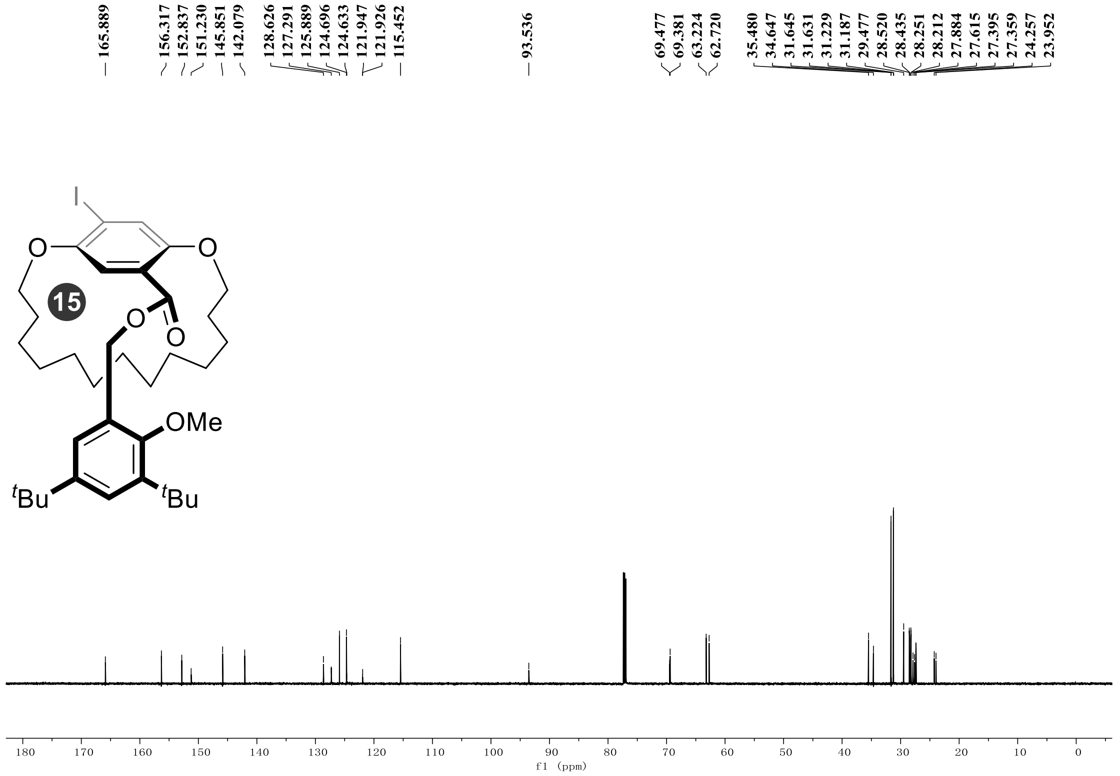
165.889 156.317 152.837 151.230 145.851 142.079 128.626 127.291 125.889 124.696 124.633 121.947 121.926 115.452 93.536 69.477 69.381 63.224 62.720 35.480 34.647 31.645 31.631 31.229 31.187 29.477 28.520 28.435 28.251 28.212 27.884 27.615 27.395 27.359 24.257 23.952
I
O
O
15
O
O
OMe
^{t}Bu
^{t}Bu
180 170 160 150 140 130 120 110 100 90 80 70 60 50 40 30 20 10 0
f1 (ppm)

Supplementary Figure 45. ^{1}H NMR and ^{13}C NMR spectrum of compound of **5de**

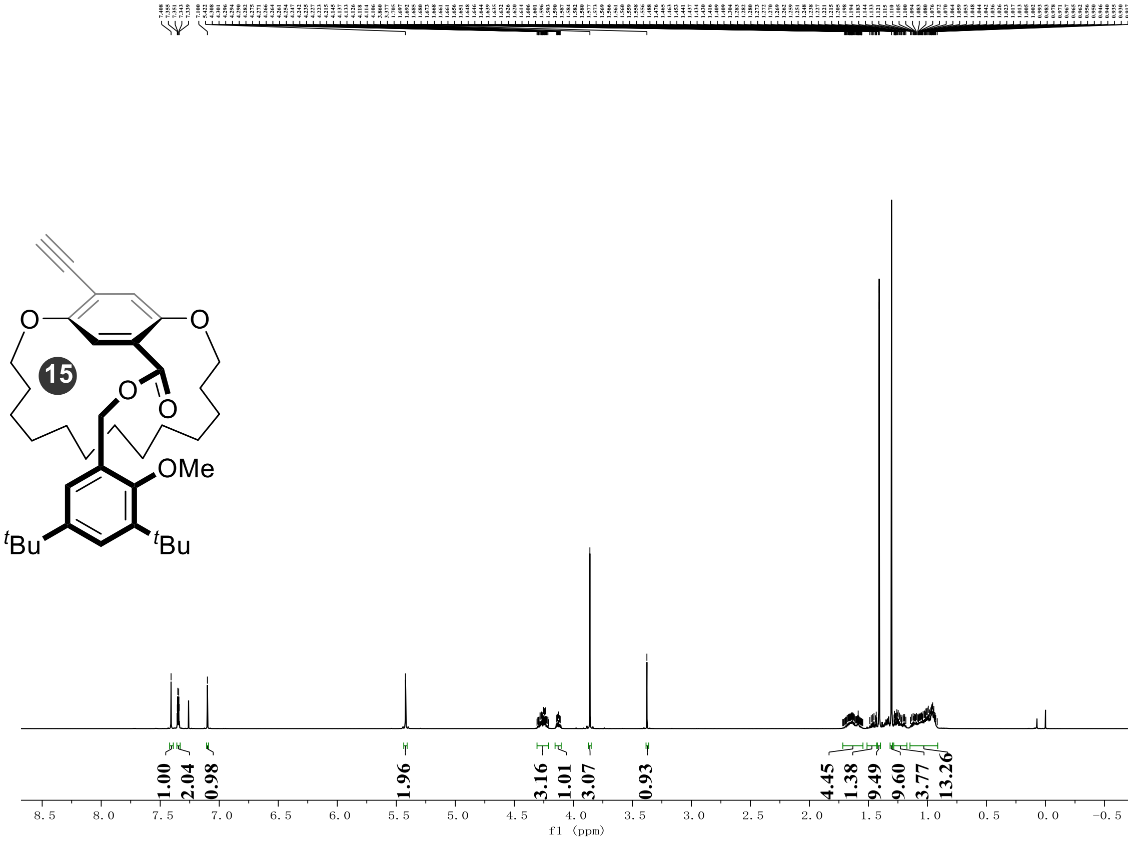

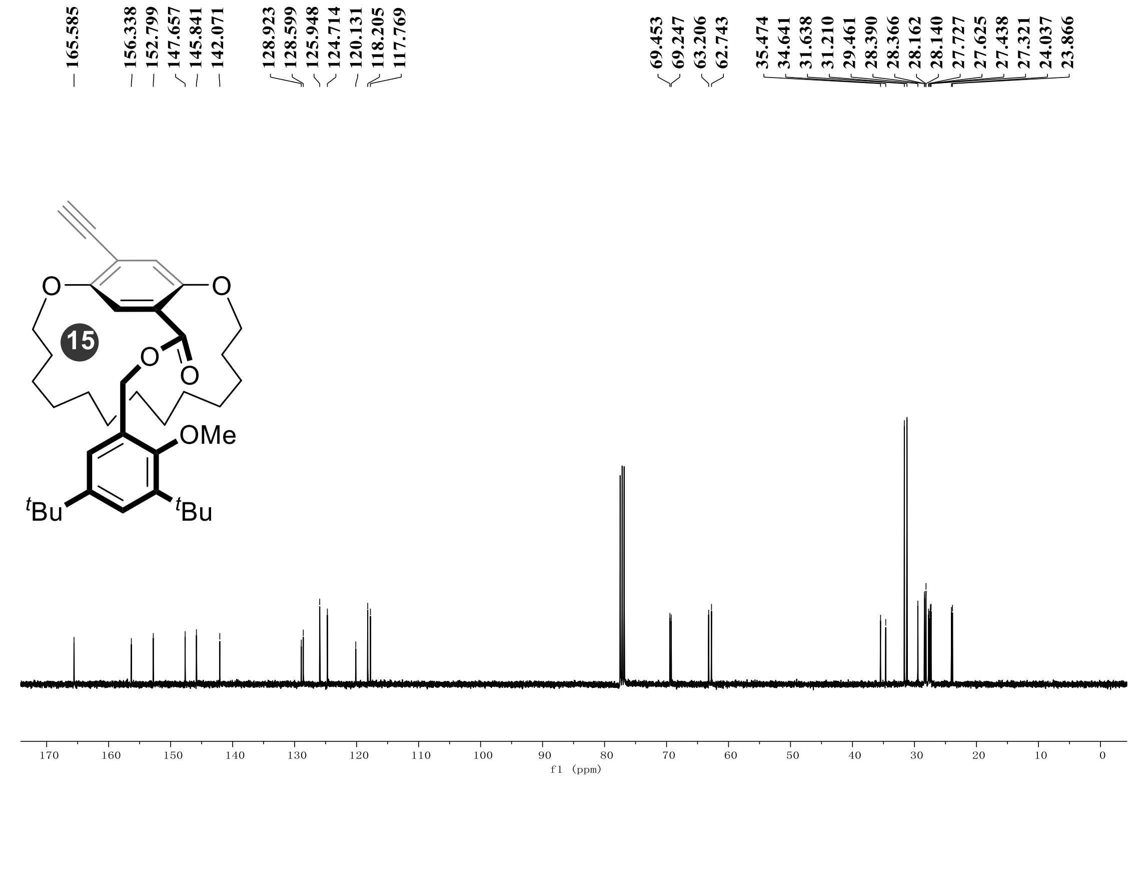

Supplementary Figure 46. ^{1}H NMR and ^{13}C NMR spectrum of compound of **5ee**

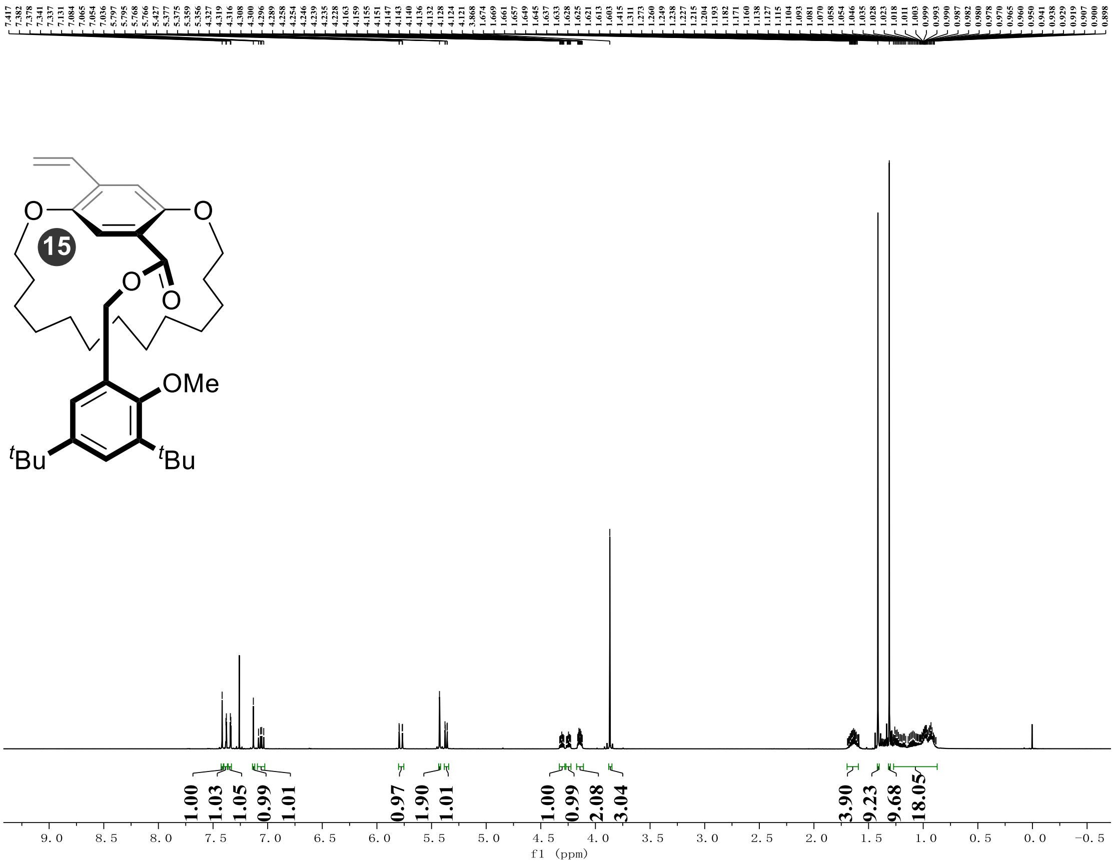

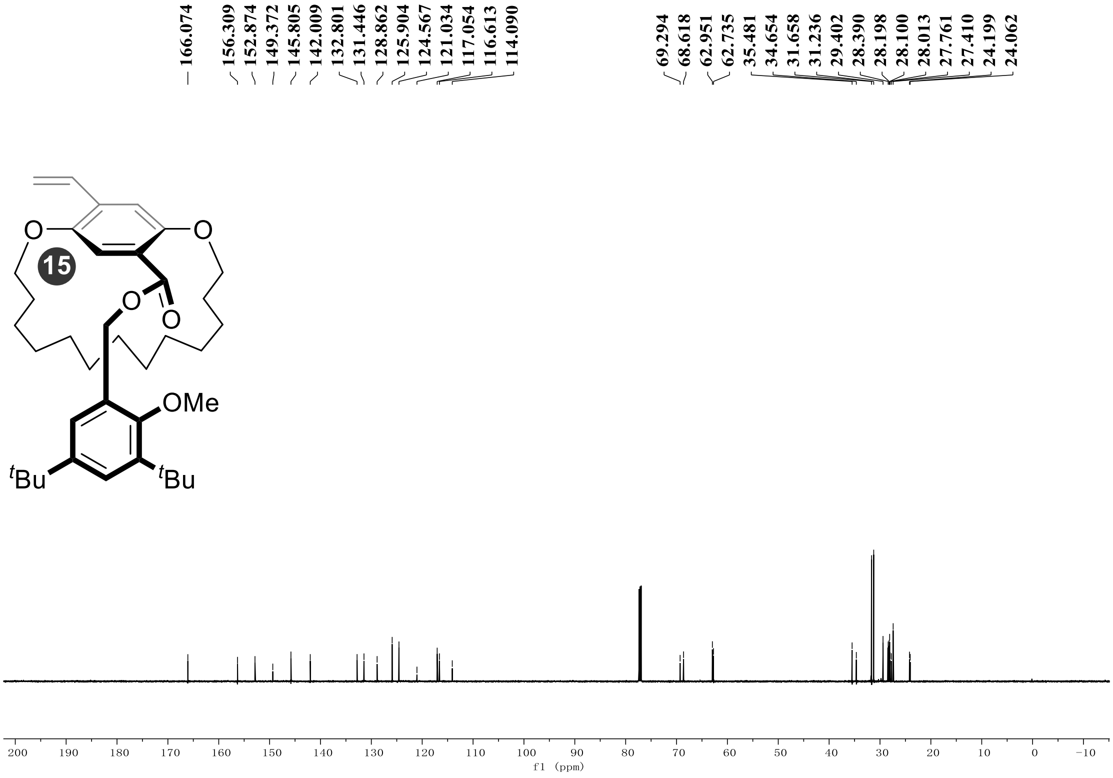

Supplementary Figure 47. ^{1}H NMR and ^{13}C NMR spectrum of compound of **5fe**

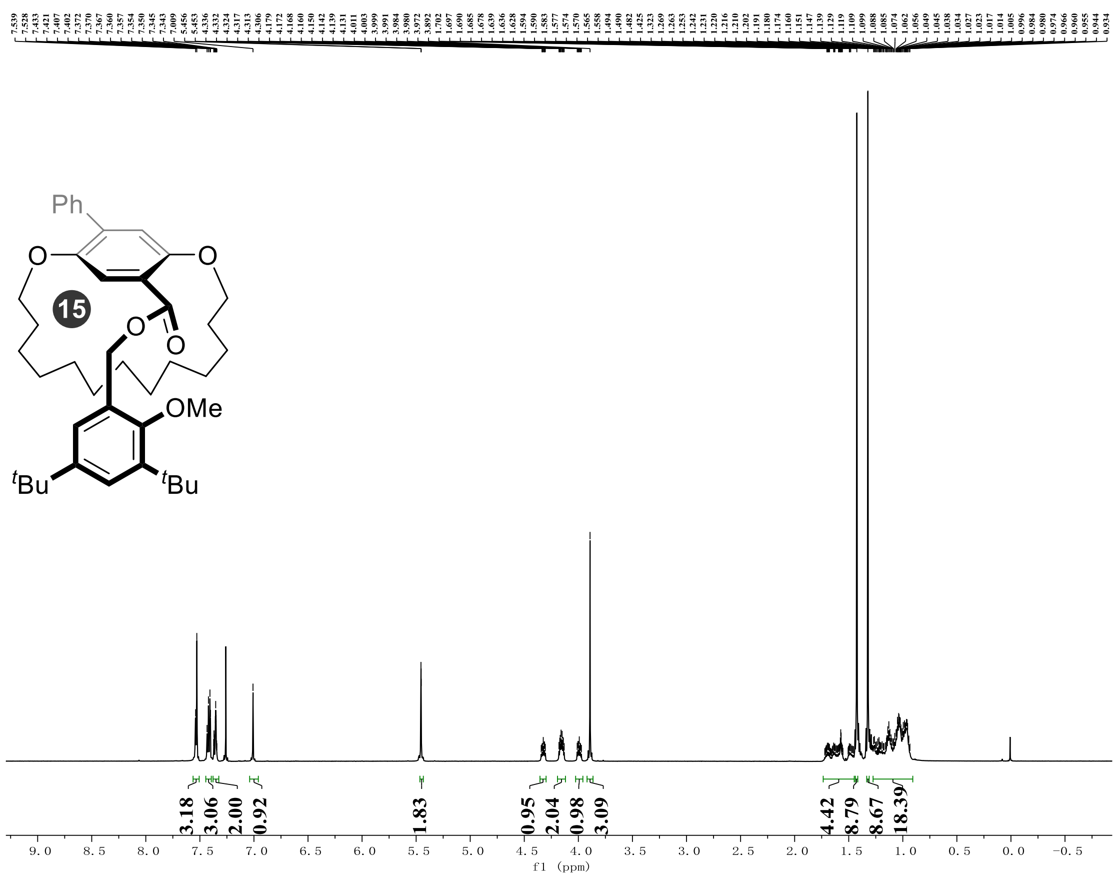

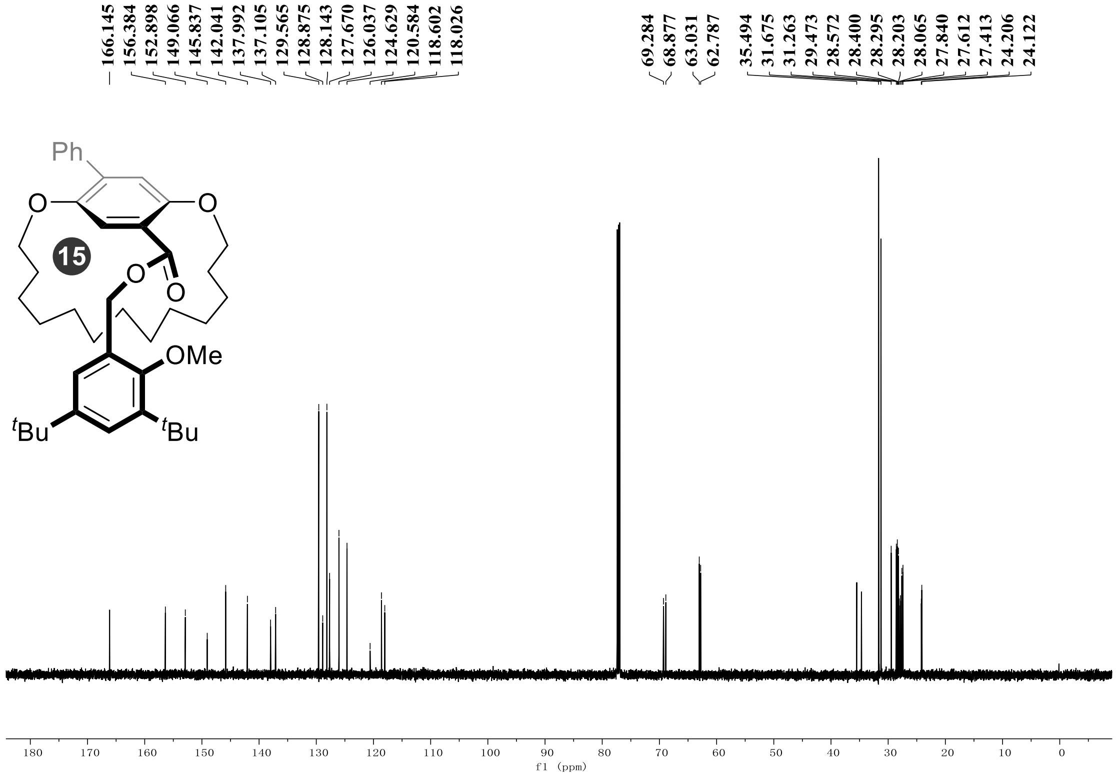

Supplementary Figure 48. ^{1}H NMR and ^{13}C NMR spectrum of compound of **5ge**

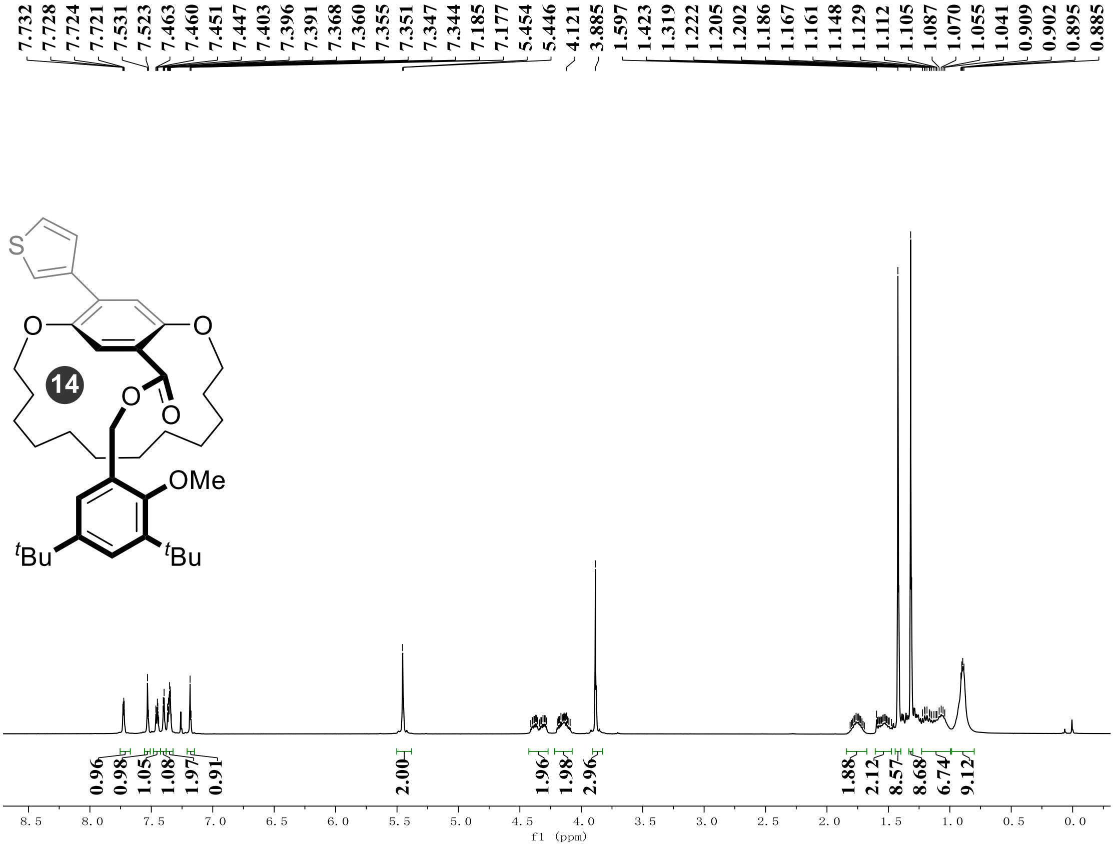

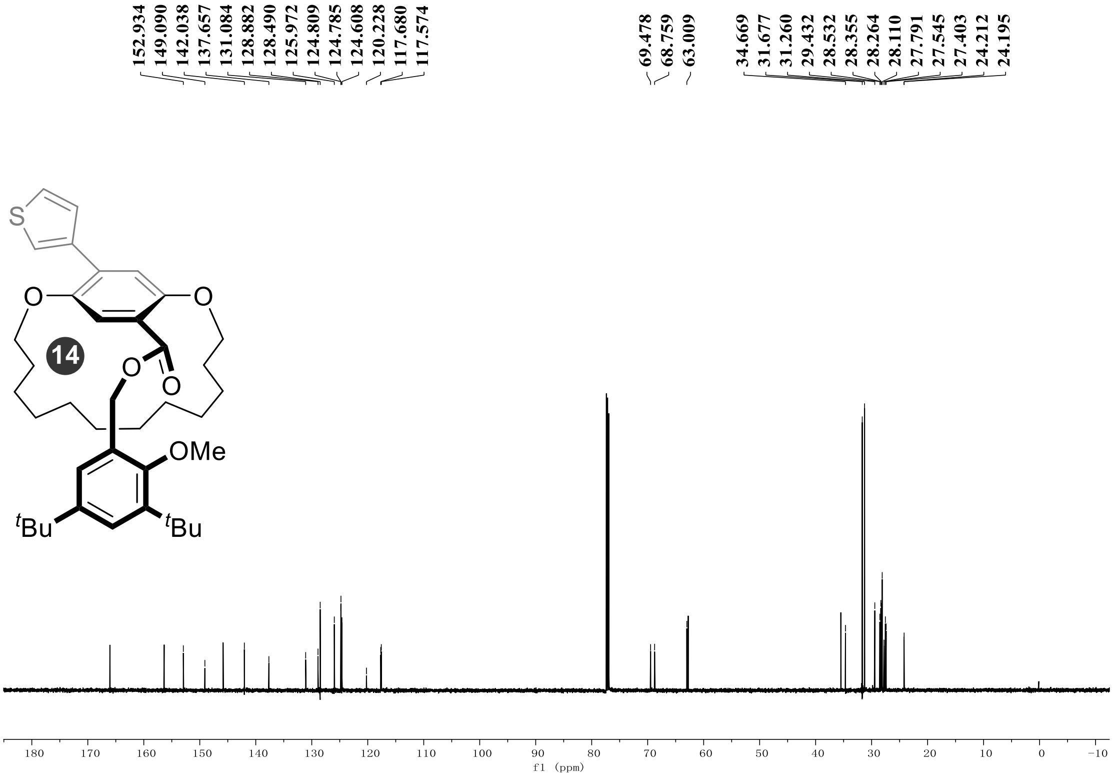

Supplementary Figure 49. ^{1}H NMR and ^{13}C NMR spectrum of compound of **5he**

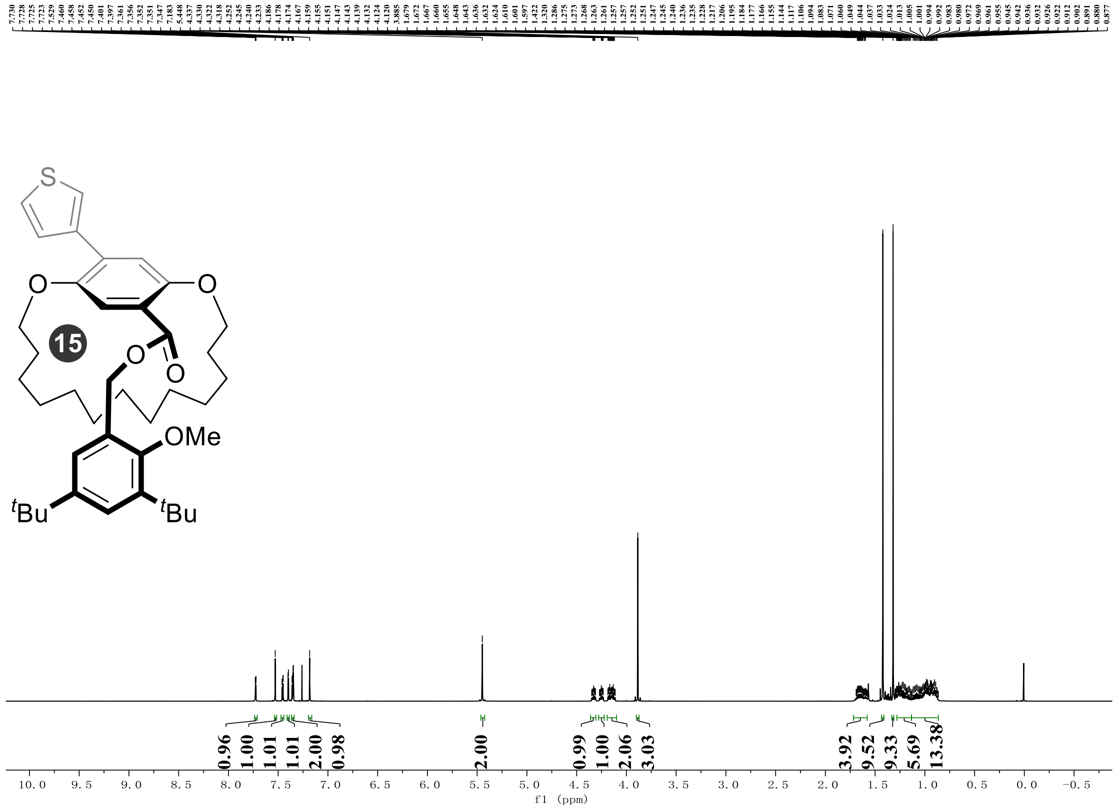

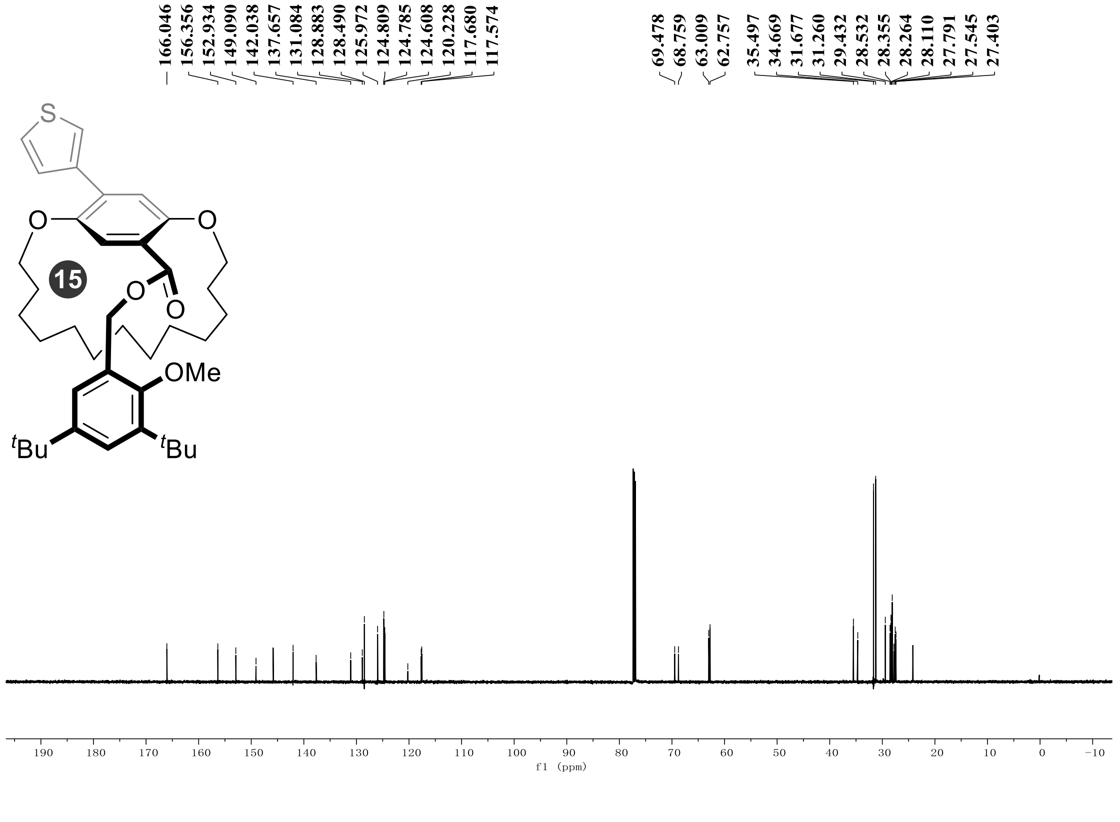

Supplementary Figure 50. ^{1}H NMR and ^{13}C NMR spectrum of compound of **5ie**

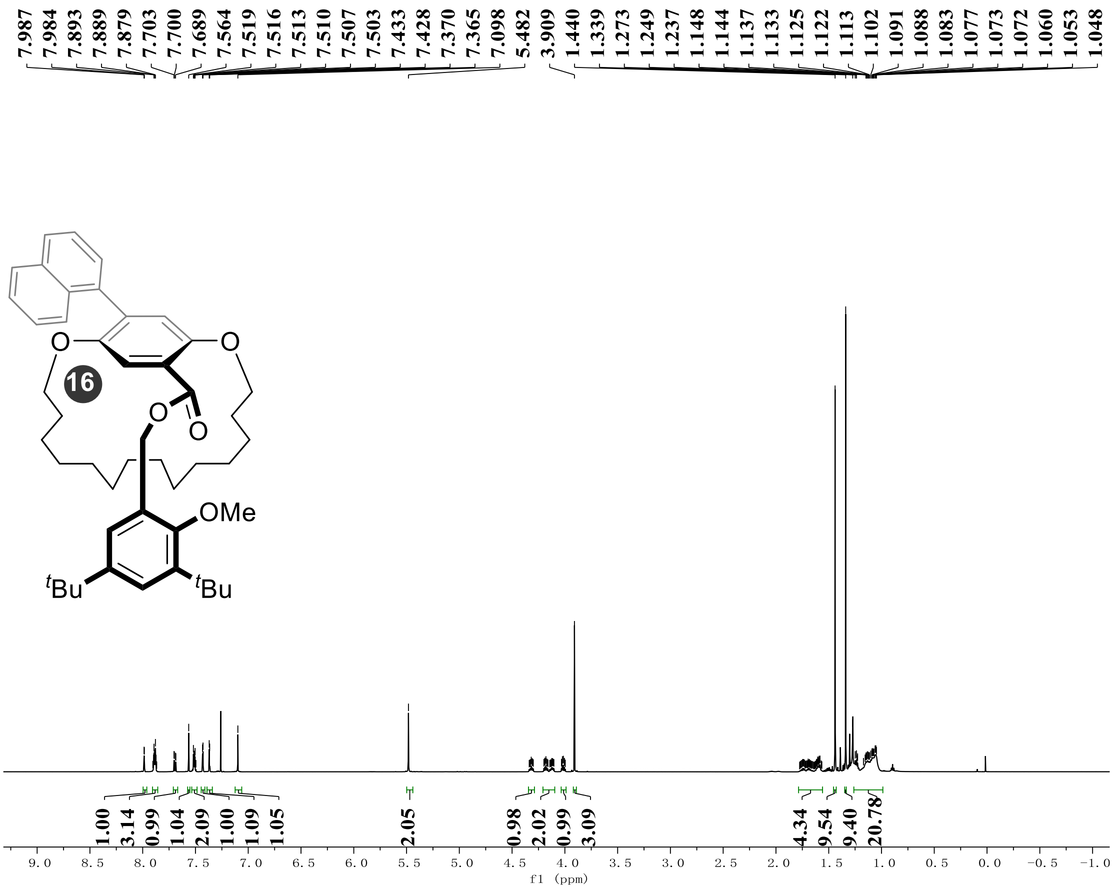

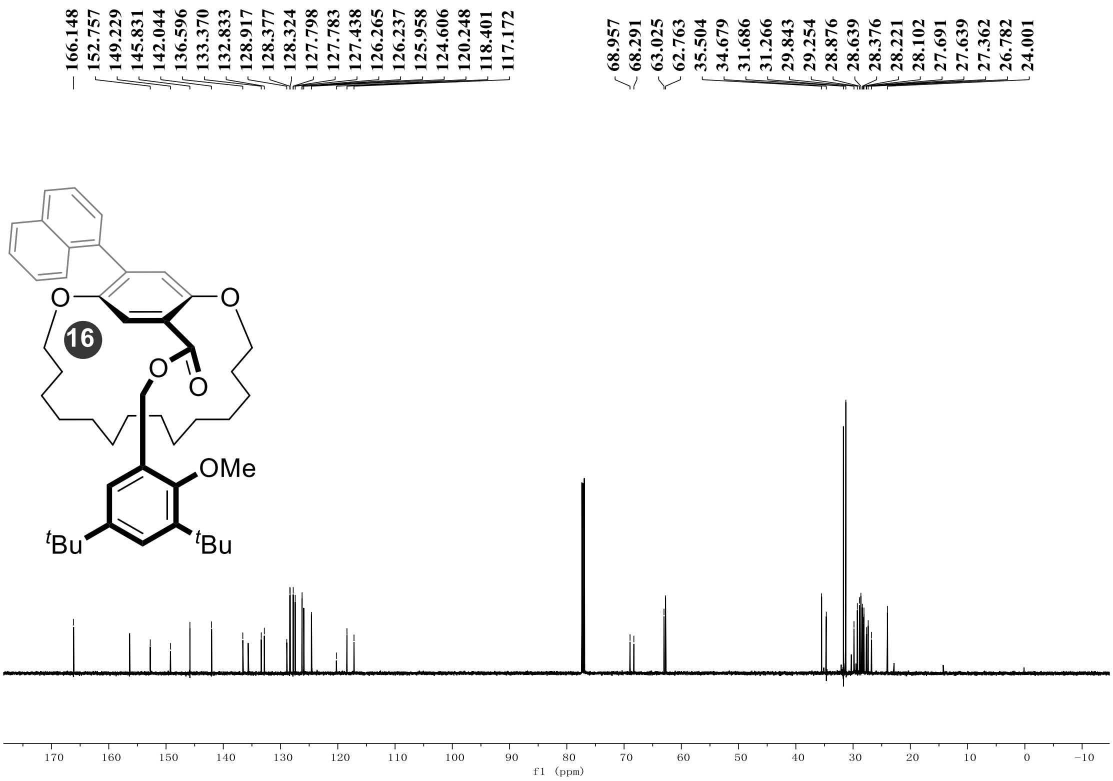

Supplementary Figure 51. ^{1}H NMR and ^{13}C NMR spectrum of compound of **5je**

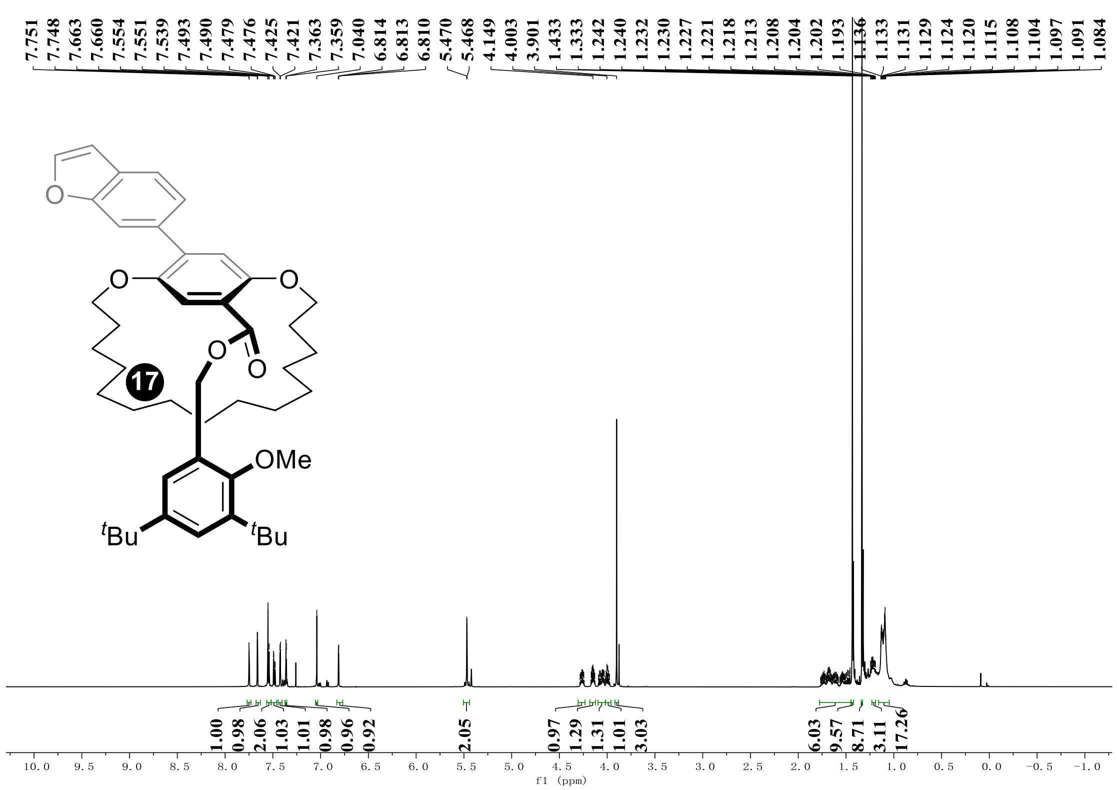

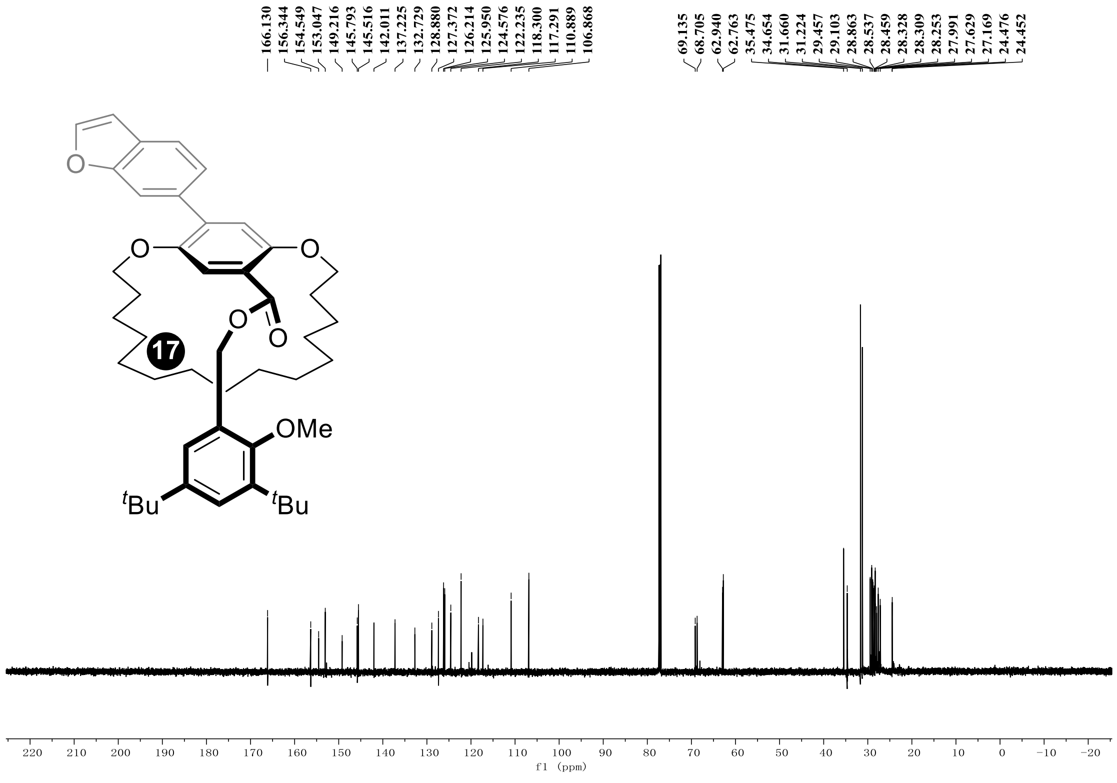

Supplementary Figure 52. ^{1}H NMR and ^{13}C NMR spectrum of compound of **5ke**

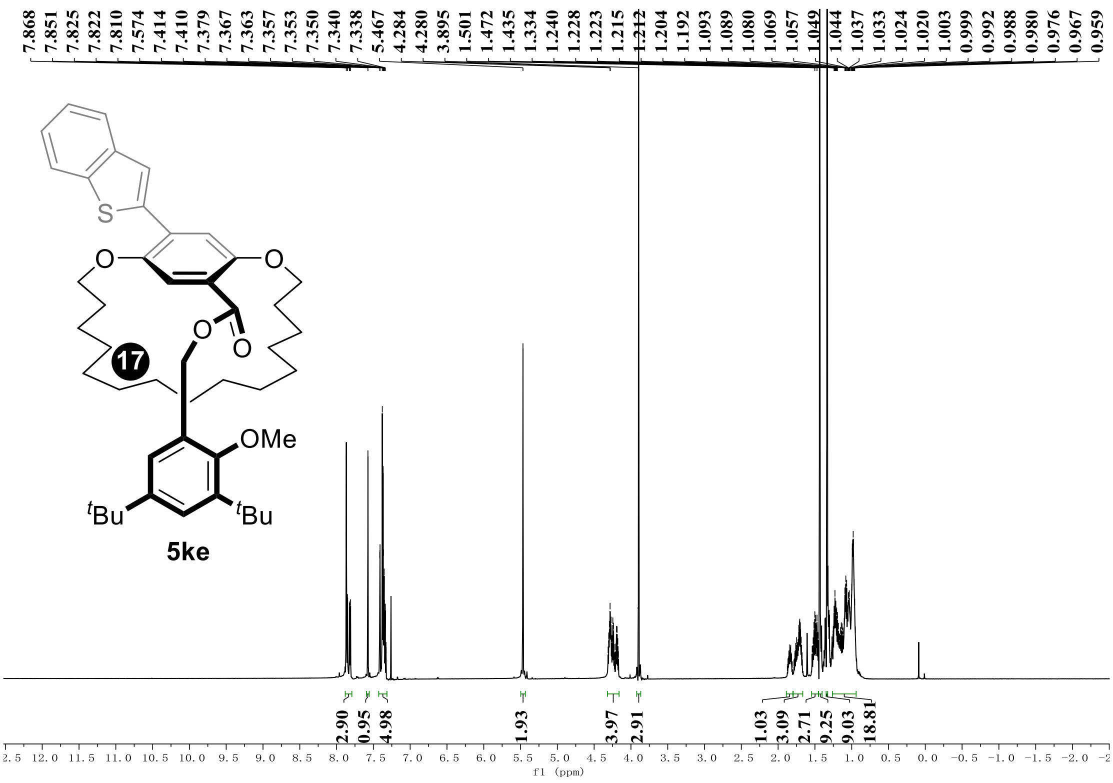

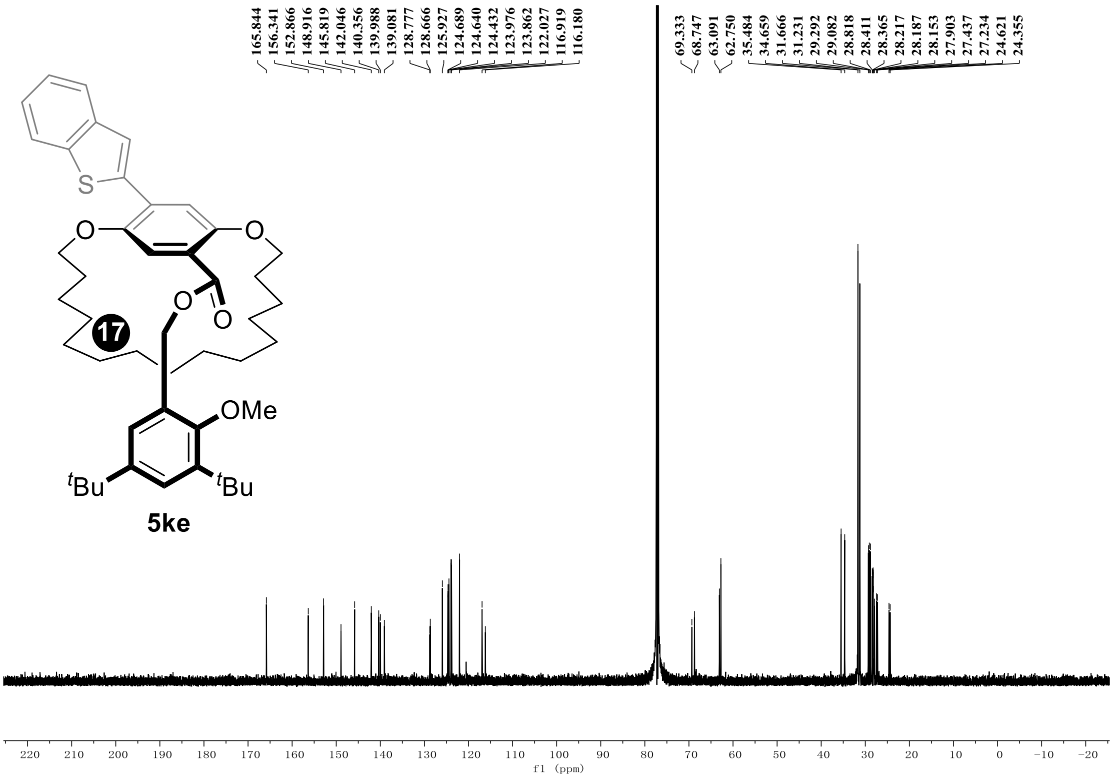

Supplementary Figure 53. ^{1}**H NMR and** ^{13}**C NMR** spectrum of compound of **5le**

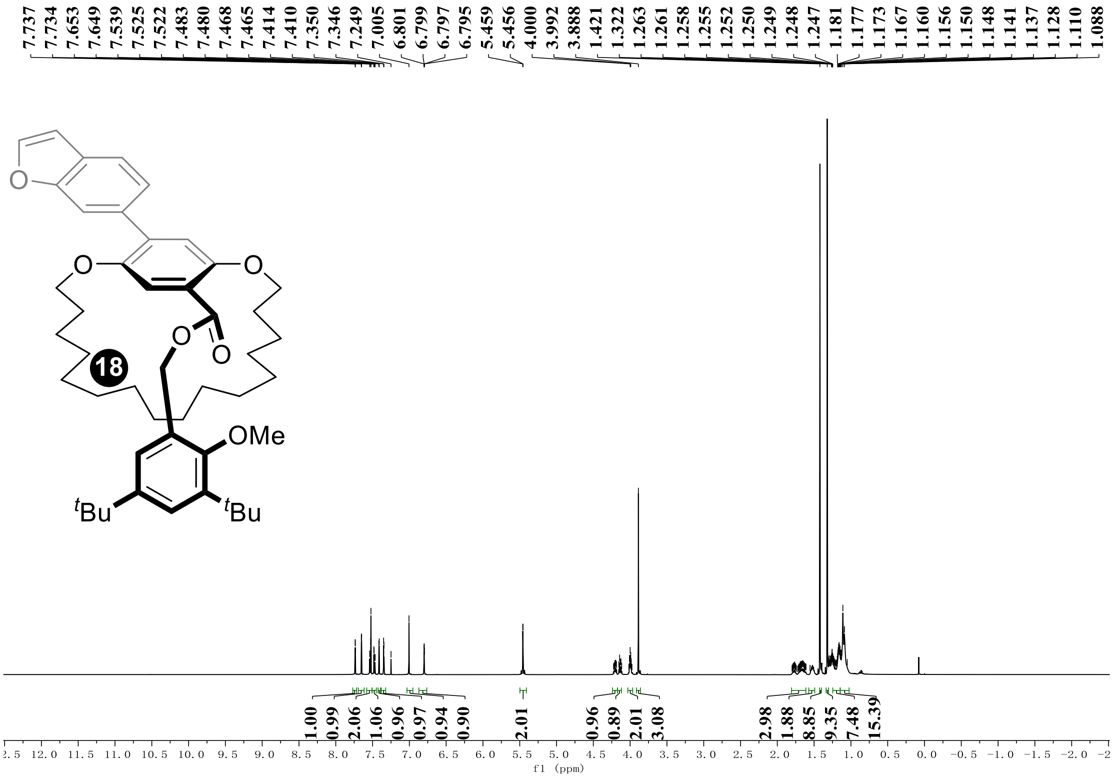

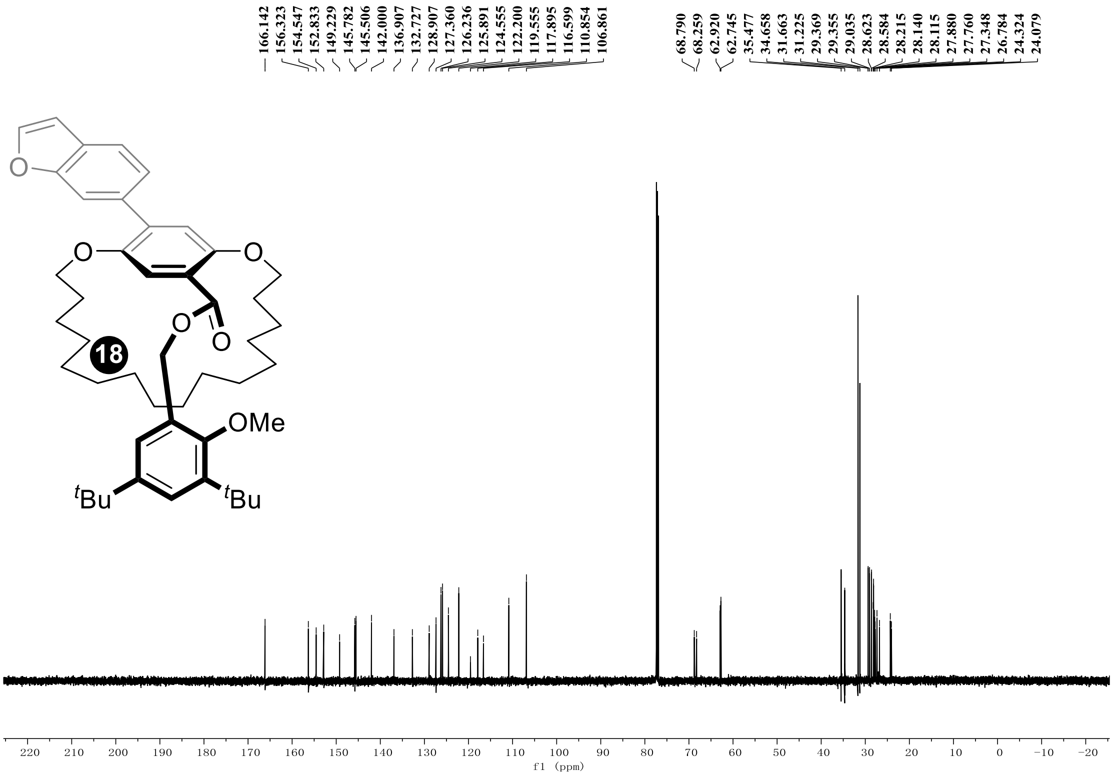

Supplementary Figure 54. ^{1}**H NMR and** ^{13}**C NMR** spectrum of compound of **5me**

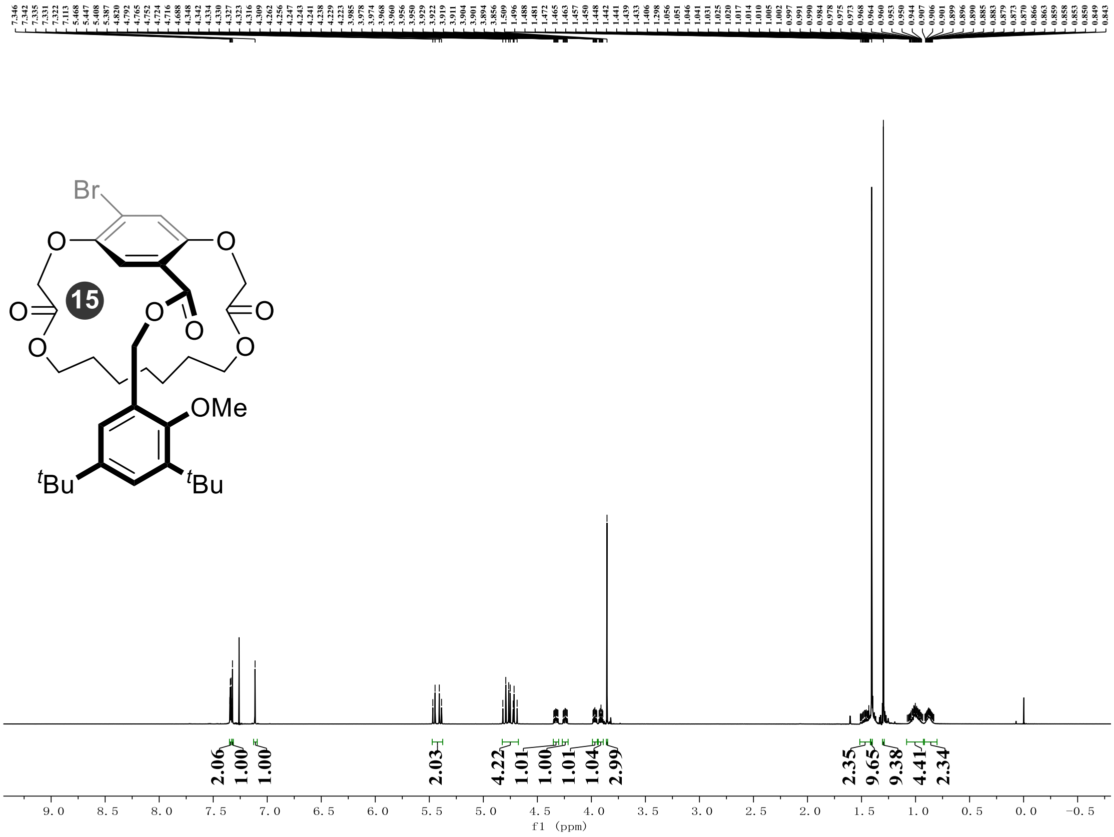

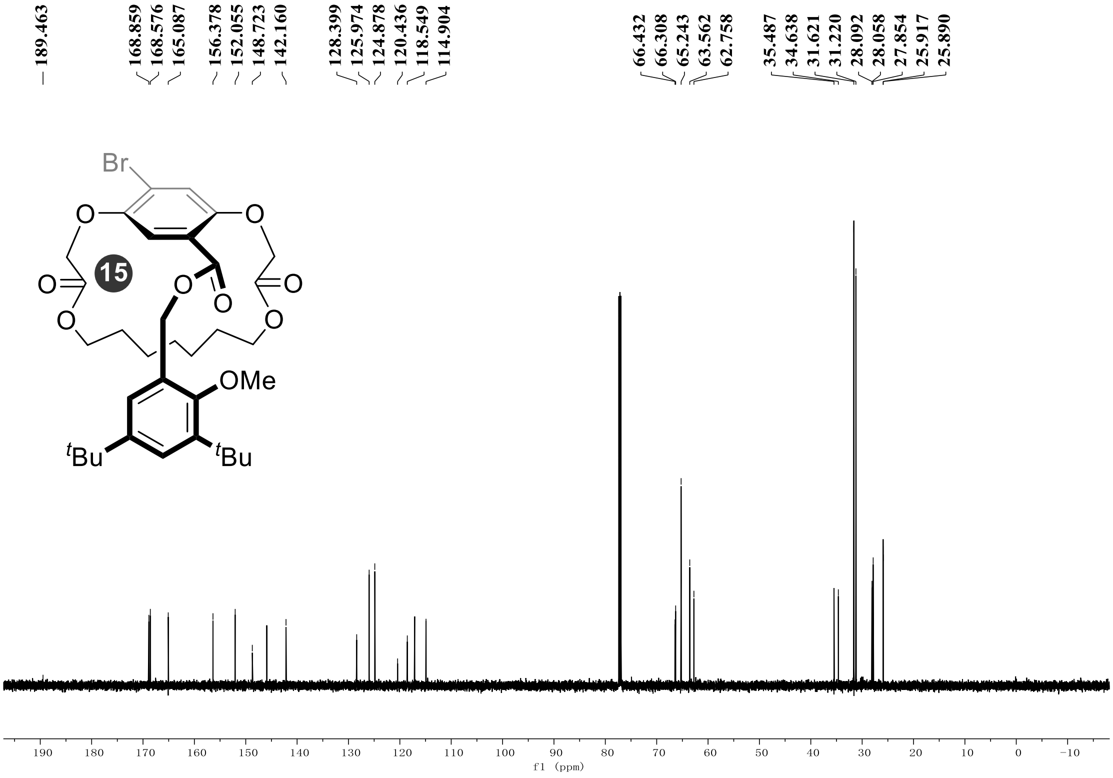

Supplementary Figure 55. ^{1}H NMR and ^{13}C NMR spectrum of compound of **5ne**

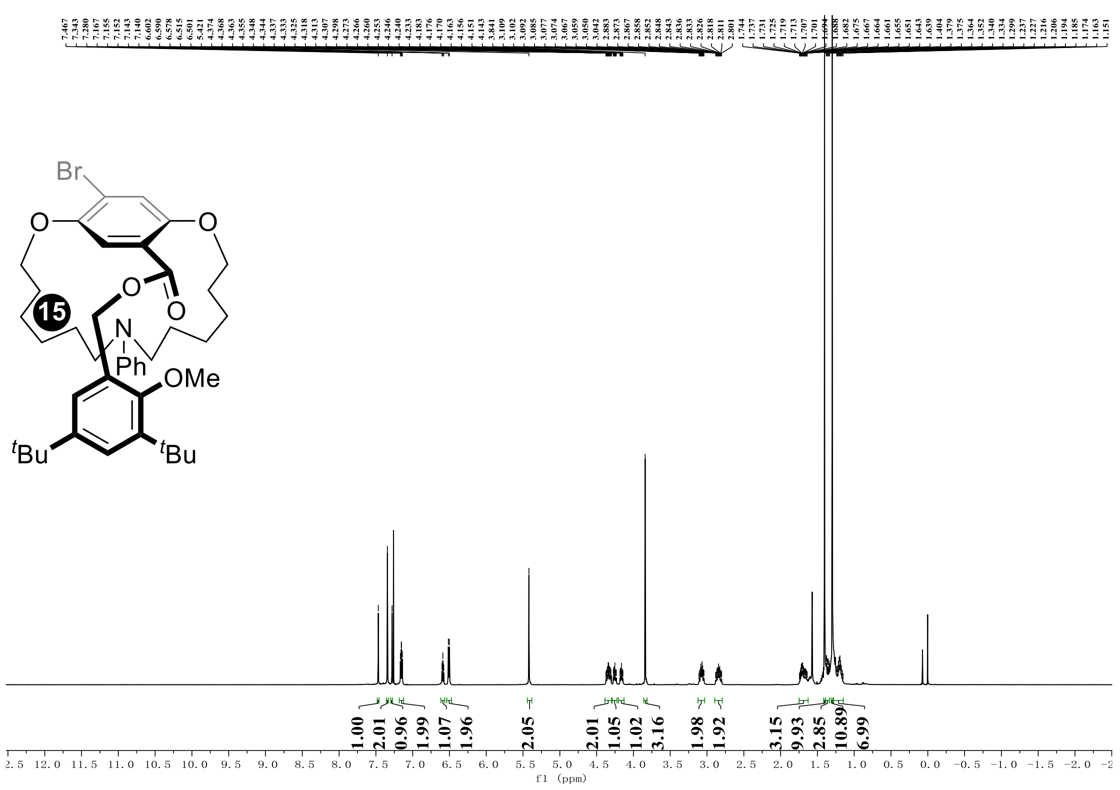

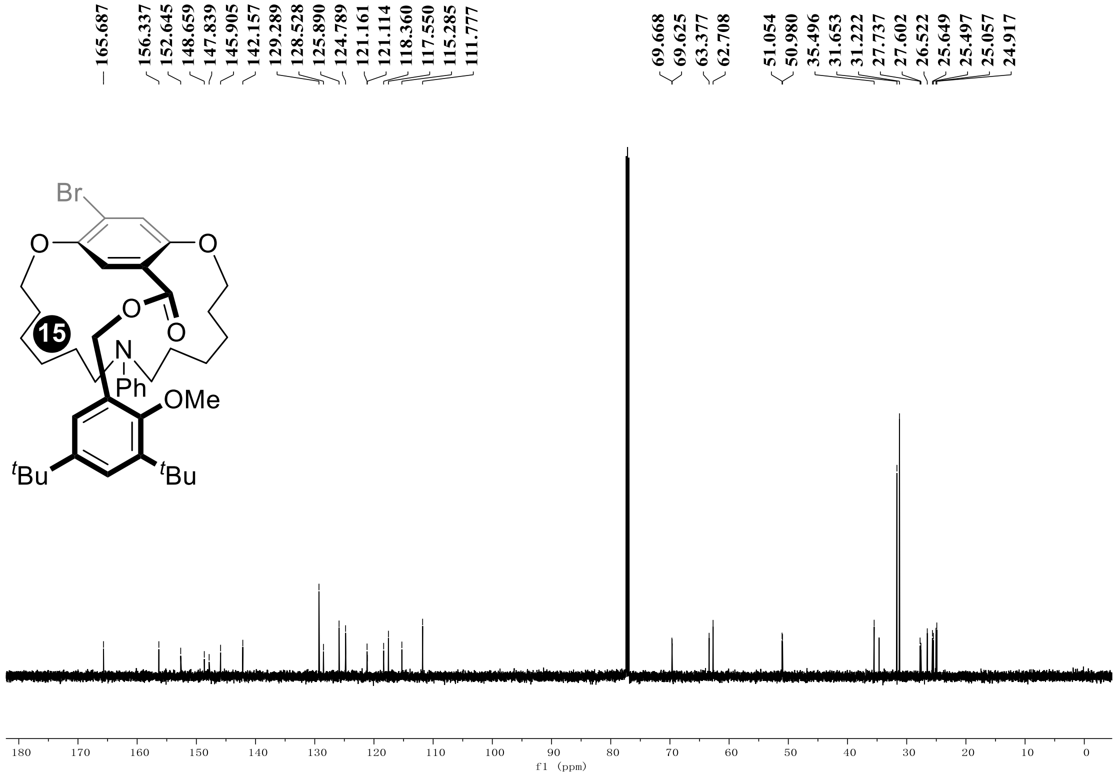

Supplementary Figure 56. ^{1}H NMR and ^{13}C NMR spectrum of compound of **5oe**

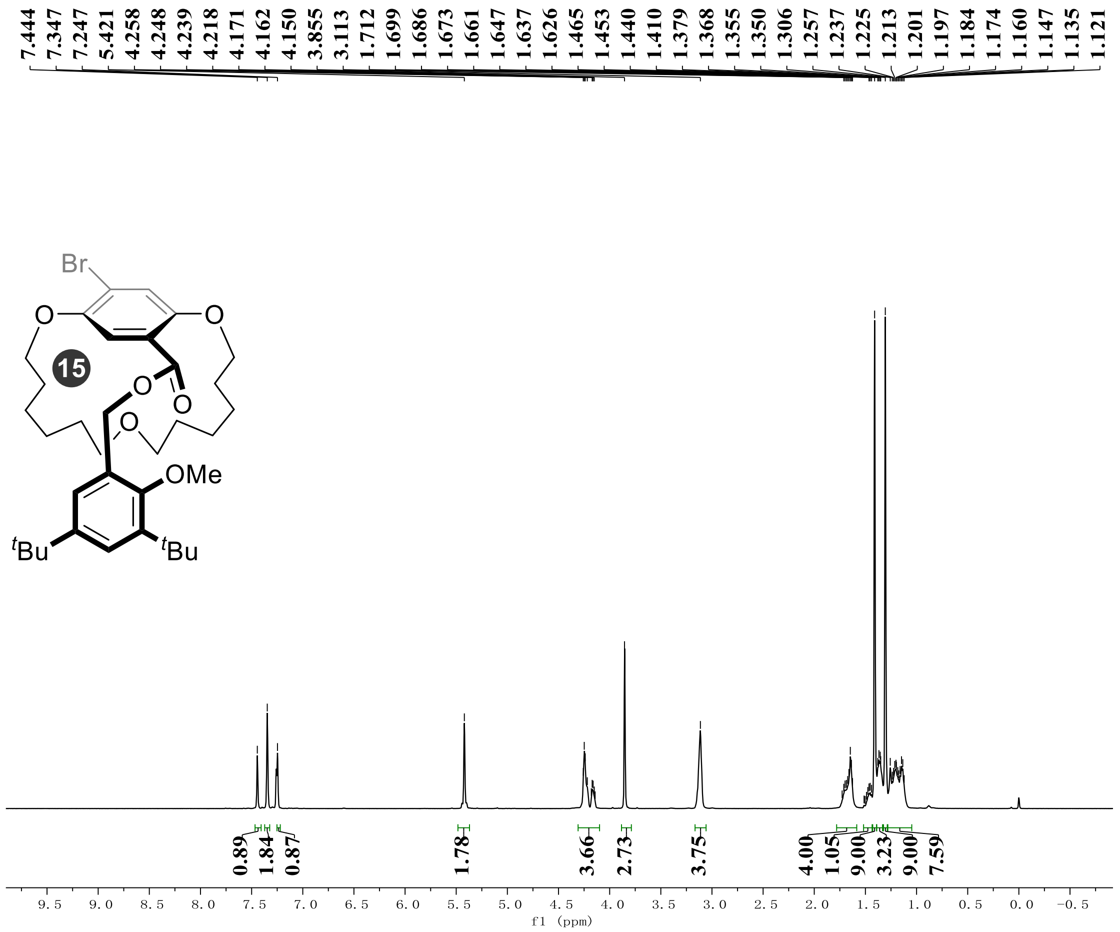

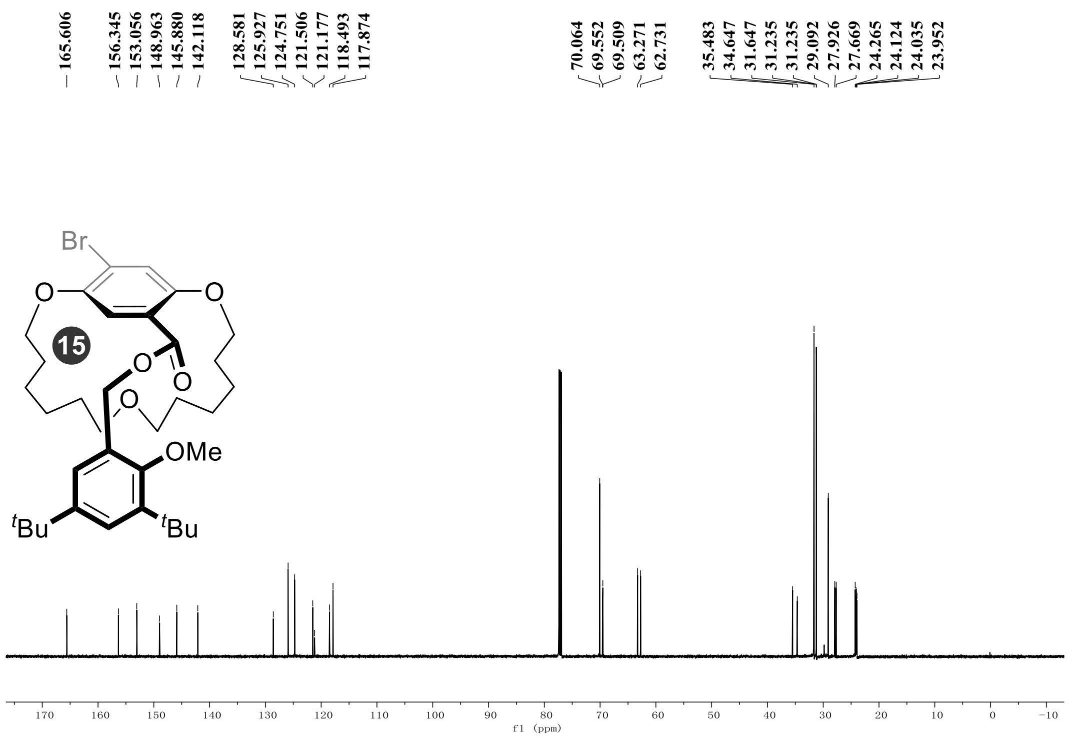

Supplementary Figure 57. ^{1}**H NMR and** ^{13}**C NMR** spectrum of compound of **6**

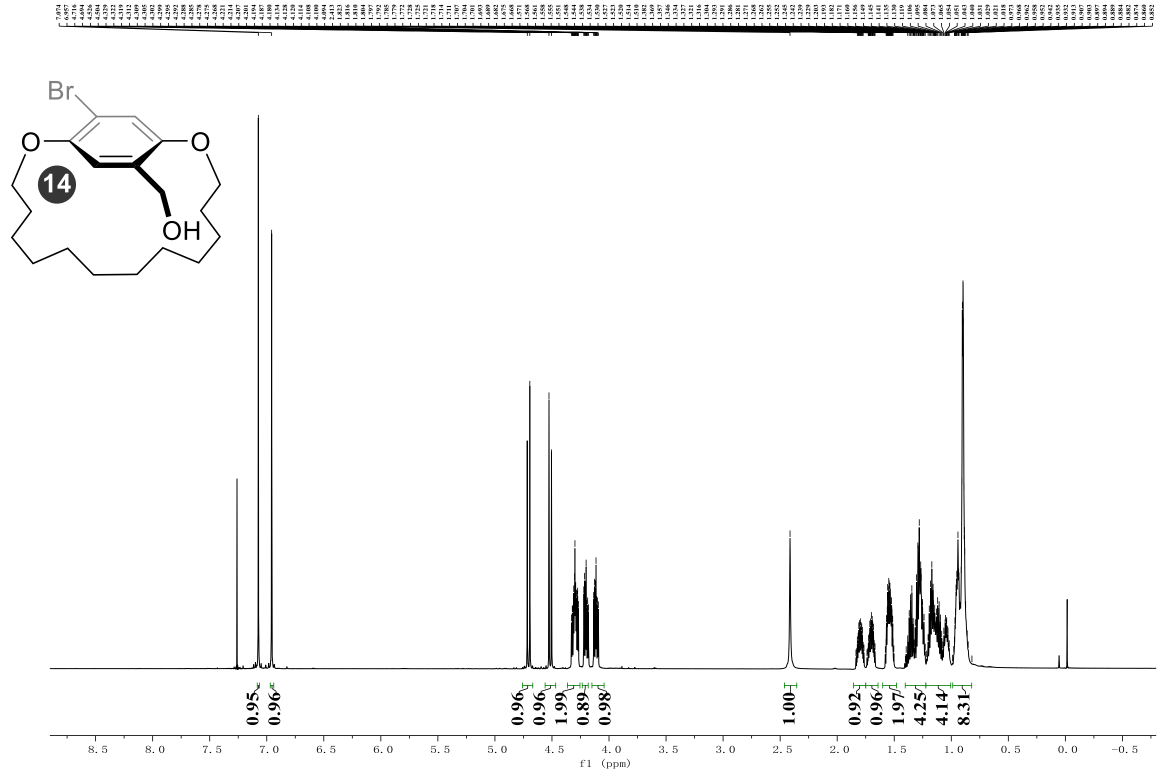

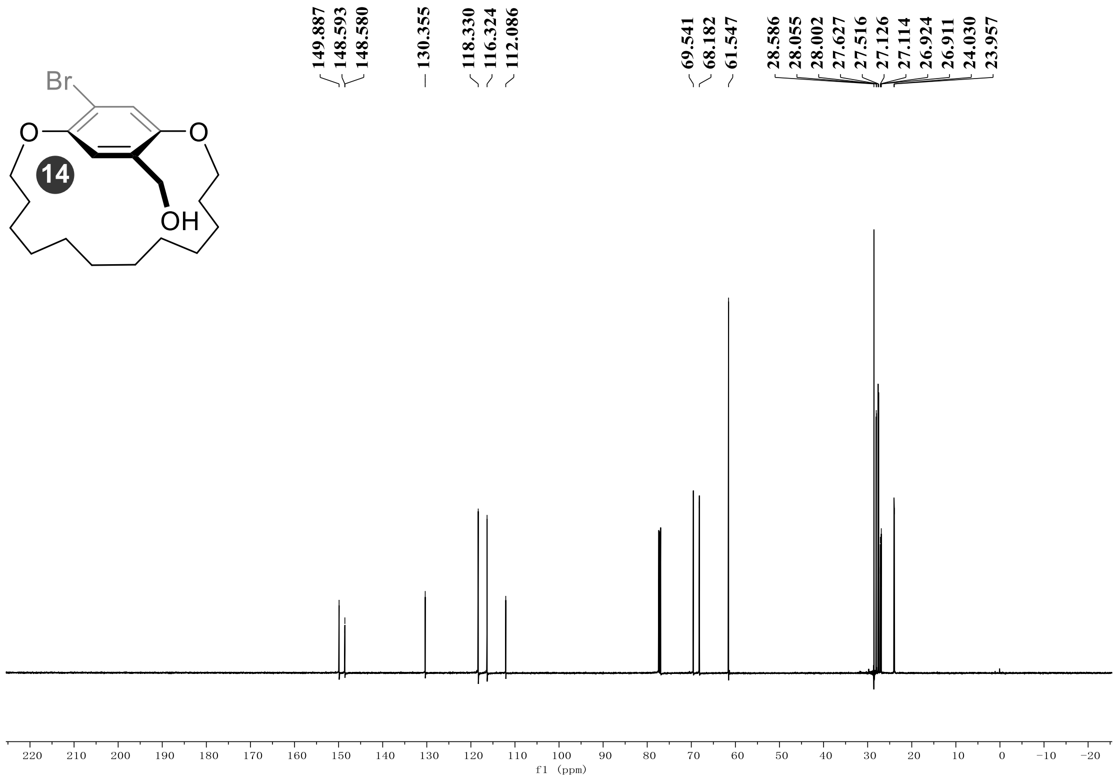

Supplementary Figure 58. ^{1}**H NMR and** ^{13}**C NMR** spectrum of compound of **7**

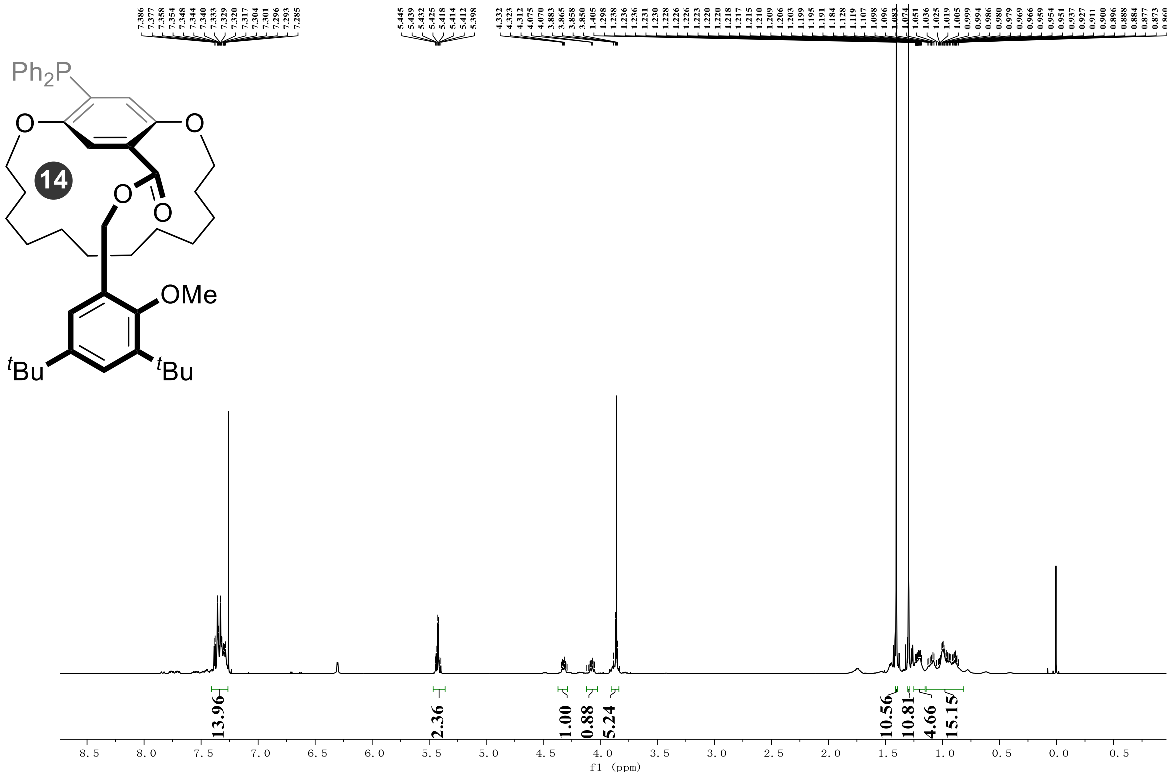

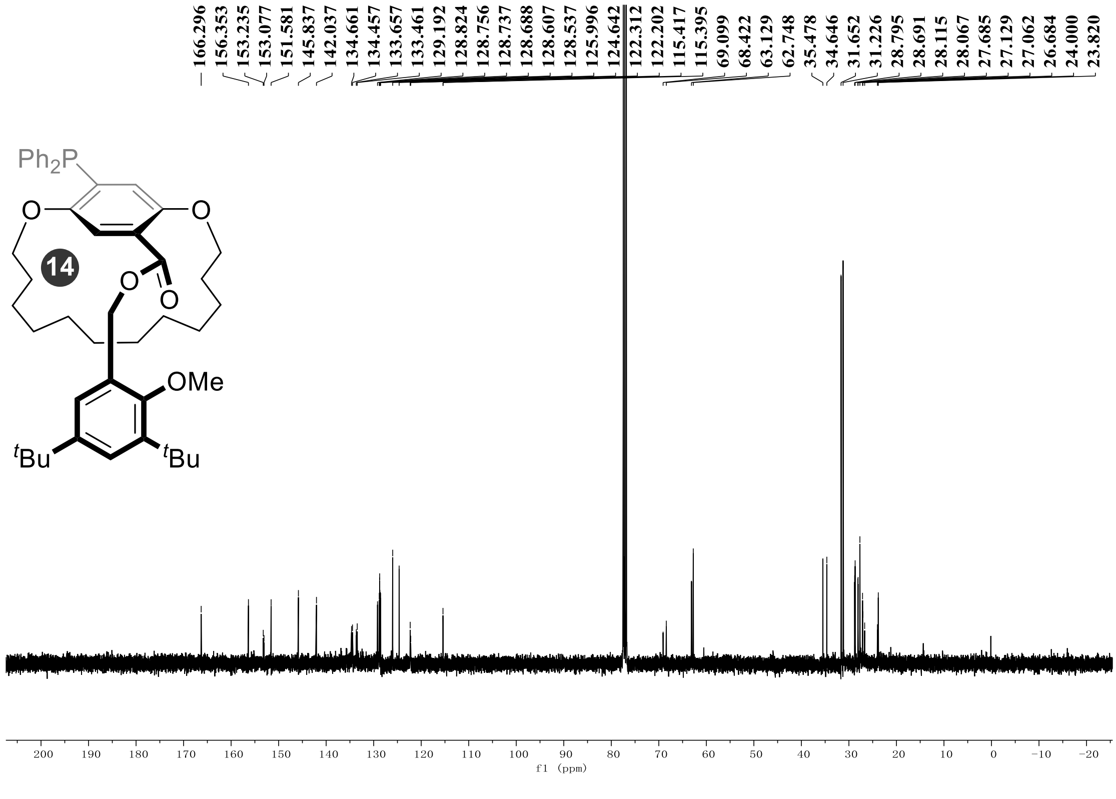

Supplementary Figure 59. **P NMR** spectrum of compound of **7**

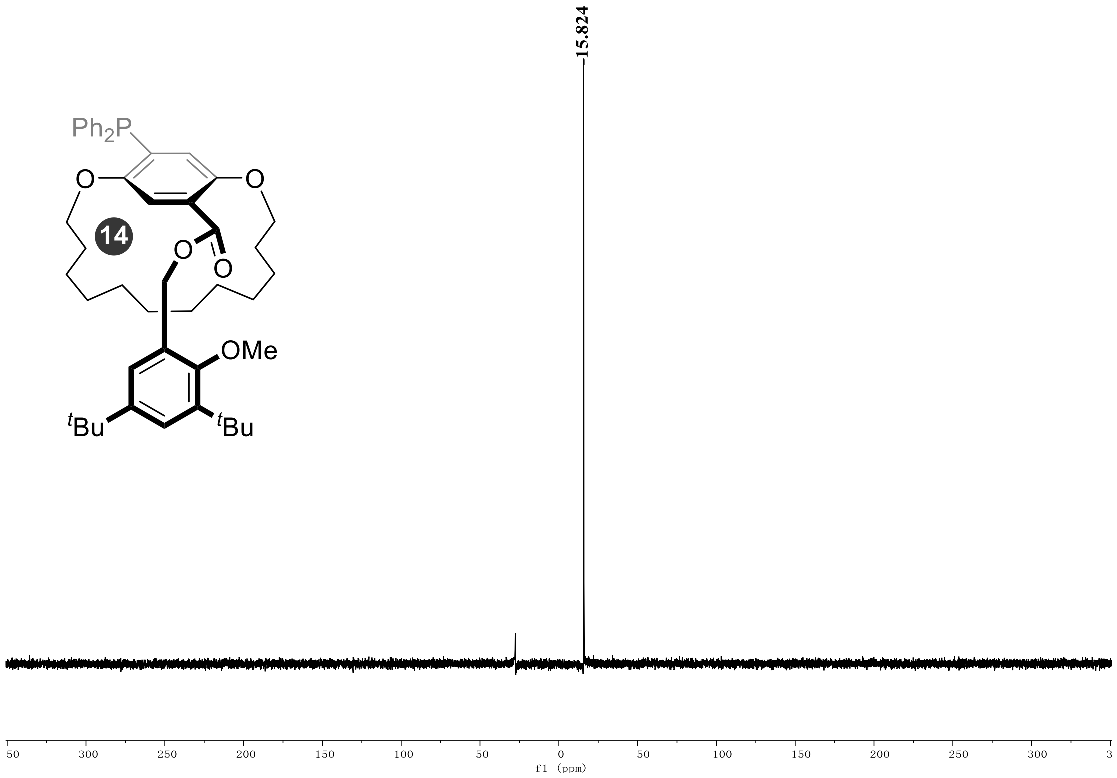

Supplementary Figure 60. ^{1}H NMR and ^{13}C NMR spectrum of compound of **8**

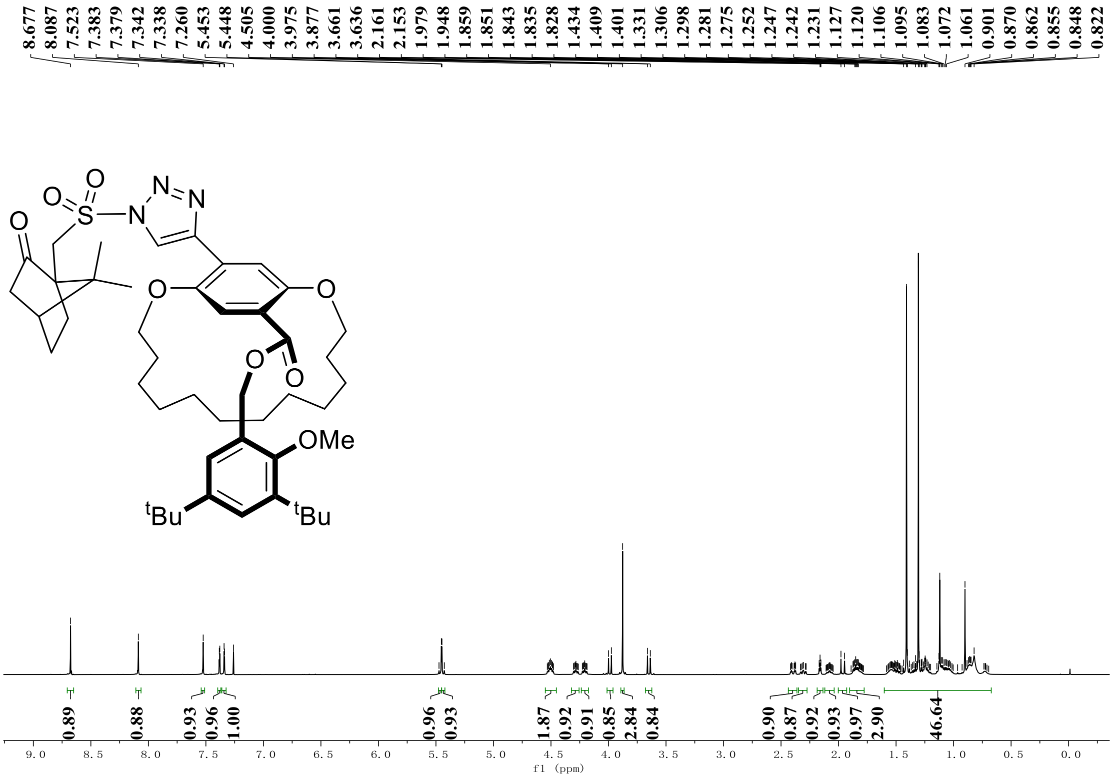

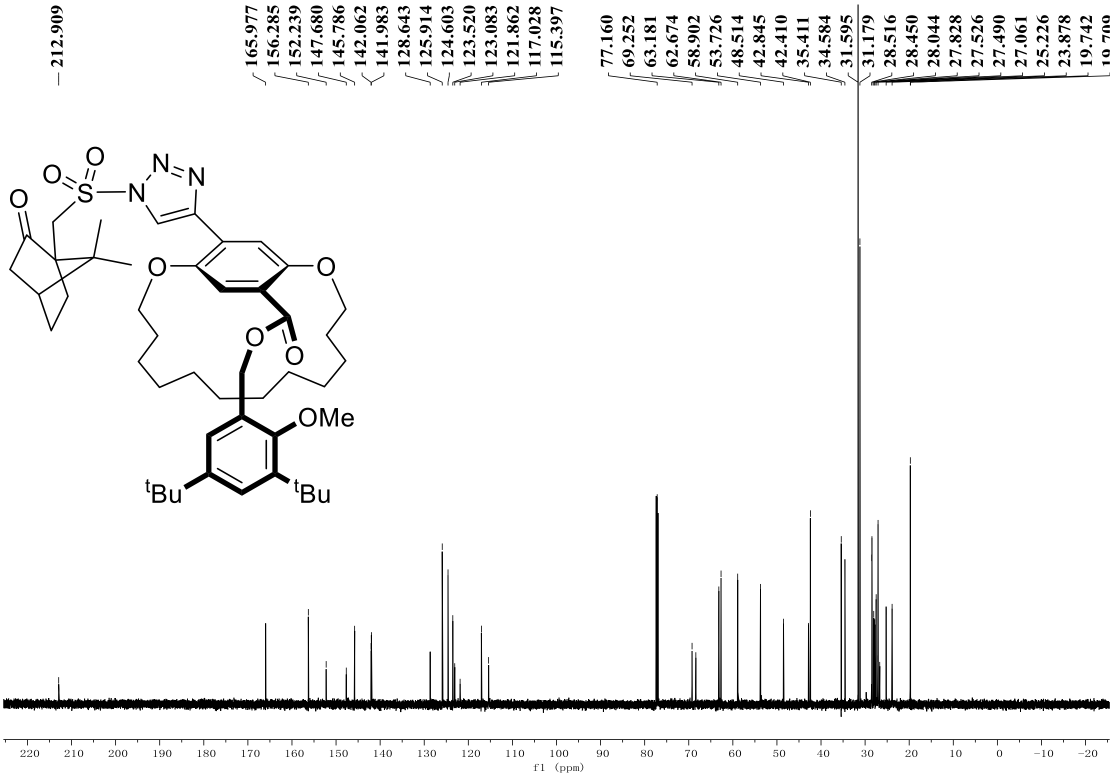

Supplementary Figure 61. ^{1}H NMR and ^{13}C NMR spectrum of compound of **9**

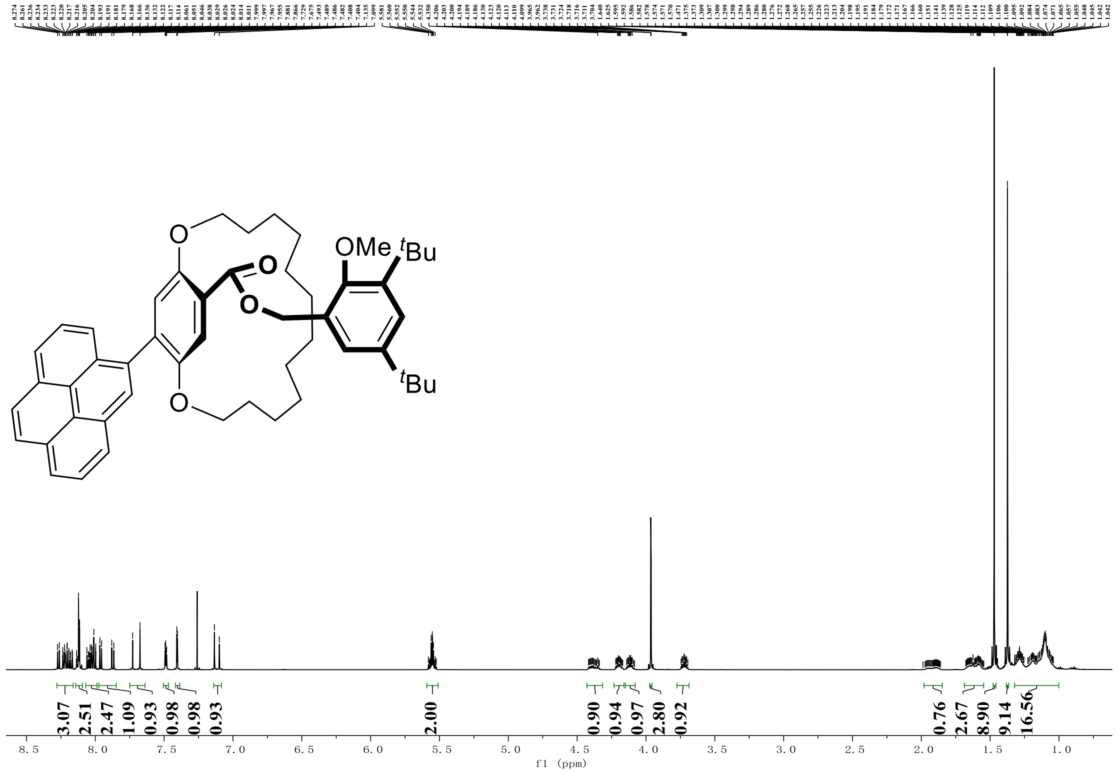

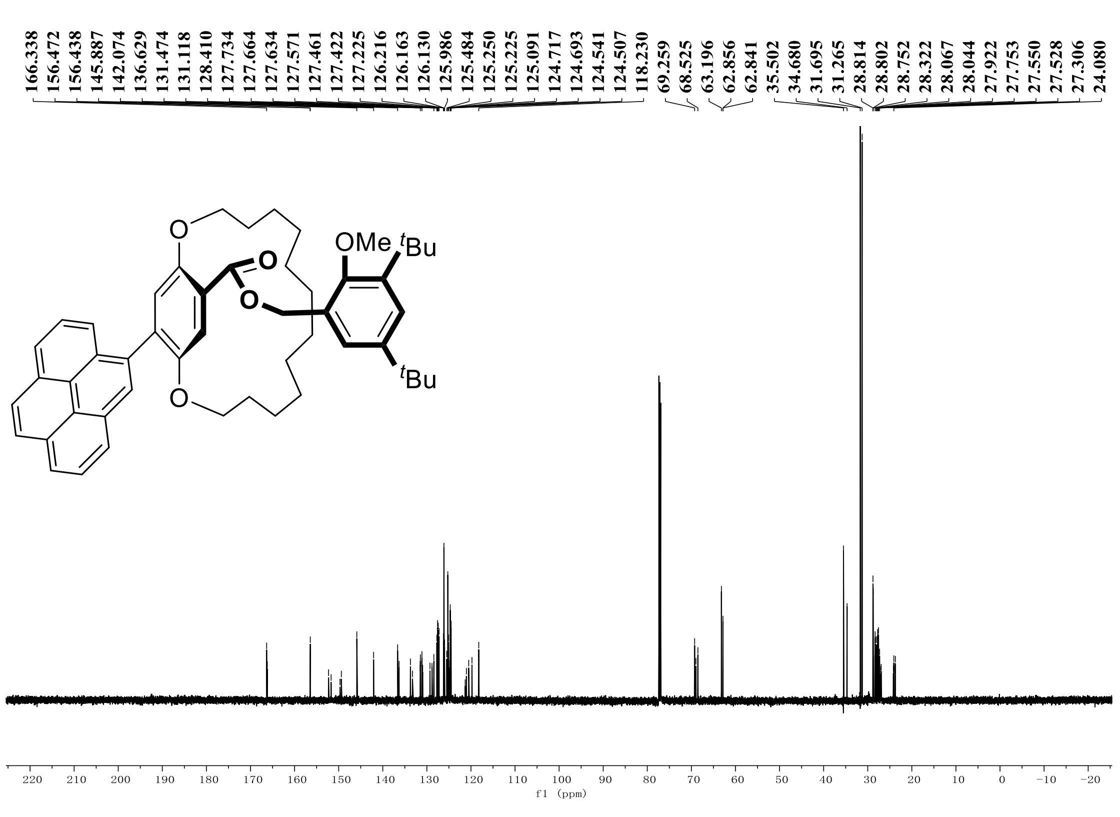

Supplementary Figure 62. ^{1}H NMR and ^{13}C NMR spectrum of compound of **10**

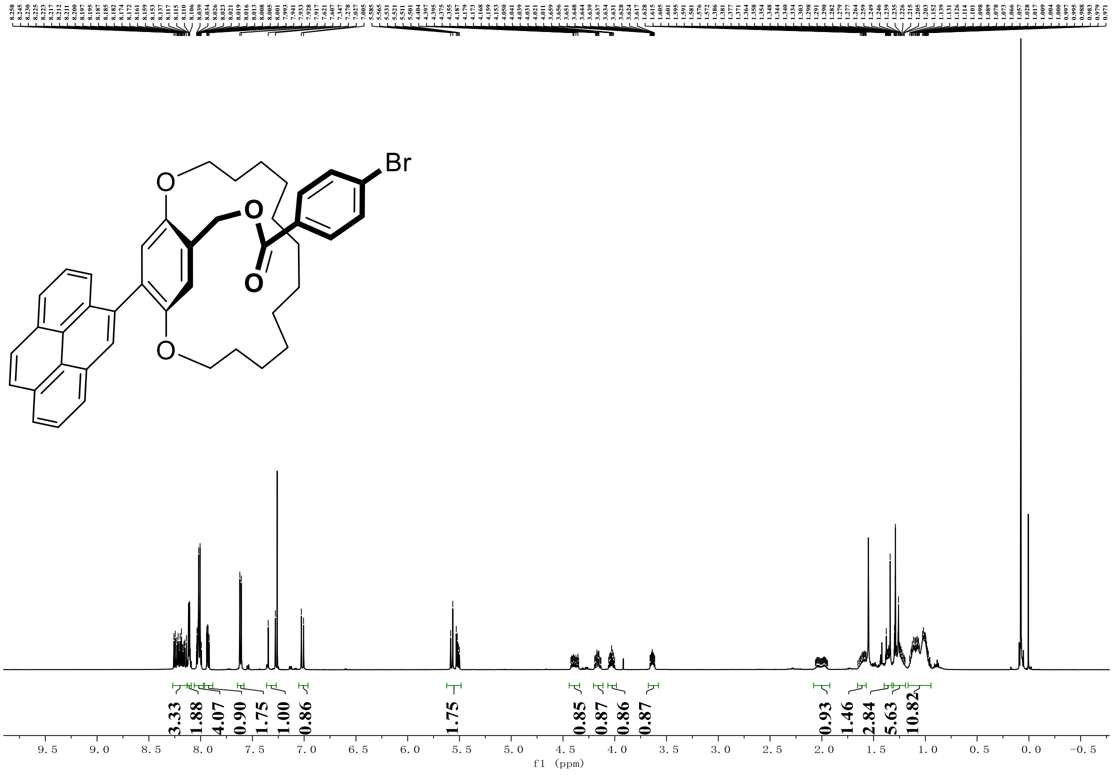

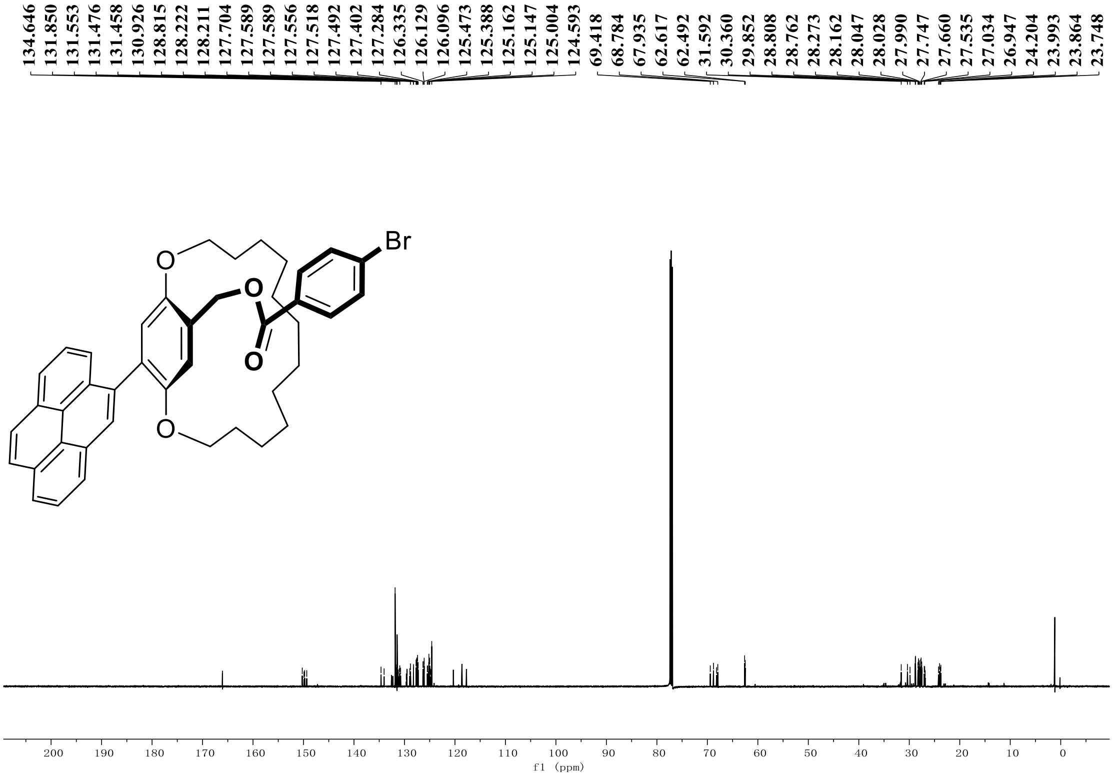

Supplementary Figure 63. **^{1}H NMR and ^{13}C NMR** spectrum of compound of **12**

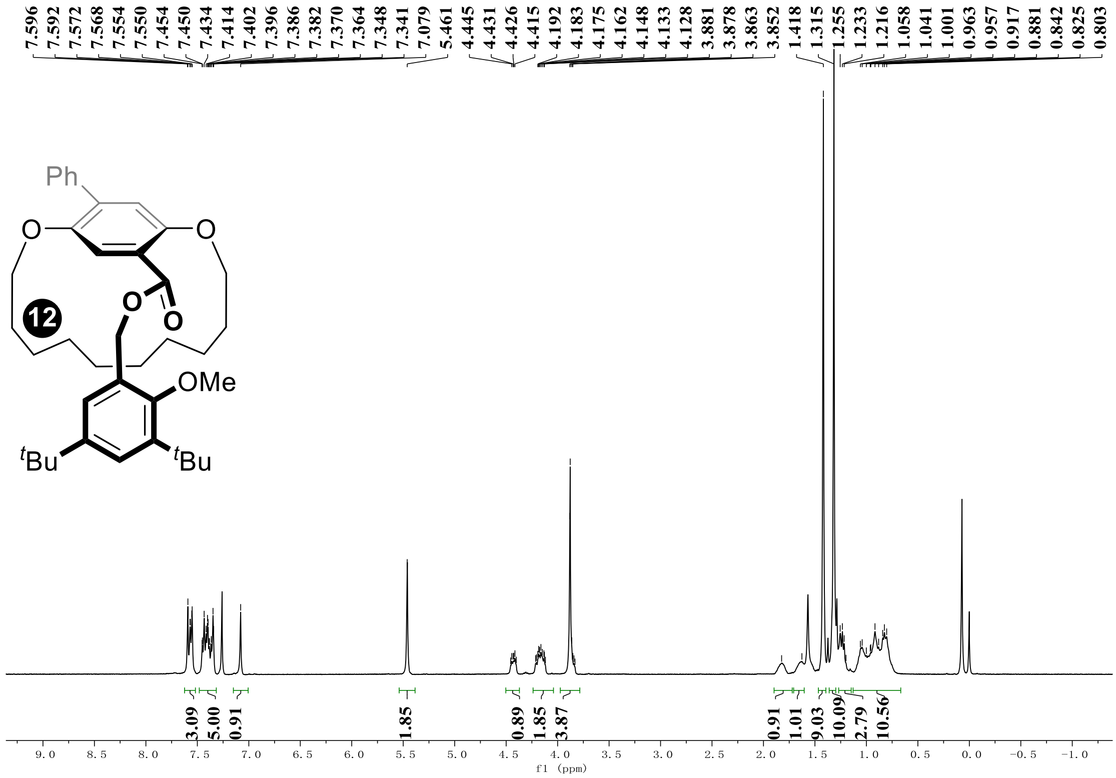

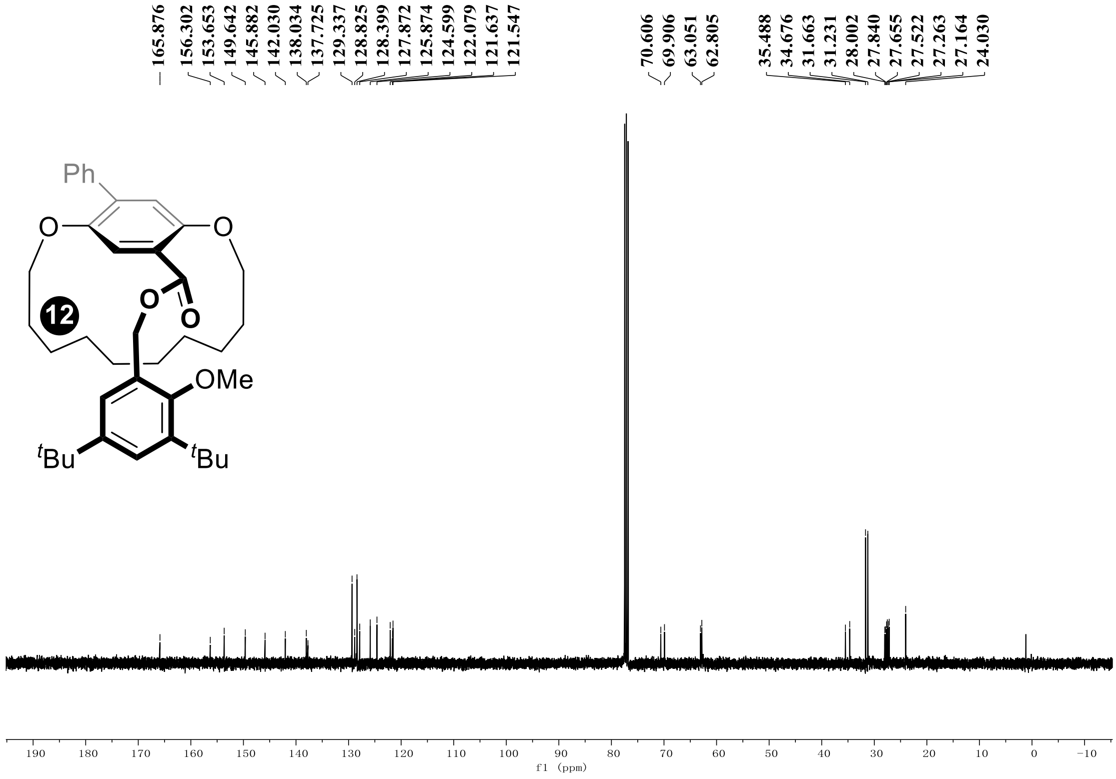

2.4 Copies of HPLC spectra

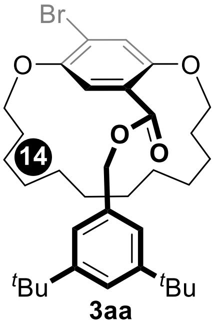

3aa

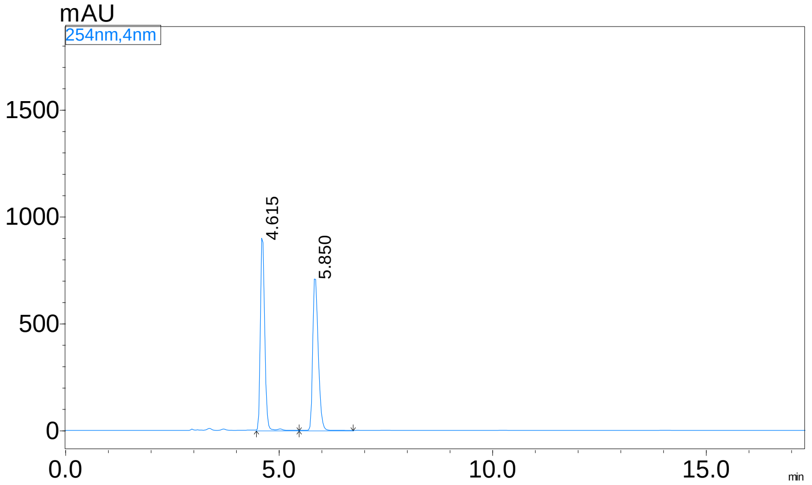

Peak#	Ret. Time	Area	Height	Area%
1	4.615	6369207	899037	50.086
2	5.850	6347369	708046	49.914

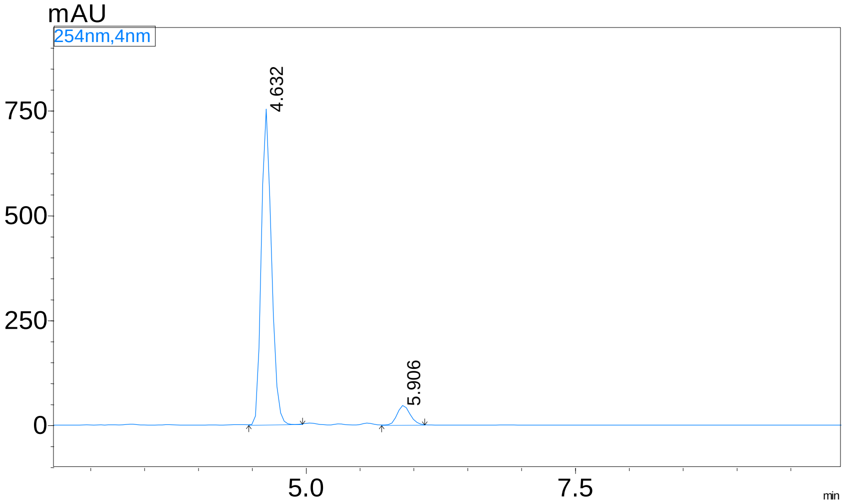

Peak#	Ret.Time	Area	Height	Area%
1	4.632	4913278	752044	92.831
2	5.906	379407	46205	7.169

Supplementary Figure 64. HPLC spectra of **3aa**

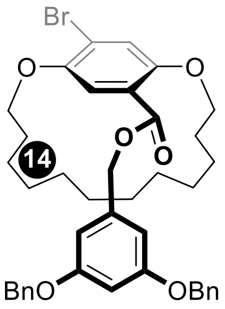

3ab

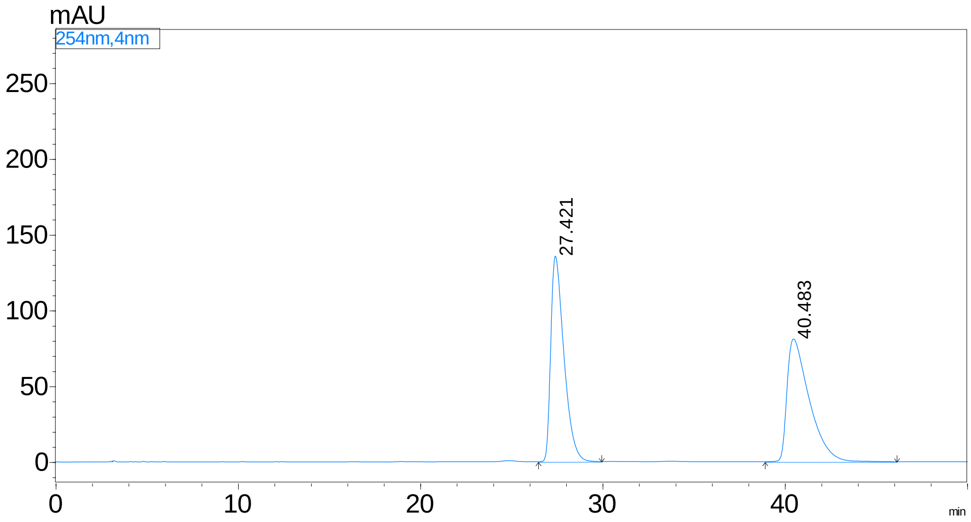

Peak#	Ret.Time	Area	Height	Area%
1	27.421	6817267	135636	49.832
2	40.483	6863112	80957	50.168

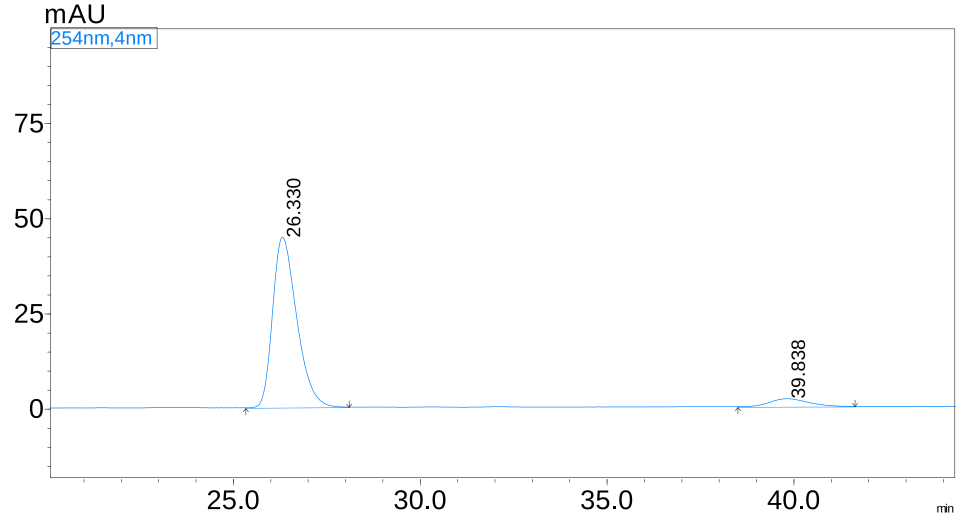

Peak#	Ret.Time	Area	Height	Area%
1	26.330	2004863	44775	93.243
2	39.838	145276	2059	6.757

Supplementary Figure 65. HPLC spectra of **3ab**

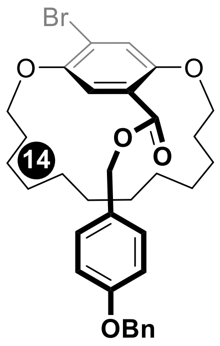

3ac

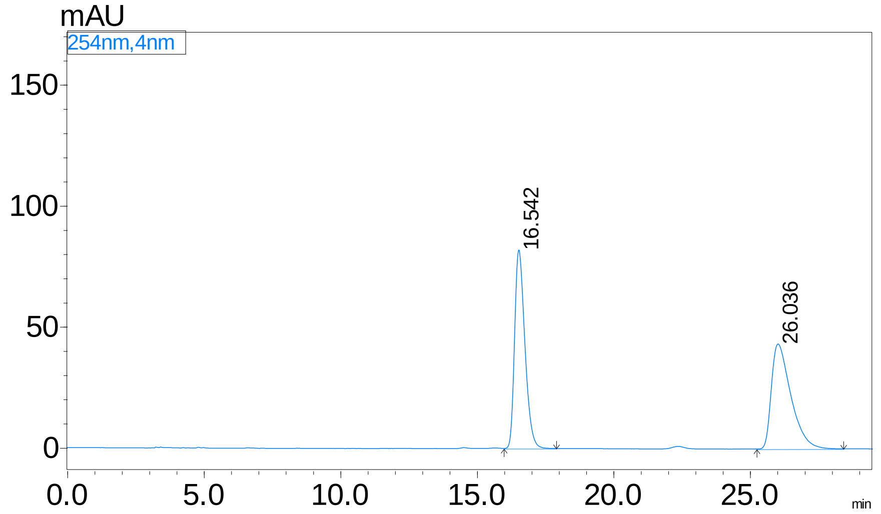

Peak#	Ret.Time	Area	Height	Area%
1	16.542	2157327	82009	50.070
2	26.036	2151325	43385	49.930

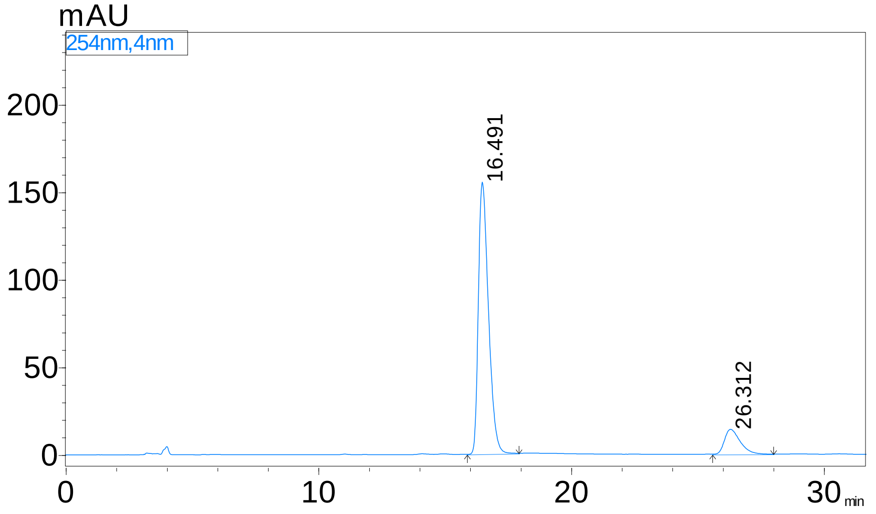

Peak#	Ret.Time	Area	Height	Area%
1	16.491	4310961	155316	87.626
2	26.312	608746	14201	12.374

Supplementary Figure 66. HPLC spectra of **3ac**

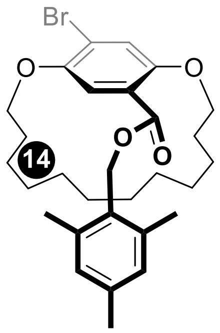

3ad

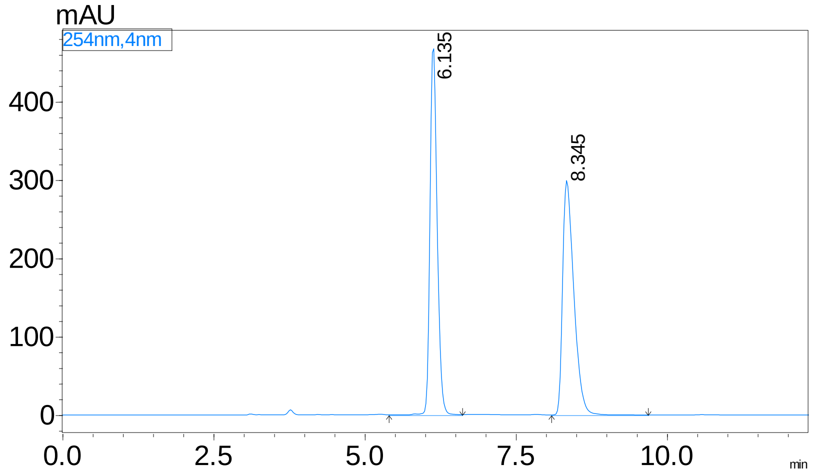

Peak#	Ret.Time	Area	Height	Area%
1	6.135	3887181	467503	50.196
2	8.345	3856882	299244	49.804

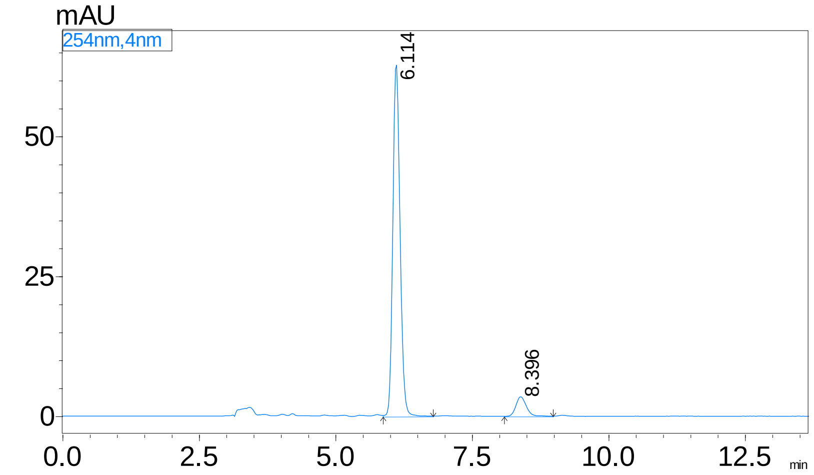

Peak#	Ret.Time	Area	Height	Area%
1	6.114	577899	62773	92.649
2	8.396	45855	3476	7.351

Supplementary Figure 67. HPLC spectra of **3ad**

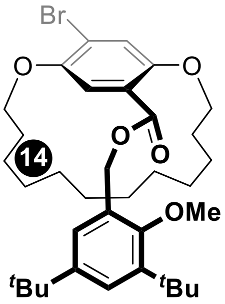

3ae

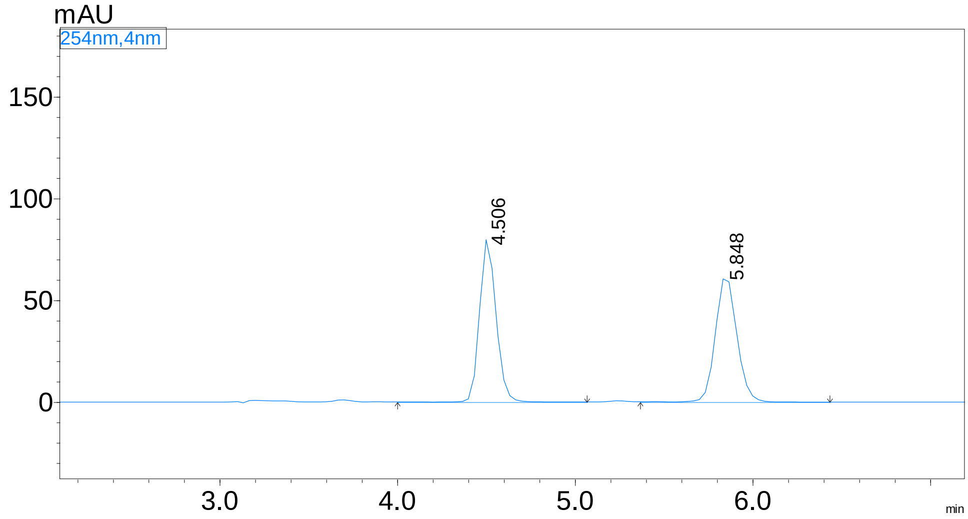

Peak#	Ret.Time	Area	Height	Area%
1	4.506	516759	79744	49.955
2	5.848	517693	60532	50.045

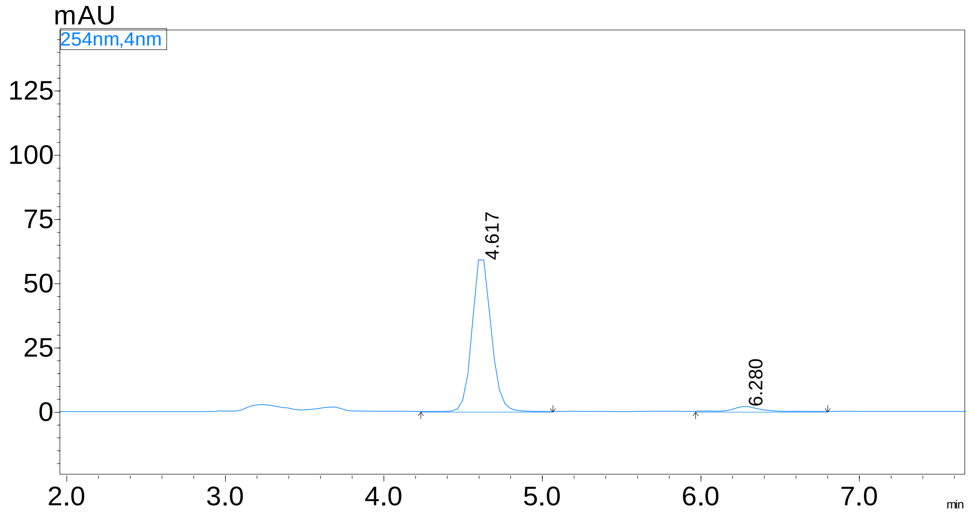

Peak#	Ret.Time	Area	Height	Area%
1	4.617	502136	59099	95.342
2	6.280	24531	1956	4.658

Supplementary Figure 68. HPLC spectra of **3ae**

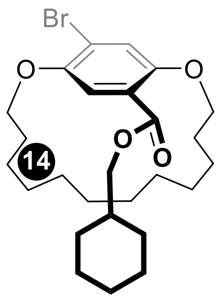

3af

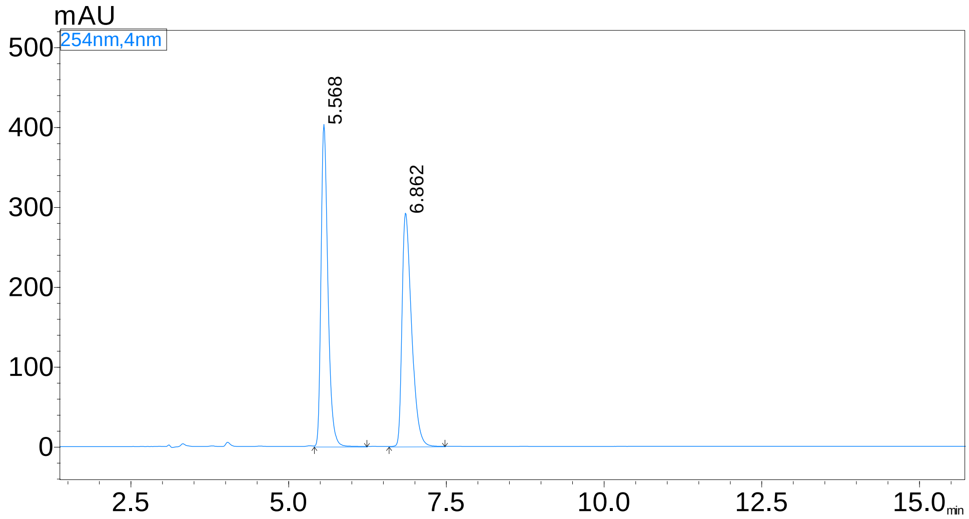

Peak#	Ret.Time	Area	Height	Area%
1	5.568	3060001	403171	49.987
2	6.862	3061631	291936	50.013

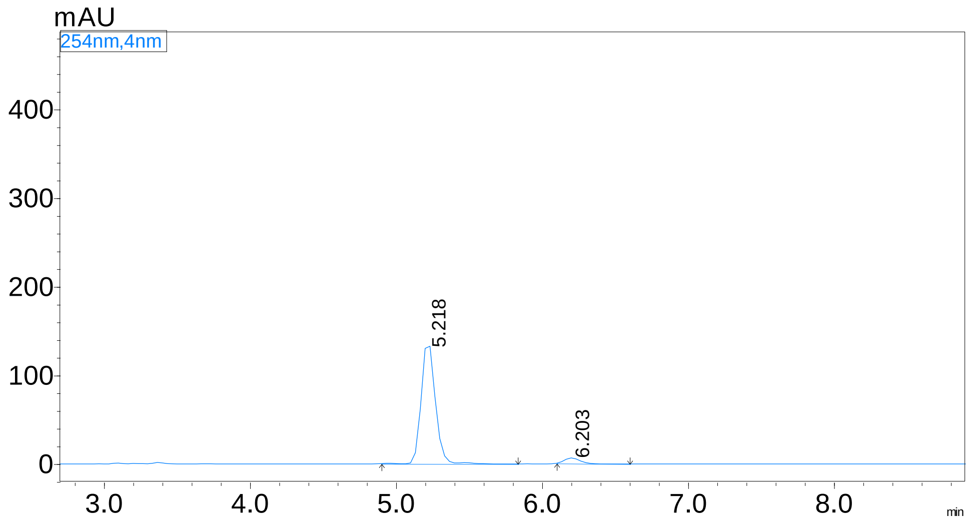

Peak#	Ret.Time	Area	Height	Area%
1	5.218	917912	132611	95.653
2	6.203	41720	6319	4.347

Supplementary Figure 69. HPLC spectra of **3af**

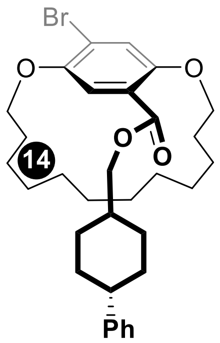

3ag

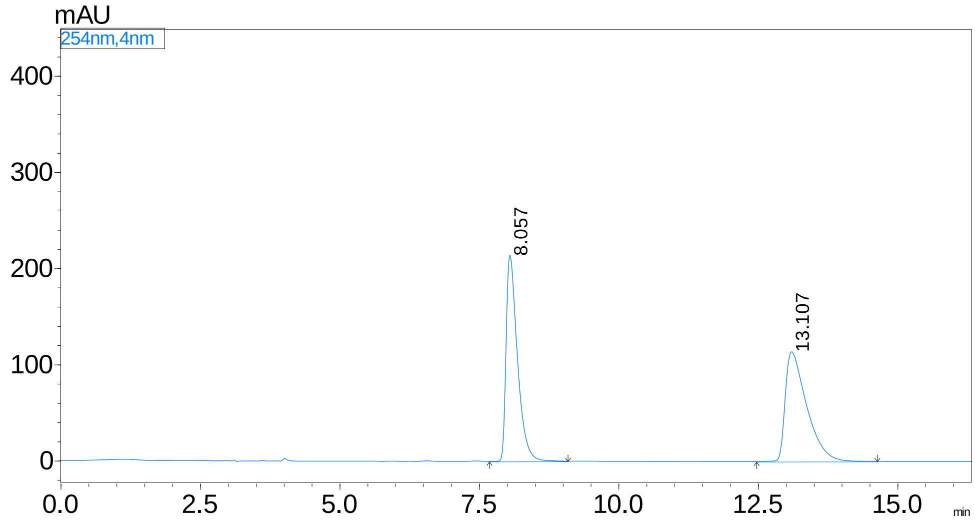

Peak#	Ret.Time	Area	Height	Area%
1	8.057	3019767	214089	49.886
2	13.107	3033589	113936	50.114

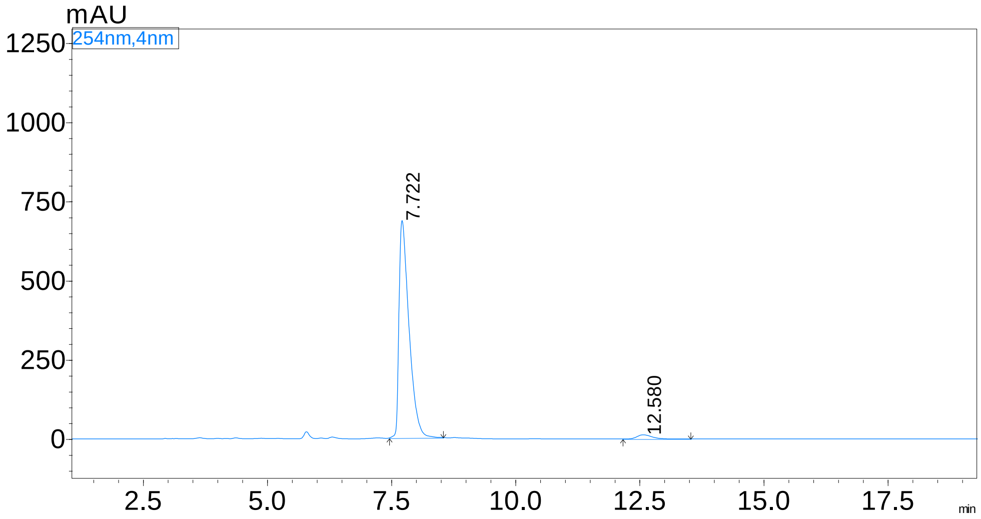

Peak#	Ret.Time	Area	Height	Area%
1	7.722	9617015	686363	97.305
2	12.580	266334	13109	2.695

Supplementary Figure 70. HPLC spectra of **3ag**

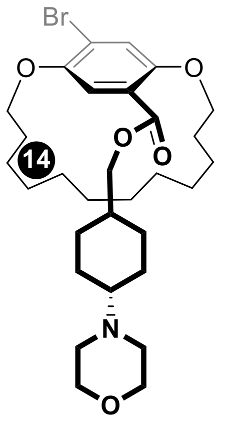

3ah

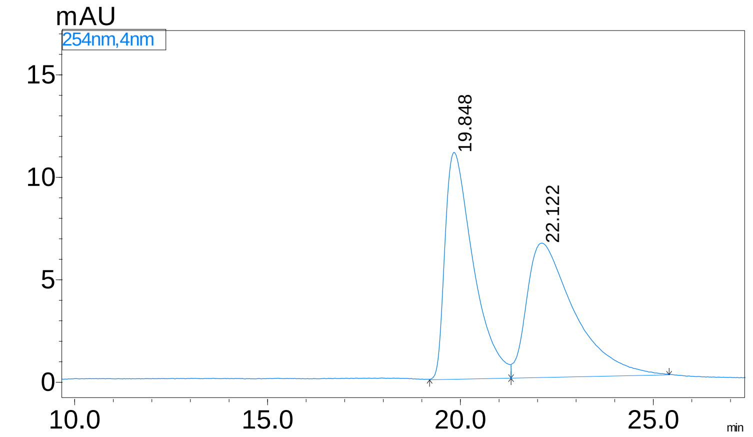

Peak#	Ret.Time	Area	Height	Area%
1	19.848	565372	11044	50.288
2	22.122	558908	6528	49.712

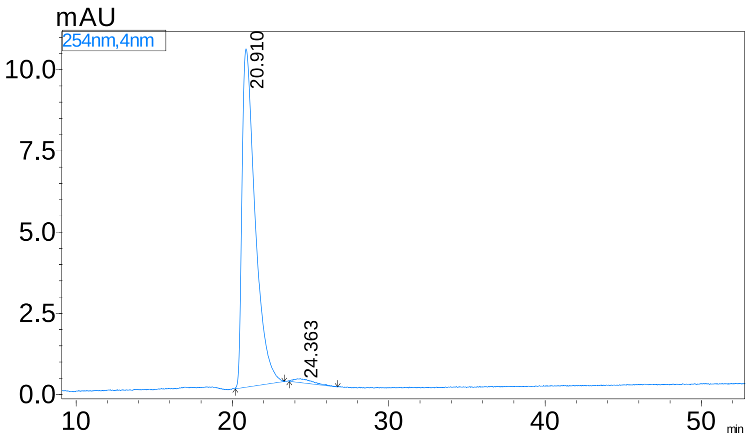

Peak#	Ret.Time	Area	Height	Area%
1	20.910	605622	10397	98.733
2	24.363	7772	113	1.267

Supplementary Figure 71. HPLC spectra of **3ah**

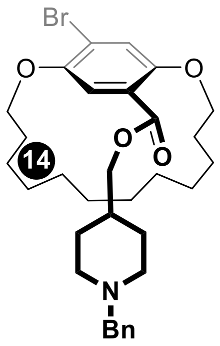

3ai

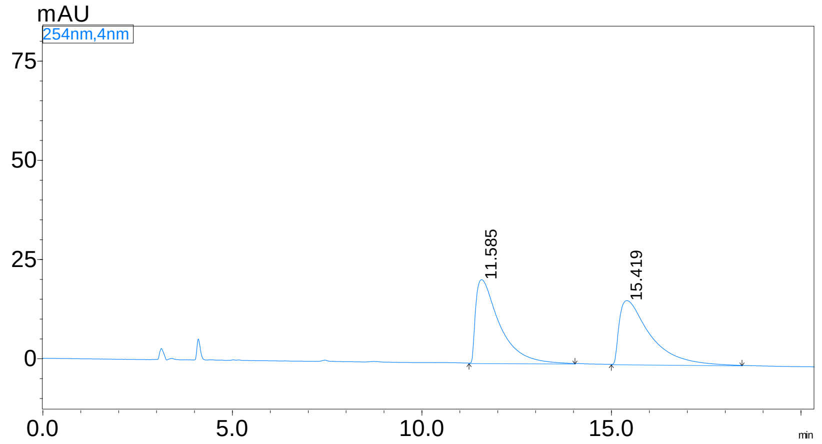

Peak#	Ret.Time	Area	Height	Area%
1	11.585	919646	21037	50.284
2	15.419	909270	16077	49.716

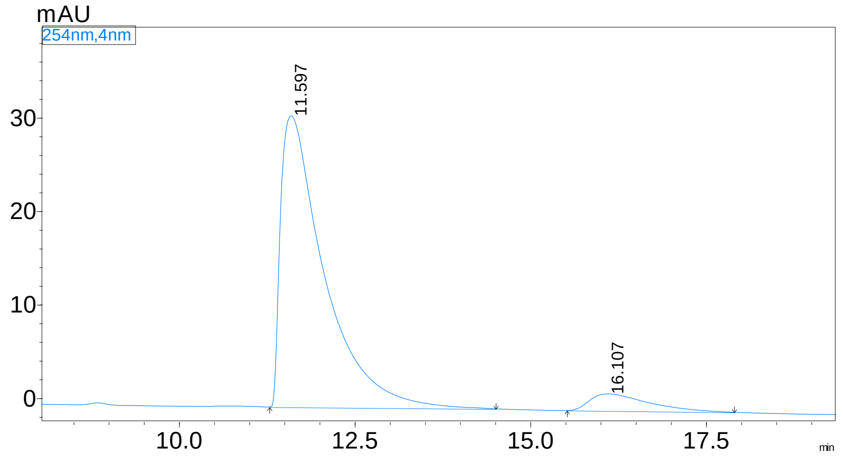

Peak#	Ret.Time	Area	Height	Area%
1	11.597	1345736	31157	92.708
2	16.107	105845	1826	7.292

Supplementary Figure 72. HPLC spectra of **3ai**

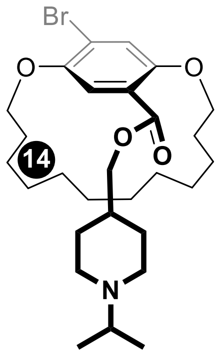

3aj

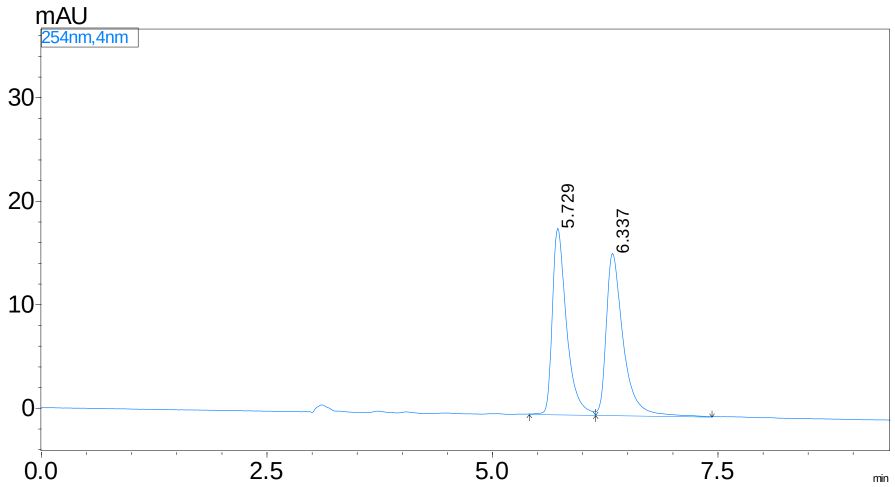

Peak#	Ret.Time	Area	Height	Area%
1	5.729	196696	18004	49.160
2	6.337	203419	15640	50.840

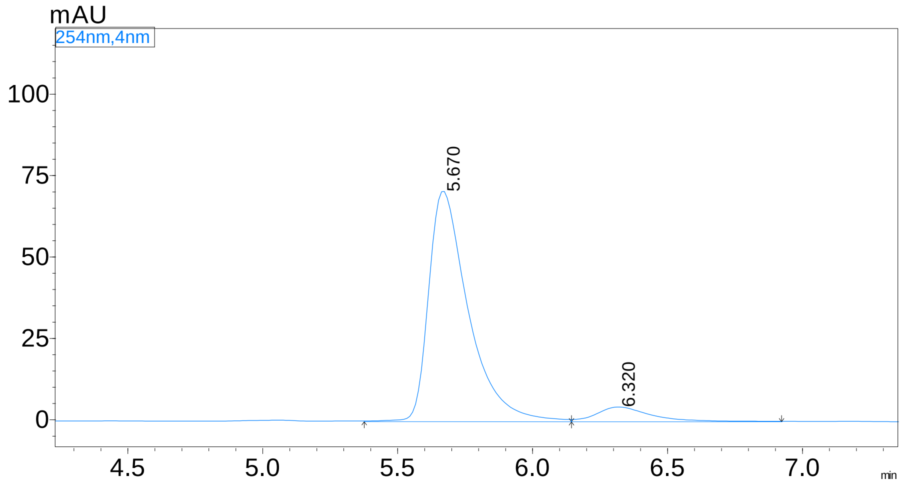

Peak#	Ret.Time	Area	Height	Area%
1	5.670	722382	70551	92.556
2	6.320	58099	4380	7.444

Supplementary Figure 73. HPLC spectra of **3aj**

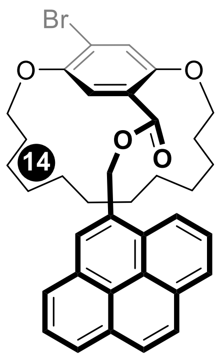

3ak

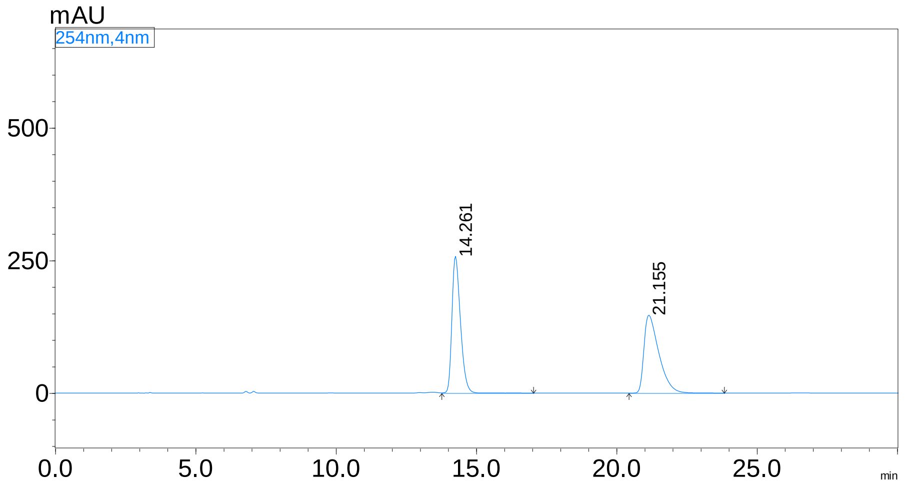

Peak#	Ret.Time	Area	Height	Area%
1	14.261	5436691	257756	49.993
2	21.155	5438138	146641	50.007

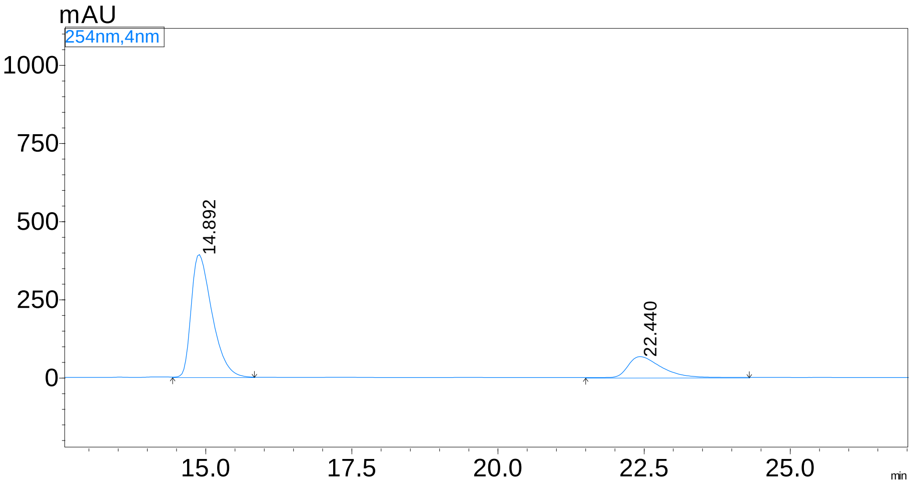

Peak#	Ret.Time	Area	Height	Area%
1	14.892	9682600	391837	79.065
2	22.440	2563845	66925	20.935

Supplementary Figure 74. HPLC spectra of **3ak**

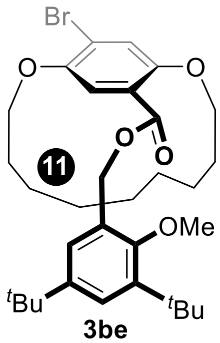

3be

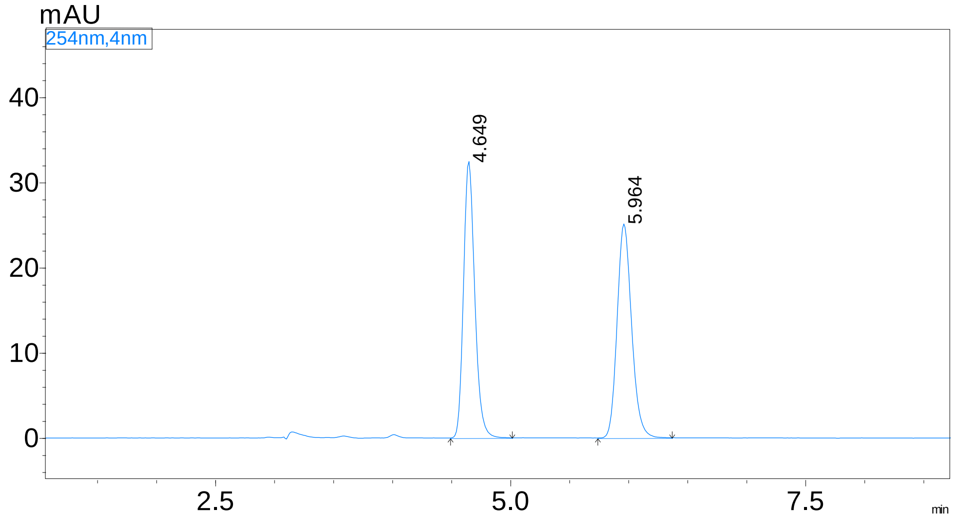

Peak#	Ret.Time	Area	Height	Area%
1	4.649	215373	32446	50.094
2	5.964	214563	25116	49.906

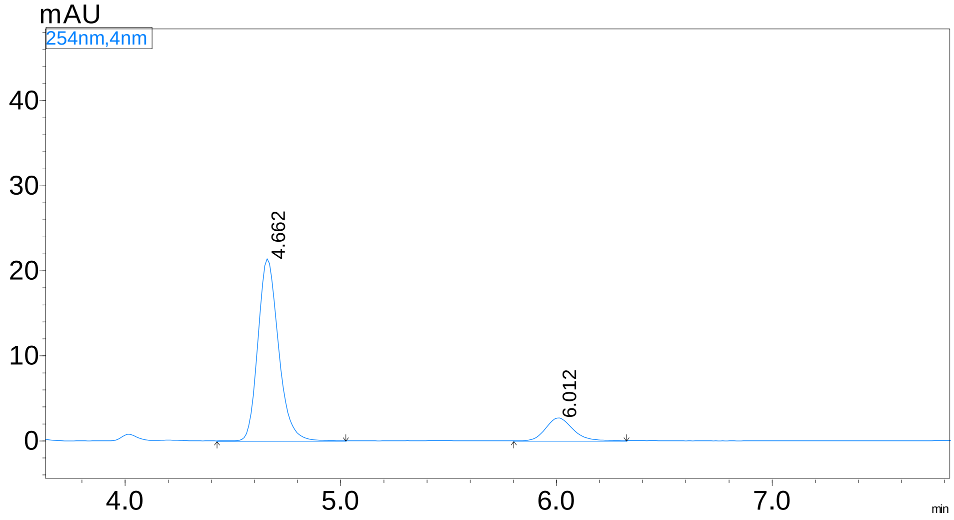

Peak#	Ret.Time	Area	Height	Area%
1	4.662	141240	21409	85.813
2	6.012	23350	2681	14.187

Supplementary Figure 75. HPLC spectra of **3be**

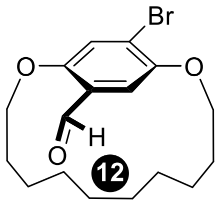

1c

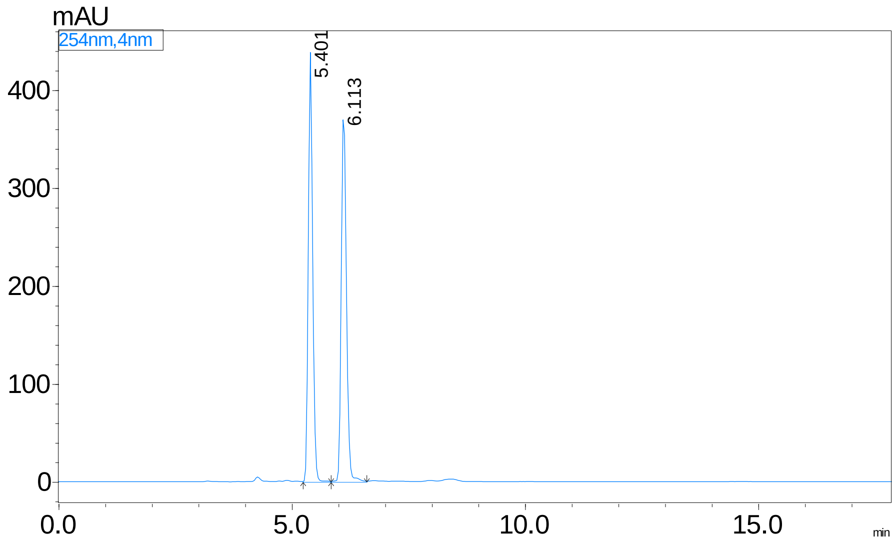

Peak#	Ret.Time	Area	Height	Area%
1	5.401	2846875	438376	49.626
2	6.113	2889829	369555	50.374

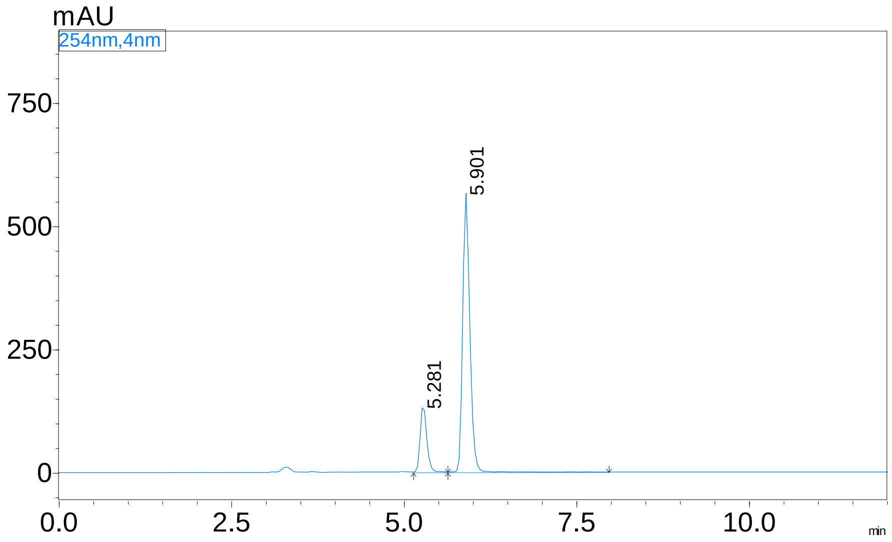

Peak#	Ret.Time	Area	Height	Area%
1	5.281	906965	130764	18.080
2	5.901	4109483	565802	81.920

Supplementary Figure 76. HPLC spectra of **1c**

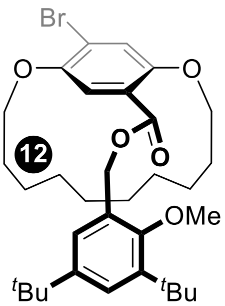

3ce

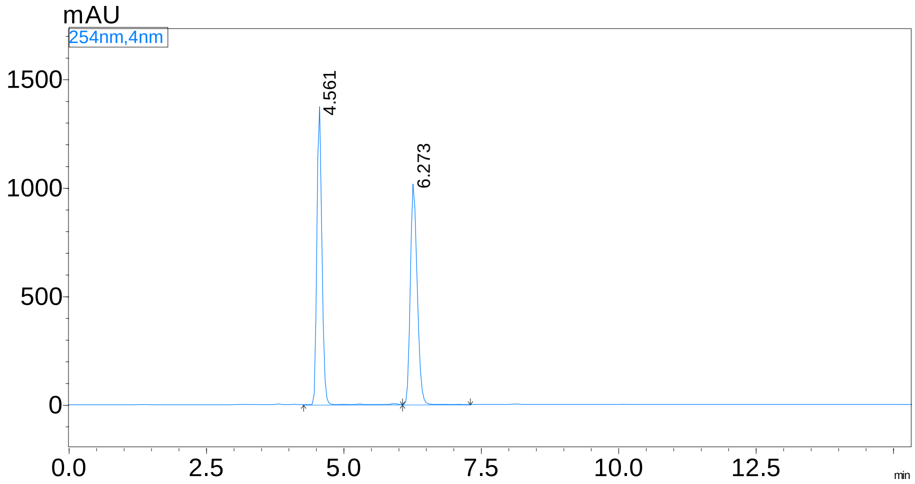

Peak#	Ret.Time	Area	Height	Area%
1	4.561	8828125	1373698	50.058
2	6.273	8807716	1016183	49.942

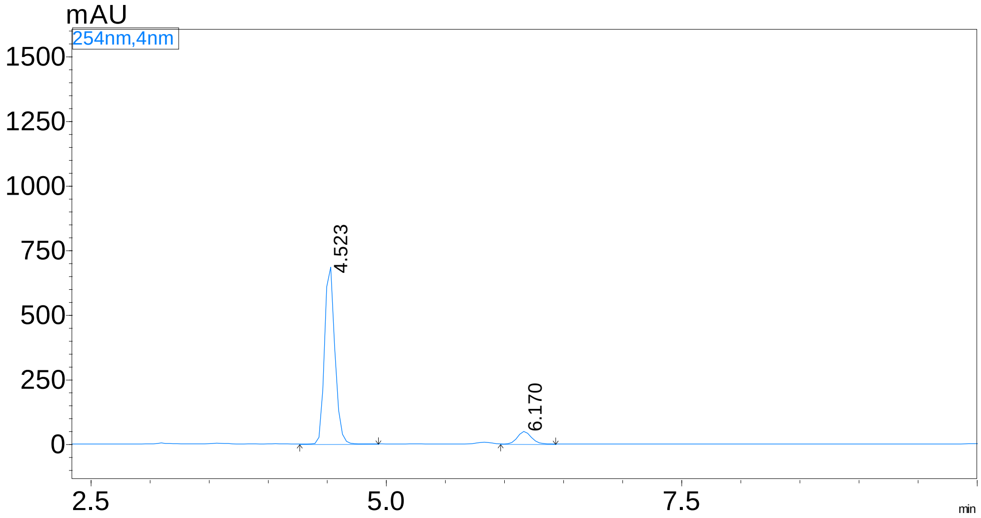

Peak#	Ret.Time	Area	Height	Area%
1	4.523	4178907	684335	91.544
2	6.170	385993	48331	8.456

Supplementary Figure 77. HPLC spectra of **3ce**

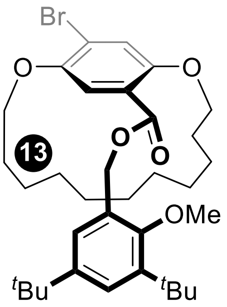

3de

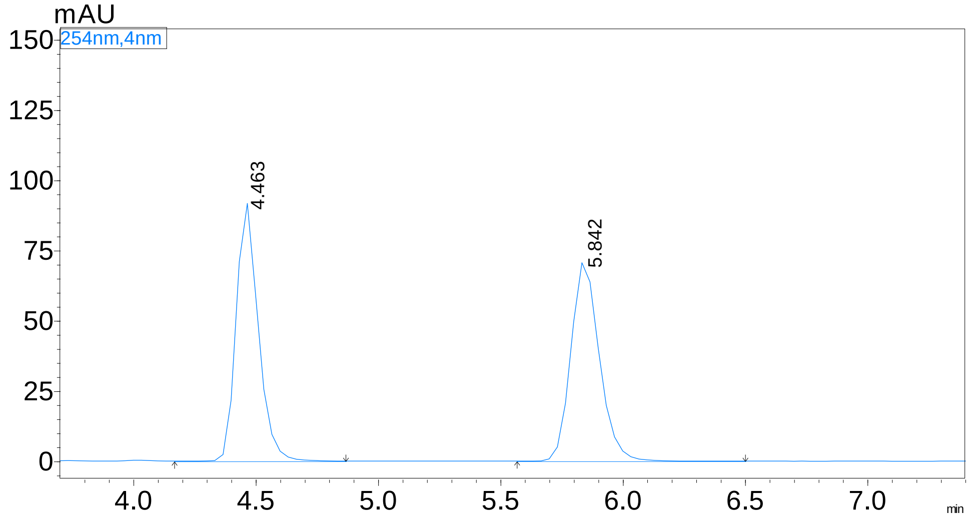

Peak#	Ret.Time	Area	Height	Area%
1	4.463	575832	91712	50.119
2	5.842	573106	70640	49.881

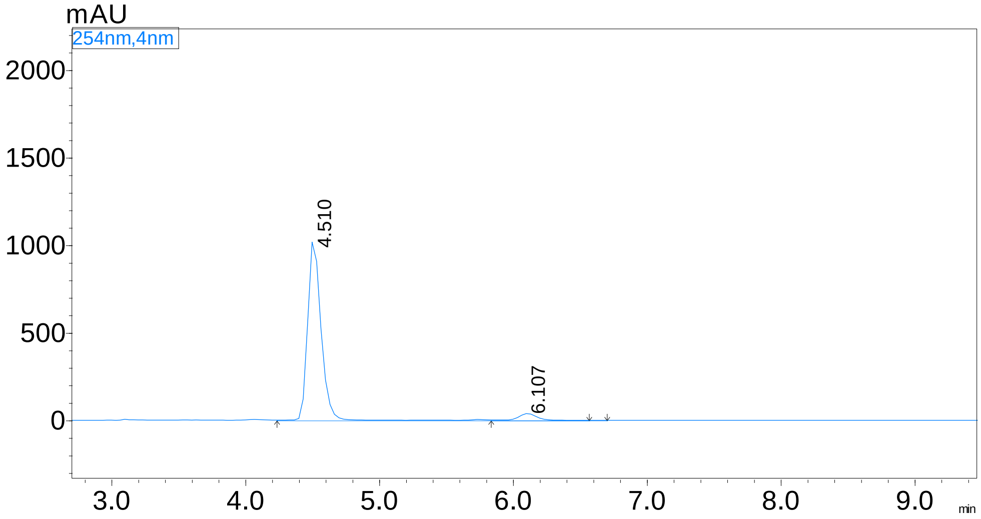

Peak#	Ret.Time	Area	Height	Area%
1	4.510	7141436	1017845	95.311
2	6.107	351355	38288	4.689

Supplementary Figure 78. HPLC spectra of **3de**

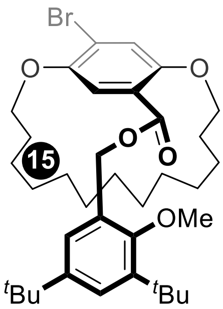

3ee

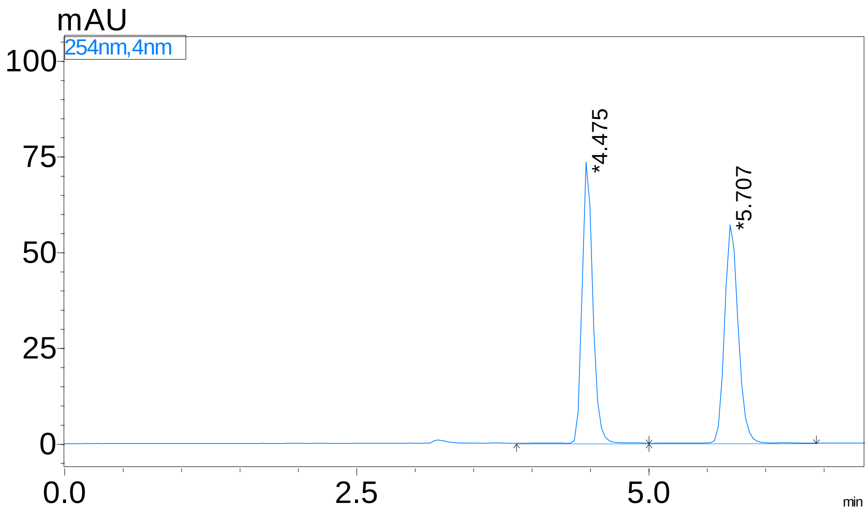

Peak#	Ret.Time	Area	Height	Area%
1	4.475	465039	73346	50.187
2	5.707	461571	57002	49.813

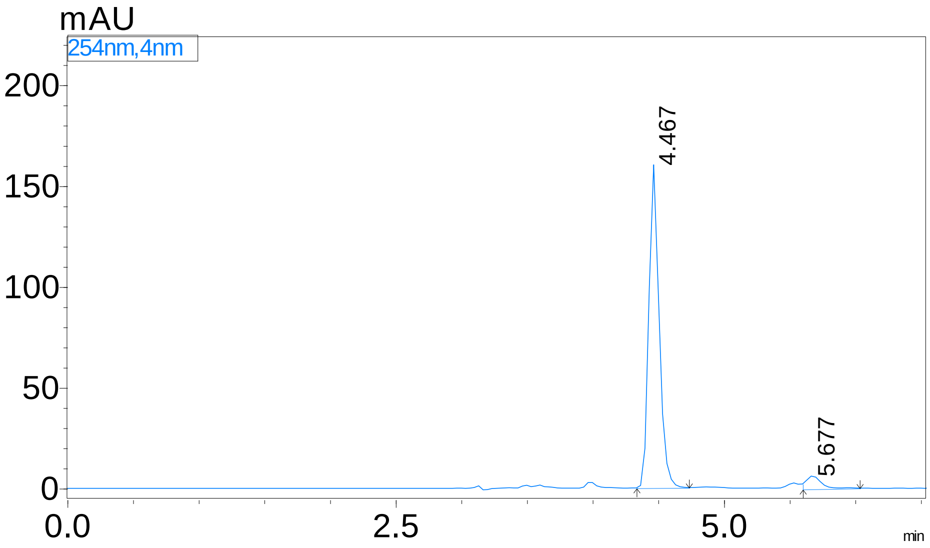

Peak#	Ret.Time	Area	Height	Area%
1	4.467	872639	160182	94.691
2	5.677	48923	6360	5.309

Supplementary Figure 79. HPLC spectra of **3ee**

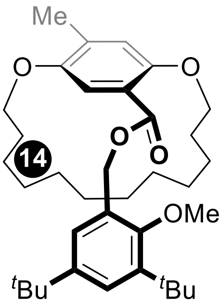

3he

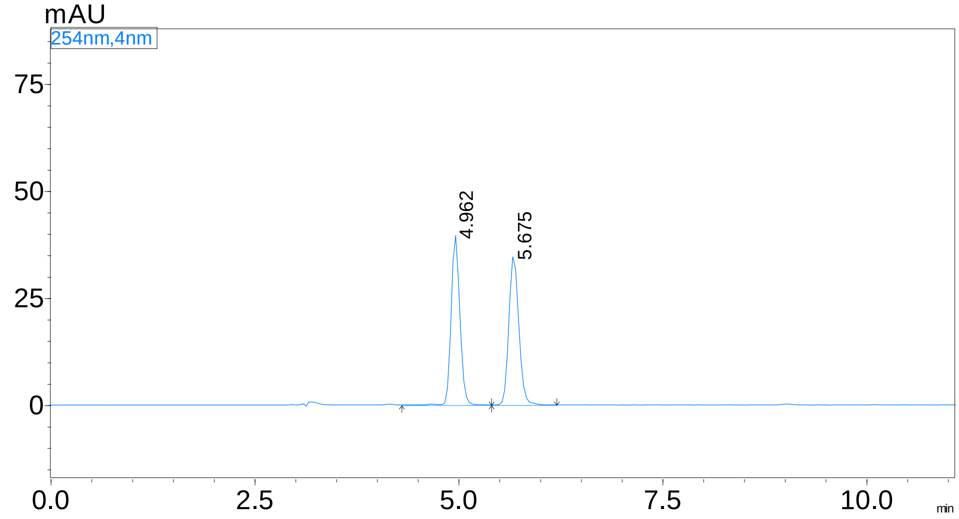

Peak#	Ret.Time	Area	Height	Area%
1	4.962	294481	39567	49.940
2	5.675	295187	34618	50.060

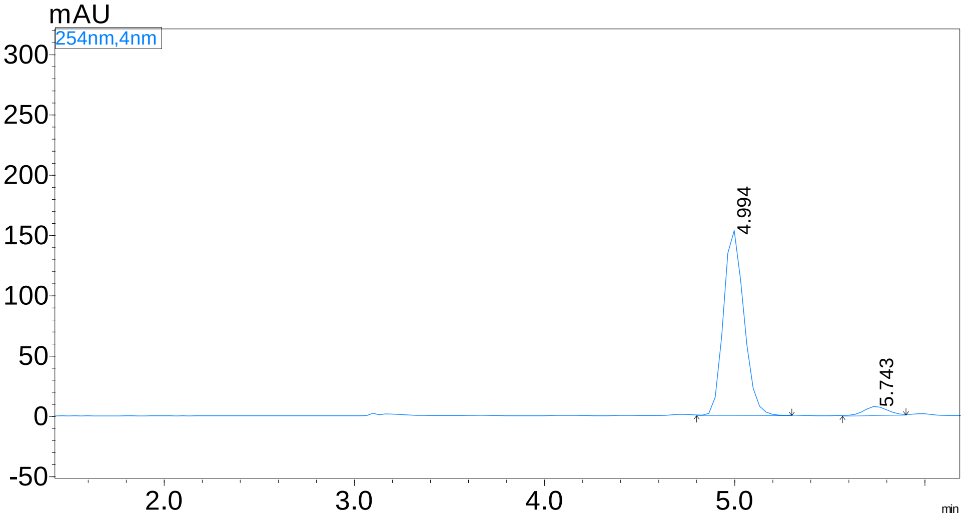

Peak#	Ret.Time	Area	Height	Area%
1	4.994	1140780	153074	95.086
2	5.743	58950	7047	4.914

Supplementary Figure 80. HPLC spectra of **3he**

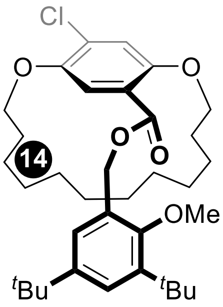

3ie

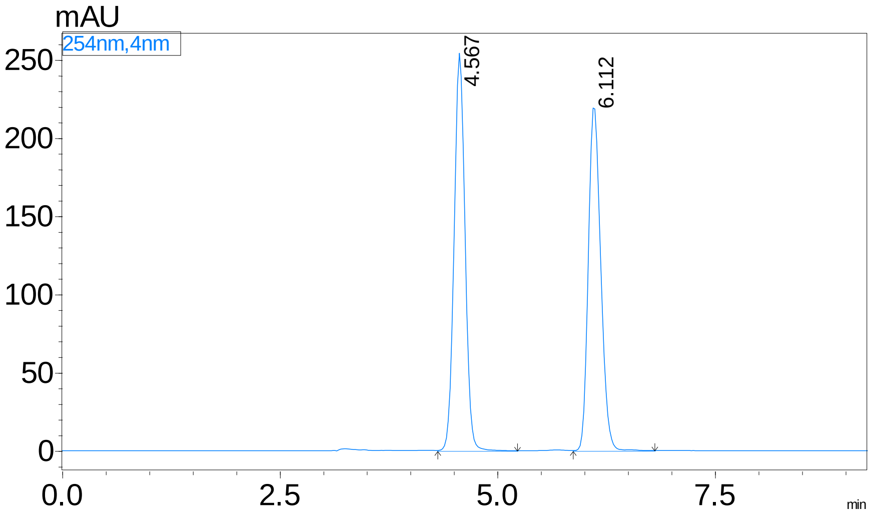

Peak#	Ret.Time	Area	Height	Area%
1	4.567	2214662	254059	50.355
2	6.112	2183430	219007	49.645

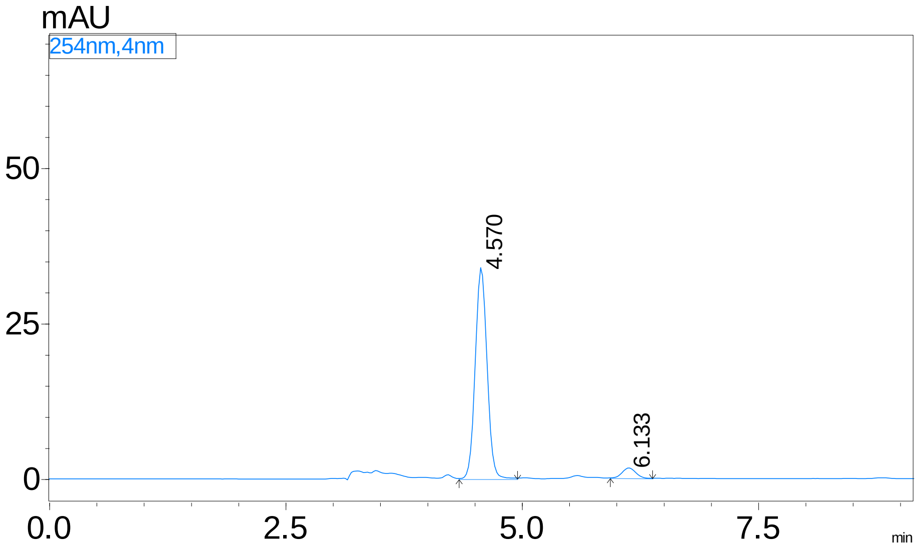

Peak#	Ret.Time	Area	Height	Area%
1	4.570	294799	33942	94.862
2	6.133	15966	1629	5.138

Supplementary Figure 81. HPLC spectra of **3ie**

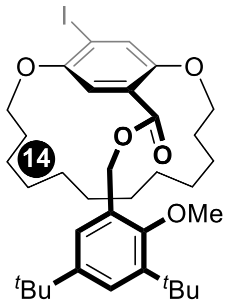

3je

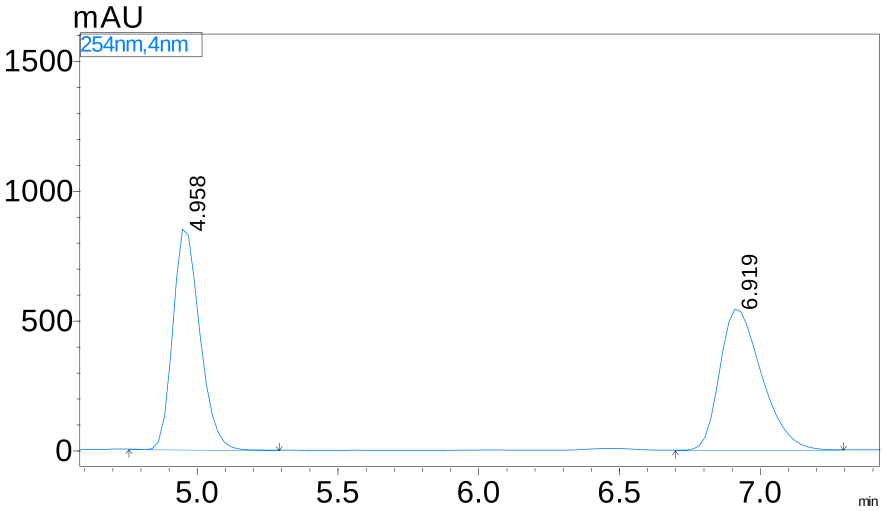

Peak#	Ret.Time	Area	Height	Area%
1	4.958	5629746	848517	50.058
2	6.919	5616592	543066	49.942

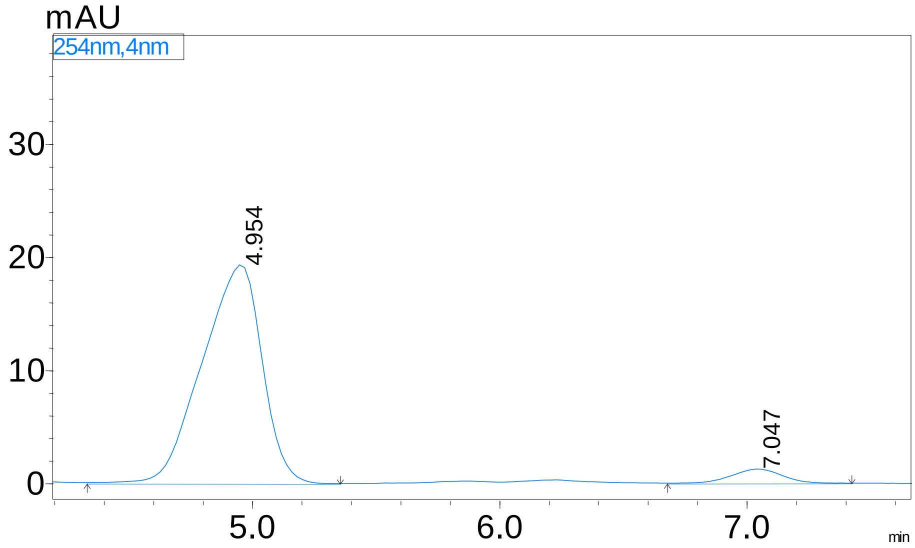

Peak#	Ret.Time	Area	Height	Area%
1	4.954	325509	19319	94.798
2	7.047	17863	1253	5.202

Supplementary Figure 82. HPLC spectra of **3je**

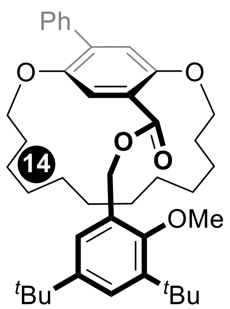

3ke

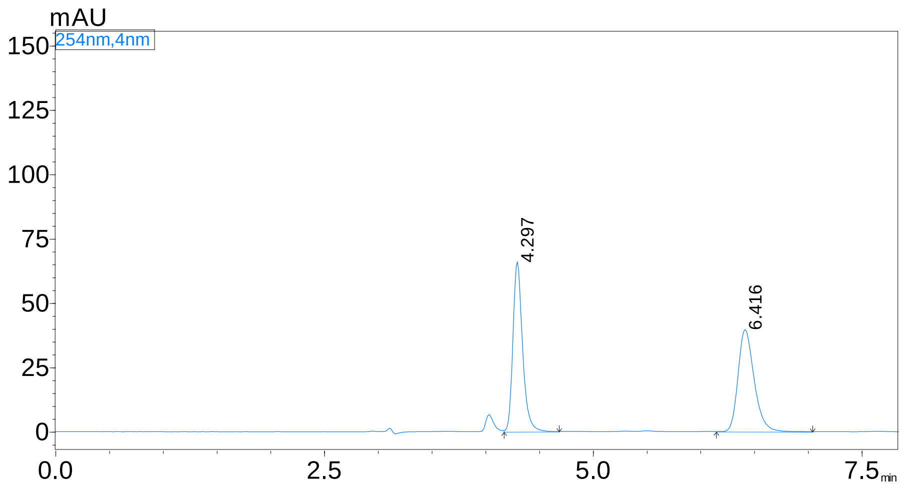

Peak#	Ret.Time	Area	Height	Area%
1	4.297	411067	65977	50.011
2	6.416	410890	39536	49.989

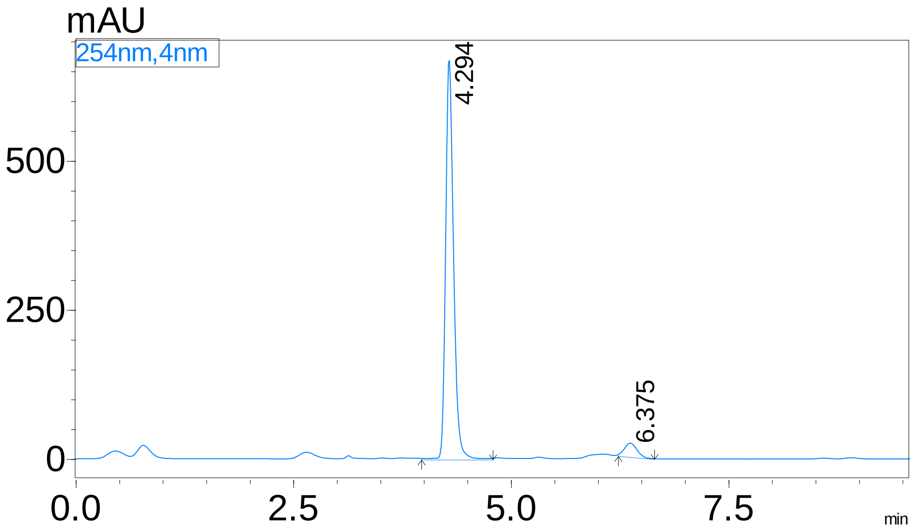

Peak#	Ret.Time	Area	Height	Area%
1	4.294	4239564	668389	95.066
2	6.375	220054	22652	4.934

Supplementary Figure 83. HPLC spectra of **3ke**

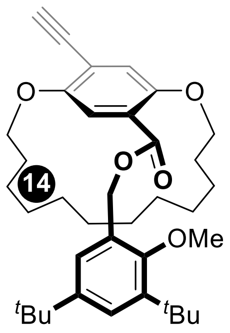

3le

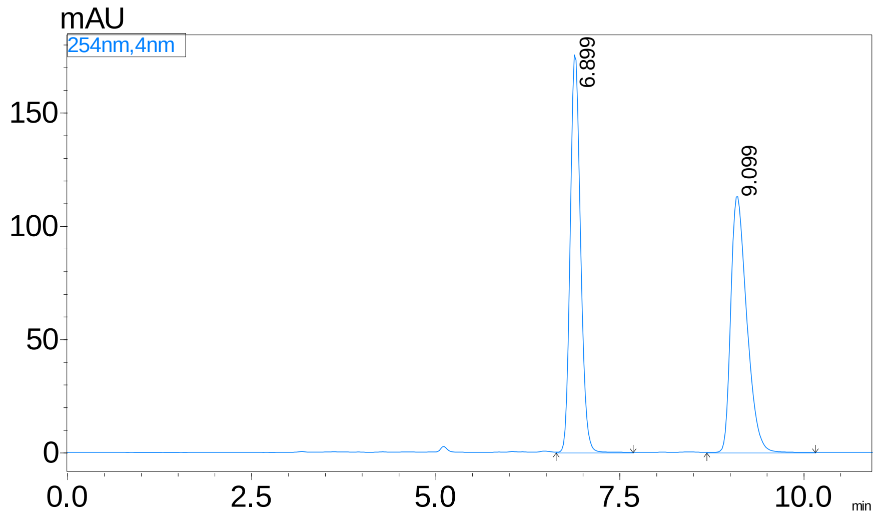

Peak#	Ret.Time	Area	Height	Area%
1	6.899	1704275	175356	49.982
2	9.099	1705495	112827	50.018

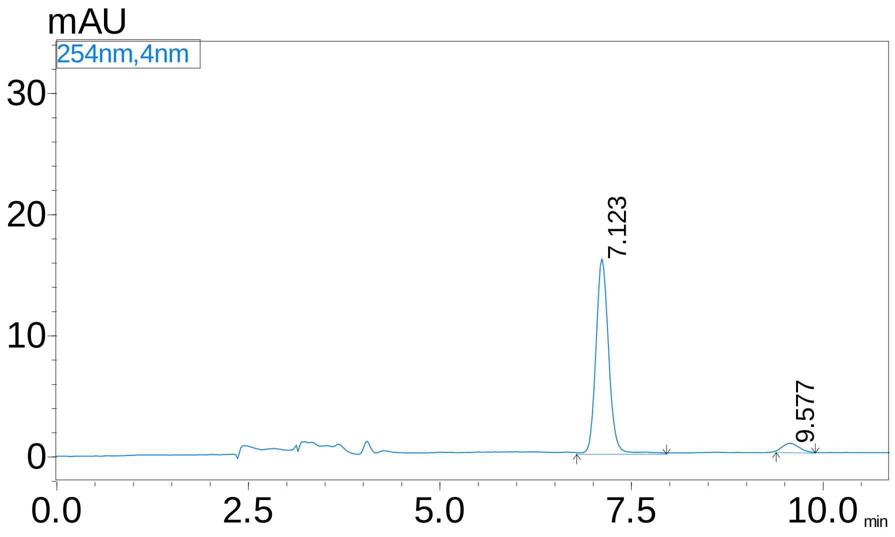

Peak#	Ret.Time	Area	Height	Area%
1	7.123	185446	16066	94.766
2	9.577	10242	705	5.234

Supplementary Figure 84. HPLC spectra of **3le**

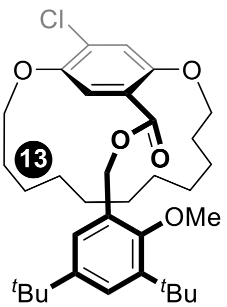

5ae

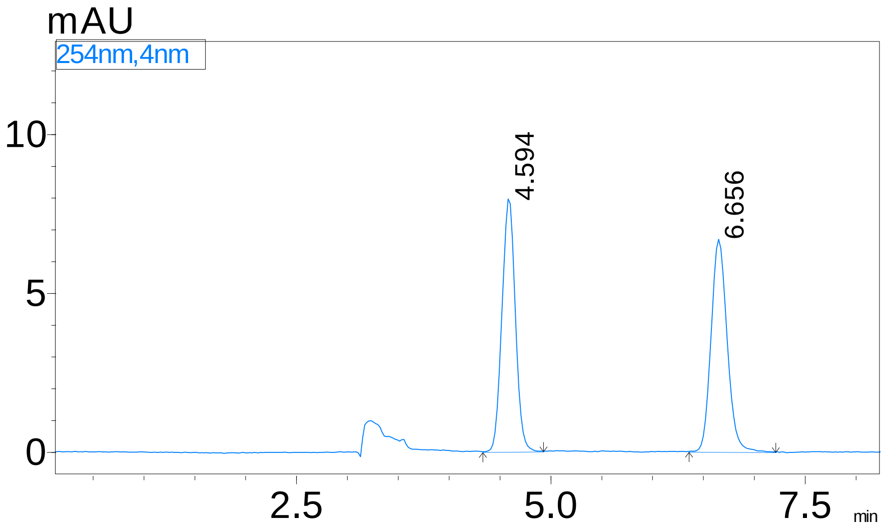

Peak#	Ret.Time	Area	Height	Area%
1	4.594	72857	7948	49.826
2	6.656	73366	6683	50.174

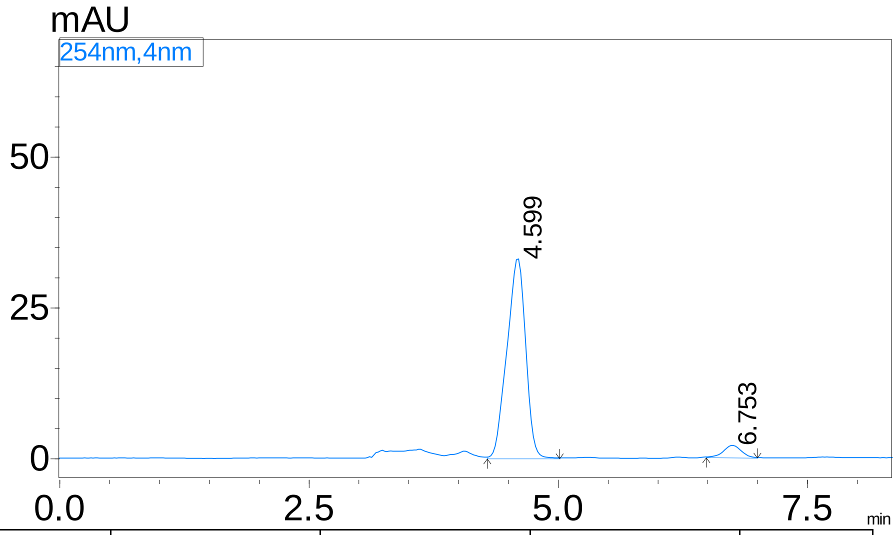

Peak#	Ret.Time	Area	Height	Area%
1	4.599	432896	33041	94.856
2	6.753	23478	1952	5.144

Supplementary Figure 85. HPLC spectra of **5ae**

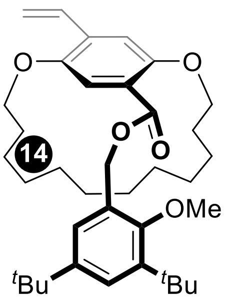

5be

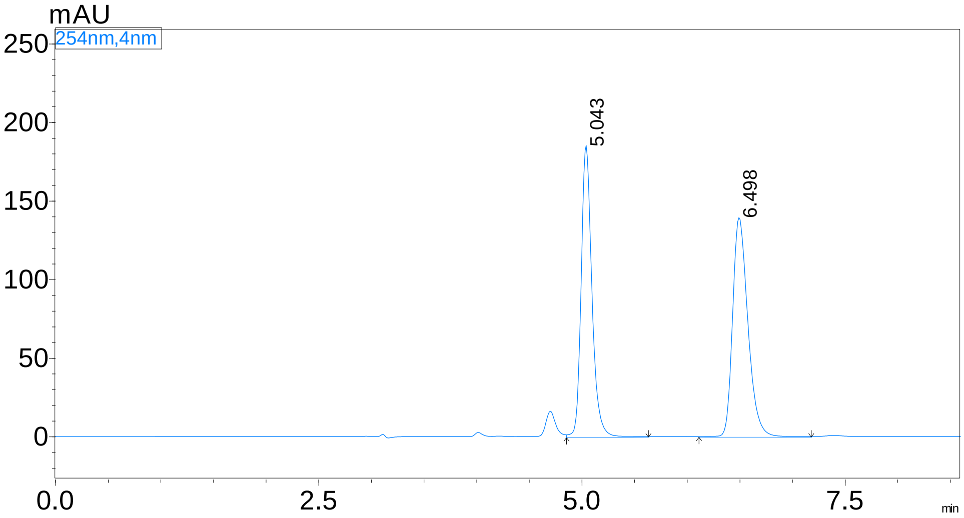

Peak#	Ret.Time	Area	Height	Area%
1	5.043	1315929	185466	48.387
2	6.498	1403677	139381	51.613

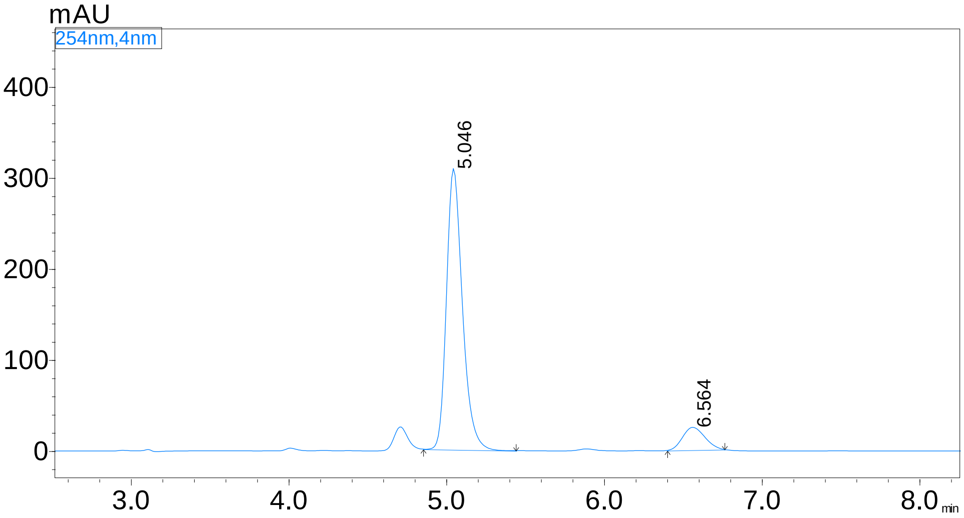

Peak#	Ret.Time	Area	Height	Area%
1	5.046	2170491	308824	89.903
2	6.564	243760	24857	10.097

Supplementary Figure 86. HPLC spectra of **5be**

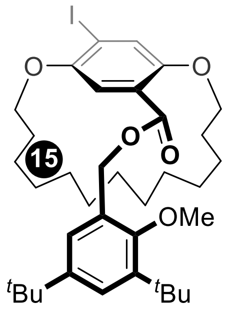

5ce

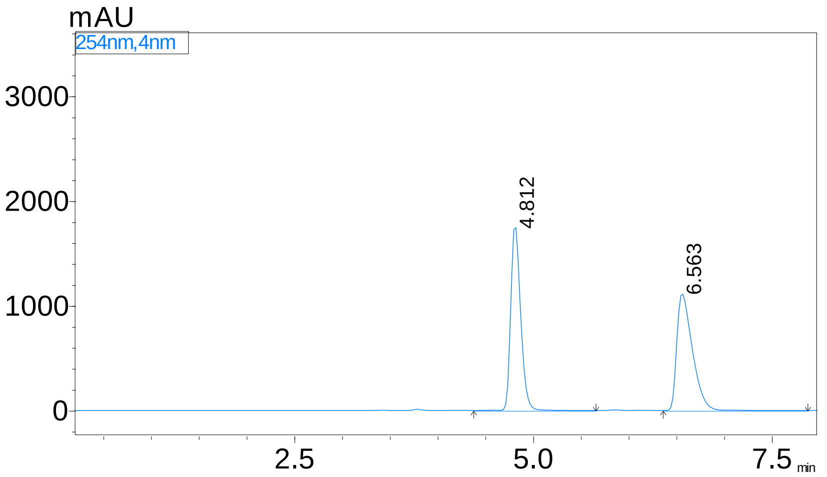

Peak#	Ret.Time	Area	Height	Area%
1	4.812	12735314	1746589	50.132
2	6.563	12668013	1110533	49.868

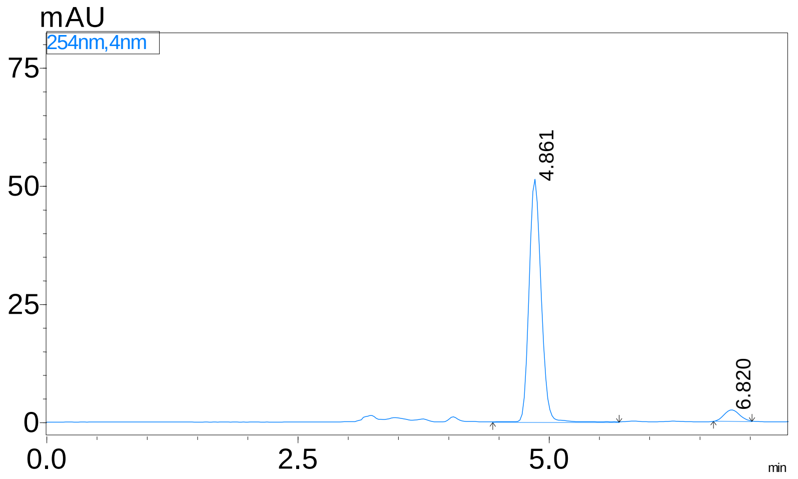

Peak#	Ret.Time	Area	Height	Area%
1	4.861	424030	51316	94.930
2	6.820	22647	2252	5.070

Supplementary Figure 87. HPLC spectra of **5ce**

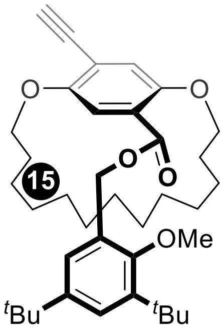

5de

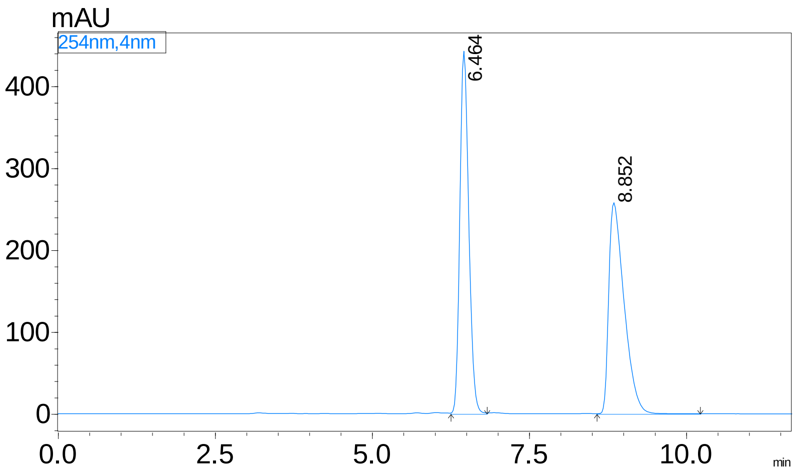

Peak#	Ret.Time	**Area**	**Height**	**Area%**
1	6.464	4375782	442568	49.998
2	8.852	4376127	257775	50.002

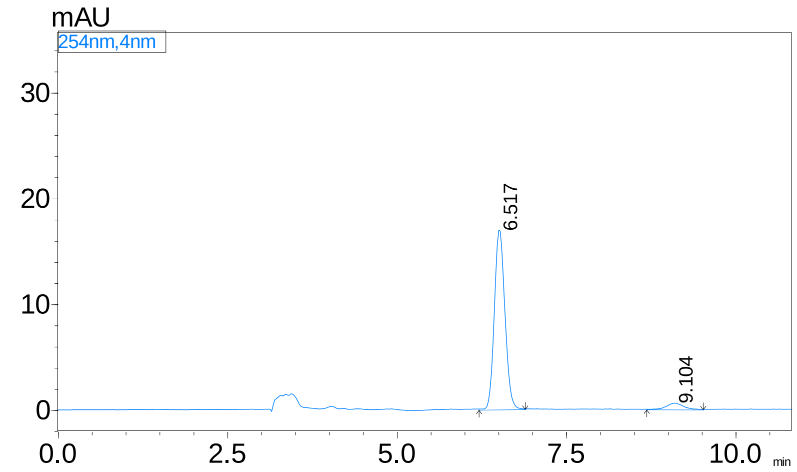

Peak#	Ret.Time	Area	Height	Area%
1	6.517	183571	16923	95.029
2	9.104	9603	586	4.971

Supplementary Figure 88. HPLC spectra of **5de**

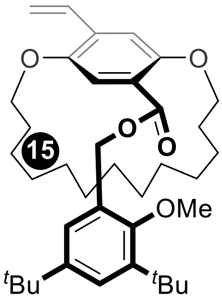

5ee

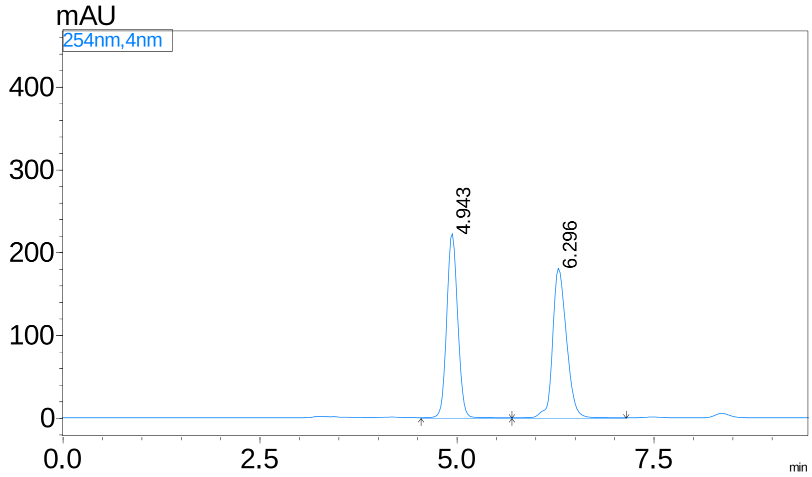

Peak#	Ret.Time	Area	Height	Area%
1	4.943	2157119	222460	49.432
2	6.296	2206676	180829	50.568

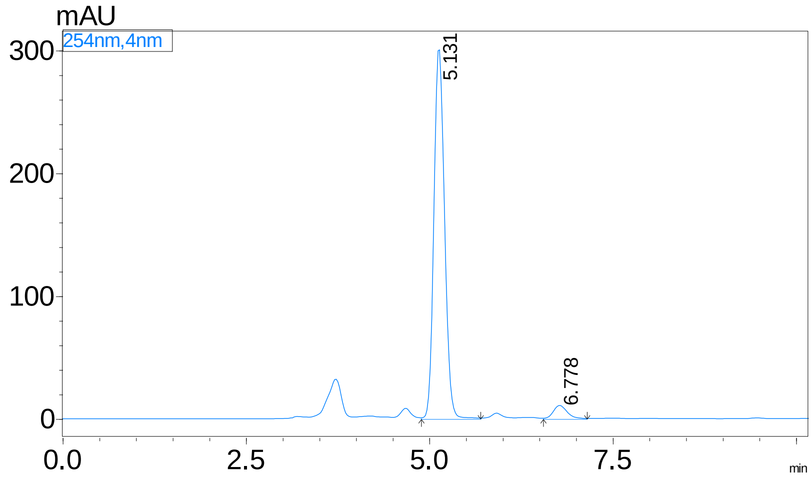

Peak#	Ret.Time	Area	Height	Area%
1	5.131	3037533	300471	95.554
2	6.778	141335	10797	4.446

Supplementary Figure 89. HPLC spectra of **5ee**

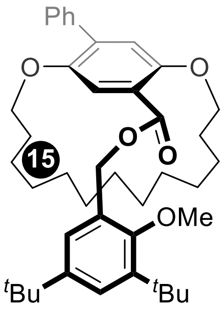

5fe

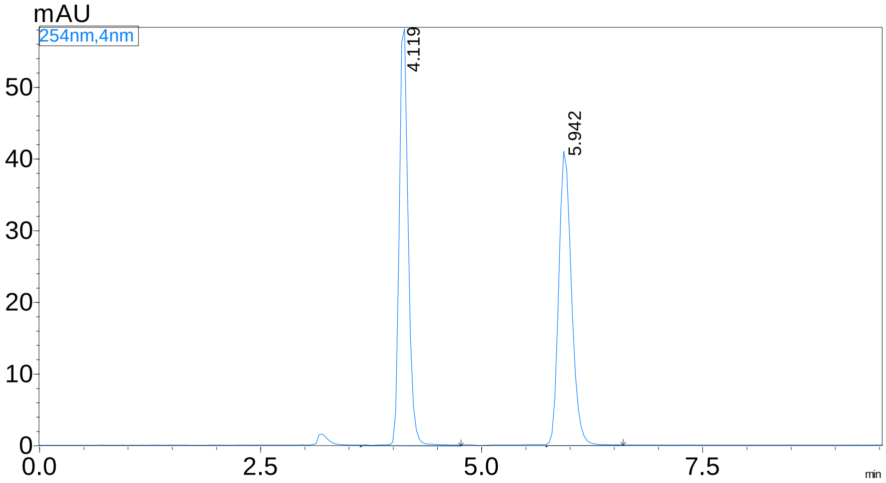

Peak#	Ret.Time	Area	Height	Area%
1	4.119	414805	58127	50.291
2	5.942	410005	40996	49.709

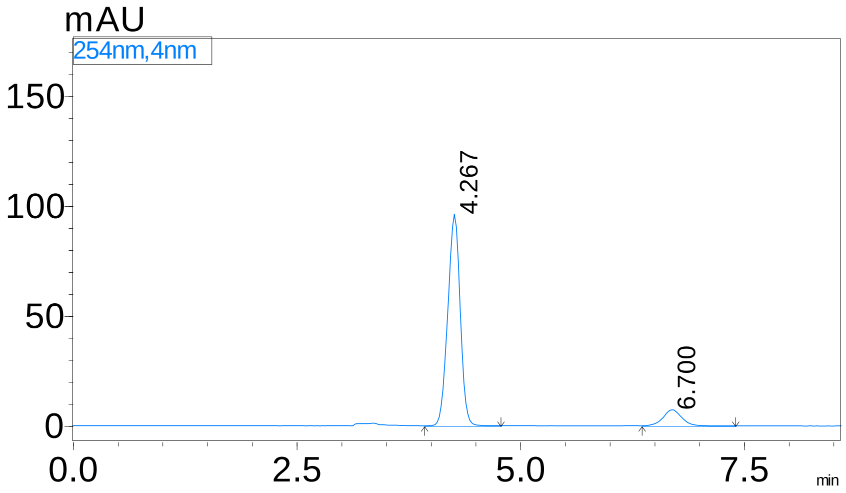

Peak#	Ret.Time	Area	Height	Area%
1	4.267	926630	96227	89.833
2	6.700	104877	7314	10.167

Supplementary Figure 90. HPLC spectra of **5fe**

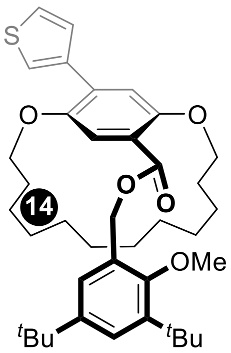

5ge

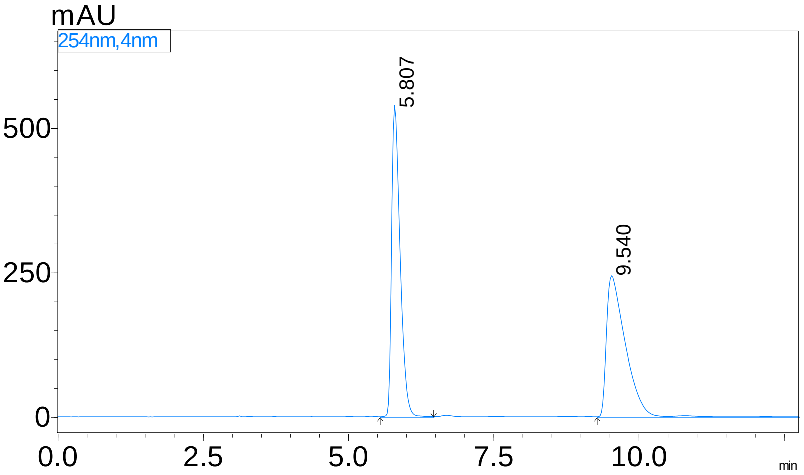

Peak#	Ret.Time	Area	Height	Area%
1	5.807	5483744	538562	49.832
2	9.540	5520757	244117	50.168

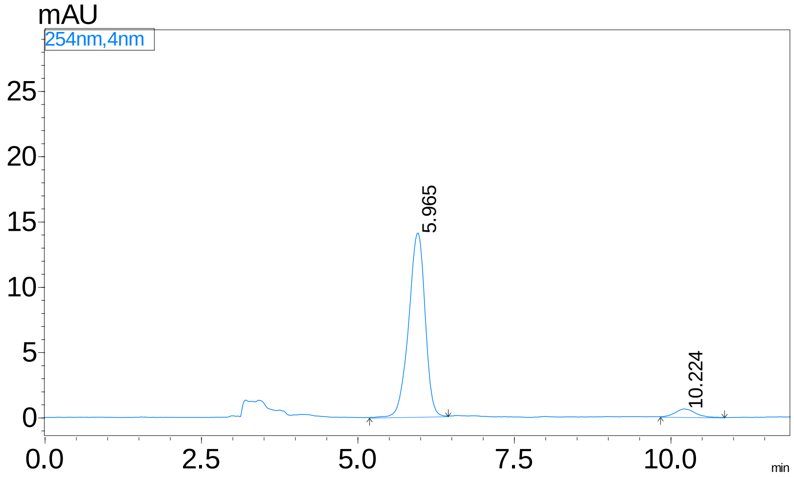

Peak#	Ret.Time	Area	Height	Area%
1	5.965	255794	14066	95.115
2	10.224	13137	611	4.885

Supplementary Figure 91. HPLC spectra of **5ge**

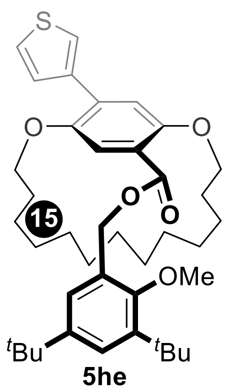

5he

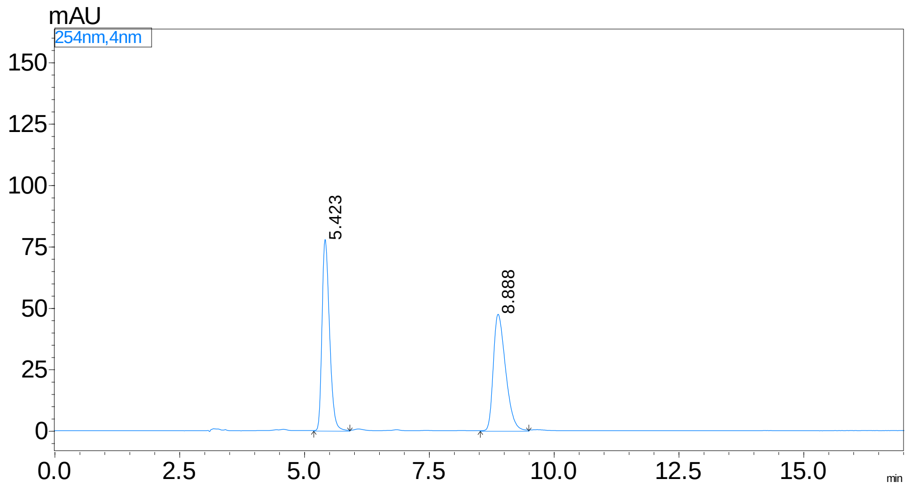

Peak#	Ret.Time	Area	Height	Area%
1	5.423	813206	77758	50.123
2	8.888	809226	47421	49.877

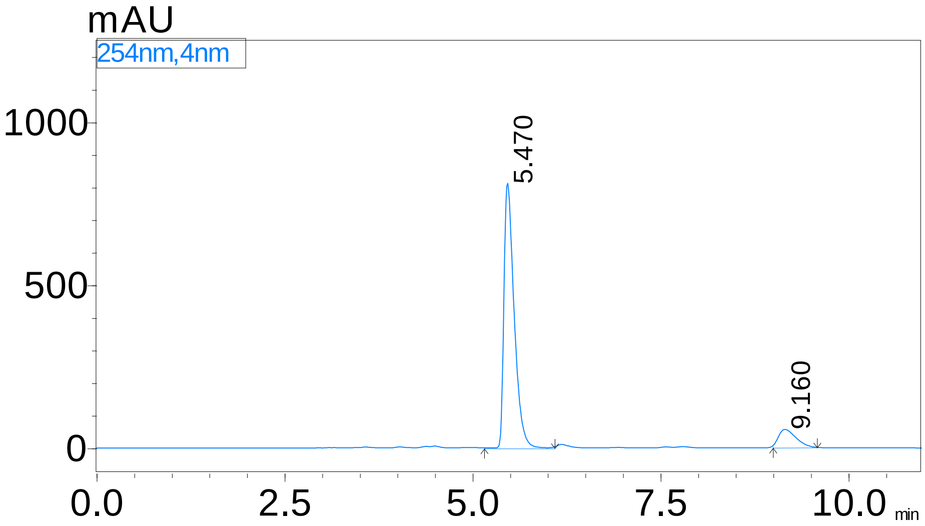

Peak#	Ret.Time	Area	Height	Area%
1	5.470	7676315	811998	89.755
2	9.160	876226	54783	10.245

Supplementary Figure 92. HPLC spectra of **5he**

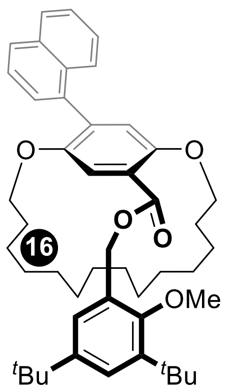

5ie

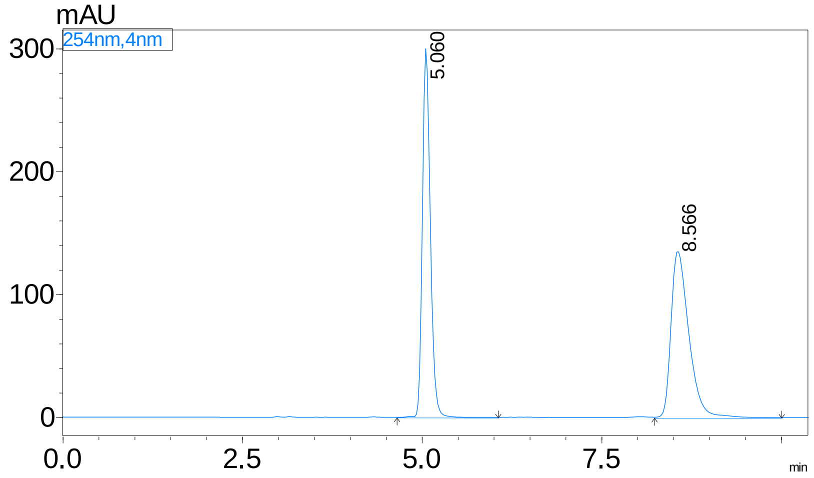

Peak#	Ret.Time	Area	Height	Area%
1	5.060	2297992	300019	49.712
2	8.566	2324633	134888	50.288

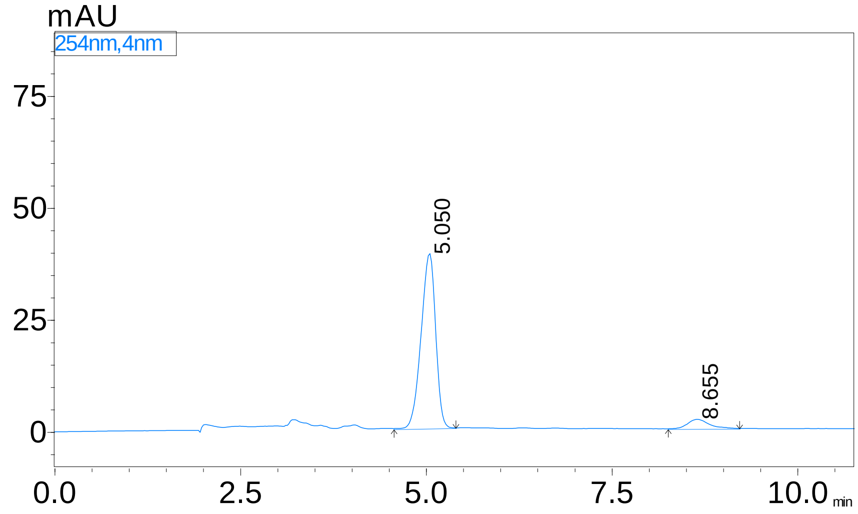

Peak#	Ret.Time	Area	Height	Area%
1	5.050	531208	39016	92.819
2	8.655	41100	2079	7.181

Supplementary Figure 93. HPLC spectra of **5ie**

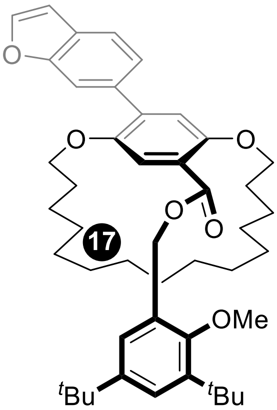

5je

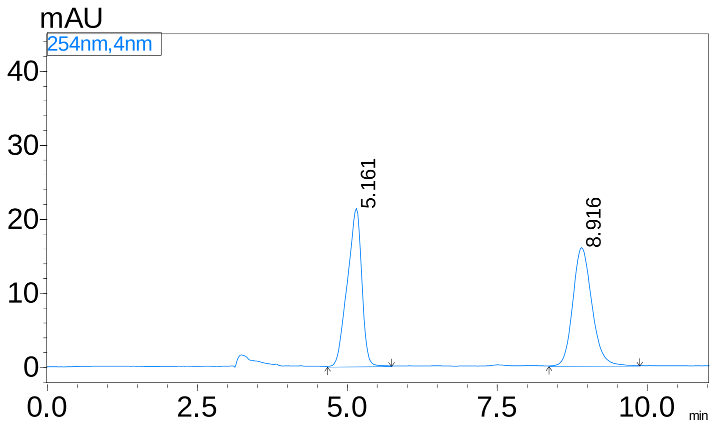

Peak#	Ret.Time	Area	Height	Area%
1	5.161	353253	21335	50.131
2	8.916	351411	15969	49.869

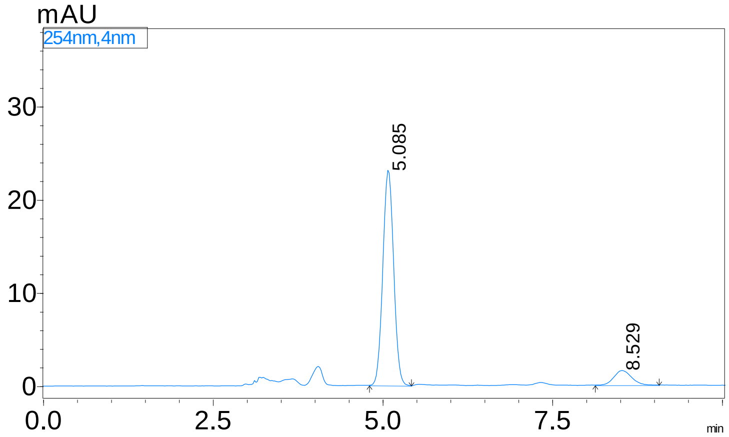

Peak#	Ret.Time	Area	Height	Area%
1	5.085	256506	23106	90.162
2	8.529	27987	1563	9.838

Supplementary Figure 94. HPLC spectra of **5je**

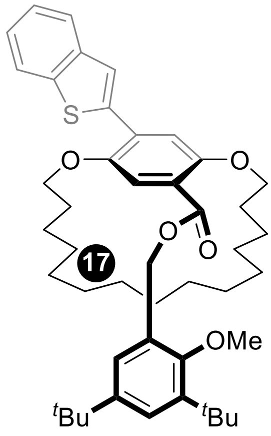

5ke

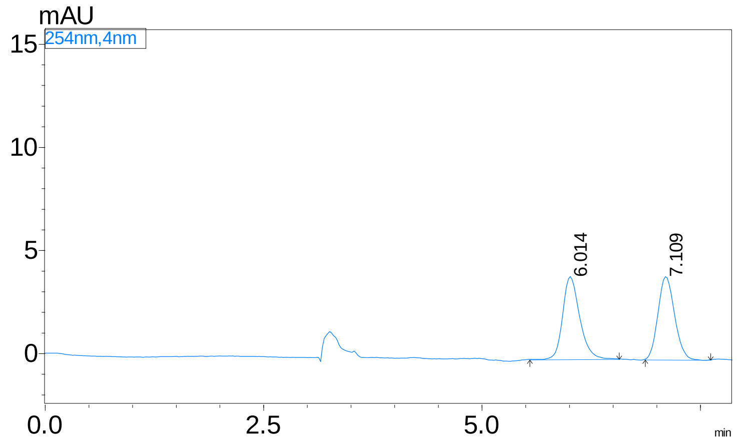

Peak#	Ret.Time	Area	Height	Area%
1	6.014	53910	4006	50.619
2	7.109	52590	4015	49.381

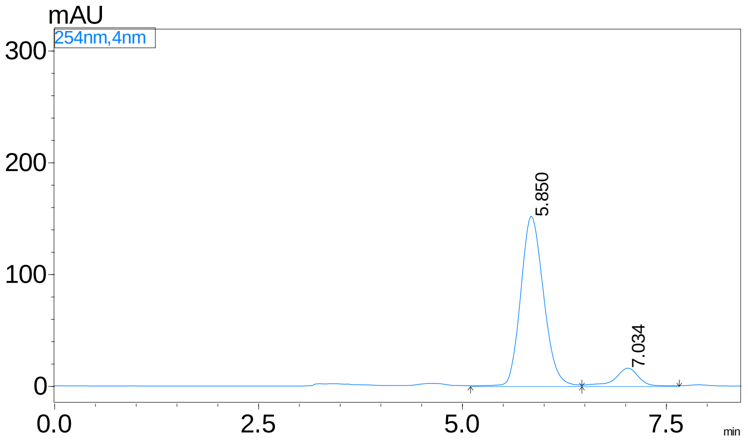

Peak#	Ret.Time	Area	Height	Area%
1	5.850	2952459	151957	90.287
2	7.034	317614	159961	9.713

Supplementary Figure 95. HPLC spectra of **5ke**

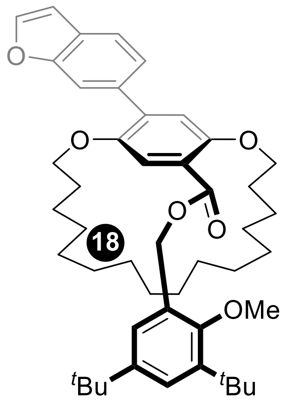

5le

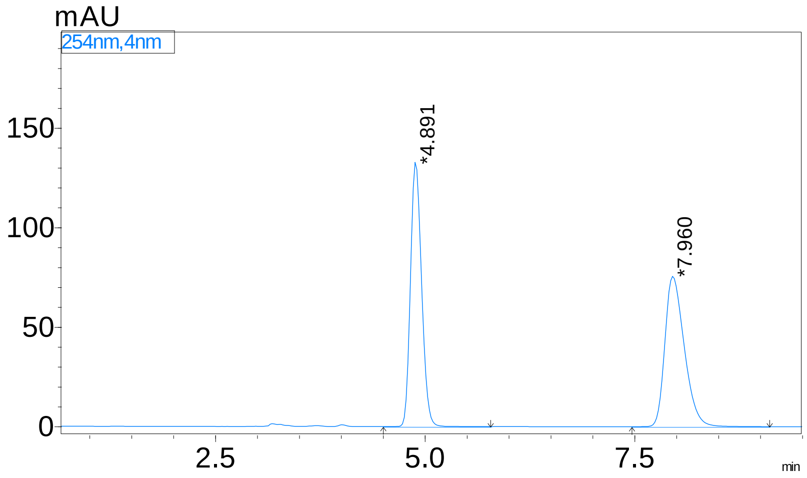

Peak#	Ret.Time	Area	Height	Area%
1	4.891	1222803	132777	50.058
2	7.960	1219955	75544	49.942

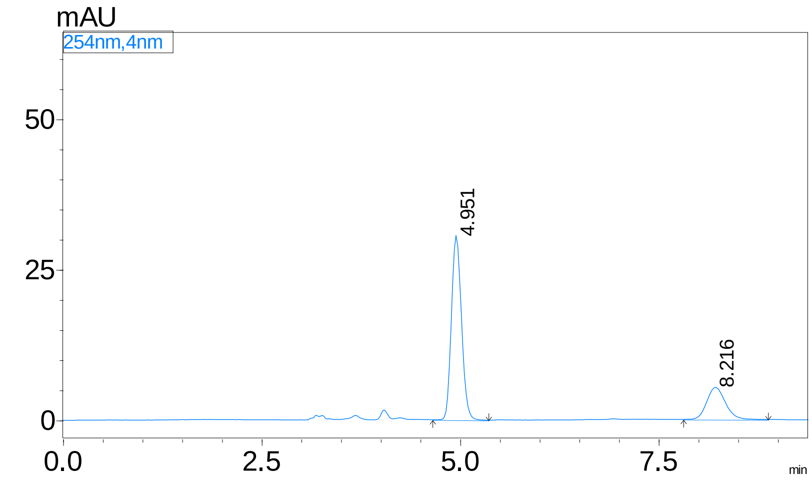

Peak#	Ret.Time	Area	Height	Area%
1	4.951	270263	30604	75.783
2	8.216	86363	5322	24.217

Supplementary Figure 96. HPLC spectra of **5le**

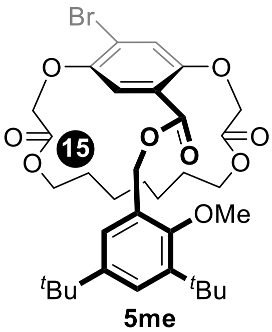

5me

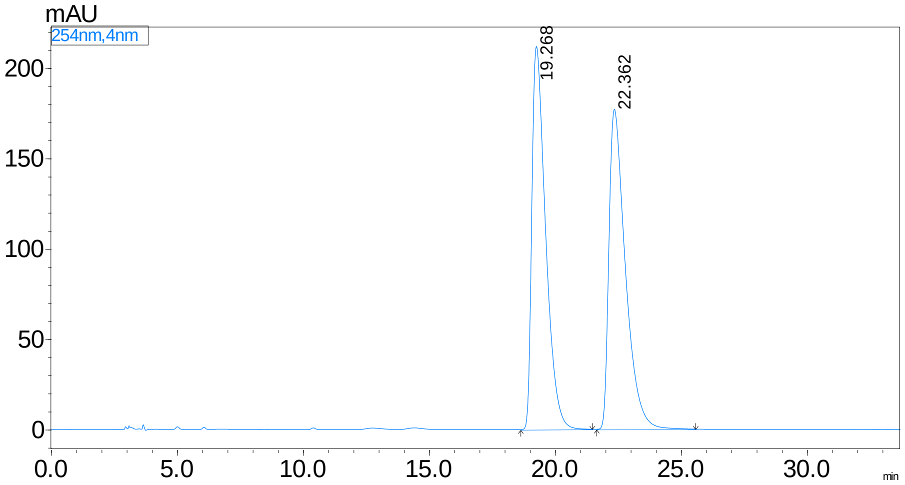

Peak#	Ret.Time	Area	Height	Area%
1	19.268	8048675	211955	49.990
2	22.362	8051961	176916	50.010

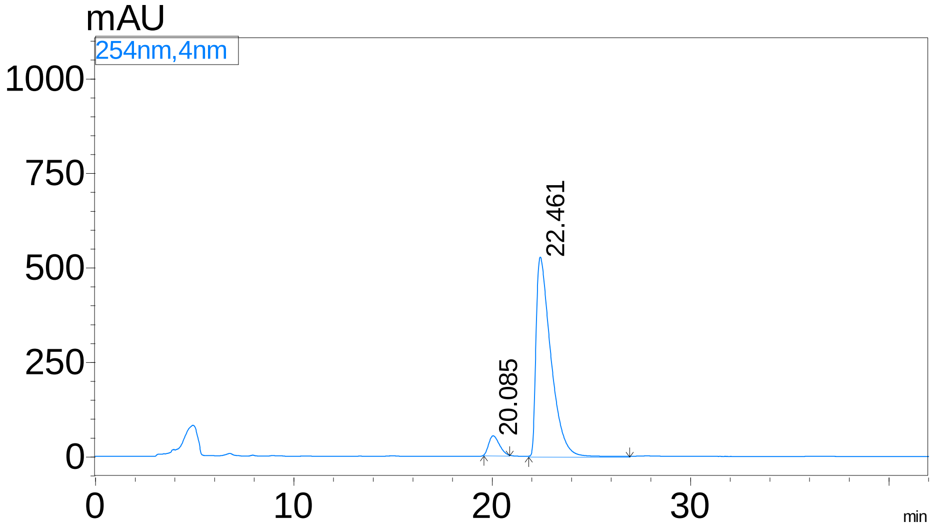

Peak#	Ret.Time	Area	Height	Area%
1	20.085	1886203	51348	6.471
2	22.461	27261427	527306	93.529

Supplementary Figure 97. HPLC spectra of **5me**

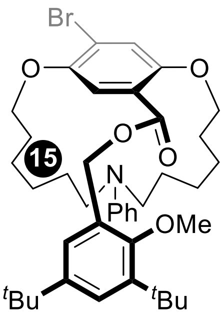

5ne

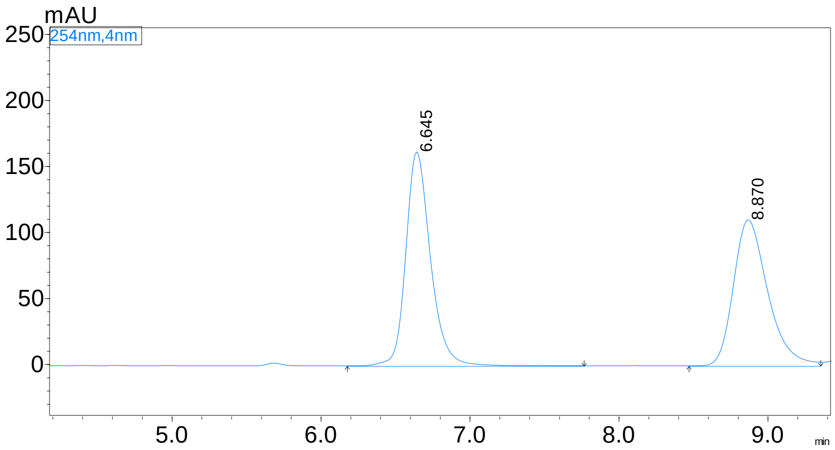

Peak#	Ret.Time	Area	Height	Area%
1	6.645	1862365	162079	51.251
2	8.870	1771457	110455	48.749

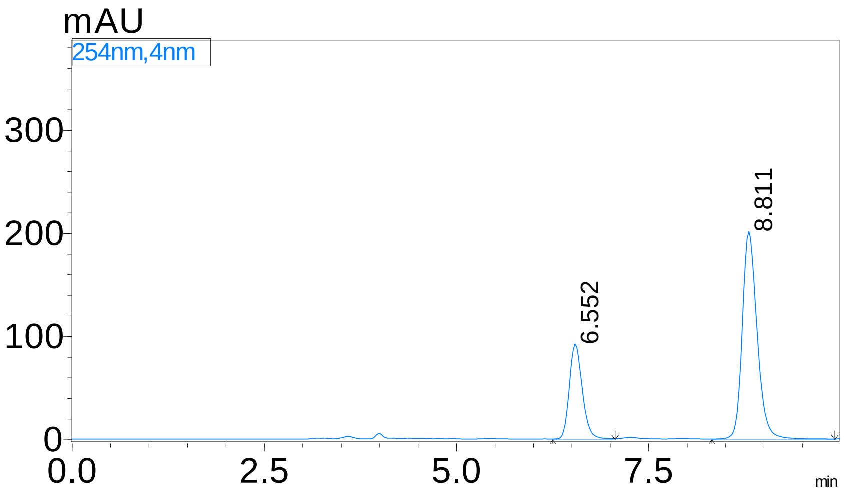

Peak#	Ret.Time	Area	Height	Area%
1	6.552	1079343	91989	28.616
2	8.811	2692416	201268	71.384

Supplementary Figure 98. HPLC spectra of **5ne**

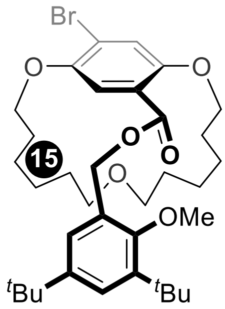

5oe

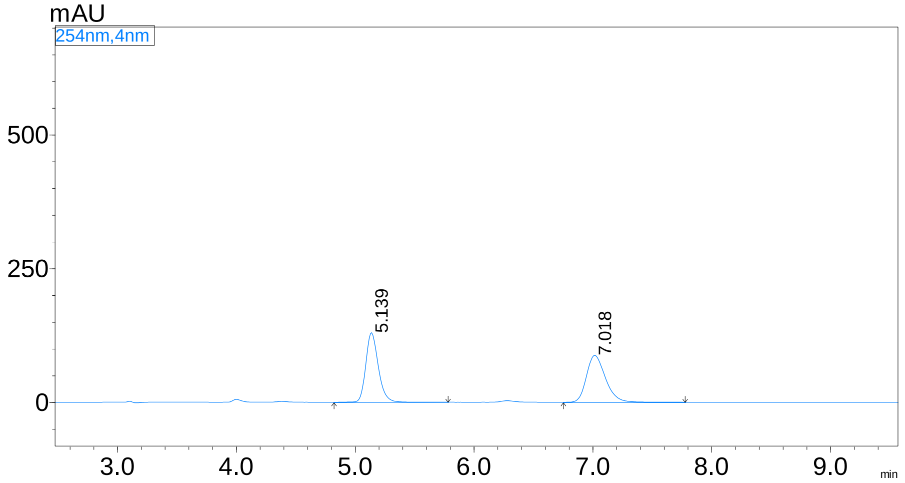

Peak#	Ret.Time	Area	Height	Area%
1	5.139	950057	130065	49.544
2	7.018	967546	87625	50.456

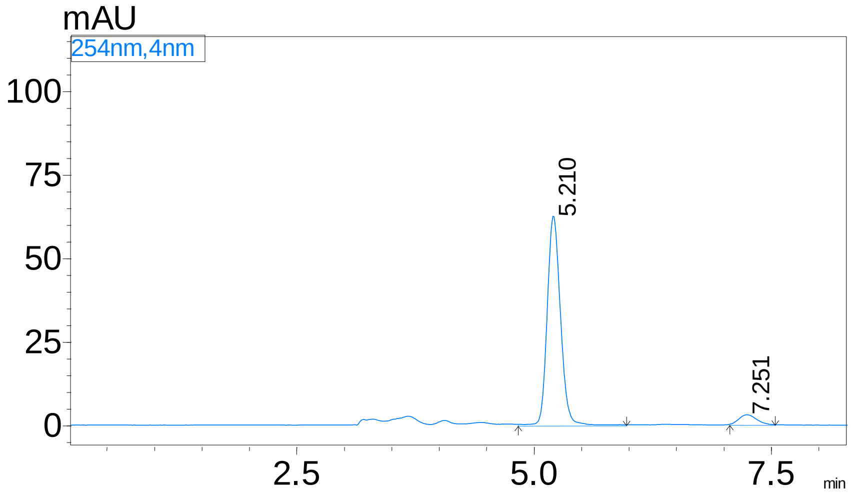

Peak#	Ret.Time	Area	Height	Area%
1	5.210	607953	62578	94.209
2	7.251	37373	2935	5.791

Supplementary Figure 99. HPLC spectra of **5oe**

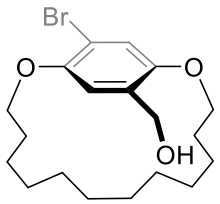

6

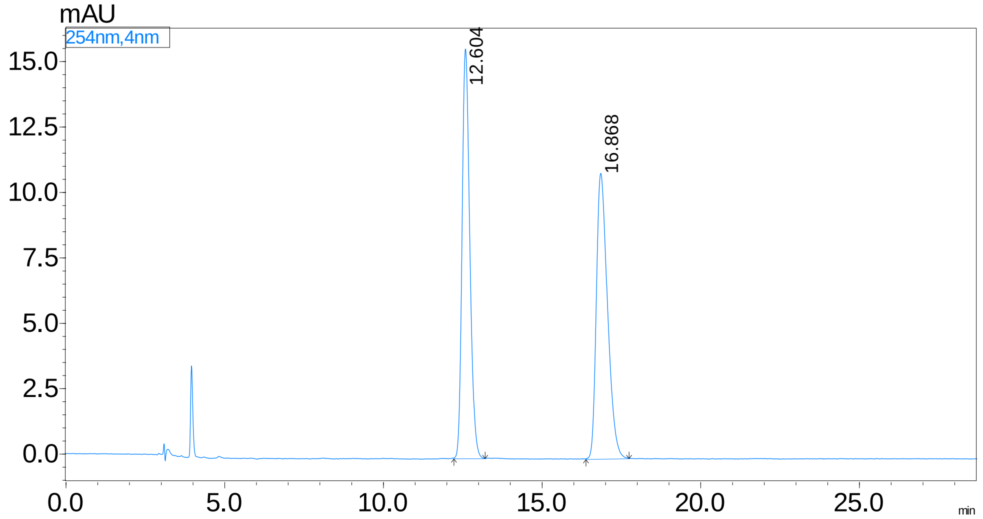

Peak#	Ret.Time	Area	Height	Area%
1	12.604	265968	15646	49.958
2	16.868	266410	10906	50.042

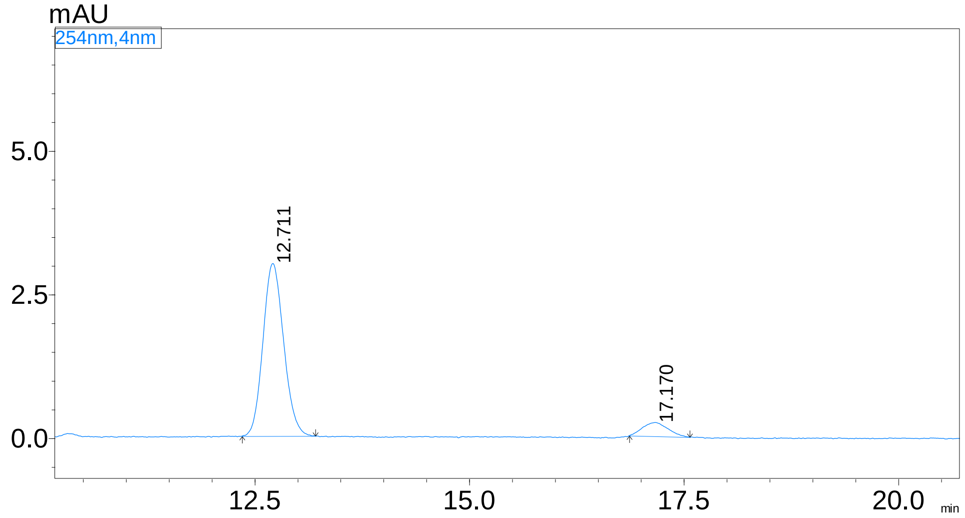

Peak#	Ret.Time	Area	Height	Area%
1	12.711	50422	3003	91.113
2	17.170	4918	240	8.887

Supplementary Figure 100. HPLC spectra of **6**

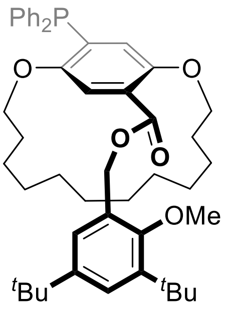

7

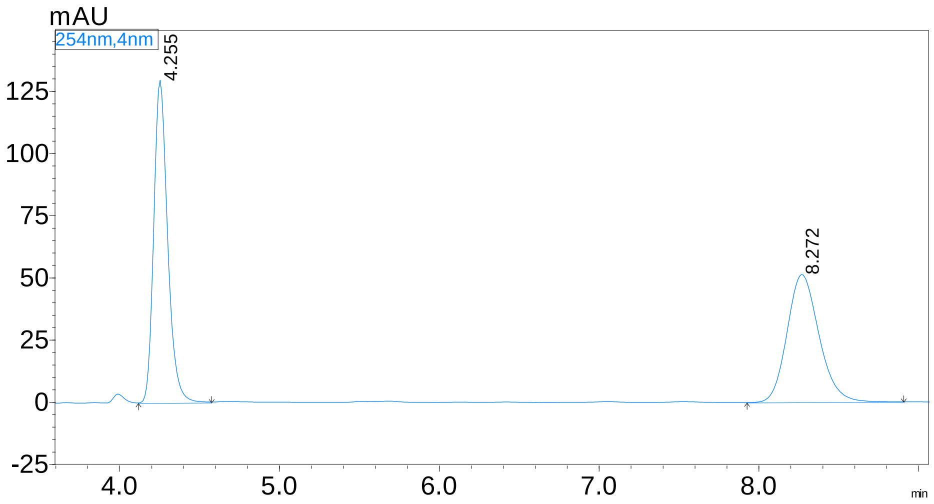

Peak#	Ret.Time	Area	Height	Area%
1	4.255	784131	129740	52.376
2	8.272	712974	51294	47.624

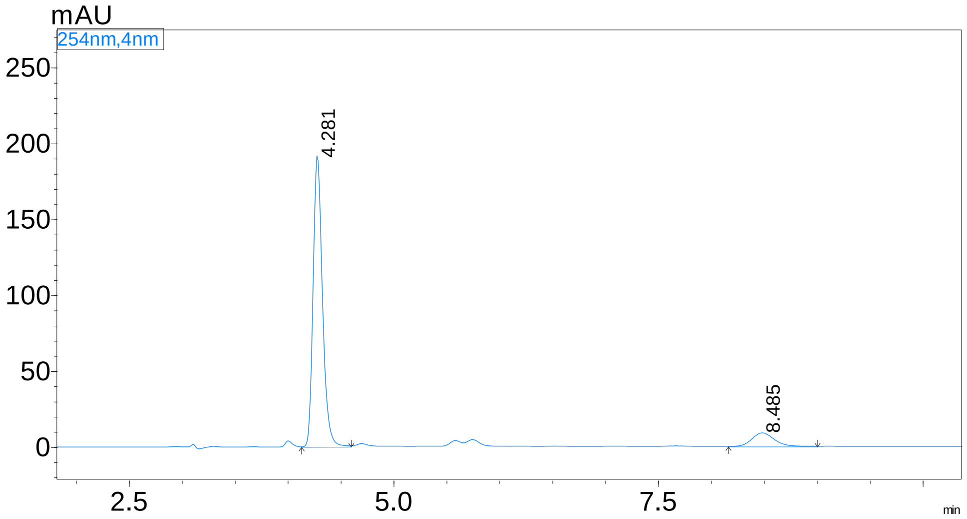

Peak#	Ret.Time	Area	Height	Area%
1	4.281	1177407	191407	90.215
2	8.485	127709	8872	9.785

Supplementary Figure 101. HPLC spectra of **7**

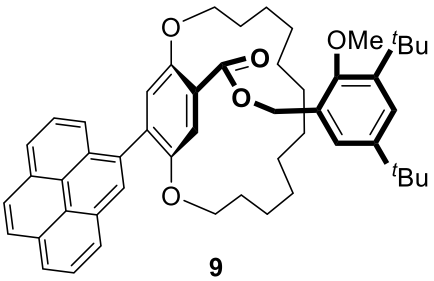

9

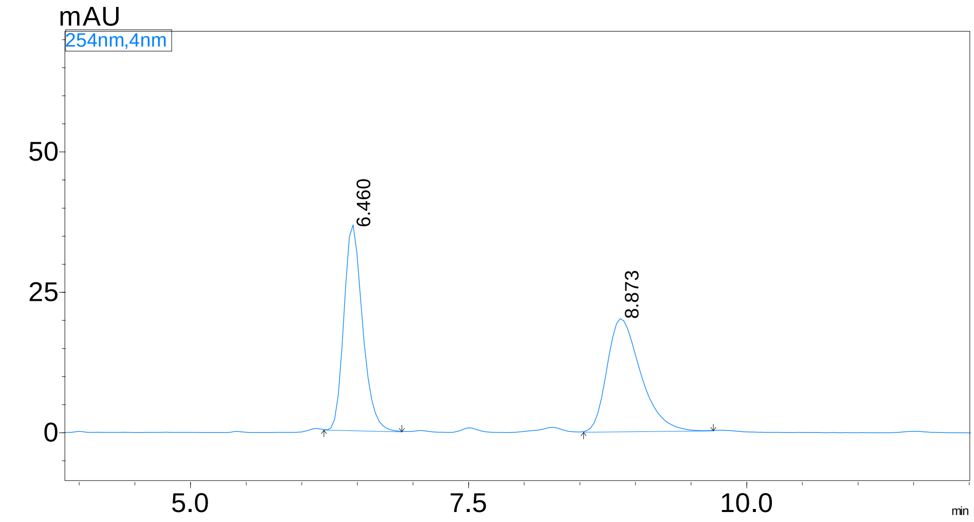

Peak#	Ret.Time	Area	Height	Area%
1	6.460	425033	36558	50.368
2	8.873	418829	20076	49.632

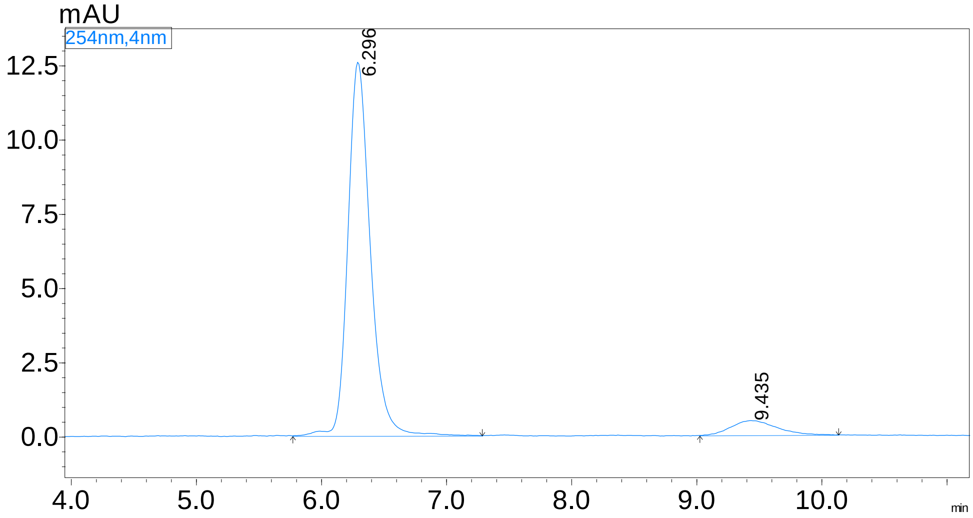

Peak#	Ret.Time	Area	Height	Area%
1	6.296	157073	12580	92.808
2	9.435	12173	489	7.192

Supplementary Figure 102. HPLC spectra of **9**

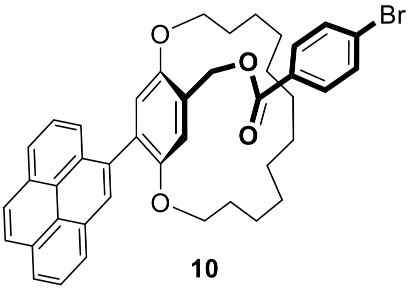

10

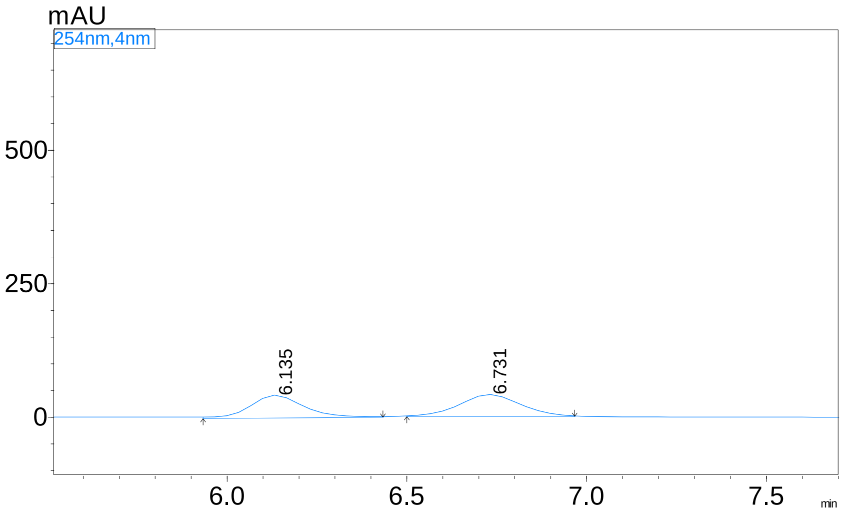

Peak#	Ret.Time	Area	Height	Area%
1	6.135	419739	41950	47.471
2	6.731	464456	40161	52.529

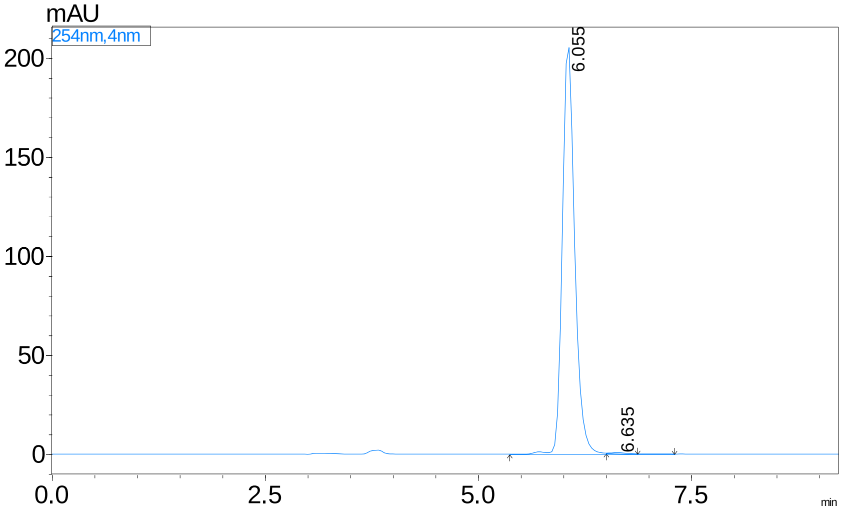

Peak#	Ret.Time	Area	Height	Area%
1	6.135	419739	41950	99.845
2	6.731	464456	40161	0.155

Supplementary Figure 103. HPLC spectra of **10**

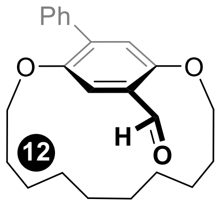

11

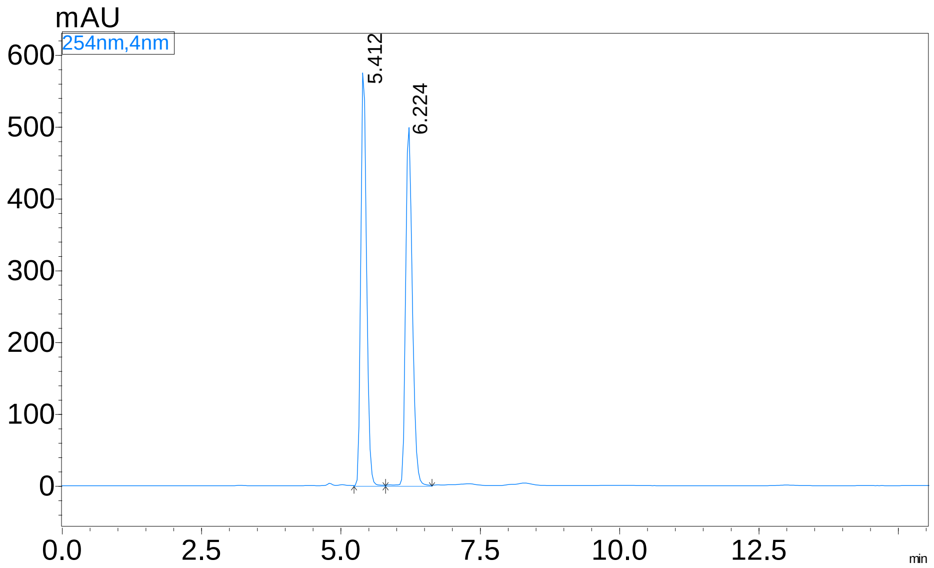

Peak#	Ret.Time	Area	Height	Area%
1	5.412	4163785	574720	49.796
2	6.224	4197840	498781	50.204

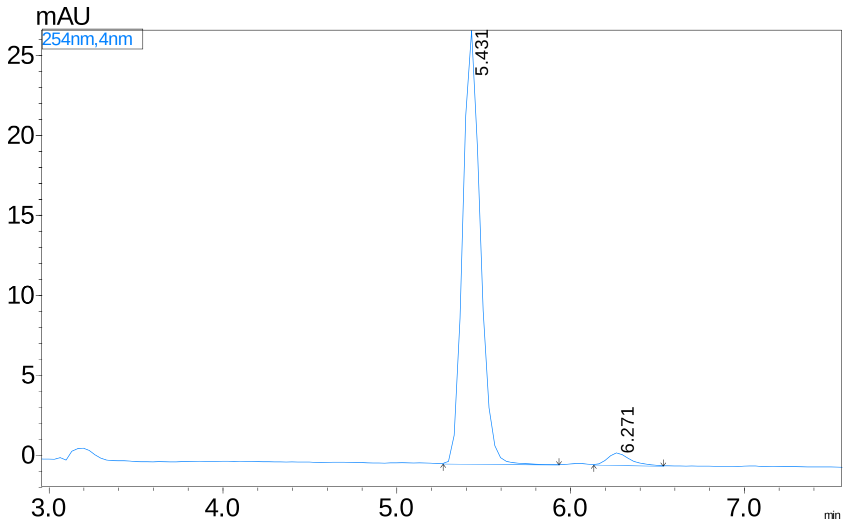

Peak#	Ret.Time	Area	Height	Area%
1	5.431	189468	27076	96.753
2	6.271	6358	748	3.247

Supplementary Figure 104. HPLC spectra of **11**

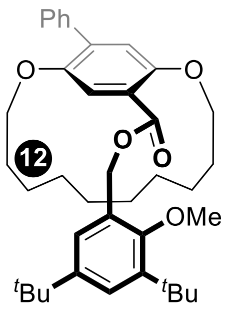

12

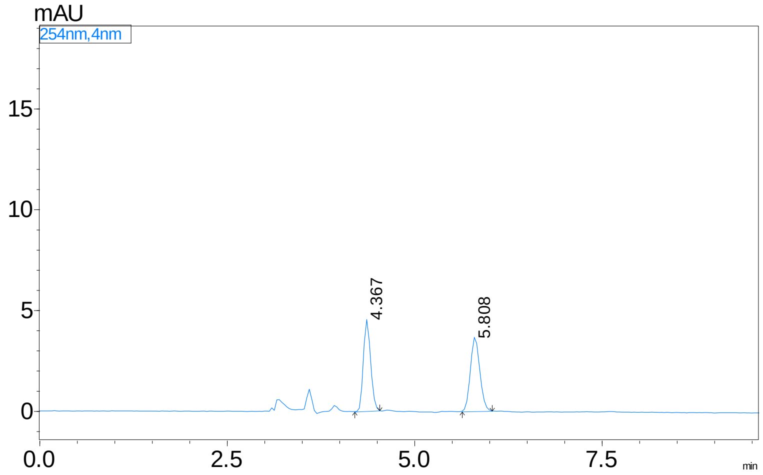

Peak#	Ret.Time	Area	Height	Area%
1	4.367	30309	4539	48.404
2	5.808	32307	3663	51.596

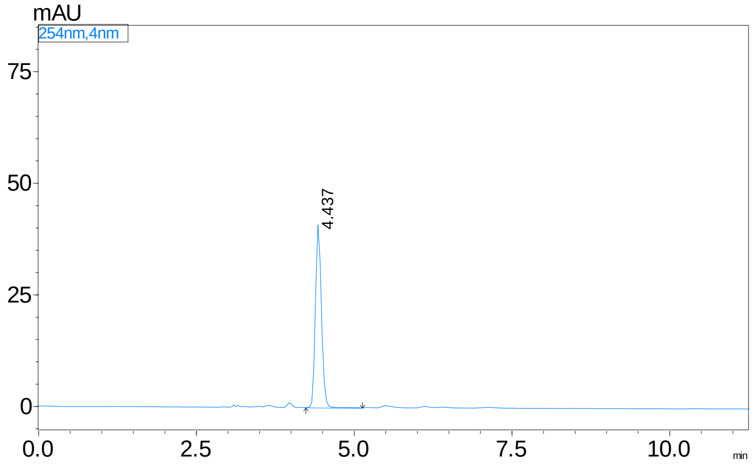

Peak#	Ret.Time	Area	Height	Area%
1	4.437	270732	40914	100.000

Supplementary Figure 105. HPLC spectra of **12**

3. Supplementary References

[1] Kaliyaperumal, S. A., Banerjee, S. & U. K, S. K. Palladium mediated intramolecular multiple C-X/C–H cross coupling and C–H activation: Synthesis of carbazole alkaloids calothrixin B and murrayaquinone A. *Org. Biomol. Chem.* **12**, 6105-6113, (2014).

[2] Kanda, K., Koike, T., Endo, K. & Shibata, T. The first asymmetric Sonogashira coupling for the enantioselective generation of planar chirality in paracyclophanes. *Chem. Commun*. 1870-1872, (2009).

[3] Voll, A. M. et al. Macrocyclic fkbp51 ligands define a transient binding mode with enhanced selectivity. *Angew. Chem. Int. Ed.* **60**, 13257-13263, (2021).

[4] Nag, O. K. et al. Two-photon absorption properties of cationic 1,4-bis(styryl)benzene derivative and its inclusion complexes with cyclodextrins. *J. Phy. Chem B* **114**, 9684-9690, (2010).

[5] Diethelm, S., Schindler, C. S. & Carreira, E. M. Access to the aeruginosin serine protease inhibitors through the nucleophilic opening of an oxabicyclo[2.2.1]heptane: Total synthesis of microcin SF608. *Chem. Eur. J.* **20**, 6071-6080, (2014).

[6] Frisch, M. J. et al. Fox, Gaussian 09, rev. B. 01; Gaussian. Inc.: Wallingford, CT, (2009).

[7] Becke, A. D. Density-functional thermochemistry. III. The role of exact exchange. *J. Chem. Phys.* **98**, 5648-5652, (1993).

[8] Lee, C., Yang, W. & Parr, R. G. Development of the colle-salvetti correlation-energy formula into a functional of the electron density. *Phys. Rev. B.* **37**, 785-789, (1988).

[9] Grimme, S., Antony, J., Ehrlich, S. & Krieg, H. A Consistent and accurate ab initio parametrizatio of density functional dispersion correction (DFT-D) for the 94 elements H-Pu. *J. Chem. Phys.* **132**, 154104, (2010).

[10] Petersson, G. A. et al. "A complete basis set model chemistry. I. The total energies of closed-shell atoms and hydrides of the first-row atoms," *J. Chem. Phys.* **89**, 2193-2218, (1988).

[11] Zhao, Y. & Truhlar, D. G. "The M06 suite of density functionals for main group thermochemistry, thermochemical kinetics, noncovalent interactions, excited states, and transition elements: two new functionals and systematic testing of four M06-class functionals and 12 other functionals," *Theor. Chem. Account.* **120***,* 215-241, (2008).
